# Construction and validation of a colon cancer prognostic model based on tumor mutation burden-related genes

**DOI:** 10.1038/s41598-024-53257-z

**Published:** 2024-02-04

**Authors:** Daoyang Zou, Tianwen Xu

**Affiliations:** The Second Affiliated Hospital of Fujian Medical University, Quanzhou, China

**Keywords:** Cancer, Computational biology and bioinformatics, Immunology, Biomarkers, Oncology

## Abstract

Currently, immunotherapy has entered the clinical diagnosis and treatment guidelines for colon cancer, but existing immunotherapy markers cannot predict the effectiveness of immunotherapy well. This study utilized the TCGA-COAD queue to perform differential gene analysis on high and low-mutation burden samples, and screen differentially expressed genes (DEGs). To explore new molecular markers or predictive models of immunotherapy by using DEGs for NMF classification and prognostic model construction. Through systematic bioinformatics analysis, the TCGA-COAD cohort was successfully divided into high mutation burden subtypes and low mutation burden subtypes by NMF typing using DEGs. The proportion of MSI-H between high mutation burden subtypes was significantly higher than that of low mutation burden subtypes, but there was no significant difference in immunotherapy efficacy between the two subtypes. Drug sensitivity analysis showed significant differences in drug sensitivity between the two subtypes. Subsequently, we constructed a prognostic model using DEGs, which can effectively predict patient survival and immunotherapy outcomes. The prognosis and immunotherapy outcomes of the low-risk group were significantly better than those of the high-risk group. The external dataset validation of the constructed prognostic model using the GSE39582 dataset from the GEO database yielded consistent results. At the same time, we also analyzed the TMB and MSI situation between the high and low-risk groups, and the results showed that there was no significant difference in TMB between the high and low-risk groups, but the proportion of MSI-H in the high-risk group was significantly higher than that in the low-risk group. Finally, we conclude that TMB is not a suitable molecular marker for predicting the efficacy of immunotherapy in colon cancer. The newly constructed prognostic model can effectively differentiate the prognosis of colon cancer patients and predict their immunotherapy efficacy.

## Introduction

Globally, colorectal cancer (CRC) is the third leading malignant tumor with the second highest incidence rate and mortality^1^. According to the prediction data of the World Cancer Research Fund, it is estimated that in 2020, new cases will be 1,931,590, and death cases will be 935,173 (https://www.wcrf.org/cancer-trends/colorectal-cancer-statistics/), which will seriously threaten human health. Colon adenocarcinoma (COAD) is the most common histological subtype of CRC^1^. Although the American Joint Commission on Cancer (AJCC) staging can be used to evaluate the prognosis of COAD patients, overall survival (OS) and disease-free survival (DFS) are not always associated with tumor staging^2^. Currently, microsatellite instability (MSI), BRAF, and RAS mutation states have been further applied in clinical practice to further differentiate the prognosis of CRC patients^3–6^. Although these molecular markers can generally better predict prognosis and drug response, clinical heterogeneity always exists, so it is particularly important to find reliable molecular markers or prognostic models for guiding clinical practice.

Currently, many clinical studies have shown the feasibility of immune checkpoint inhibitors in the treatment of colorectal cancer. Based on the results of the Keynote-177 study, pembrolizumab has been approved for the treatment of MSI-H(MicroSatellite Instability-High)/dMMR(MisMatch Repair-deficient) in colorectal cancer patients^7^. The CheckMate-142 study also demonstrates the feasibility of combining nivolumab with Ipilimumab in the treatment of MSI-H/dMMR colorectal cancer^8^, and has entered clinical diagnosis and treatment guidelines. In the immunotherapy of colorectal cancer, existing studies often use MSI-H/dMMR as a biomarker to predict treatment efficacy^9–11^, but overall only predict the efficacy of some patients. In the Keynote-177 study, the overall effective rate of immunotherapy was 43%^12^, and in the CheckMate-142 study, the overall effective rate was 65%^13^. Therefore, there is an urgent need for more accurate molecular biomarkers in clinical practice to predict the clinical efficacy of immunotherapy.

Studies have shown that approximately 80% of sporadic dMMR colorectal cancer cases are caused by methylation of the *MLH1* gene promoter, while over 70% of genetic cases are related to germline mutations in the *MLH1* and *MSH2* genes^7^. Methylation and mutation of dMMR-related genes result in cells being unable to recognize and repair spontaneous mutations, leading to a significant increase in tumor mutation burden (TMB) and altered microsatellite sequences, these tumors exhibit high microsatellite instability^14–16^. Meanwhile, research has shown that DNA mismatch repair defects tumors are sensitive to immune checkpoint inhibitors because the high mutation burden of dMMR tumors leads to a large number of mutated new antigens on major histocompatibility complex(MHC) molecules, making these cancer cells highly recognized by T cells^10^. Studies have shown the feasibility of using TMB to predict immune therapy response^17,18^. Based on previous research, we speculate that tumor mutation burden may be a potential biological marker for predicting the clinical efficacy of immunotherapy in colorectal cancer.

Therefore, this study intends to use the TCGA-COAD queue to group the samples according to mutation burden (low (1–5 mutations/Mb), medium (6–19 mutations/Mb), high (≥ 20 mutations/Mb))^19^, Differential gene analysis will be conducted between the low and high mutation burden groups to obtain mutation burden related DEGs. Non-negative matrix Factorization (NMF) will be performed on colon adenocarcinoma samples based on the obtained DEGs, Evaluate the immune microenvironment and immune cell infiltration among different mutation burden subgroups, and then evaluate the differences in immunotherapy efficacy and drug sensitivity among different mutation burden subgroups to explore the feasibility of predicting immunotherapy with tumor mutation burden in colon adenocarcinoma. At the same time, a prognostic model was constructed using DEGs, and survival analysis, immune microenvironment analysis, immunotherapy effect prediction, and drug sensitivity analysis were performed on the constructed model. Finally, the obtained prognostic model was validated using the GEO dataset.

## Method

**Figure 1:**
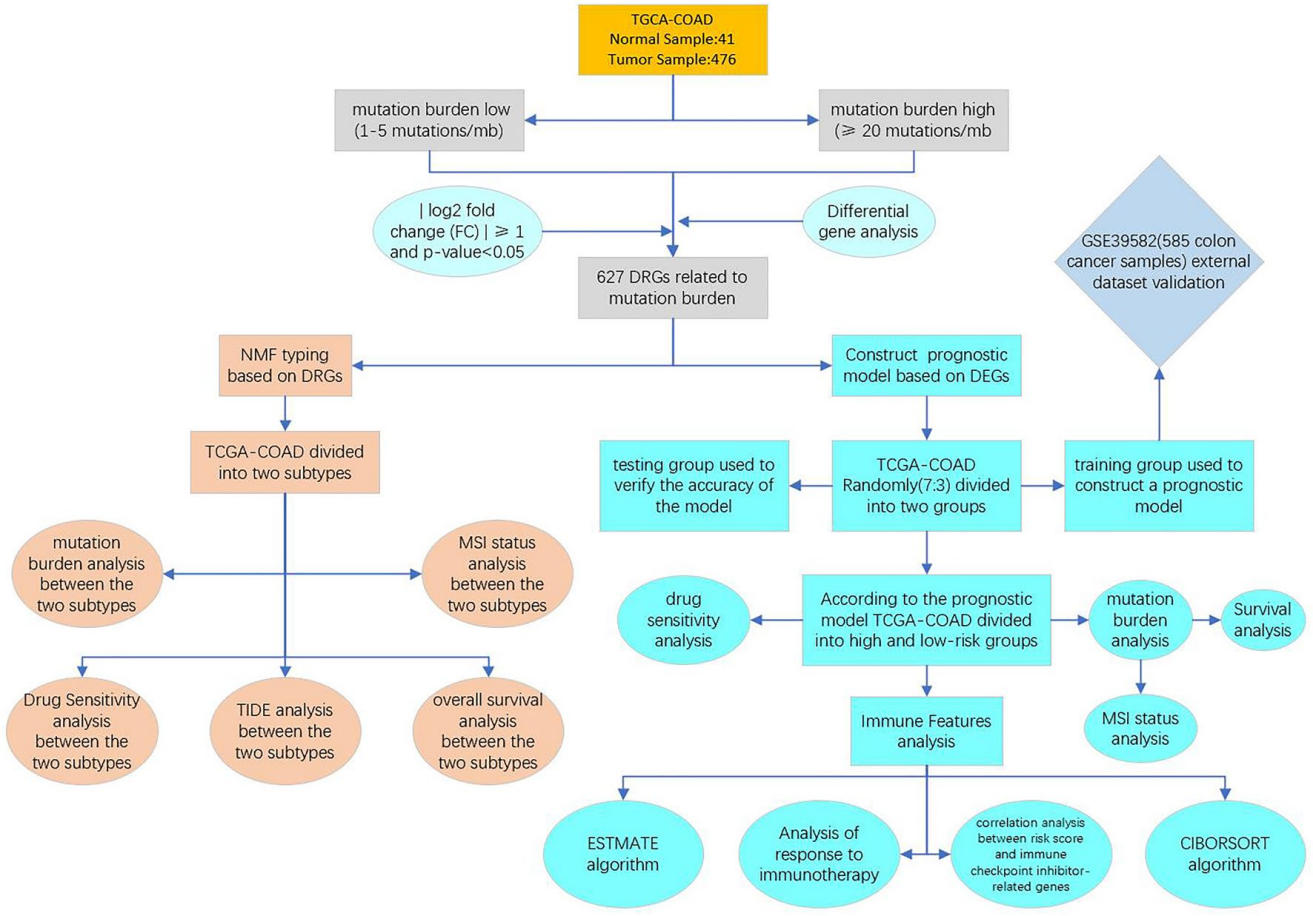
The workflow of the study.

The workflow of the whole study is presented in Fig. 1.

### Data processing and preprocessing

From The Cancer Genome Atlas (TCGA) database (https://portal.gdc.cancer.gov/) We downloaded transcriptome data (TSV format), clinical information data (XML format), and single nucleotide variation (SNV) data (MAF format) from colon adenocarcinoma (COAD) samples (including 476 tumor tissues and 41 normal tissue samples), and processed the data using Perl script to obtain the required gene expression matrix, clinical information file, and mutation data file. From the Gene Expression Omnibus (GEO) website (http://www.ncbi.nlm.nih.gov/geo/) Download the raw microarray data of the colon cancer dataset (GSE39582), use Perl script to organize and convert the raw data into a gene matrix, and obtain the necessary clinical information based on the raw data as an independent validation set for subsequent prognostic models.

### Obtaining DEGs related to mutation burden

Based on the mutation burden data of the TCGA-COAD queue, the queue samples were divided into a high mutation burden group, medium mutation burden group, and low mutation burden group. Differential gene analysis was performed on the high and low mutation burden groups to obtain differentially expressed genes DEGs related to mutation burden.

### Identification of subtypes of colon adenocarcinoma using Non-Negative Matrix Factorization (NMF) based on DEGs

Single-factor Cox analysis was performed using mutation burden-related DEGs to obtain feature genes. Molecular typing of TCGA-COAD was performed using the "NMF" package^20^ in R language combined with feature genes. The k value of the typing number is set between 2 and 10. Referring to the parting parameter graph, determine the optimal K value as 2.

### Analysis of tumor microenvironment, immune cell infiltration, MSI, and mutation burden for two subtypes

Use ESTIMATE algorithm^21^ to score the tumor microenvironment and analyze the differences in microenvironment among different subtypes of tumors. Use the "MCPcounter" package in R language to perform immune cell analysis on two subtypes. Subsequently, the difference in mutation burden and MSI between different subtypes was analyzed (MSI data from https://tcia.at/home).

### Survival analysis, drug sensitivity analysis, and immunotherapy sensitivity analysis of two subtypes

Evaluate whether there are differences in overall survival (OS) between different subtypes using the R language "survivor" and "survival" packages. From the GDSC website (https://www.cancerrxgene.org/) Obtain the database files (GDSC2 Expr. rds and GDSC2 Res. rds)^22^, and use the "oncoPredict" software package^23^ to evaluate the drugs sensitivity of different subtypes. Finally, use TIDE scoring (http://tide.dfci.harvard.edu/) to predict the effectiveness of immunotherapy in different subtypes.

### Constructing a prognostic model using DEGs

Firstly, univariate Cox and survival analysis were performed on DEGs to obtain prognostic-related genes, with a correlation threshold set at p < 0.05. Then, the TCGA cohort was randomly divided into two groups: the training group and the testing group. Lasso-Cox regression analysis was used to select prognostic-related genes and construct a risk prediction model. Use the GEO database colon cancer dataset (GSE39582) as the validation queue for external dataset validation of the constructed model.

The risk score is calculated using the following formula: $Risk score={\sum }_{k=1}^{n}[\mathrm{Exp }({\text{Gene}}) *\mathrm{ coef }({\text{Gene}})]$, while Exp (Gene) is the prognostic-related expression level and coef (Gene) is the relevant regression coefficient. Use R software packages such as "limma", "survival", "care", "glmnet", "surveyor", "timeROC", etc. to construct a DEGs prognosis model, and create Receiver Operating Characteristic Curve (ROC) for each group based on the obtained model. Analyze the OS of the TCGA group, training group, and testing group. Finally, perform independent prognostic analysis, the establishment of the nomogram, and clinical grouping model validation on the TCGA group.

### Functional enrichment analysis of high and low-risk groups

The ClusterProfiler software package^24^ is used for high and low-risk gene ontology (GO) and Kyoto Encyclopedia of Genes and Genomes (KEGG) enrichment analysis^25–27^. GSEA algorithm^28^ is an abundance method for calculating the measurement proportion of specific paths or features in different clusters, using the gene set (c5.go.symbols.gmt) (downloaded from the MSigDB database: https://www.gsea-msigdb.org/gsea/index.jsp) perform GSEA analysis, with p < 0.05 and FDR < 0.05.

### Analysis of immune microenvironment and immune infiltration in high and low-risk groups

Based on the constructed prognostic model, the TCGA-COAD queue was divided into high-risk and low-risk groups. Use ESTIMATE algorithm^21^ to evaluate the tumor microenvironmental characteristics of high and low-risk groups. Use the CIBERSORT algorithm^29^ to analyze the infiltration of 22 types of immune cells in both high and low-risk groups. Use R language software packages such as "GSVA" and "GSEABase" to analyze the immune function of high and low-risk groups. Use the "MCPcounter" package in R language to analyze the correlation between risk score and immune cell infiltration. Finally, analyze the correlation between risk scores and immune checkpoint inhibitor-related genes.

### High and low-risk groups mutation burden analysis and MSI analysis

Use the "Maftools" package in R language to evaluate the mutation characteristics of high and low-risk groups and analyze the relationship between tumor mutation burden and clinical prognosis. Subsequently, MSI analysis was conducted on two groups of patients with high and low risk, to observe whether there were differences in microsatellite instability between the two groups.

### Analysis of drug sensitivity and immunotherapy sensitivity in high and low-risk groups

Obtain database files (GDSC2nExpr. rds and GDSC2Res. rds)^22^, from the GDSC website and use the "oncoPredict" software package^23^ to evaluate the drug sensitivity of high and low risk groups. TIDE (http://tide.dfci.harvard.edu/) Upload TCGA-COAD gene expression data on the website to obtain TIDE scores to evaluate the response of high and low-risk groups to immunotherapy. Obtain immunotherapy scoring files for the TCGA-COAD queue on the TCIA website (https://tcia.at/home) to evaluate the response of high and low-risk groups to different immunotherapies.

### Verifying the accuracy of the prognostic model using the colon cancer dataset (GSE39582)

According to the obtained prognostic model formula, the samples in the GSE39582 queue were divided into two groups: high and low risk. The OS analysis was performed on the high and low-risk groups, and the ROC curve was drawn to evaluate the accuracy of the model prediction. The TIDE score of the GSE39582 queue was obtained by uploading expression data on the TIDE website, and the immune treatment effects of the high and low-risk groups were analyzed.

### Statistical method

Statistical analysis was conducted using R language, software version 4.3.1, with P < 0.05 as the difference with statistical significance. At the same time, we defined (P < 0.05 as *; P < 0.01 as **; P < 0.001 as ***). Select the criteria of | log2 fold change (FC) |≥ 1 and p-value < 0.05 to identify DEGs. When analyzing drug sensitivity, select a P-value < 0.05.

## Result

### Differential gene expression between high mutation burden and low mutation burden COAD

**Figure 2:**
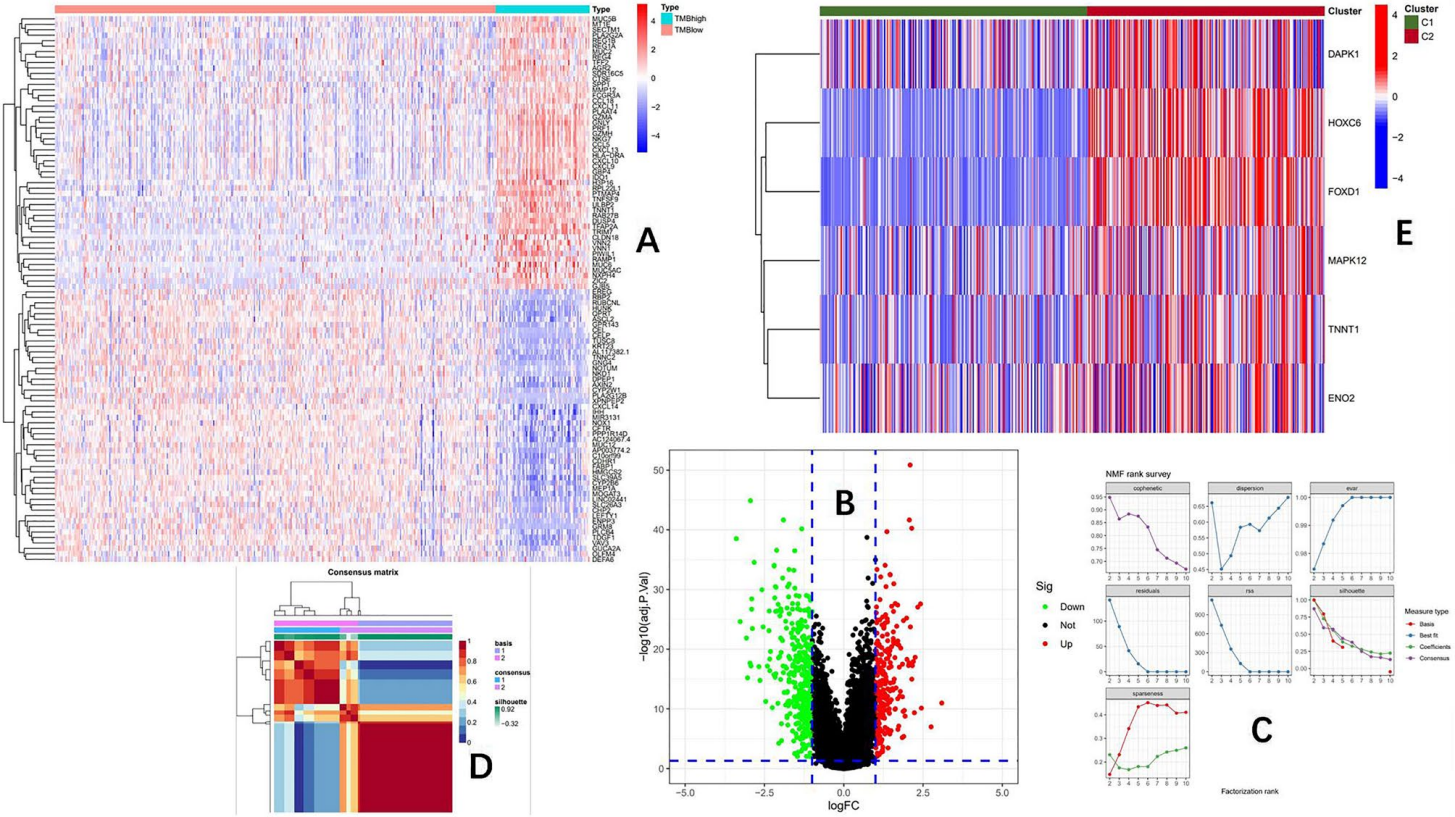
(**A**) Heat map of differentially expressed genes between high mutation burden and low mutation burden colon cancer (top 50 genes). (**B**) Volcano map of differentially expressed genes between high mutation burden and low mutation burden colon cancer. (**C**) The NMF classification parameter diagram shows that the slope of the curve is the highest when k = 2 to k = 3, so k = 2 is the best choice for clustering and grouping the queue. (**D**) The classification chart shows that the red color inside the classification has high correlation, while the blue color outside the classification has low correlation. (**E**) From the heat map, it can be seen that MAPK12, TNNT1, HOXC6, ENO2, FOXD1, and DAPK1 are significantly overexpressed in C2.

Based on the mutation status of the TCGA-COAD queue, we divided the queue samples into low mutation burden group (1–5 mutations/Mb), medium mutation burden group (6–19 mutations/Mb), and high mutation burden group (≥ 20 mutations/Mb) according to reference^19^. Differential gene analysis was conducted between the high mutation burden group and the low mutation burden group, with conditions | log2 fold change (FC) |≥ 1 and p-value < 0.05, resulting in 627 differentially expressed genes DEGs (Fig. 2A,B).

### Identification of subtypes using non-negative matrix factorization (NMF) based on DEGs

To further identify the characteristic tumor mutation burden-related genes, we used the obtained DEGs to perform NMF typing on the TCGA-COAD queue. Based on Fig. 2C, we determined that the best choice for clustering grouping the queue was when k = 2. From the classification chart (Fig. 2D), it can be seen that the graph inside the classification is red with a high correlation, while the graph outside the classification is blue with a low correlation. Finally, six genes were identified as the most relevant genes for mutation burden (*MAPK12*, *TNNT1*, *HOXC6*, *ENO2*, *FOXD1*, *DAPK1*). From the gene heatmap (Fig. 2E), it can be seen that these six genes are significantly overexpressed in the C2 group.

### Significant difference in mutation burden and MSI status between the two subtypes

**Figure 3:**
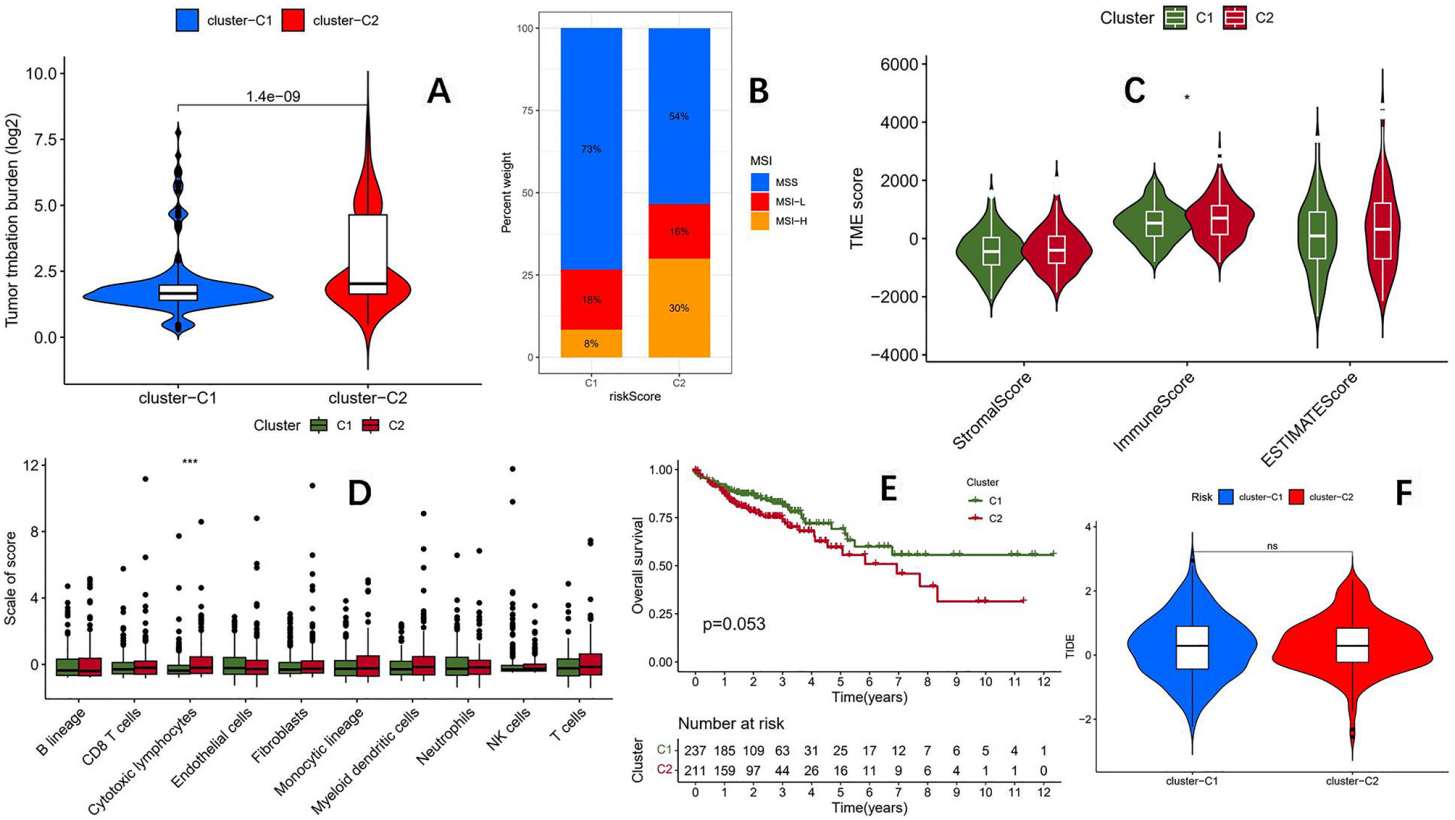
(**A**) There is an incredible difference in the mutation burden between C1 and C2 (p = 1.4e-09). (**B**) The proportion of MSI-H in the C2 group was significantly higher than that in the C1 group (30% vs.8%). (**C**) The immune score of the C2 group was significantly higher than that of the C1 group. (**D**) The infiltration of cytotoxic lymphocytes in group C2 was significantly higher than that in group C1. (**E**) The overall survival (OS) of the C1 group was better than that of the C2 group, but P = 0.053. (**F**) There was no significant difference in TIDE scores between the C1 group and the C2 group, indicating that the immunotherapy effects were equivalent between the two groups.

To evaluate whether the identified subtypes can effectively distinguish the tumor mutation burden status, we analyzed the tumor mutation burden between two subtypes, and the results showed (Fig. 3A) that there was an incredible difference in mutation burden between the two subtypes, with the C1 group having significantly lower mutation burden than the C2 group. It is suggested that using the aforementioned six genes can effectively distinguish the mutation burden status of tumors. Previous studies have shown a significant correlation between TMB and MSI status, and previous studies have shown that TMB can predict the response of MSI-H metastatic colorectal cancer to immune checkpoint inhibitors^30^. Therefore, we further analyzed the MSI status between the two subtypes, and the results showed (Fig. 3B) that the proportion of MSI-H in the C2 group was significantly higher than that in the C1 group (30% vs. 8%), indicating a significant correlation between high mutation burden and MSI-H, which is consistent with previous studies^14–16^. Further analysis of the immune microenvironment of the two subtypes (Fig. 3C) showed that the immune score of the C2 group was significantly higher than that of the C1 group. The final analysis of immune cell infiltration showed that the infiltration of cytotoxic lymphocytes in the C2 group was significantly higher than that in the C1 group (Fig. 3D), which is similar to previous studies^31^.

### Analysis of drug sensitivity, overall survival, and immunotherapy sensitivity of two subtypes

To evaluate whether there is a difference in survival between the two subtypes, we plotted the OS curves of the two groups of patients (Fig. 3E), and the results showed that the C1 group had a better prognosis than the C2 group, but the P-value was 0.053, indicating that patients with low mutation burden had a better prognosis in colon adenocarcinoma. Similar results have been obtained in studies of head and neck tumors^32^. Previous studies have shown that the prognosis of colon cancer patients is related to mutation burden. Among tumor patients with Microsatellite stable(MSS) and high TMB (> 8 mutations/Mb), the median OS is longer than that of tumor patients with MSS and low TMB (33.8 months vs. 28.1 months; P = 0.02)^33^. However, although the mutation burden in the C2 group is higher than that in the C1 group, the prognosis in the C2 group is poorer than that in the C1 group. We performed survival analysis only on MSS patients based on NMF subtype results, and the overall survival of the two subtypes was similar (Fig. 12A). The reason may be that the proportion of MSS patients in the C2 group is significantly lower than that in the C1 group, resulting in a poorer prognosis in the C2 group compared to the C1 group. With the emergence of high-throughput technologies, the application of gene expression profiling in predicting drug sensitivity has become increasingly widespread. Studies have combined gene expression profiles to reveal the role of traditional Chinese medicine ingredients in the development of human cancer^34,35^. We used the "oncoPredict" R package to analyze the drug sensitivity between two subtypes and found significant differences in drug sensitivity between them. Among the predicted 197 drugs, 113 drugs exhibit differences in sensitivity (Table 1). A detailed analysis result of drug sensitivity can be found in the supplementary file. These different drugs include anticancer chemotherapy drugs and targeted therapy drugs, and the overall drug sensitivity of the C2 group was significantly better than that of the C1 group. Among the 113 drugs with differences, 107 drugs in the C2 group had better sensitivity than that of the C1 group, which was the opposite, The sensitivity of only 6 drugs was better in the C1 group than in the C2 group. The KEYNOTE-158 study suggests that TMB may be a new biomarker for predicting the effectiveness of immunotherapy^36^. We used the TIDE score to predict the effectiveness of immunotherapy between two subtypes, and the results showed (Fig. 3F) that there was no significant difference in the TIDE score between the two subtypes. It is speculated that the TMB in colon cancer cannot be used as a biomarker for predicting immunotherapy.

**Table 1:** Drug sensitivity of C1 group and C2 group.

	C1	C2
AZD2014		Better
AZD4547		Better
AZD5153		Better
AZD5363		Better
AZD5438		Better
5-Fluorouracil		Better
Alisertib		Better
AMG-319		Better
AZ960		Better
AZD1332		Better
BDP-00009066		Better
BMS-345541		Better
BMS-536924		Better
BMS-754807		Better
Buparlisib		Better
AZD5582		Better
AZD6738		Better
AZD7762		Better
AZD8055		Better
AZD8186		Better
CDK9_5576		Better
Cisplatin		Better
Crizotinib		Better
Cytarabine		Better
Dabrafenib		Better
Dactinomycin		Better
Dactolisib		Better
Dinaciclib		Better
Camptothecin		Better
CDK9_5038		Better
ERK_2440		Better
ERK_6604		Better
Fludarabine		Better
Foretinib		Better
Docetaxel		Better
Eg5_9814		Better
Elephantin		Better
Entinostat		Better
Entospletinib		Better
Epirubicin		Better
IAP_5620		Better
I-BRD9		Better
IGF1R_3801		Better
IRAK4_4710		Better
Irinotecan		Better
JAK_8517		Better
Gemcitabine		Better
GNE-317		Better
GSK269962A		Better
GSK2606414		Better
LJI308		Better
Luminespib		Better
MIM1		Better
Mirin		Better
JAK1_8709		Better
JQ1		Better
KU-55933		Better
LCL161		Better
Leflunomide		Better
KRAS (G12C) inhibitor-12		Better
Olaparib		Better
OTX015		Better
PAK_5339		Better
Palbociclib		Better
Mitoxantrone		Better
MK-8776		Better
Niraparib		Better
NU7441		Better
Nutlin-3a (−)		Better
Obatoclax mesylate		Better
PLX-4720		Better
PRIMA-1MET		Better
PRT062607		Better
Pyridostatin		Better
Ribociclib		Better
PCI-34051		Better
PD0325901		Better
Pevonedistat		Better
Pictilisib		Better
Podophyllotoxin bromide		Better
Savolitinib		Better
SCH772984		Better
Sorafenib		Better
Staurosporine		Better
Talazoparib		Better
RO-3306		Better
Ruxolitinib		Better
RVX-208		Better
Sabutoclax		Better
Telomerase inhibitor IX		Better
Ulixertinib		Better
ULK1_4989		Better
Uprosertib		Better
VE821		Better
VE-822		Better
Vinblastine		Better
Vincristine		Better
Teniposide		Better
Topotecan		Better
Trametinib		Better
Wnt-C59		Better
WZ4003		Better
XAV939		Better
YK-4-279		Better
Vinorelbine		Better
VX-11e		Better
Wee1 inhibitor		Better
Lapatinib	Better	
Sapitinib	Better	
TAF1_5496	Better	
WEHI-539	Better	
Acetalax	Better	
Dihydrorotenone	Better	

### Constructing and validating a prognostic model based on DEGs

**Figure 4:**
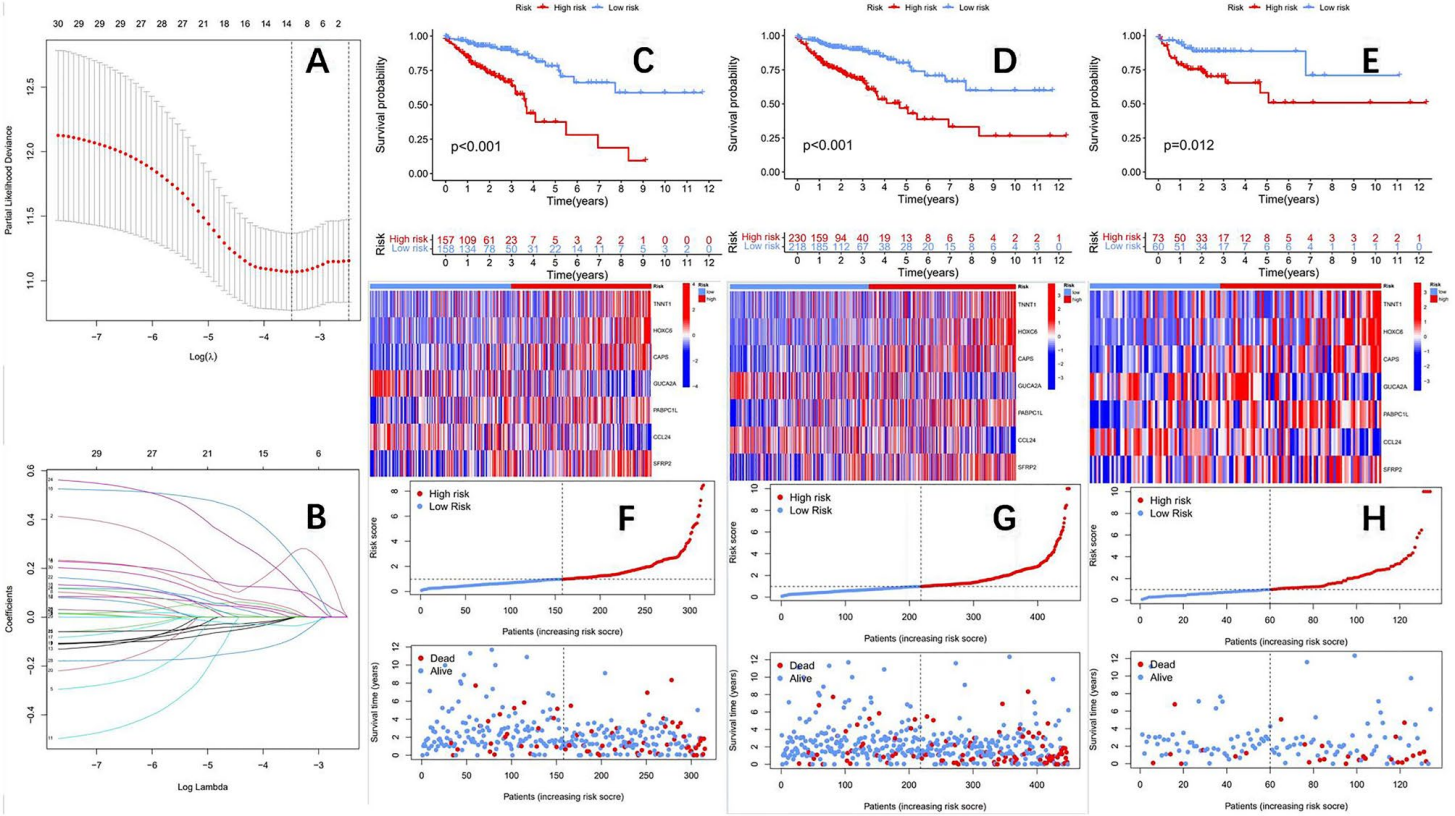
(**A**) The tuning parameter(λ) in the LASSO model. (**B**) LASSO coefficient distribution of DEGs. (**C**) Survival curve of the training group (P < 0.001). (**D**) Survival curve of the TCGA group (P < 0.001). (**E**) Survival curve of the test group (P = 0.012). (**F**–**H**) Training group, TCGA group, testing group risk heat map, risk score distribution map, and survival status distribution map.

Using DEGs, the most relevant prognostic genes were screened through univariate and Cox regression. The TCGA cohort was randomly divided into two groups (clinical characteristics of the two groups of patients are shown in Table 2), one group being the training group and the other group being the testing group. The training group was used to construct a prognostic model, and the testing group was used to verify the accuracy of the model. Lasso regression analysis was used to construct a prognostic model for DEGs. Use cross-validation to achieve optimal results (λ) Value to further identify genes related to prognosis (Fig. 4A,B). Finally, a prognostic model was determined for 7 DEGs, including *TNNT1, HOXC6, CAPS, GUCA2A, PABPC1L, CCL24*, and *SFRP2*. And obtain the model formula: The risk score is calculated using the following formula: *TNNT1* (Exp)* (0.183779401950069) + *HOXC6* (Exp) * (0.201142479025189) + *CAPS* (Exp) * (0.498479272258504) + *GUCA2A* (Exp) * (− 0.10640063476006) + *PABPC1L* (Exp) * (0.389482649796005) + *CCL24* (Exp) * (− 0.214745574612184) + *SFRP2* (Exp) * (0.0892478268418448).

**Table 2:** Clinical characteristics of the training group and testing group.

Clinical trait	Type	Total	Test	Train	P value
Age	<=65	184 (40.98%)	51 (38.06%)	133 (42.22%)	0.4741
Age	> 65	265 (59.02%)	83 (61.94%)	182 (57.78%)	
Gender	Female	214 (47.66%)	59 (44.03%)	155 (49.21%)	0.3672
Gender	Male	235 (52.34%)	75 (55.97%)	160 (50.79%)	
Stage	Stage I	75 (16.7%)	22 (16.42%)	53 (16.83%)	0.1822
Stage	Stage II	177 (39.42%)	48 (35.82%)	129 (40.95%)	
Stage	Stage III	124 (27.62%)	47 (35.07%)	77 (24.44%)	
Stage	Stage IV	62 (13.81%)	16 (11.94%)	46 (14.6%)	
Stage	Unknownn	11 (2.45%)	1 (0.75%)	10 (3.17%)	
T	T1	11 (2.45%)	3 (2.24%)	8 (2.54%)	0.7823
T	T2	76 (16.93%)	20 (14.93%)	56 (17.78%)	
T	T3	306 (68.15%)	96 (71.64%)	210 (66.67%)	
T	T4	56 (12.47%)	15 (11.19%)	41 (13.02%)	
M	M0	331 (73.72%)	105 (78.36%)	226 (71.75%)	0.4377
M	M1	62 (13.81%)	16 (11.94%)	46 (14.6%)	
M	Unknown	56 (12.47%)	13 (9.7%)	43 (13.65%)	
N	N0	267 (59.47%)	75 (55.97%)	192 (60.95%)	0.3735
N	N1	102 (22.72%)	30 (22.39%)	72 (22.86%)	
N	N2	80 (17.82%)	29 (21.64%)	51 (16.19%)

**Figure 5:**
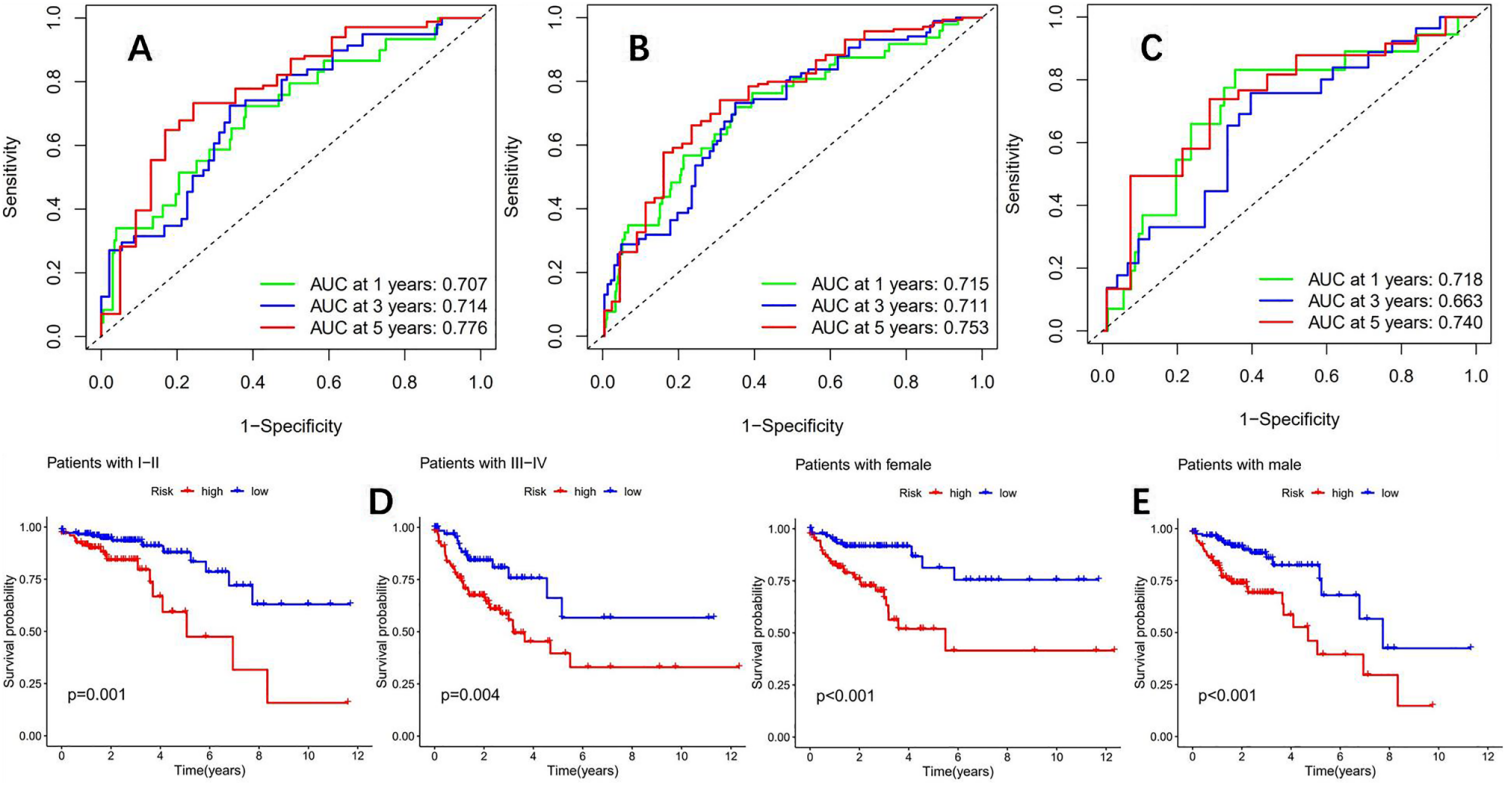
(**A**) The ROC curves of the training group (AUC values of 0.707, 0.714, and 0.776 for 1, 3, and 5 years, respectively). (**B**) The ROC curves of the TCGA group (1 year, 3 year, and 5 year AUC values were 0.715, 0.711, and 0.752, respectively). (**C**) The ROC curves of the test group (AUC values of 0.718, 0.663, and 0.740 for 1 year, 3 years, and 5 years, respectively). Clinical subgroup validation model predictive ability: (**D**) (disease stage), (**E**) (gender).

**Figure 6:**
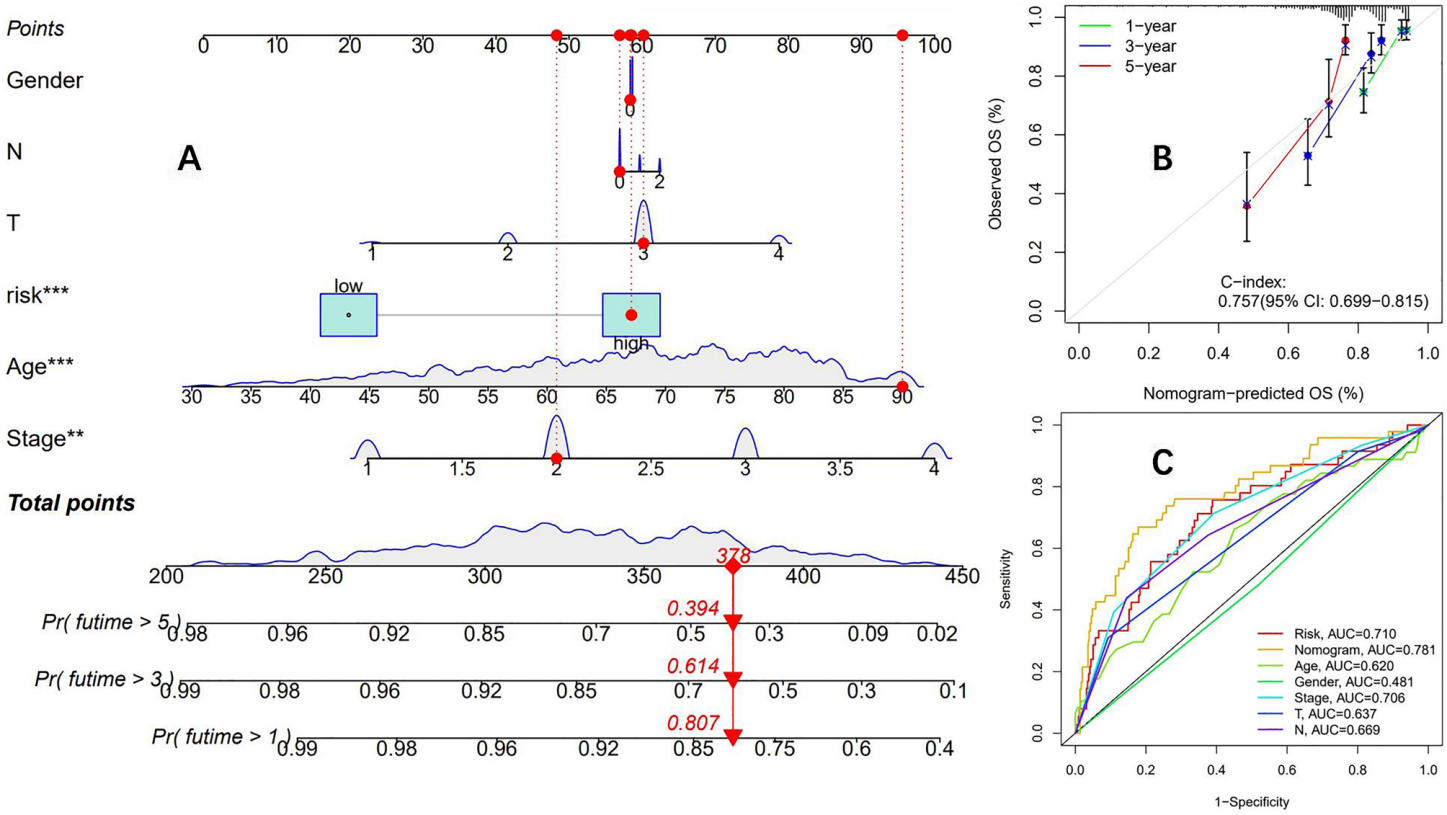
(**A**) A nomogram of a clinical prediction model based on prognostic model risk score combined with clinical features. (**B**) Calibration curve of nomogram. (**C**) Comparison of predictive power of nomograms, risk scores, and clinical characteristics.

According to the risk formula, we divided patients into two groups: high and low risk. From the survival curve, it can be seen that in the training group, TCGA group, and testing group, the OS of the high-risk group is significantly lower than that of the low-risk group, with a P value < 0.05, and the difference is statistically significant (Fig. 4C–E). At the same time, R language software was used to draw risk heatmaps, risk score distribution maps, and survival status distribution maps for three groups of patients (Fig. 4F–H). Subsequently, we plotted ROC curves in the training group (Fig. 5A), TCGA group (Fig. 5B), and testing group (Fig. 5C), with AUC of 0.707, 0.714, and 0.776 (training group), 0.715, 0.711, and 0.752 (TCGA group), 0.718, 0.663, and 0.740 (testing group) for 1, 3, and 5 years, respectively. The comprehensive analysis of the survival curve and ROC curve indicates the reliability of this prognostic model in predicting the prognosis of colon adenocarcinoma patients. Finally, clinical subgroup model validation showed that the prognostic model was a good predictor of patient survival in different clinical trait groups: tumor stage (Fig. 5D) and sex (Fig. 5E). To further enhance the predictive ability of the model's prognosis, we established a nomogram (Fig. 6A) in conjunction with clinical characteristics. The calibration curve showed that the predicted results of the nomogram were consistent with actual observations at 1, 3, and 5 years of OS, with a C-index of 0.765 (95% CI: 0.696 − 0.834) (Fig. 6B). The combined ROC curve (Fig. 6C) shows that the AUC value of the predicted risk score obtained by the model is comparable to that of tumor staging, indicating that the model's predictive ability for prognosis is comparable to that of traditional tumor staging. At the same time, the accuracy of the constructed nomogram prediction is further improved, and its predictive ability is significantly better than that of tumor staging.

### Functional enrichment analysis of high and low-risk groups

**Figure 7:**
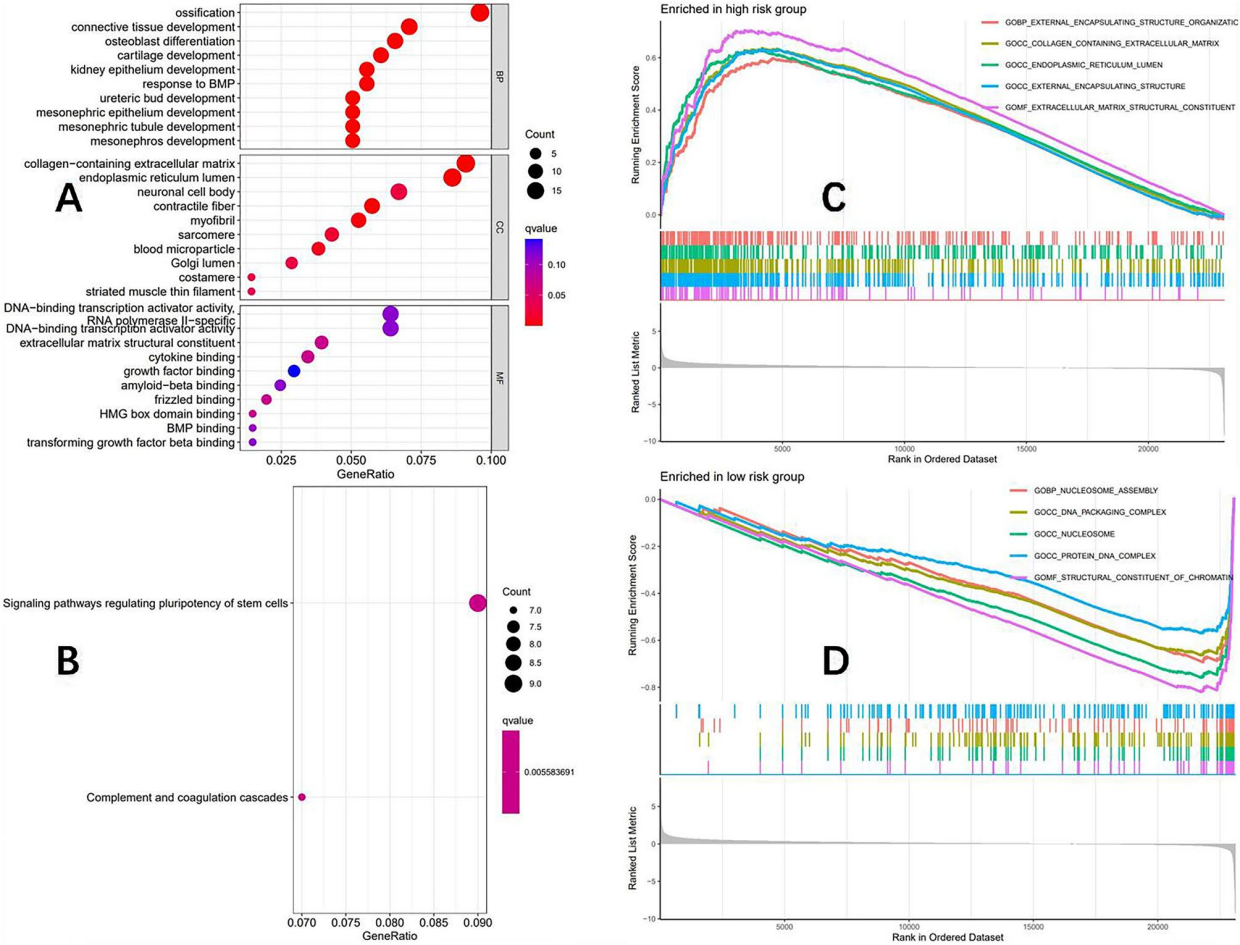
According to the prognosis model, the TCGA-COAD queue was divided into two groups: high and low risk. (**A**) GO analysis bubble chart. (**B**) KEGG analysis bubble chart. (**C**) High-risk group GSEA analysis results. (**D**) Low-risk group GSEA analysis results.

According to the prognostic model, the TCGA-COAD queue was divided into two groups: high and low risk. GO analysis of the two groups (Fig. 7A) showed that the differences in molecular function mainly focused on the DNA binding transcription activator activity pathway, while KEGG analysis (Fig. 7B) showed that there were differences in the Signaling pathways regulating pluripotency of stem cells between the high and low-risk groups. GSEA analysis showed that in the high-risk group (Fig. 7C), enrichment was mainly found in pathways such as external encapsulating structure organization, collagen-containing extracellular matrix, endoplasmic reticulum lumen, external encapsulating structure, and extracellular matrix structural constituent, while in the low-risk group (Fig. 7D), enrichment was mainly found in pathways such as nucleosome assembly, DNA packaging complexes, nucleosome, protein DNA complex and structural constituent of chromatin.

### The high and low-risk groups have different immune microenvironments and immune infiltration characteristics

**Figure 8:**
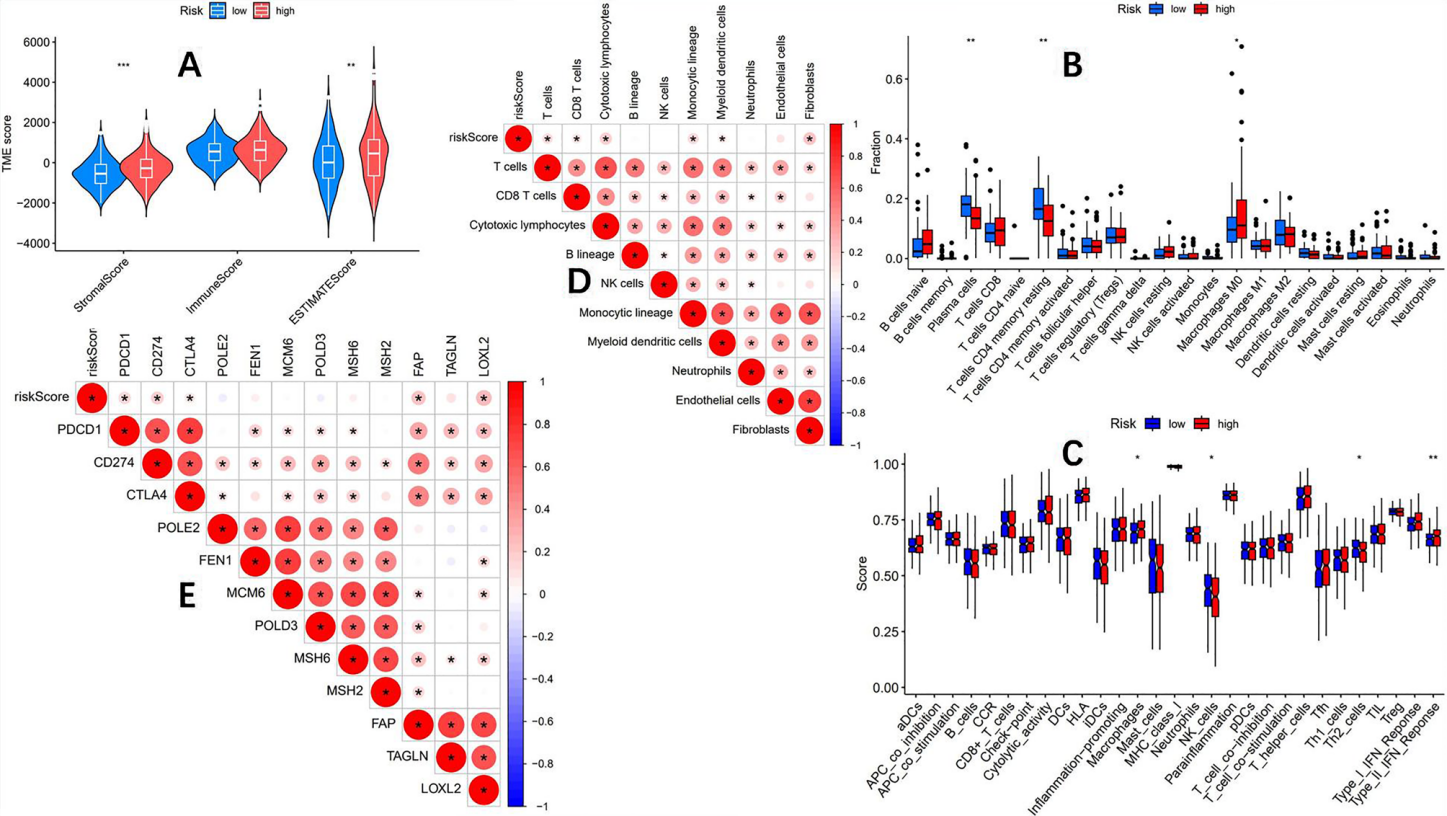
According to the prognosis model, the TCGA-COAD queue was divided into two groups: high and low risk. (**A**) Significant difference in stromal cell score and comprehensive score between the high and low-risk groups. (**B**) The CIBERSORT algorithm evaluates the infiltration of 22 types of immune cells in both high and low-risk groups, and there is a significant difference in the infiltration abundance of plasma cells, memory resting CD4+ T cells, and M0 macrophages. (**C**) The analysis of immune function between high and low-risk groups showed significant differences in NK cells, Th2 cells, macrophages, and type II interferon response function between the two groups. (**D**) The correlation analysis between risk score and immune cell infiltration showed that the risk score was significantly correlated with T cells, CD8+ T cells, cytotoxic lymphocytes, monocytes, myeloid dendritic cells, and fibroblasts. (**E**) The correlation analysis between risk score and immune checkpoint inhibitor related genes showed that the risk score was significantly correlated with genes PDCD1, CD274, and CTLA4. (P < 0.05 is *; P < 0.01 is **; P < 0.001 is ***).

Research has shown that immune cells in the tumor microenvironment play a crucial role in the occurrence and development of tumors^37^. The composition of the tumor microenvironment is closely related to the clinical efficacy of immune checkpoint inhibitors (ICIs)^38^. We used ESTIMATE algorithm^21^ to score the tumor microenvironment in the TCGA-COAD queue and analyze the differences in immune microenvironment between the high and low-risk groups. The results showed significant differences in stromal cell scores and comprehensive scores between the high and low-risk groups (Fig. 8A). Use the CIBERSORT algorithm^29^ to analyze the infiltration of 22 types of immune cells in both high and low-risk groups. The results showed (Fig. 8B) that the infiltration of plasma cells and memory resting CD4+ T cells was significantly higher in the low-risk group than in the high-risk group, while the infiltration of M0 macrophages was significantly higher in the high-risk group than in the low-risk group. Further immune function analysis of the high and low-risk groups (Fig. 8C) showed that the low-risk group was significantly active in NK cell and Th2 cell functions, while the high-risk group was significantly active in macrophage and type II interferon response functions. The correlation analysis between risk score and immune cell infiltration using the "MCPcounter" package in R language (Fig. 8D) showed that the risk score obtained from the prognostic model was significantly correlated with T cells, CD8+ T cells, cytotoxic lymphocytes, monocytes, myeloid dendritic cells, and fibroblasts. Finally, a correlation analysis was conducted between risk score and ICI-related genes (Fig. 8E), and the risk score was significantly correlated with genes *PDCD1*, *CD274*, *CTLA4*, *FAP*, and *LOXL2* indicating a close relationship between the risk score obtained by this prognostic model and immunotherapy.

### The high and low-risk groups have different MSI status, but the TMB status is the same

**Figure 9:**
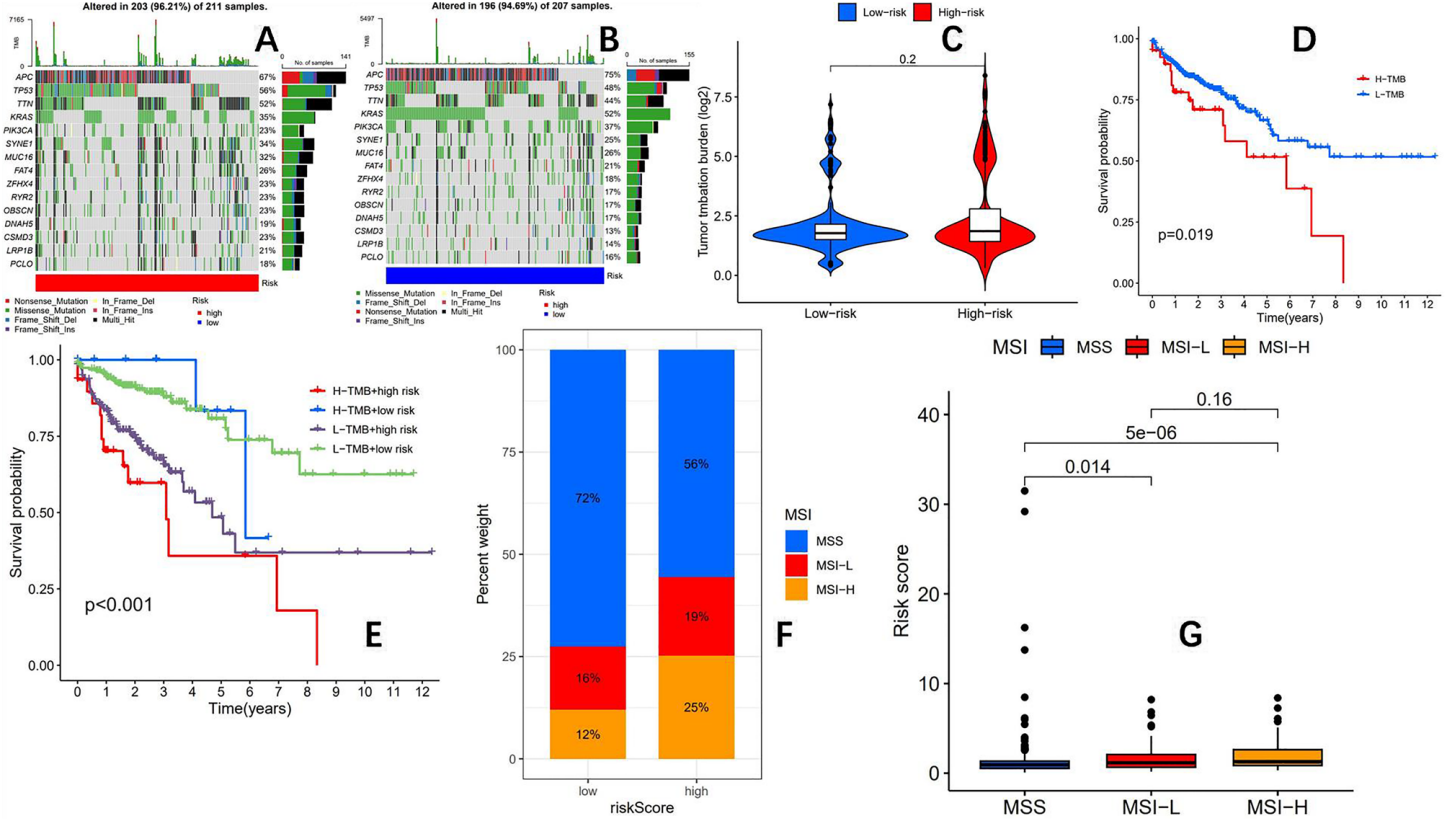
According to the prognosis model, the TCGA-COAD queue was divided into two groups: high and low risk. (**A**) Waterfall plot for high-risk groups. (**B**) Waterfall plot for low-risk groups. (**C**) There was no statistically significant difference in mutation burden between the high and low-risk groups. (**D**) The OS of the low mutation burden group was better than that of the high mutation burden group (p = 0.019). (**E**) Risk score and mutation burden combined survival analysis OS curve. (**F**,**G**) The proportion of MSI-H in the high-risk group was significantly higher than that in the low-risk group (25% vs12%).

Based on the prognostic model, we conducted a mutation burden analysis on the high and low-risk groups of the TCGA-COAD queue. From the waterfall plot (Fig. 9A,B), we found that the top 15 highly mutated genes in the high and low-risk groups were *APC, TP53, TTN, KRAS, PIK3CA, SYNE1, MUC16, FAT4, ZFHX4, RYR2, OBSCN, DNAH5, CSMD3, LRP1B, PCLO*, but the mutation proportion of each highly mutated gene was not the same between the two groups, Further analysis of the mutation burden between the high and low-risk groups showed no significant difference in mutation burden between the two groups (Fig. 9C). Survival analysis showed that the prognosis of patients with low mutation burden was significantly better than that of patients with high mutation burden (Fig. 9D), with p = 0.019, and the difference was statistically significant. Subsequently, we conducted a joint survival analysis using risk scores and mutation burden, and the results showed (Fig. 9E) that overall, the prognosis of patients in the low-risk group was better than that in the high-risk group, which once again suggests the accuracy of the prognosis model. Finally, we conducted MSI analysis on the high and low-risk groups, and the results showed that the proportion of MSI-H in the high-risk group was significantly higher than that in the low-risk group (Fig. 9F,G).

### The response to drugs and immunotherapy is significantly different between the high and low-risk groups

**Figure 10:**
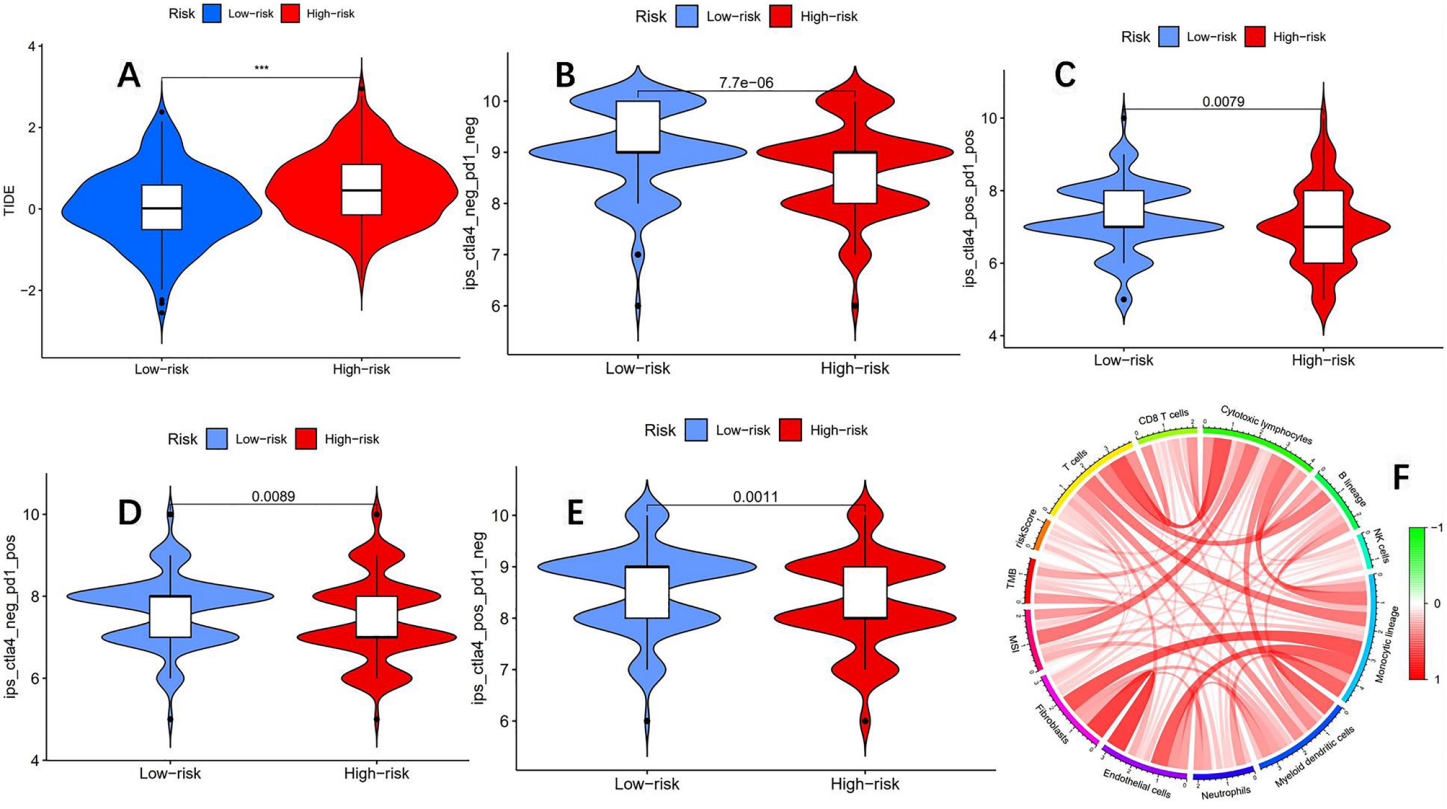
According to the prognosis model, the TCGA-COAD queue was divided into two groups: high and low risk. (**A**) The TIDE score of the low-risk group was significantly lower than that of the high-risk group, indicating that the immunotherapy effect of the low-risk group was significantly better than that of the high-risk group (P < 0.001 is ***). (**B–E**) The low-risk group showed significantly better results than the high-risk group in both individual and combined immunotherapy. (**F**) A comprehensive analysis of the correlation between risk scores, TMB, MSI, and immune cells showed that these four factors were basically positively regulated.

Using the "oncoPredict" software package^23^ to evaluate the sensitivity of high and low-risk groups to drugs, the results showed that among the 197 evaluated anti-tumor drugs, there were differences in the sensitivity of 62 drugs between the high and low-risk groups (Table 3). A detailed analysis result of drug sensitivity can be found in the supplementary file. Careful analysis of drugs with sensitivity differences revealed that in the low-risk group, the sensitivity of the commonly used chemotherapy drug oxaliplatin for colon cancer was higher than that of the high-risk group. Afterward, we get the TIDE score from the TIDE website (http://tide.dfci.harvard.edu/), and use the TIDE score to evaluate the response of the high and low-risk groups to immunotherapy. The results showed (Fig. 10A) that the TIDE score of the low-risk group was significantly lower than that of the high-risk group, indicating that the response of the low-risk group to immunotherapy was significantly better than that of the high-risk group. Finally, on the TCIA website (https://tcia.at/home) obtain the scoring file of the TCGA-COAD queue and evaluate the response of high and low-risk groups to different immunotherapies. The results showed that the low-risk group had significantly better effects than the high-risk group in both individual and combined immunotherapy (Fig. 10B–E). The prognostic model constructed in this study can effectively predict the efficacy of immunotherapy in colon cancer. Finally, we comprehensively analyzed the correlation between risk score, TMB, MSI, and immune cells in the TCGA-COAD cohort (Fig. 10F), and the results showed that these four were positive regulatory relationships.

**Table 3:** Drug sensitivity of low-risk group and high-risk group.

	Low-risk group	High-risk group
AZD3759	Better	
AZD5438	Better	
AZD5991	Better	
AZD6482	Better	
Bortezomib	Better	
Cyclophosphamide	Better	
Afatinib	Better	
Afuresertib	Better	
AGI-5198	Better	
AT13148	Better	
EPZ5676	Better	
Erlotinib	Better	
GDC0810	Better	
Gefitinib	Better	
GSK343	Better	
GSK591	Better	
GSK2578215A	Better	
Dabrafenib	Better	
Dihydrorotenone	Better	
Entinostat	Better	
Ibrutinib	Better	
KU-55933	Better	
MIRA-1	Better	
OF-1	Better	
Osimertinib	Better	
Oxaliplatin	Better	
Picolinici-acid	Better	
Ribociclib	Better	
Sapitinib	Better	
IAP_5620	Better	
TAF1_5496	Better	
Temozolomide	Better	
Trametinib	Better	
Venetoclax	Better	
Savolitinib	Better	
SB216763	Better	
Sinularin	Better	
ERK_2440		Better
GSK2606414		Better
IGF1R_3801		Better
Alpelisib		Better
AZ960		Better
AZD1332		Better
AZD8186		Better
BMS-754807		Better
Dasatinib		Better
Entospletinib		Better
PLX-4720		Better
PRIMA-1MET		Better
RVX-208		Better
JQ1		Better
Linsitinib		Better
Luminespib		Better
OSI-027		Better
PAK_5339		Better
Pictilisib		Better
Sepantronium bromide		Better
Taselisib		Better
WIKI4		Better
WZ4003		Better
XAV939		Better
Telomerase inhibitor IX		Better

### GSE39582 dataset validation of prognostic model accuracy

**Figure 11:**
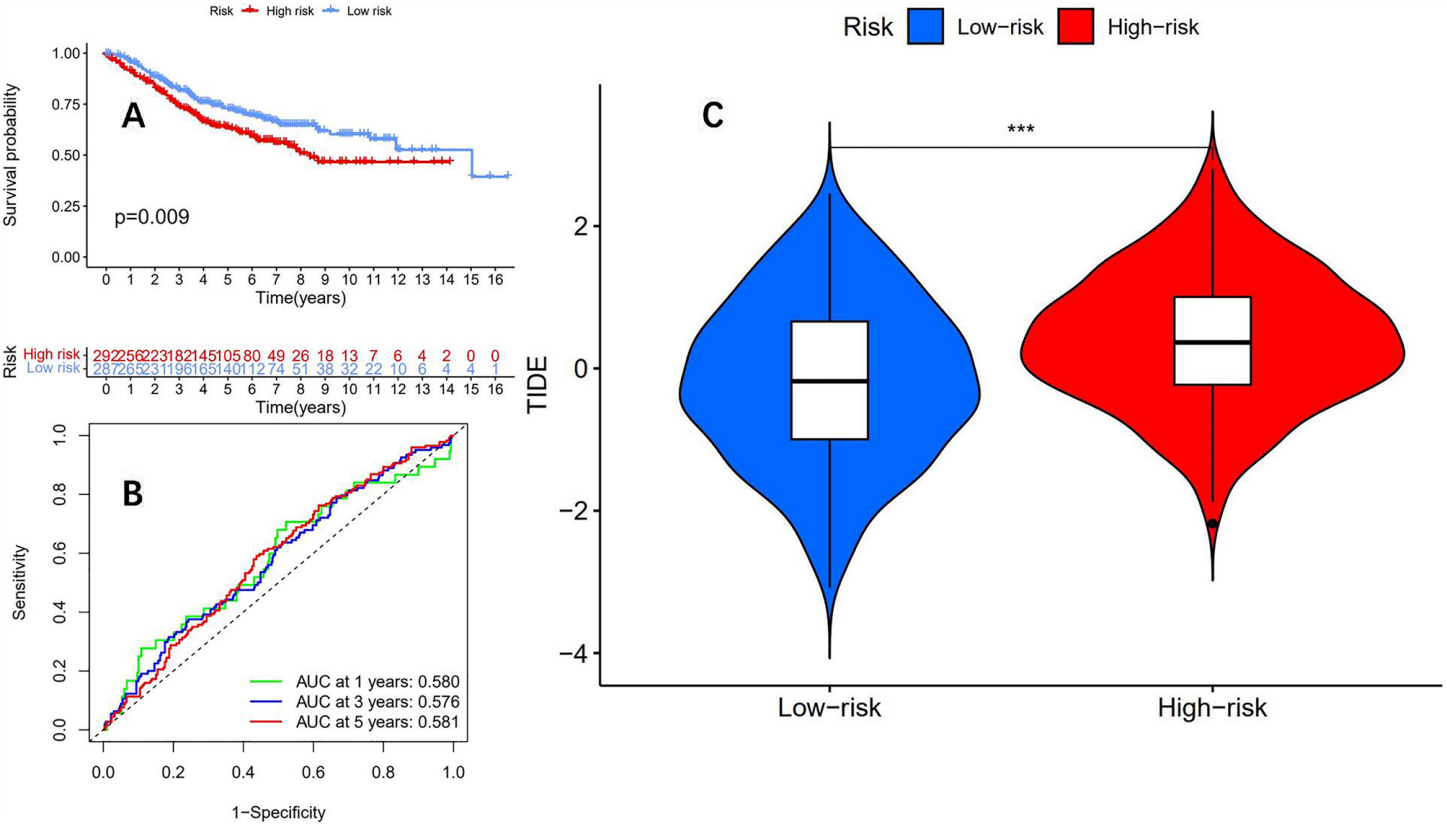
The GSE39582 dataset was used to verify the accuracy of the prognostic model. (**A**) The OS of the low-risk group was significantly better than that of the high-risk group (p = 0.009). (**B**) The ROC curve shows that the AUC values of the prognostic model in the GSE39582 dataset for 1 year, 3 years, and 5 years are 0.580, 0.576, and 0.581, respectively. (**C**) In the GSE39582 dataset, the prognostic model can still predict the effectiveness of immunotherapy well (* * * means P < 0.001).

To evaluate the accuracy of the prognostic model, we used the GSE39582 dataset from the GEO database to validate the accuracy of the model. The overall survival curve (Fig. 11A) showed that the prognosis of the low-risk group was significantly better than that of the high-risk group, with p = 0.009, and the difference was statistically significant. The ROC curve (Fig. 11B) showed that in the GSE39582 cohort, the 1-year, 3-year, and 5-year AUCs were 0.580, 0.576, and 0.581, respectively. Further use of the TIDE score to predict the immunotherapy efficacy of the high and low-risk groups in the GSE39582 cohort showed that the immunotherapy efficacy of the low-risk group was significantly better than that of the high-risk group (Fig. 11C), indicating the reliability of the risk model in predicting the efficacy of immunotherapy.

### The newly constructed prognostic model surpasses the traditional biomarkers MSI and TMB

**Figure 12:**
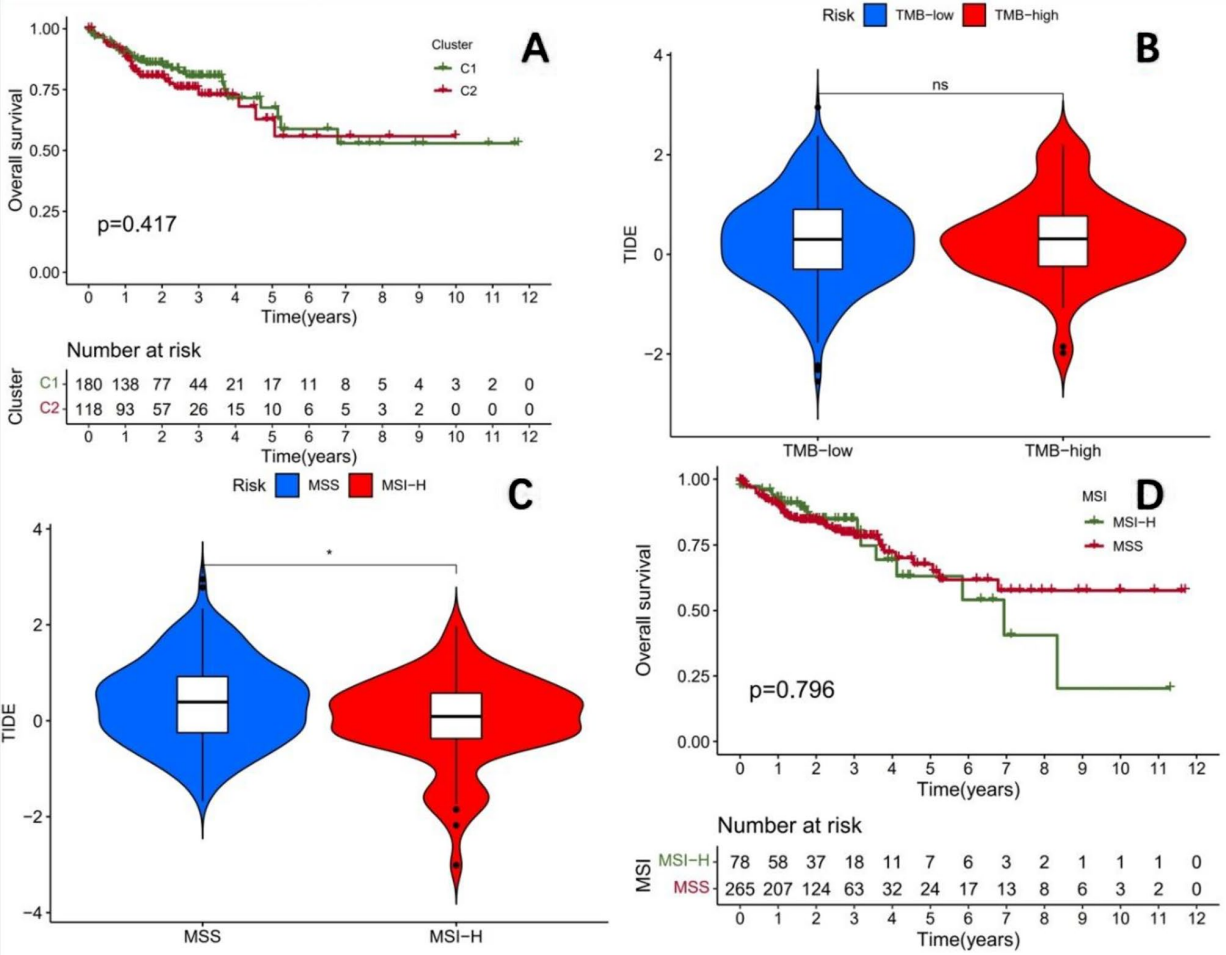
(**A**) According to the results of NMF classification, survival analysis of only MSS patients shows that the overall survival between the two subtypes is similar. (**B**) There is no significant difference in TIDE scores between the two groups of patients with high and low mutation burden in the TCGA-COAD cohort. (**C**) The TIDE score of the MSI-H group was significantly lower than that of the MSS group, indicating that the immunotherapy effect of the MSI-H group was significantly better than that of the MSS group. (P < 0.05 is *). (**D**) There is no significant difference in survival between MSI-H and MSS patients in the TCGA-COAD cohort.

To further compare the differences between the prognostic model constructed in this study and the traditional colon cancer prognostic markers MSI and TMB, we grouped the TCGA-COAD cohort based on the TMB status and found no significant difference in TIDE scores between the two groups with high and low mutation burdens (Fig. 12B). We further grouped the TCGA-COAD cohort based on the MSI status and found that MSI-H patients had significantly lower TIDE scores compared to MSS (P < 0.05), indicating a statistically significant difference (Fig. 12C). However, the predictive efficiency was not as good as the prognostic model constructed in this study. There was no significant difference in survival between the MSI-H and MSS groups (Fig. 12D), suggesting that MSI alone cannot predict the prognosis of colon cancer patients. Taking into account the above analysis results, TMB can predict the prognosis of colon cancer patients (Fig. 9D), but cannot predict the efficacy of immunotherapy. On the other hand, MSI can predict the efficacy of immunotherapy for colon cancer, but cannot predict the clinical prognosis of patients. The prognostic model constructed in this study not only effectively differentiates patient prognosis but also predicts the efficacy of immunotherapy. Therefore, the newly constructed prognostic model is superior to the traditional biomarkers MSI and TMB.

## Discussion

Since the first approval of ipilimumab for the treatment of metastatic melanoma in 2011, oncology treatment has entered the era of immunotherapy. Immune checkpoint inhibitors (ICIs) have demonstrated durable anti-tumor effects in the treatment of many types of cancers. For example, non-small cell lung cancer^39^, urothelial cancer^40^, triple-negative breast cancer^41^, renal cell cancer^42^, etc. Predictive biomarkers are needed for ICI treatment to screen potential beneficiaries. Currently, most ICI treatments use programmed cell death-Ligand 1(PD-L1) to predict treatment efficacy, but PD-L1 is not a perfect biomarker. Although there is a correlation between PD-L1 expression and immunotherapy response rate in pan-cancer analysis, many PD-L1-expressing patients are resistant to ICI, and some patients without PD-L1 expression benefit from treatment^43^. PD-L1 expression is not an ideal biomarker for screening potential beneficiaries of ICI treatment. Therefore, it is urgent to explore new markers for predicting the efficacy of immunotherapy. Based on Keynote-177^12^ and CheckMate-142^13^ studies in colorectal cancer, MSI-H/dMMR is a reliable biomarker for predicting the effectiveness of immunotherapy in colorectal cancer. There are also studies indicating that high mutation burden tumors have high microsatellite instability^14–16^, suggesting that TMB may be a candidate biomarker for predicting immunotherapy efficacy in colon adenocarcinoma patients. Therefore, this study screened DEGs related to mutation burden and performed NMF typing on the TCGA-COAD queue. Based on the expression levels of *MAPK12, TNNT1, HOXC6, ENO2, FOXD1*, and *DAPK1*, the TCGA-COAD queue was successfully divided into two subtypes. There was a significant difference in the mutation burden and the ratio of MSI-H between the two subtypes, and the proportion of MSI-H was significantly higher in the high mutation burden subtype than in the low mutation burden subtype, this indicates that high mutation burden means high microsatellite instability, which is consistent with previous studies^14–16^. Previous studies have shown that patients with MSI-H in colon cancer may not be sensitive to the chemotherapy drug 5-FU^6,44^. In this study, although the proportion of MSI-H in the high mutation burden subtype was significantly higher than that in the low mutation burden subtype, the sensitivity of the high mutation burden subtype to 5-FU was significantly higher than that of the low mutation burden subtype. This indicates that although there is a close relationship between mutation burden and MSI status, there is still a significant difference between the two biomarkers. Previous studies have shown that tumors with high mutation levels will have higher levels of tumor neoantigens and exhibit higher immunogenicity, resulting in a better response to immunotherapy^45^. Studies have shown that patients with high tumor mutation burden respond better to immunotherapy^17,18^. However, there are also studies indicating that a high mutation burden does not predict immunotherapy response well^46,47^. This study conducted immunotherapy analysis on the two subtypes of high and low mutation burden and found that there was no significant difference in immunotherapy between the high mutation burden subtype and the low mutation burden subtype. Therefore, this study suggests that TMB is not a suitable biomarker for predicting the effectiveness of immunotherapy in colon adenocarcinoma.

To further explore molecular markers or prognostic models for predicting immunotherapy, we successfully constructed a prognostic model using mutation burden-related DEGs. This prognostic model can effectively predict the prognosis of colon adenocarcinoma patients. The AUC values at 1 year, 3 years, and 5 years in the training group were 0.707, 0.714, and 0.776, respectively. However, similar AUC values were found in the test group and TCGA group, indicating that the prognostic model has good stability. Further external validation using the GEO database large sample colon cancer dataset (GSE39582) showed that the model can still predict patient prognosis well, indicating its reliability. In the past, many studies have utilized genes to construct prognostic models for colon cancer. For example, a study has constructed a prognostic model for colon cancer based on metabolism-related lncRNA, with AUC values of 0.768 and 0.735 at 3 and 5 years, respectively^48^, which are comparable to this study. However, the model obtained from this study has not been validated by external datasets. There are also studies using Pyroptosis-related genes to construct a prognosis model for colon cancer, and the results show that the AUC values at 1 year, 3 years, and 5 years are 0.659, 0.630, and 0.627, respectively^49^, which were significantly worse than the prognostic model constructed in this study. At the same time, to further improve the predictive ability of the prognostic model, we combined clinical characteristics to construct a nomogram. The comprehensive AUC value of the constructed nomogram was 0.781, while the AUC value of the risk model was 0.710.

Subsequently, to further explore the feasibility of constructing a prognostic model to predict the effectiveness of immunotherapy, we analyzed the immune microenvironment and immune infiltration characteristics of the high and low-risk groups and found significant differences in stromal cell scores and ESTIMATE comprehensive scores between the high and low-risk groups. Research has shown that the tumor microenvironment is closely related to immune therapy response^50^. It suggests that the high and low-risk groups may have different reactions to immunotherapy. At the same time, we analyzed the infiltration of immune cells in both high and low-risk groups, and the results showed that plasma cells and memory resting CD4+ T cells infiltration were significantly higher in the low-risk group than in the high-risk group, while M0 macrophage infiltration was significantly higher in the high-risk group than in the low-risk group. Studies have shown that CD4 T cells can significantly improve the effectiveness of immunotherapy^51^, and studies have also shown that tumor-associated macrophages (TAMs) promote cancer progression by promoting tumor invasion and immunosuppression^52^. Therefore, speculate the response of the low-risk group to immunotherapy may be better than that of the high-risk group. According to the correlation analysis between the risk score obtained from the prognosis model and the genes related to ICIs, it was found that the risk score was significantly correlated with *PDCD1, CD274,* and *CTLA4*. It is well known that these three genes are closely related to Immune checkpoint inhibitor therapy, so there is a strong correlation between the prognosis model and immune therapy.

Finally, we used the TIDE score to predict the response of the high and low-risk groups to immunotherapy. The results showed that the TIDE score of the low-risk group was significantly lower than that of the high-risk group, indicating that the immunotherapy of the low-risk group was significantly better than that of the high-risk group. Subsequently, we downloaded the immunotherapy scoring file for the TCGA-COAD queue on the TCIA website and analyzed the responses of the high and low-risk groups between different immunotherapy regimens. The results showed that whether using Programmed Death receptor-1(PD1) inhibitors or CTLA4 inhibitors alone for immunotherapy, or PD1 inhibitors combined with CTLA4 inhibitors for treatment, the treatment effect of the low-risk group was significantly better than that of the high-risk group, Finally, we validated the reliability of the risk model in predicting immunotherapy in the GSE39582 cohort, and the results showed that the immunotherapy efficacy of the low-risk group was still significantly better than that of the high-risk group. To further explore the mechanism by which risk scores predict the effectiveness of immunotherapy. We analyzed the mutation burden and MSI status of the high and low-risk groups, and the results showed that there was no significant difference in mutation burden between the high and low-risk groups, but the proportion of MSI-H in the high-risk group was significantly higher than that in the low-risk group. Currently, many studies have used MSI-H as a potential beneficiary population for immunotherapy in colon cancer patients^12,13^. Although the proportion of MSI-H in the high-risk group is significantly higher than that in the low-risk group, but the immunotherapy efficacy of the low-risk group is significantly better than that of the high-risk group. This indicates that the prognostic model constructed by our study is an independent biomarker for predicting the effectiveness of immunotherapy. At the same time, there was no significant difference in mutation burden between the high and low-risk groups, but the response to immunotherapy was significantly different, which once again proves that TMB is not a reasonable biomarker for predicting immunotherapy efficacy in colon cancer.

In summary, through systematic bioinformatics analysis, we have demonstrated that TMB is not a feasible biomarker for predicting immune therapy response in colon adenocarcinoma. At the same time, we successfully constructed a prognosis model containing 7 genes using mutation burden-related DEGs. This model can effectively predict the prognosis of colon adenocarcinoma patients and predict the effectiveness of immunotherapy. Of course, our research also has limitations. Firstly, this study is a bioinformatics study based on a public database. Although the results were validated with large sample external datasets, the response of patients to immunotherapy was predicted by the TIDE score, which does not necessarily predict the response of patients to immunotherapy. Secondly, the prognostic model obtained in this study lacks real-world clinical research data. Finally, the underlying mechanism by which the prognostic models obtained from the study predict the effectiveness of immunotherapy has not been fully elucidated, and further basic research is still needed.

In conclusion, this study obtained DEGs by analyzing the high mutation burden and low mutation burden samples in the TCGA-COAD queue. By using DEGs and NMF typing, the TCGA-COAD queue was successfully divided into a high mutation burden subtype and a low mutation burden subtype. Although there was a significant difference in the proportion of MSI-H between the two subtypes, but there was no difference in the efficacy of immunotherapy between the two subtypes. It indicated that TMB is not feasible to predict the response of colon cancer immunotherapy. Drug sensitivity analysis showed that the drug sensitivity of the high mutation burden subtype was significantly better than that of the low mutation burden subtype. To further explore the predictive biomarkers for the efficacy of immunotherapy, we successfully constructed a prognostic model using DEGs. The prognostic model can well distinguish the prognosis and immunotherapy effect of patients with high and low risk, and consistent results were obtained in the GSE39582 dataset validation. We also evaluated the TMB and MSI of the high and low-risk groups, and there was no significant difference in TMB between the high and low-risk groups. However, the proportion of MSI-H in the high-risk group was significantly higher than that in the low-risk group. This once again confirms that TMB cannot predict the immunotherapy effect of colon cancer. It also suggests that this risk model is an independent molecular marker for immunotherapy, which is significantly superior to the traditional colon cancer biomarkers TMB and MSI. Further clinical validation is needed.

### Supplementary Information

Supplementary Information.

## Data Availability

The data that support the findings of this study are openly available in the TCGA database (https://portal.gdc.cancer.gov/repository) and GEO database (https://www.ncbi.nlm.nih.gov/geo/query/acc.cgi?acc=GSE39582).
