# Supplementary figures and images for: Construction and validation of a colon cancer prognostic model based on tumor mutation burden-related genes (part 2 of 2)
Source: Sci Rep. 2024 Feb 4;14:2867. doi: 10.1038/s41598-024-53257-z (PMC10838917; doi:10.1038/s41598-024-53257-z)

Risk C1 C2

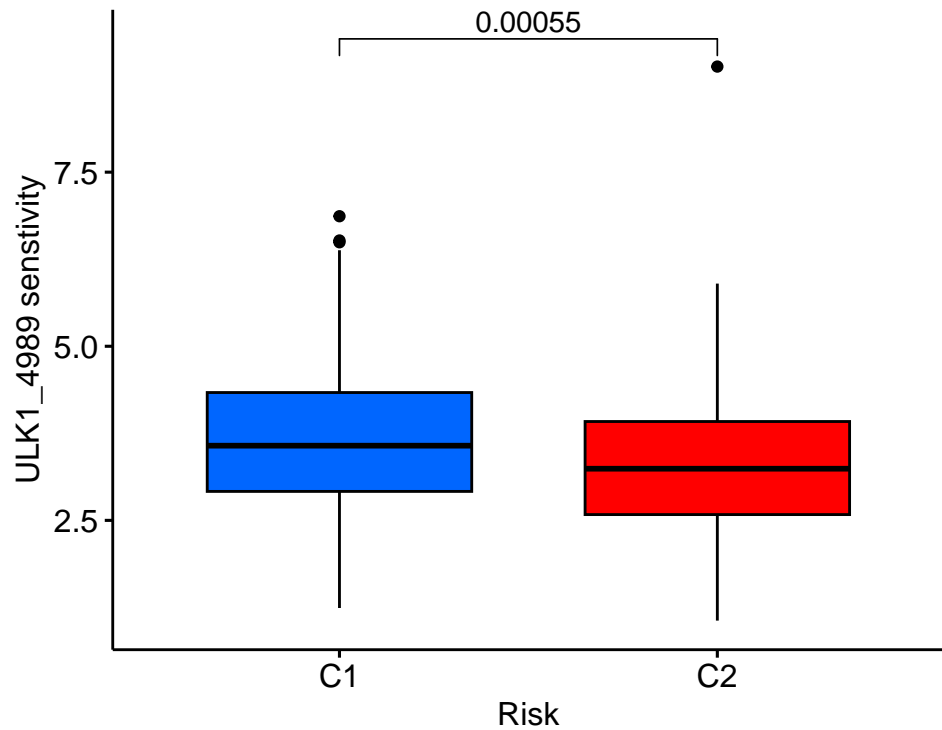

Supplement: Supplementary file 1 — Supplementary Information. [file 41598_2024_53257_MOESM1_ESM.zip › supplementary files/Drug sensitivity of C1 group and C2 group/C2 better/drugSenstivity.ULK1_4989.pdf]

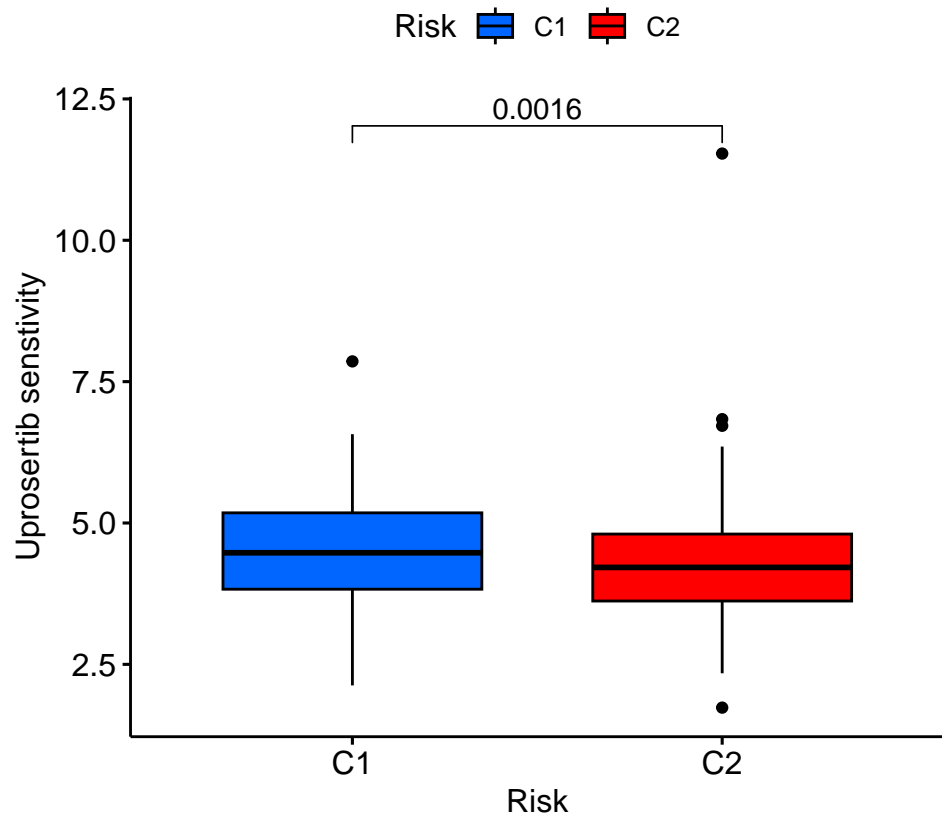

Supplement: Supplementary file 1 — Supplementary Information. [file 41598_2024_53257_MOESM1_ESM.zip › supplementary files/Drug sensitivity of C1 group and C2 group/C2 better/drugSenstivity.Uprosertib.pdf]

Risk C1 C2

0.0077

VE821 sensitivity

10.0

7.5

5.0

2.5

C1

C2

Risk

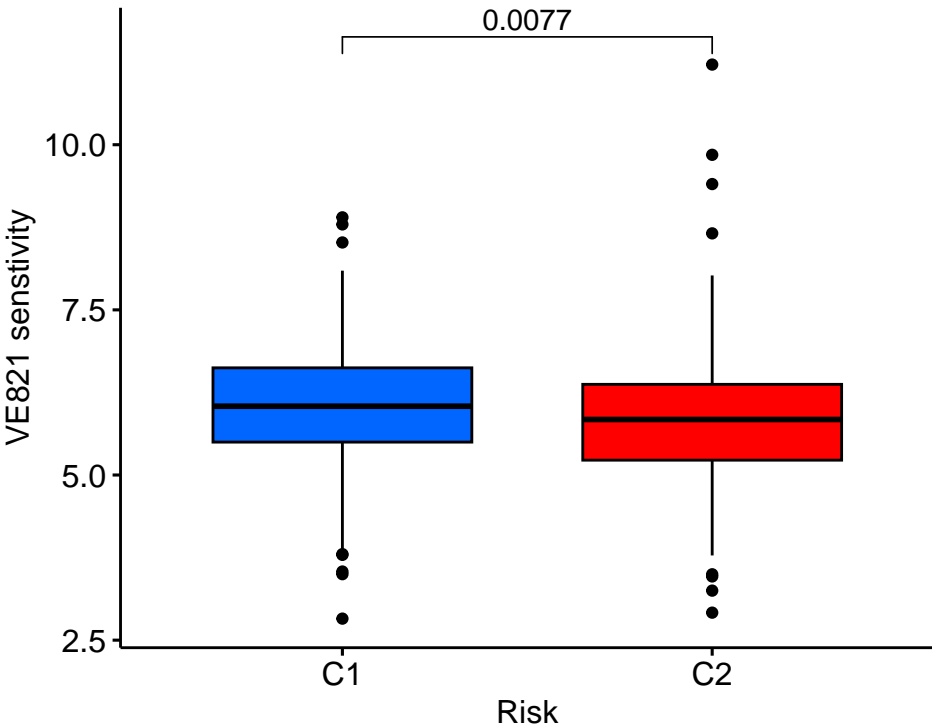

Supplement: Supplementary file 1 — Supplementary Information. [file 41598_2024_53257_MOESM1_ESM.zip › supplementary files/Drug sensitivity of C1 group and C2 group/C2 better/drugSenstivity.VE821.pdf]

Risk C1 C2

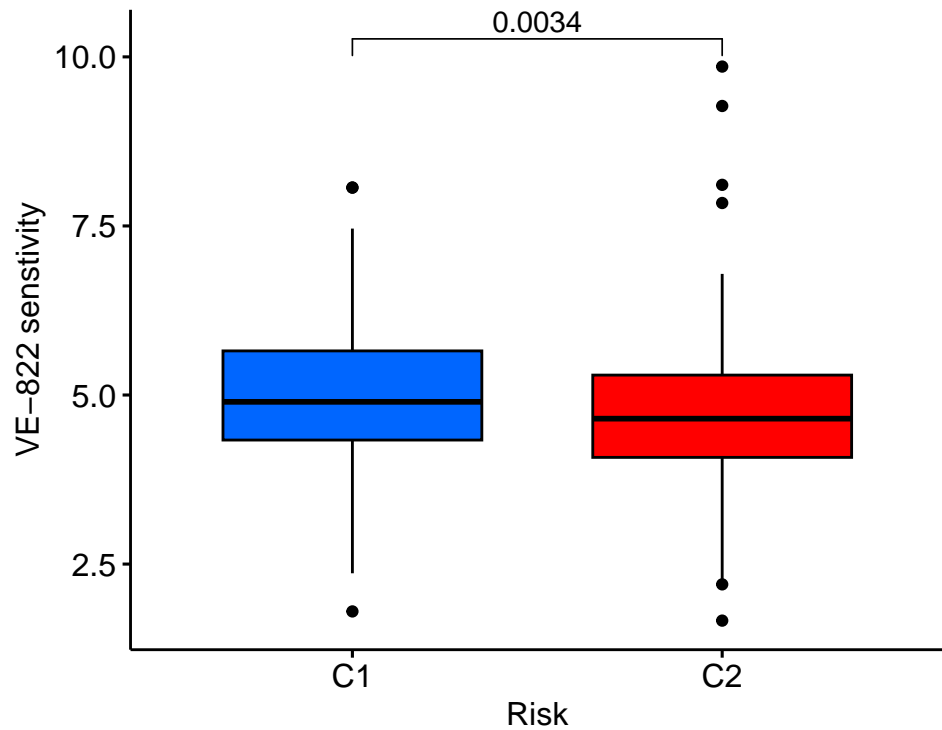

Supplement: Supplementary file 1 — Supplementary Information. [file 41598_2024_53257_MOESM1_ESM.zip › supplementary files/Drug sensitivity of C1 group and C2 group/C2 better/drugSenstivity.VE-822.pdf]

Risk C1 C2

5.6e-06

Vinblastine sensitivity

2.0

1.5

1.0

0.5

0.0

C1

C2

Risk

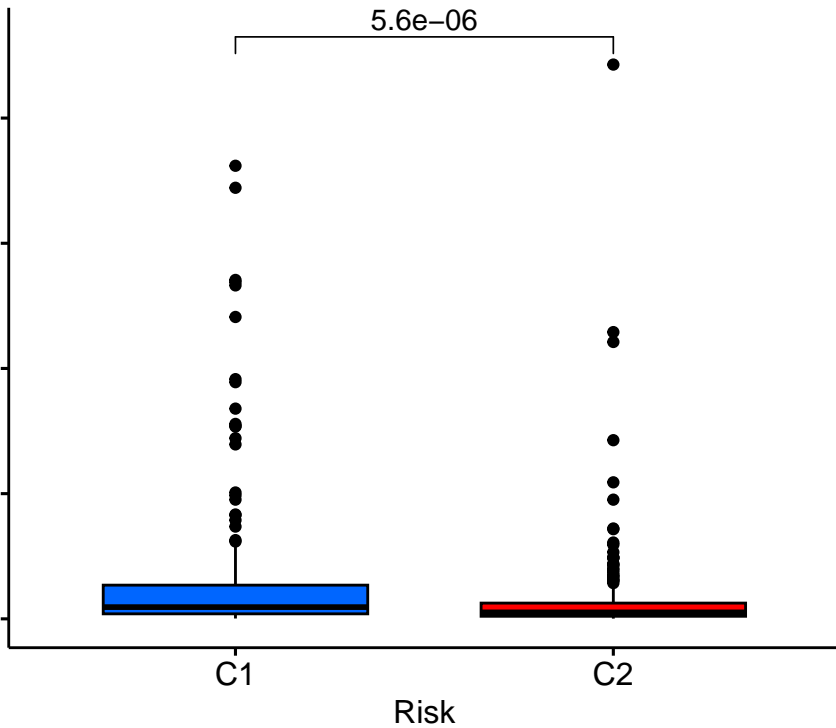

Supplement: Supplementary file 1 — Supplementary Information. [file 41598_2024_53257_MOESM1_ESM.zip › supplementary files/Drug sensitivity of C1 group and C2 group/C2 better/drugSenstivity.Vinblastine.pdf]

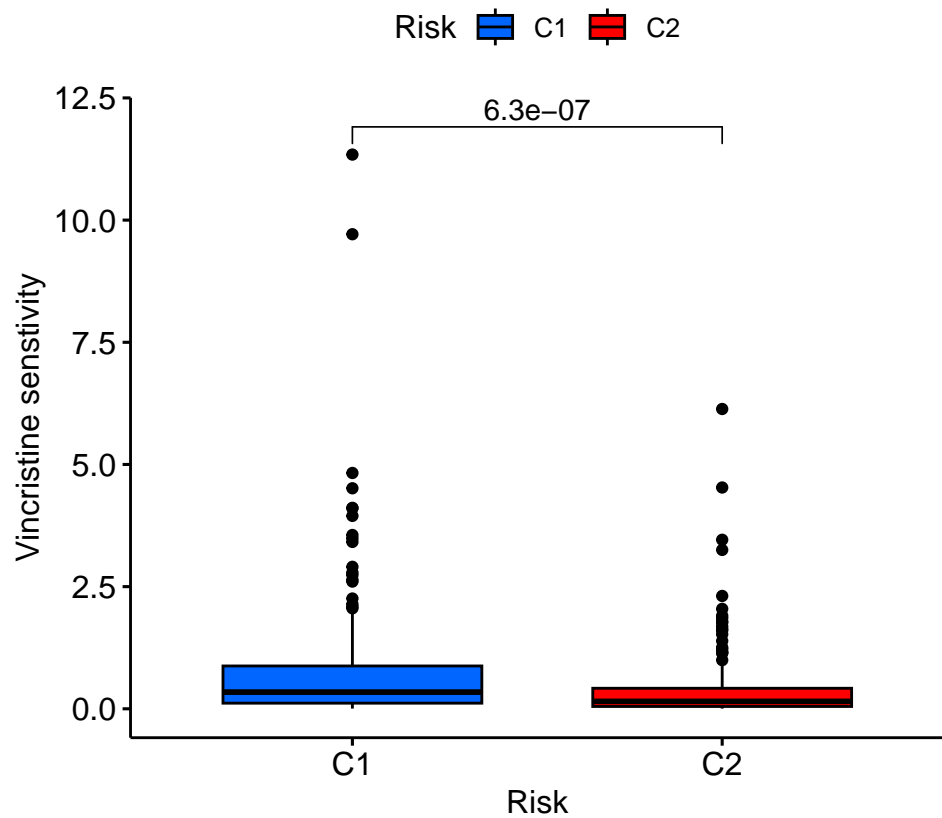

Supplement: Supplementary file 1 — Supplementary Information. [file 41598_2024_53257_MOESM1_ESM.zip › supplementary files/Drug sensitivity of C1 group and C2 group/C2 better/drugSenstivity.Vincristine.pdf]

Risk C1 C2

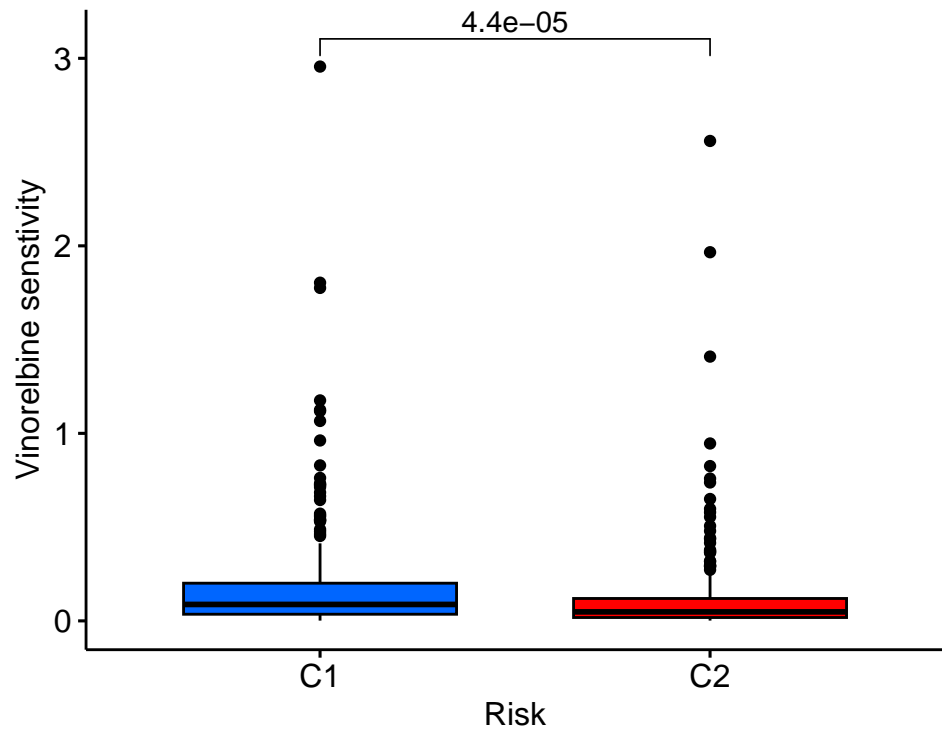

Supplement: Supplementary file 1 — Supplementary Information. [file 41598_2024_53257_MOESM1_ESM.zip › supplementary files/Drug sensitivity of C1 group and C2 group/C2 better/drugSenstivity.Vinorelbine.pdf]

Risk C1 C2

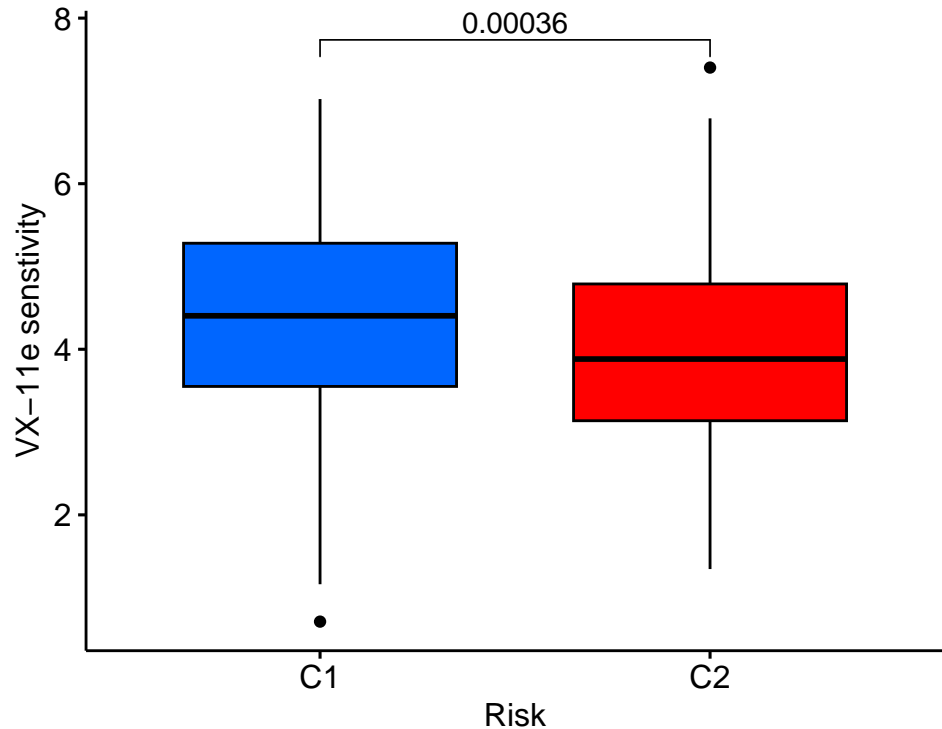

Supplement: Supplementary file 1 — Supplementary Information. [file 41598_2024_53257_MOESM1_ESM.zip › supplementary files/Drug sensitivity of C1 group and C2 group/C2 better/drugSenstivity.VX-11e.pdf]

Wee1 Inhibitor sensitivity

Risk C1 C2

0.011

C1

C2

Risk

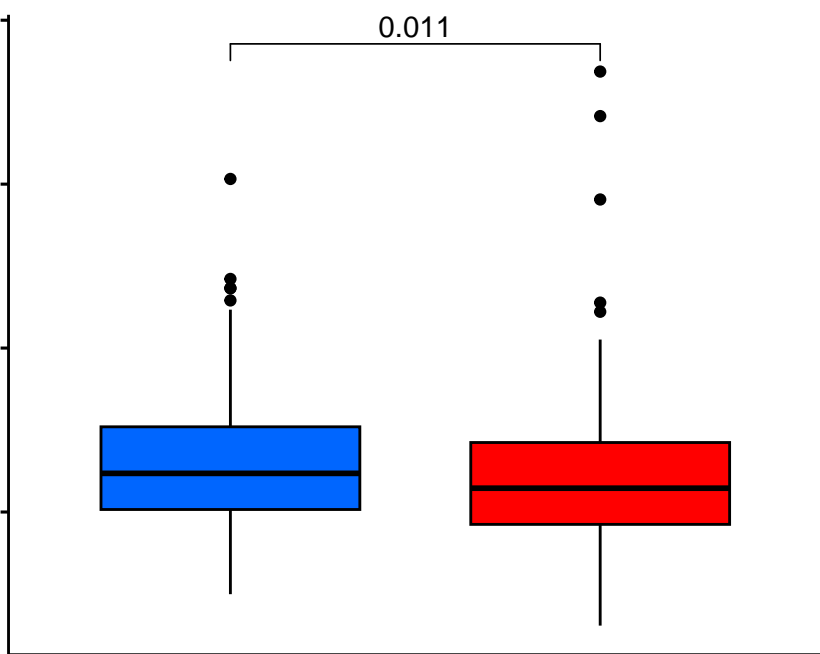

Supplement: Supplementary file 1 — Supplementary Information. [file 41598_2024_53257_MOESM1_ESM.zip › supplementary files/Drug sensitivity of C1 group and C2 group/C2 better/drugSenstivity.Wee1 Inhibitor.pdf]

Risk C1 C2

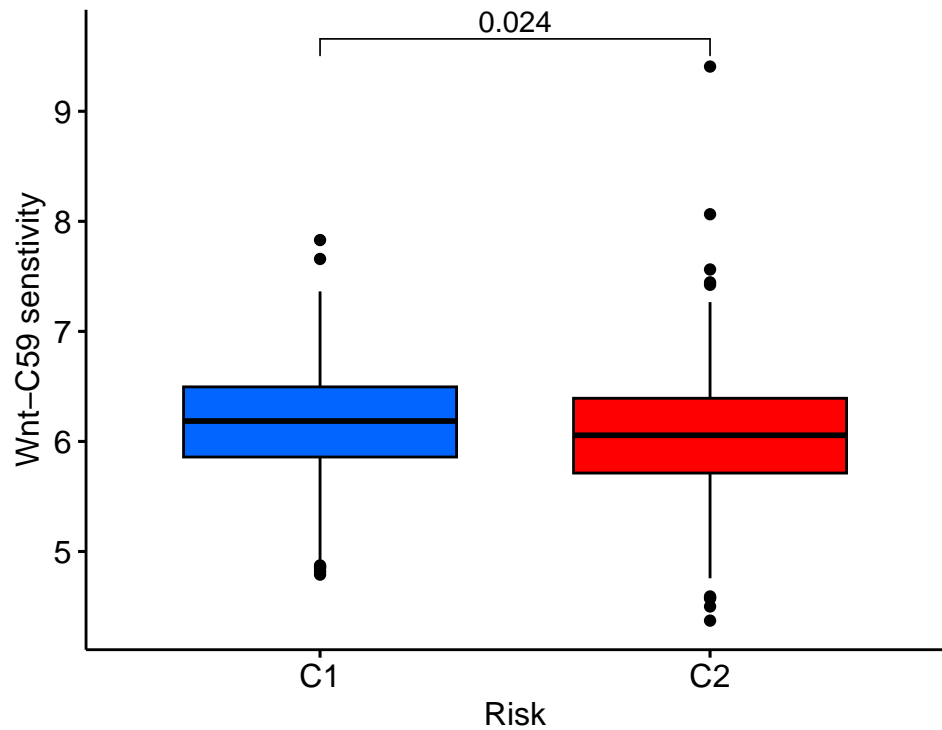

Supplement: Supplementary file 1 — Supplementary Information. [file 41598_2024_53257_MOESM1_ESM.zip › supplementary files/Drug sensitivity of C1 group and C2 group/C2 better/drugSenstivity.Wnt-C59.pdf]

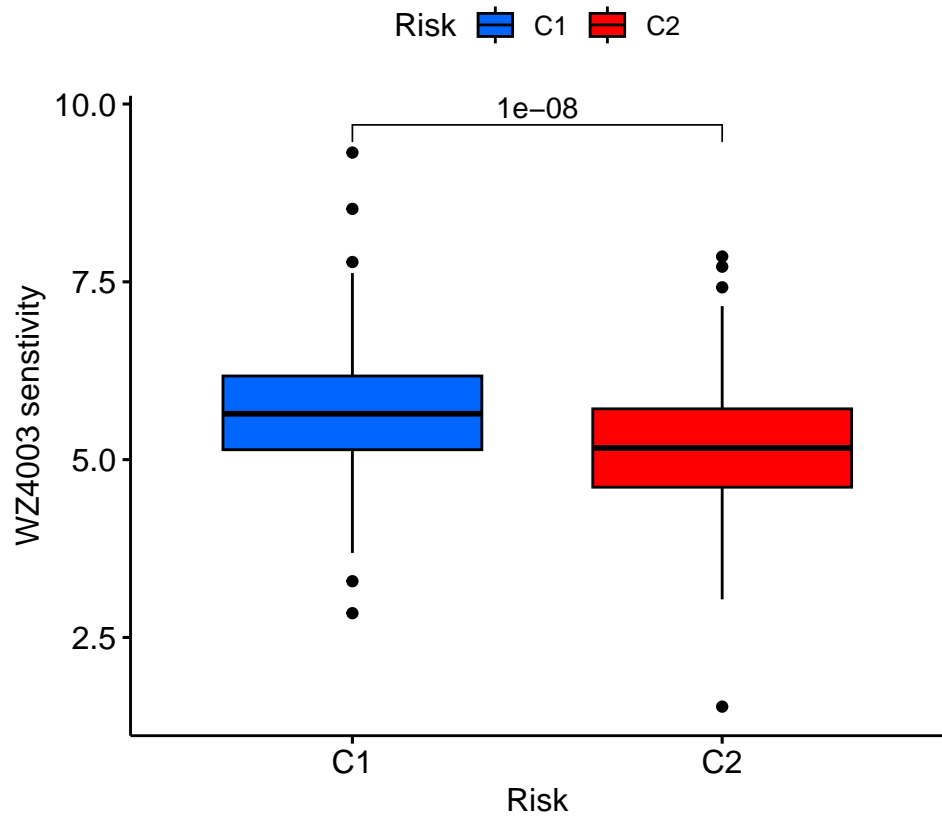

Supplement: Supplementary file 1 — Supplementary Information. [file 41598_2024_53257_MOESM1_ESM.zip › supplementary files/Drug sensitivity of C1 group and C2 group/C2 better/drugSenstivity.WZ4003.pdf]

XAV939 sensitivity

Risk C1 C2

$2e-07$

C1

C2

Risk

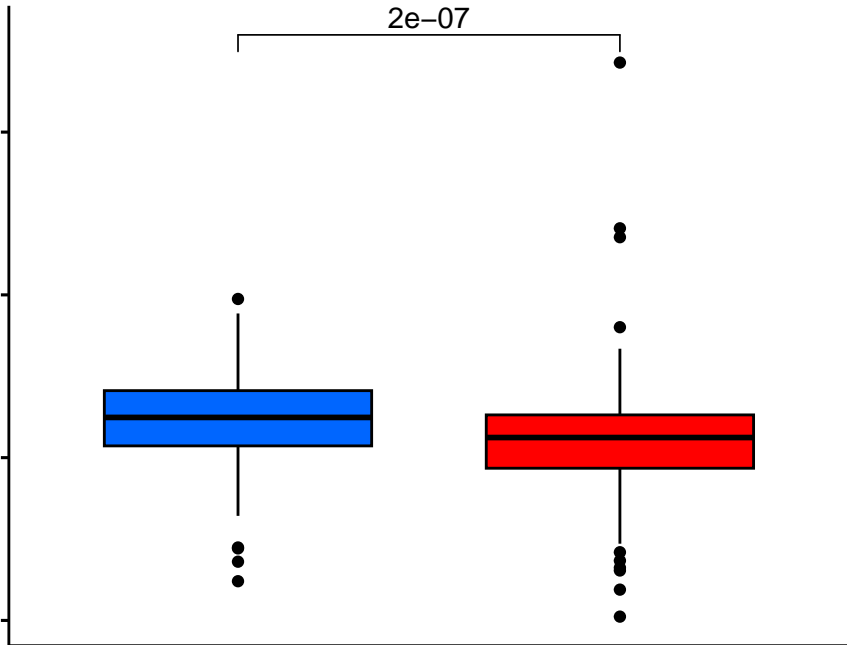

Supplement: Supplementary file 1 — Supplementary Information. [file 41598_2024_53257_MOESM1_ESM.zip › supplementary files/Drug sensitivity of C1 group and C2 group/C2 better/drugSenstivity.XAV939.pdf]

Risk C1 C2

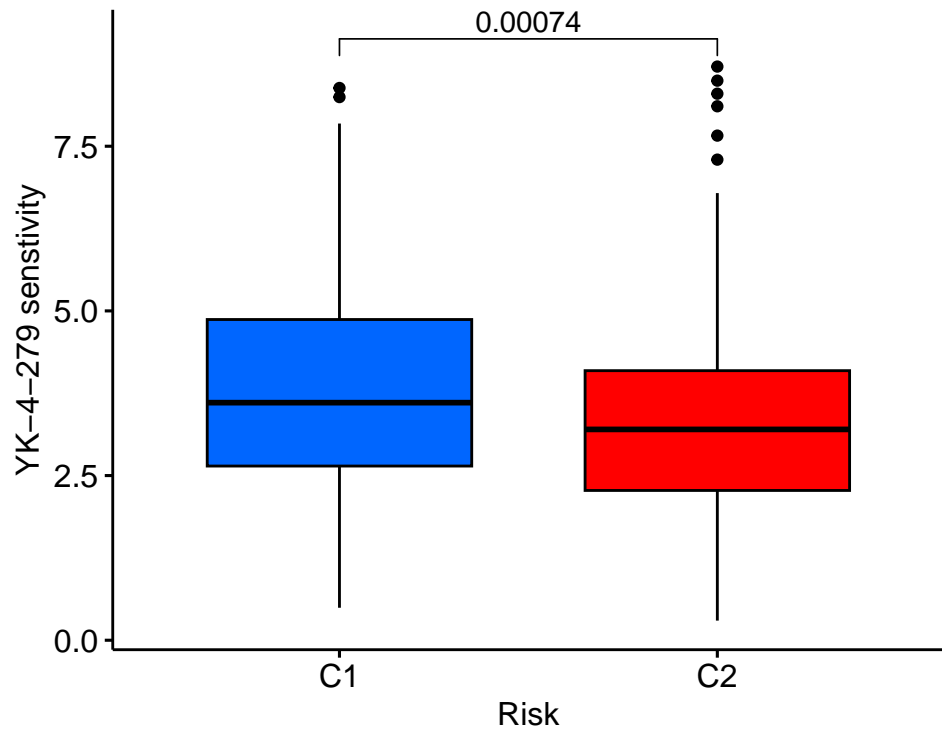

Supplement: Supplementary file 1 — Supplementary Information. [file 41598_2024_53257_MOESM1_ESM.zip › supplementary files/Drug sensitivity of C1 group and C2 group/C2 better/drugSenstivity.YK-4-279.pdf]

Risk 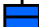 low 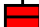 high

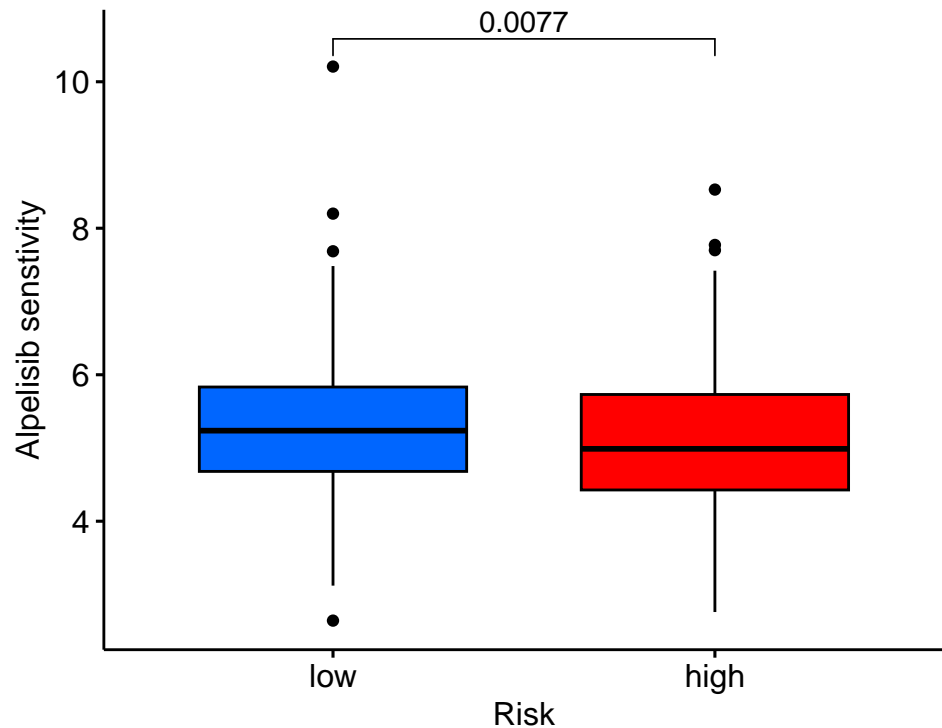

Supplement: Supplementary file 1 — Supplementary Information. [file 41598_2024_53257_MOESM1_ESM.zip › supplementary files/Drug sensitivity of low and high risk group/high risk group better/drugSenstivity.Alpelisib.pdf]

Risk 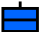 low 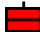 high

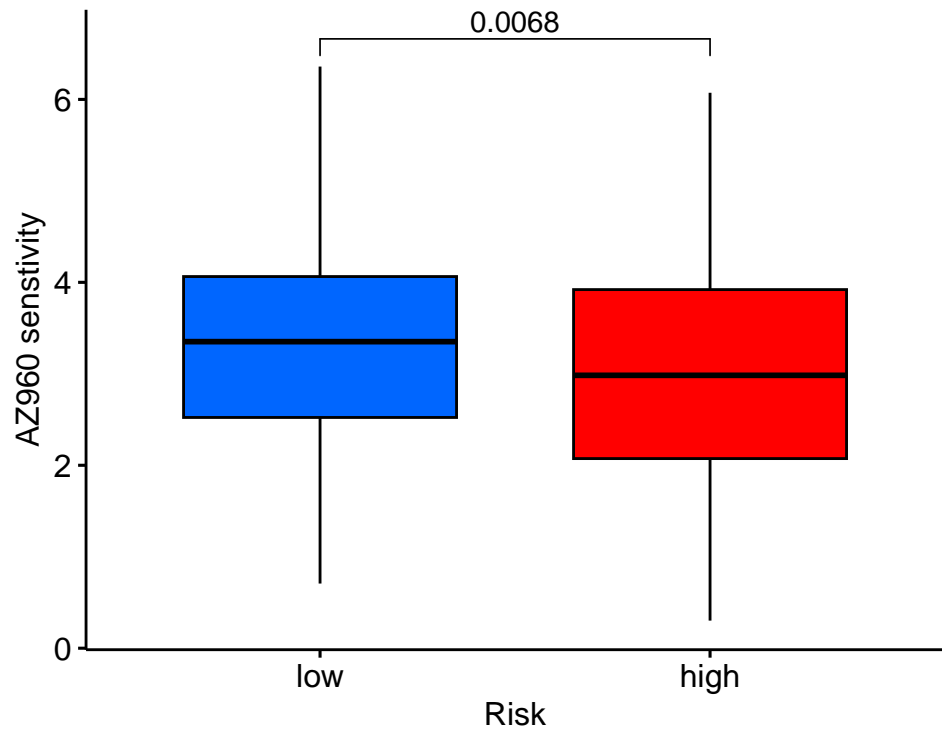

Supplement: Supplementary file 1 — Supplementary Information. [file 41598_2024_53257_MOESM1_ESM.zip › supplementary files/Drug sensitivity of low and high risk group/high risk group better/drugSenstivity.AZ960.pdf]

Risk 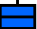 low 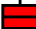 high

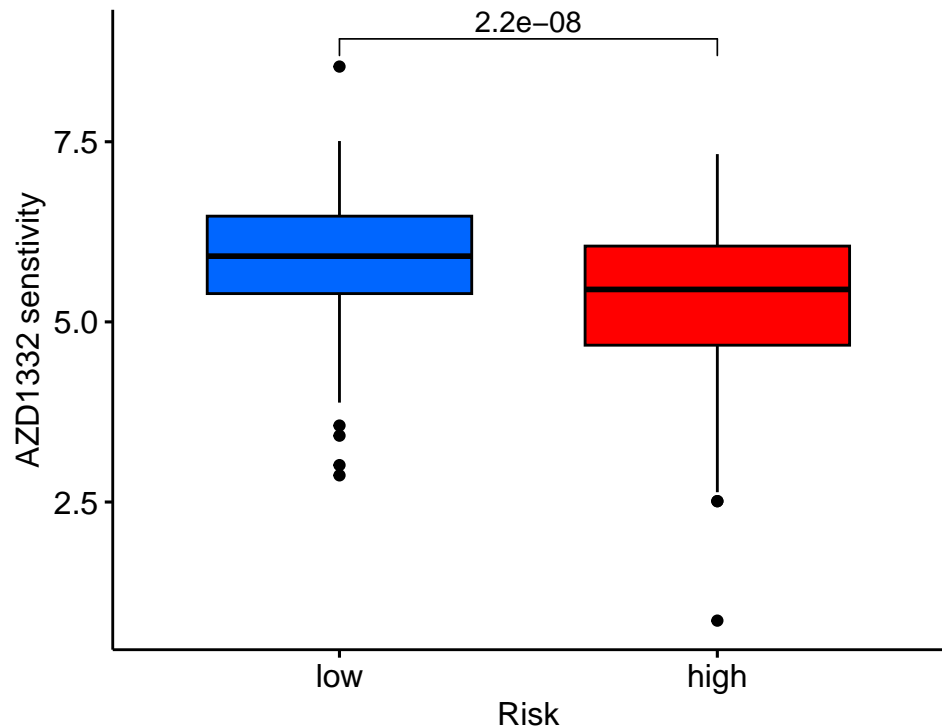

Supplement: Supplementary file 1 — Supplementary Information. [file 41598_2024_53257_MOESM1_ESM.zip › supplementary files/Drug sensitivity of low and high risk group/high risk group better/drugSenstivity.AZD1332.pdf]

Risk 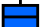 low 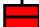 high

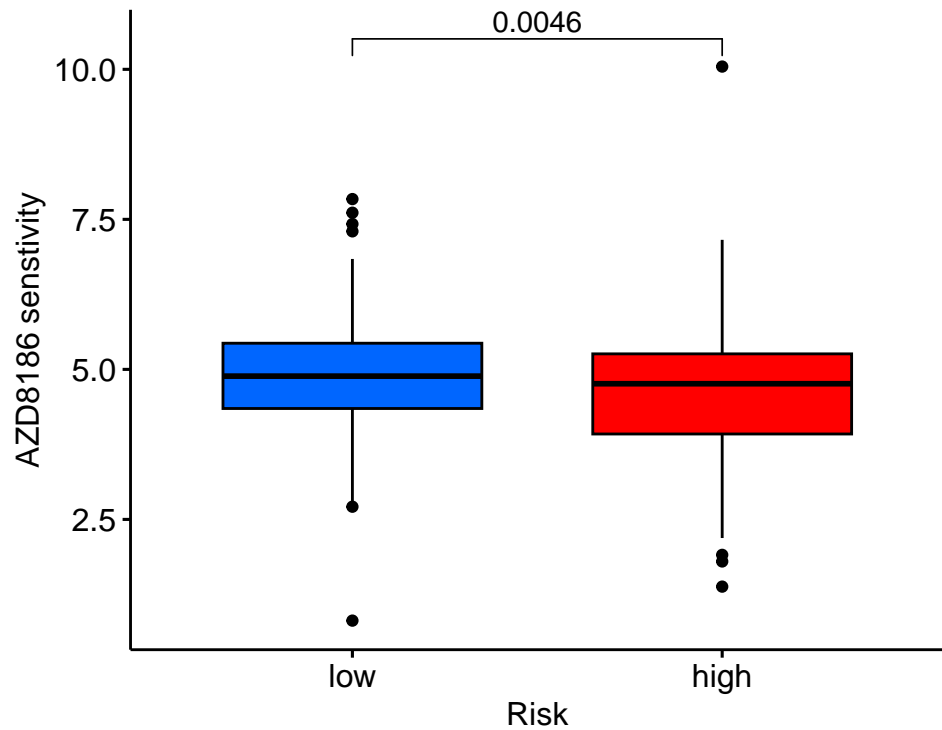

Supplement: Supplementary file 1 — Supplementary Information. [file 41598_2024_53257_MOESM1_ESM.zip › supplementary files/Drug sensitivity of low and high risk group/high risk group better/drugSenstivity.AZD8186.pdf]

Risk 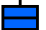 low 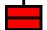 high

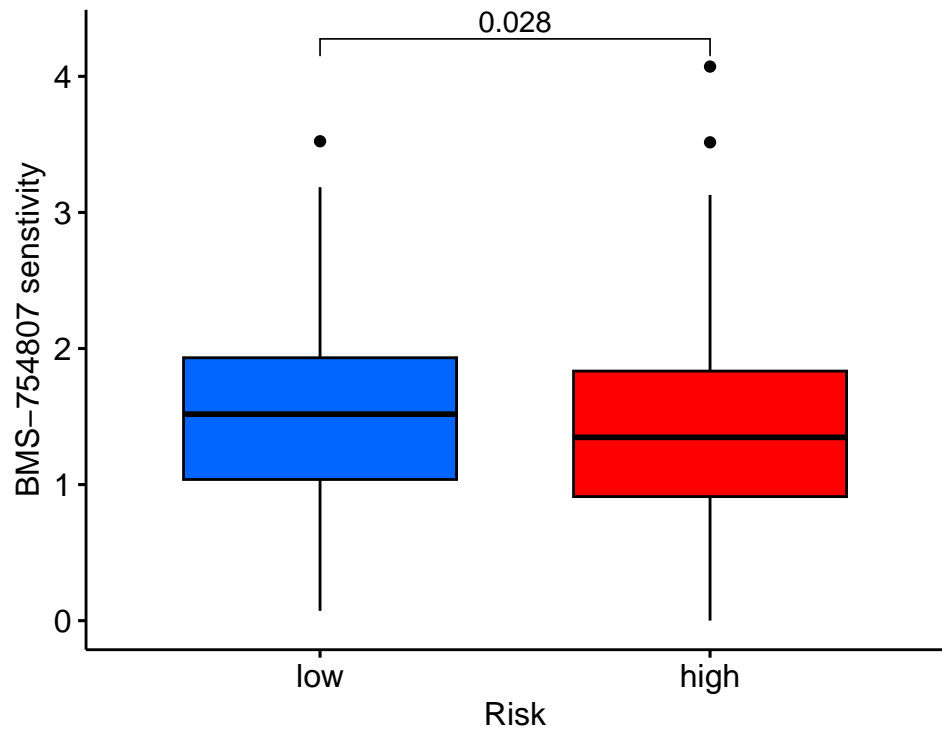

Supplement: Supplementary file 1 — Supplementary Information. [file 41598_2024_53257_MOESM1_ESM.zip › supplementary files/Drug sensitivity of low and high risk group/high risk group better/drugSenstivity.BMS-754807.pdf]

Risk 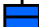 low 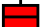 high

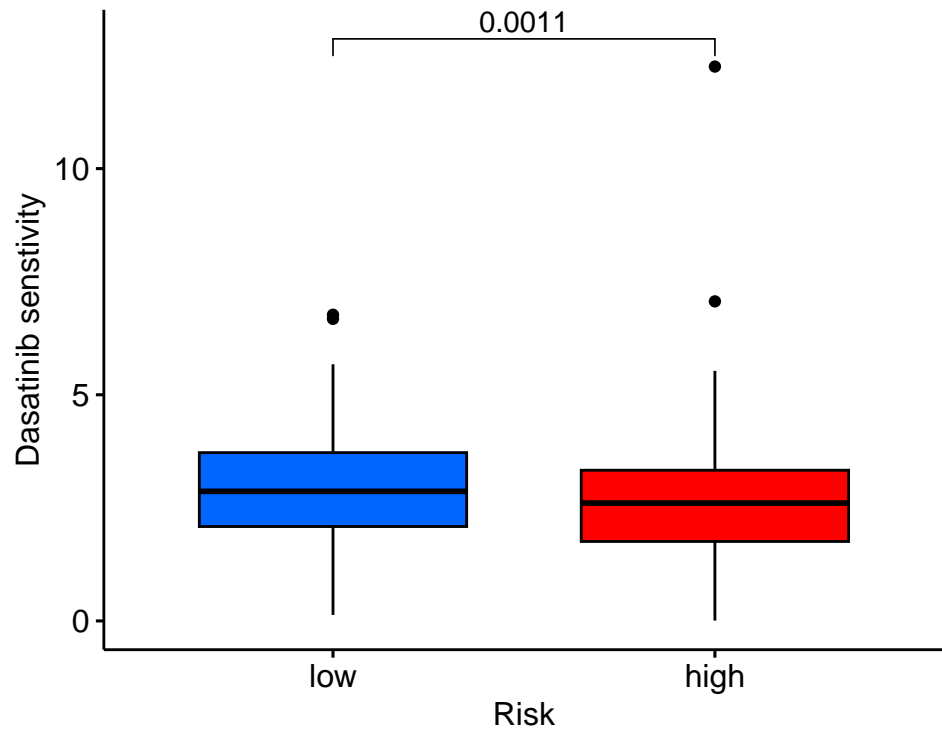

Supplement: Supplementary file 1 — Supplementary Information. [file 41598_2024_53257_MOESM1_ESM.zip › supplementary files/Drug sensitivity of low and high risk group/high risk group better/drugSenstivity.Dasatinib.pdf]

Risk 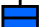 low 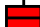 high

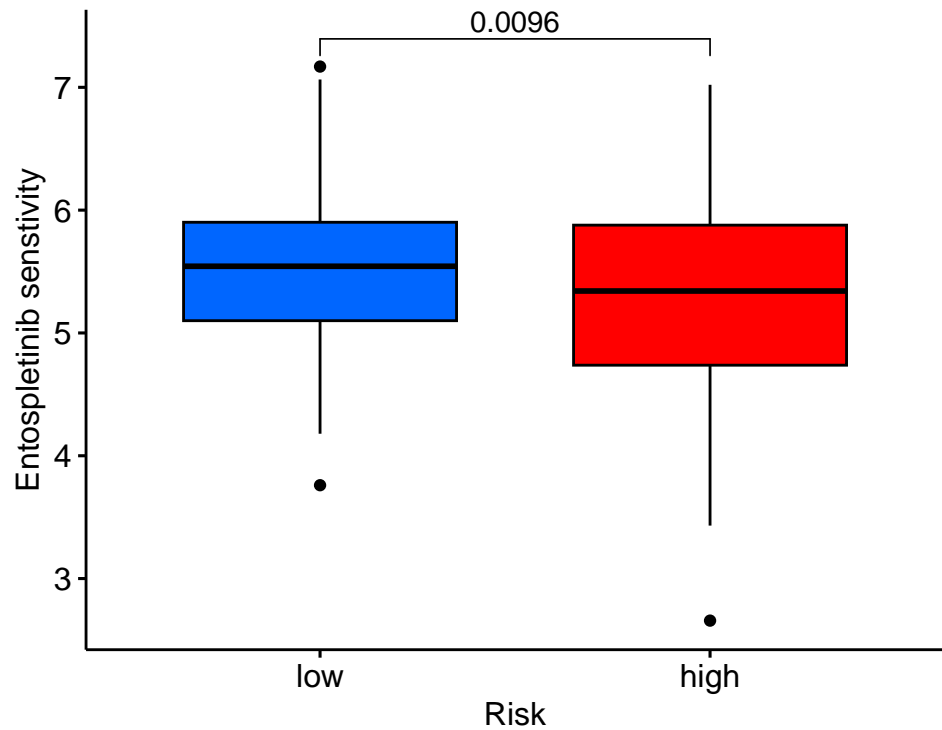

Supplement: Supplementary file 1 — Supplementary Information. [file 41598_2024_53257_MOESM1_ESM.zip › supplementary files/Drug sensitivity of low and high risk group/high risk group better/drugSenstivity.Entospletinib.pdf]

Risk 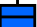 low 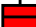 high

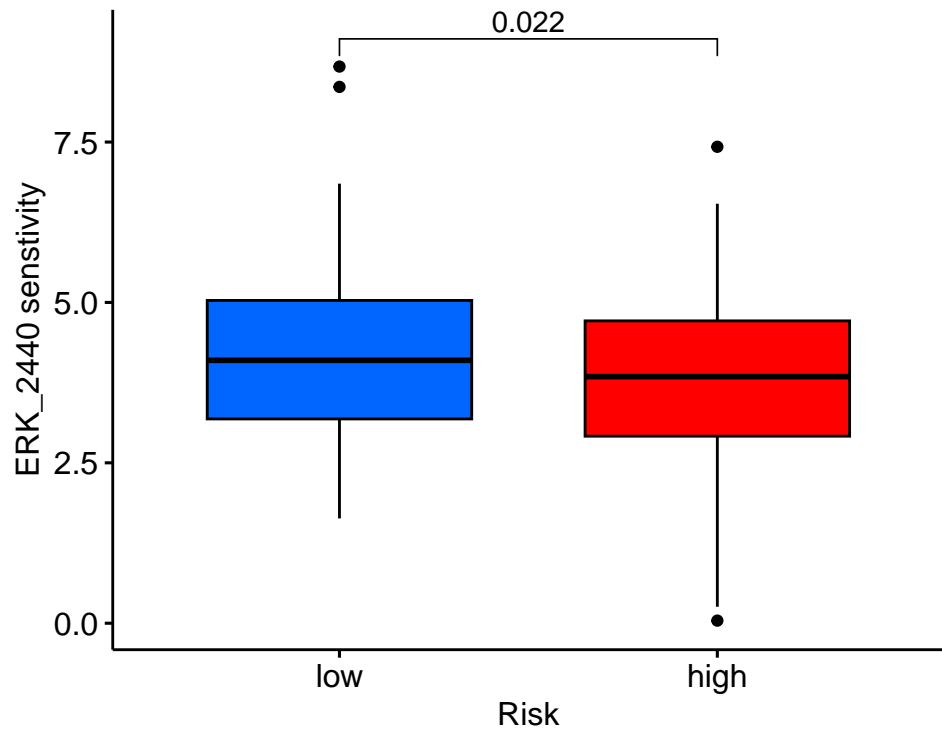

Supplement: Supplementary file 1 — Supplementary Information. [file 41598_2024_53257_MOESM1_ESM.zip › supplementary files/Drug sensitivity of low and high risk group/high risk group better/drugSenstivity.ERK_2440.pdf]

Risk 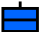 low 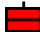 high

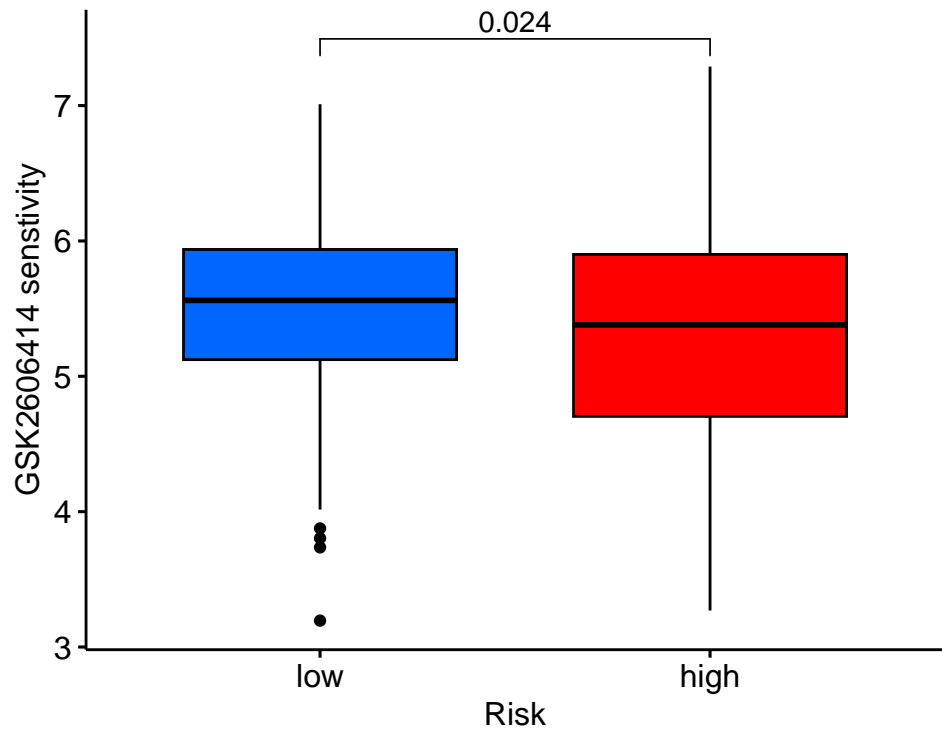

Supplement: Supplementary file 1 — Supplementary Information. [file 41598_2024_53257_MOESM1_ESM.zip › supplementary files/Drug sensitivity of low and high risk group/high risk group better/drugSenstivity.GSK2606414.pdf]

Risk low high

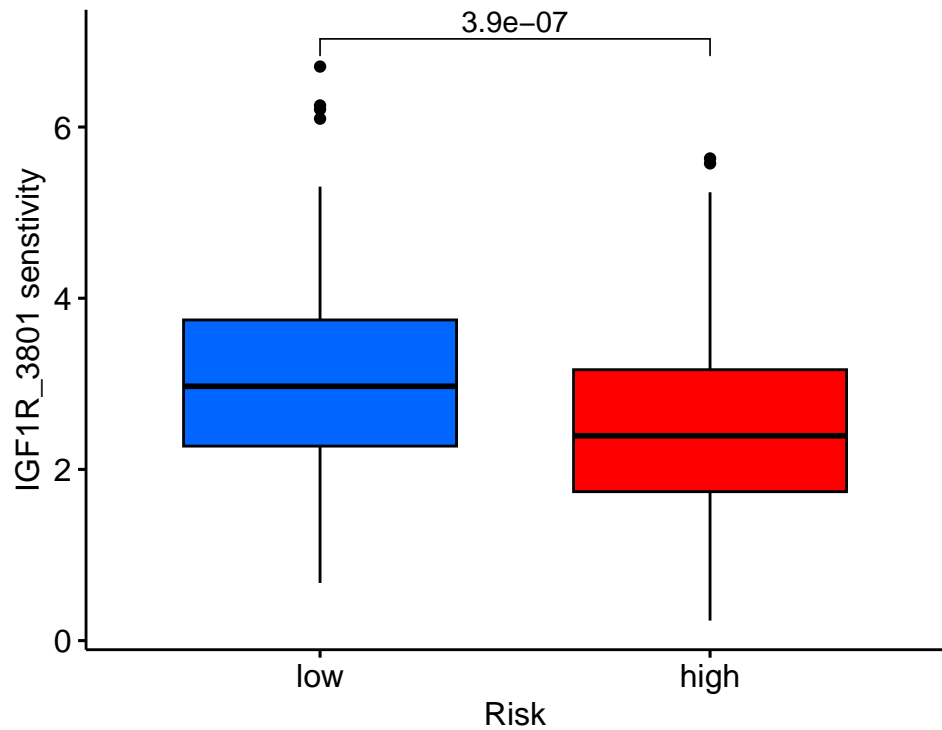

Supplement: Supplementary file 1 — Supplementary Information. [file 41598_2024_53257_MOESM1_ESM.zip › supplementary files/Drug sensitivity of low and high risk group/high risk group better/drugSenstivity.IGF1R_3801.pdf]

Risk 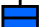 low 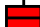 high

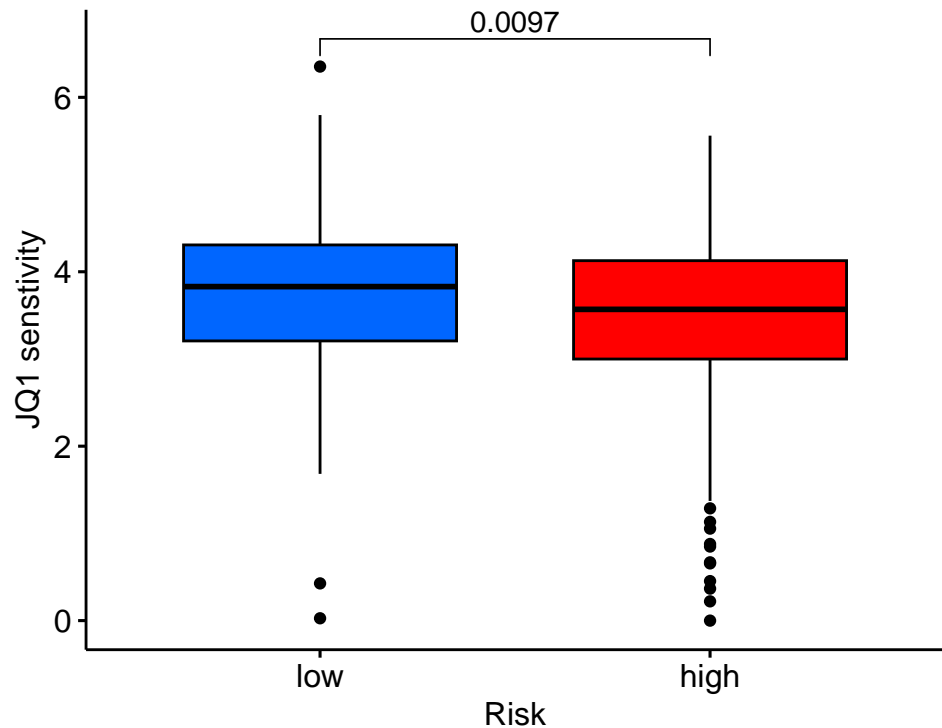

Supplement: Supplementary file 1 — Supplementary Information. [file 41598_2024_53257_MOESM1_ESM.zip › supplementary files/Drug sensitivity of low and high risk group/high risk group better/drugSenstivity.JQ1.pdf]

Risk 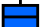 low 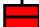 high

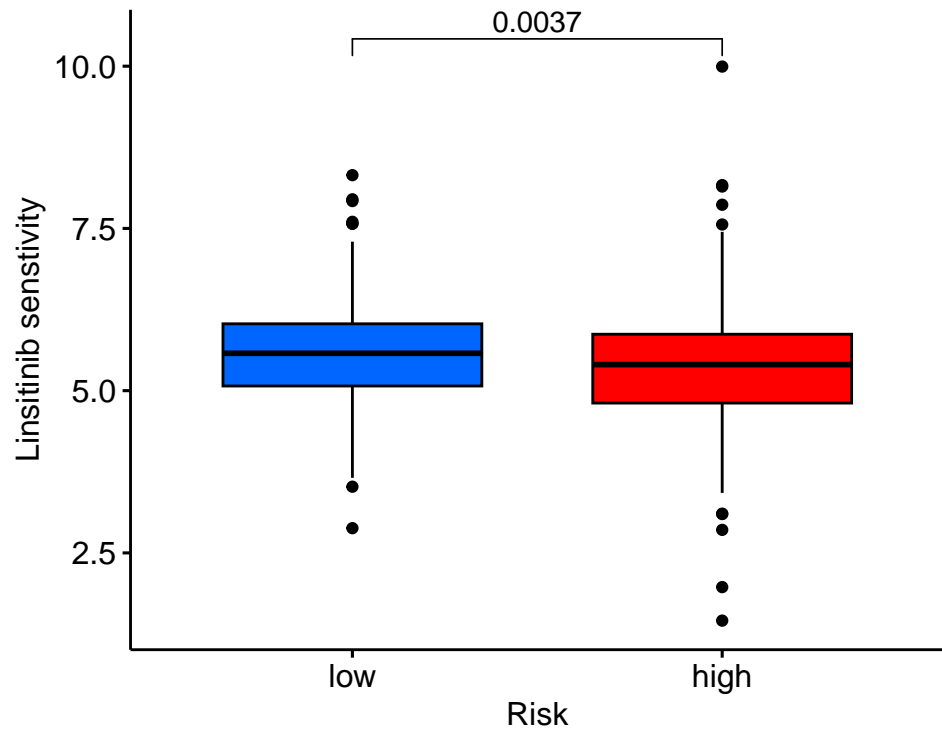

Supplement: Supplementary file 1 — Supplementary Information. [file 41598_2024_53257_MOESM1_ESM.zip › supplementary files/Drug sensitivity of low and high risk group/high risk group better/drugSenstivity.Linsitinib.pdf]

Risk 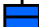 low 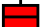 high

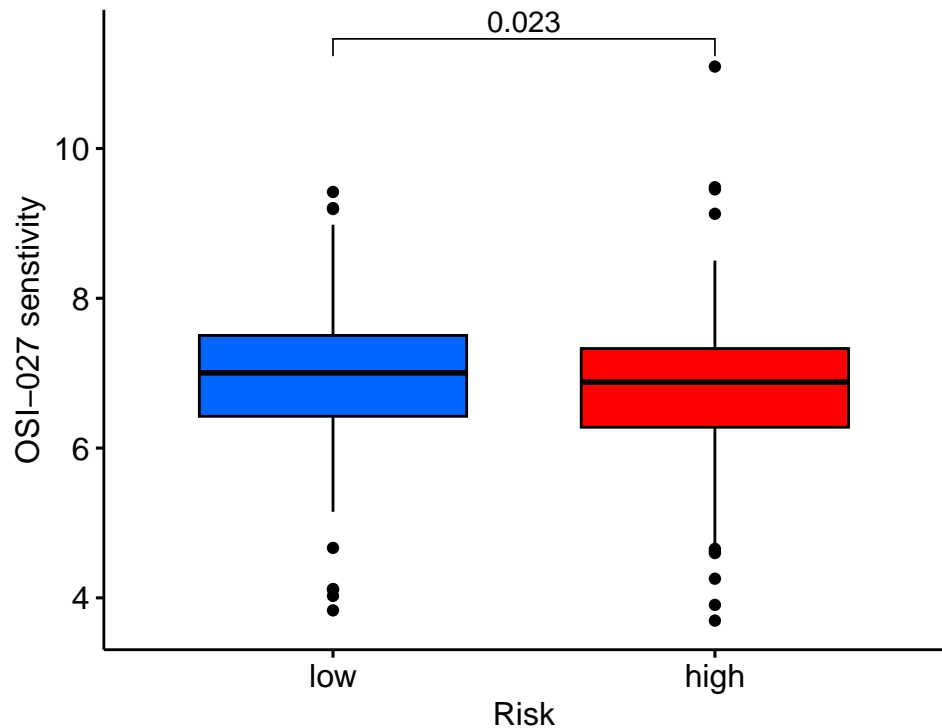

Supplement: Supplementary file 1 — Supplementary Information. [file 41598_2024_53257_MOESM1_ESM.zip › supplementary files/Drug sensitivity of low and high risk group/high risk group better/drugSenstivity.OSI-027.pdf]

Risk 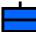 low 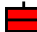 high

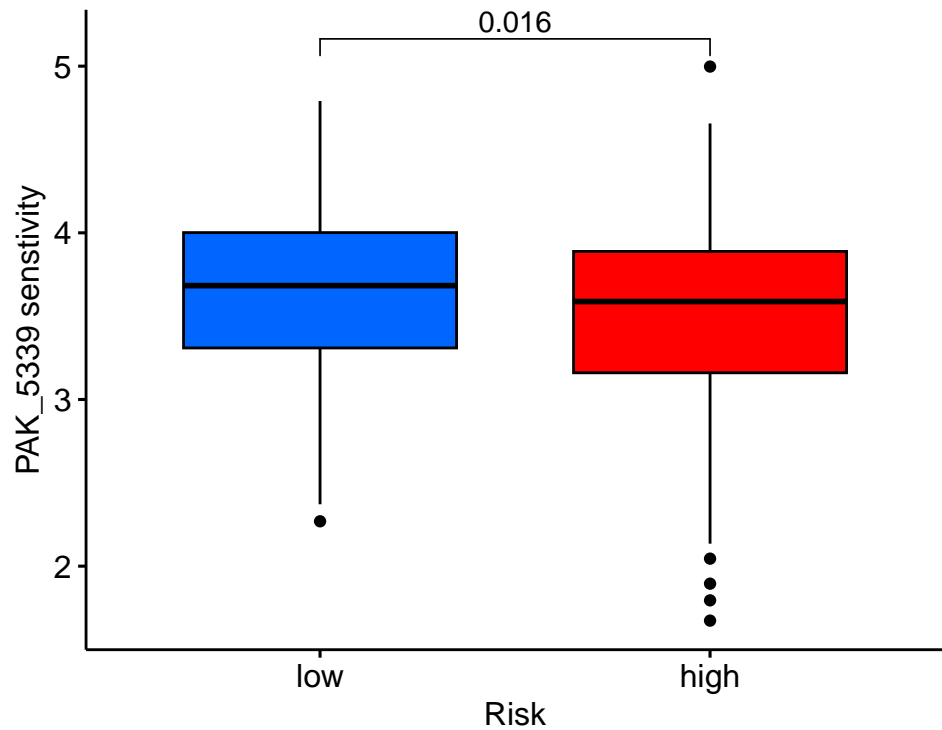

Supplement: Supplementary file 1 — Supplementary Information. [file 41598_2024_53257_MOESM1_ESM.zip › supplementary files/Drug sensitivity of low and high risk group/high risk group better/drugSenstivity.PAK_5339.pdf]

Risk 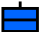 low 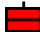 high

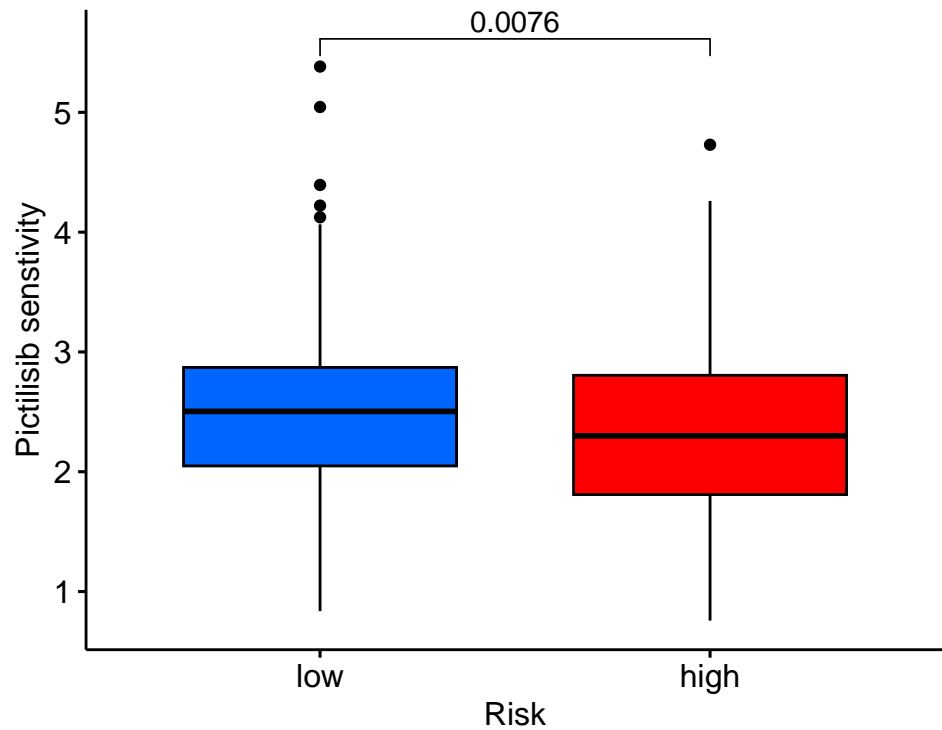

Supplement: Supplementary file 1 — Supplementary Information. [file 41598_2024_53257_MOESM1_ESM.zip › supplementary files/Drug sensitivity of low and high risk group/high risk group better/drugSenstivity.Pictilisib.pdf]

Risk 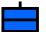 low 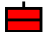 high

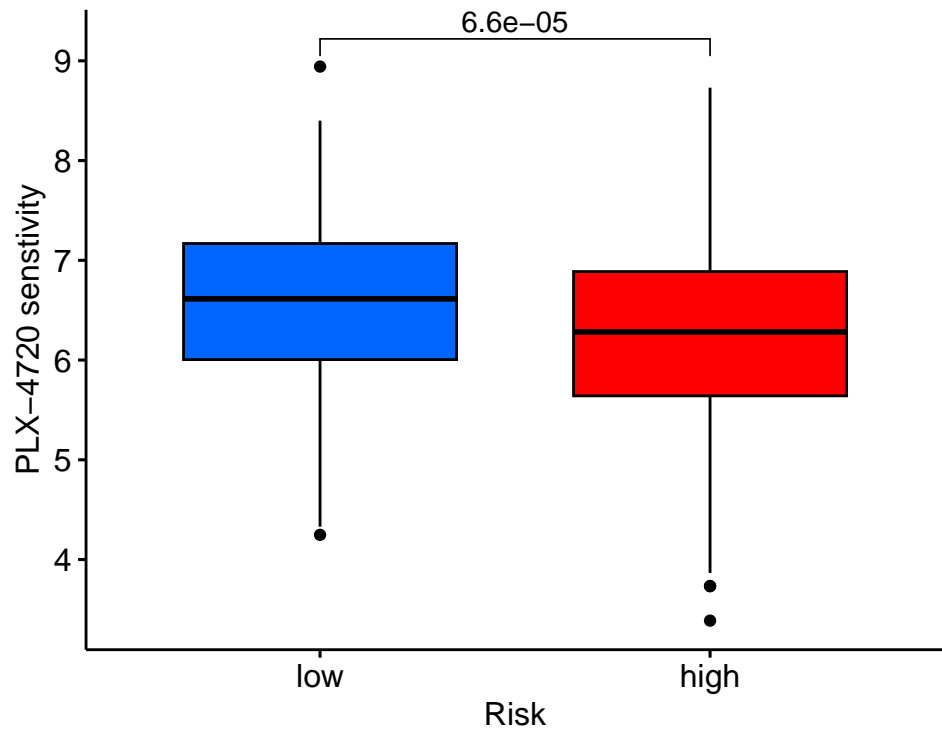

Supplement: Supplementary file 1 — Supplementary Information. [file 41598_2024_53257_MOESM1_ESM.zip › supplementary files/Drug sensitivity of low and high risk group/high risk group better/drugSenstivity.PLX-4720.pdf]

Risk 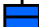 low 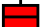 high

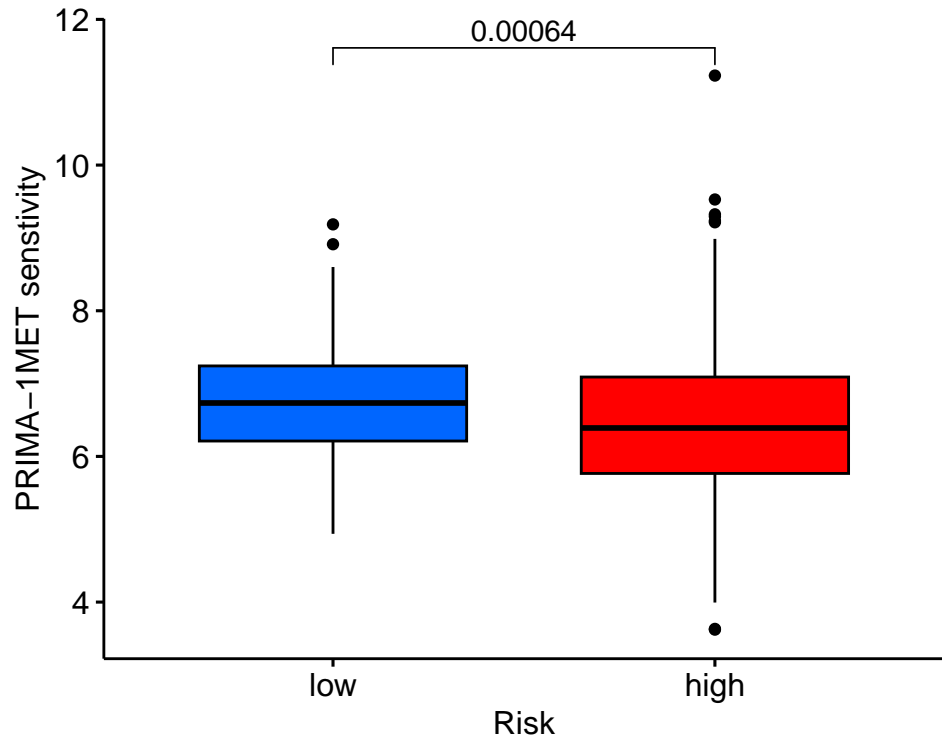

Supplement: Supplementary file 1 — Supplementary Information. [file 41598_2024_53257_MOESM1_ESM.zip › supplementary files/Drug sensitivity of low and high risk group/high risk group better/drugSenstivity.PRIMA-1MET.pdf]

Risk 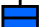 low 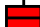 high

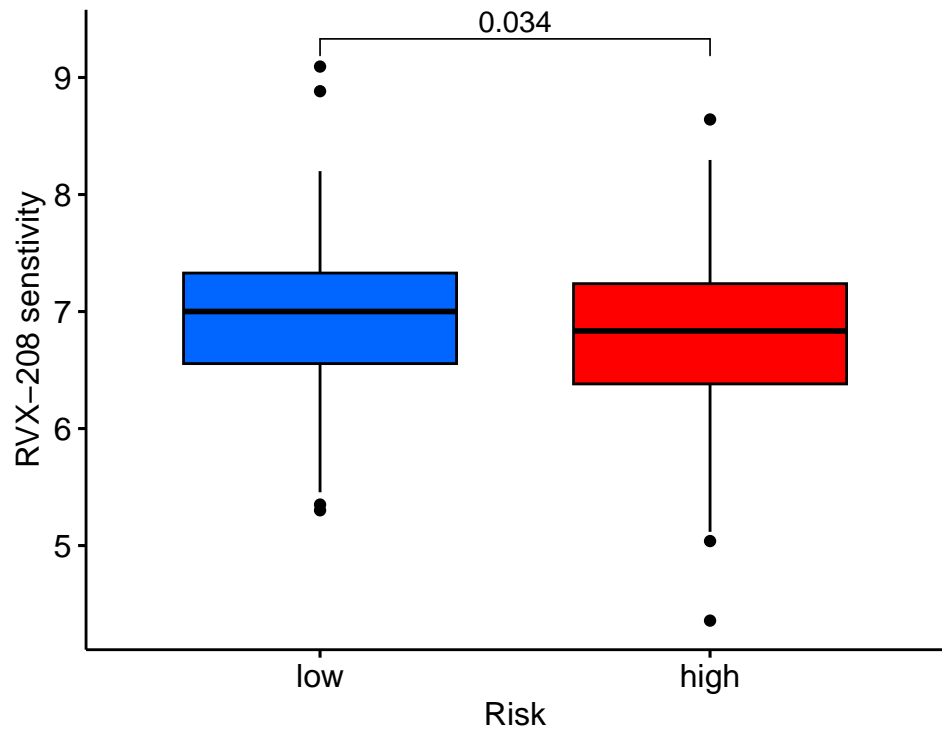

Supplement: Supplementary file 1 — Supplementary Information. [file 41598_2024_53257_MOESM1_ESM.zip › supplementary files/Drug sensitivity of low and high risk group/high risk group better/drugSenstivity.RVX-208.pdf]

Risk 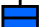 low 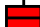 high

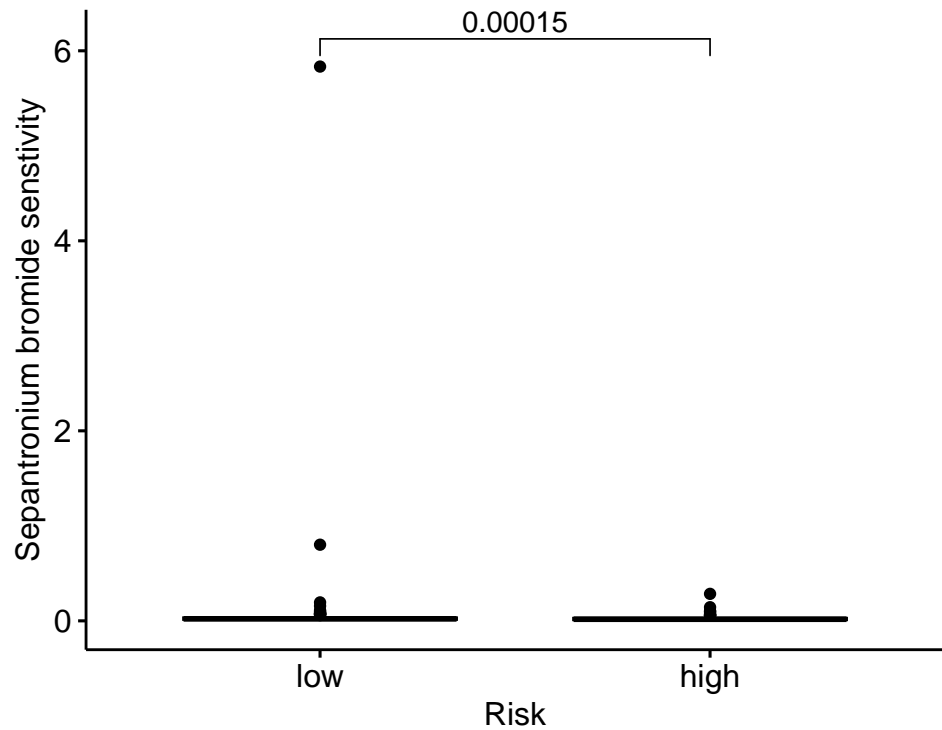

Supplement: Supplementary file 1 — Supplementary Information. [file 41598_2024_53257_MOESM1_ESM.zip › supplementary files/Drug sensitivity of low and high risk group/high risk group better/drugSenstivity.Sepantronium bromide.pdf]

Risk 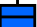 low 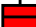 high

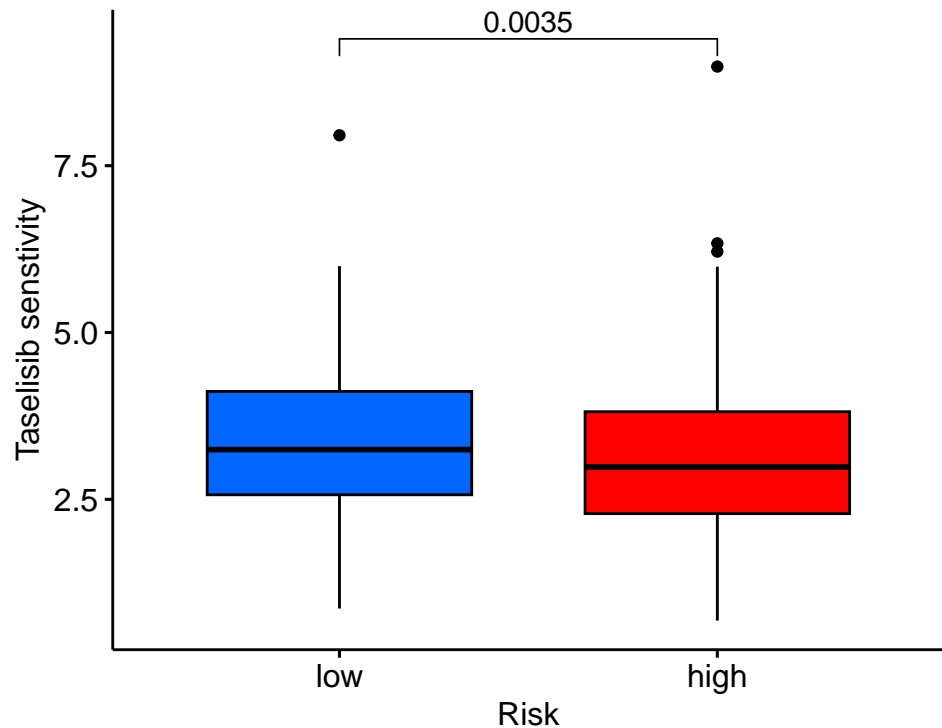

Supplement: Supplementary file 1 — Supplementary Information. [file 41598_2024_53257_MOESM1_ESM.zip › supplementary files/Drug sensitivity of low and high risk group/high risk group better/drugSenstivity.Taselisib.pdf]

Risk 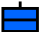 low 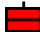 high

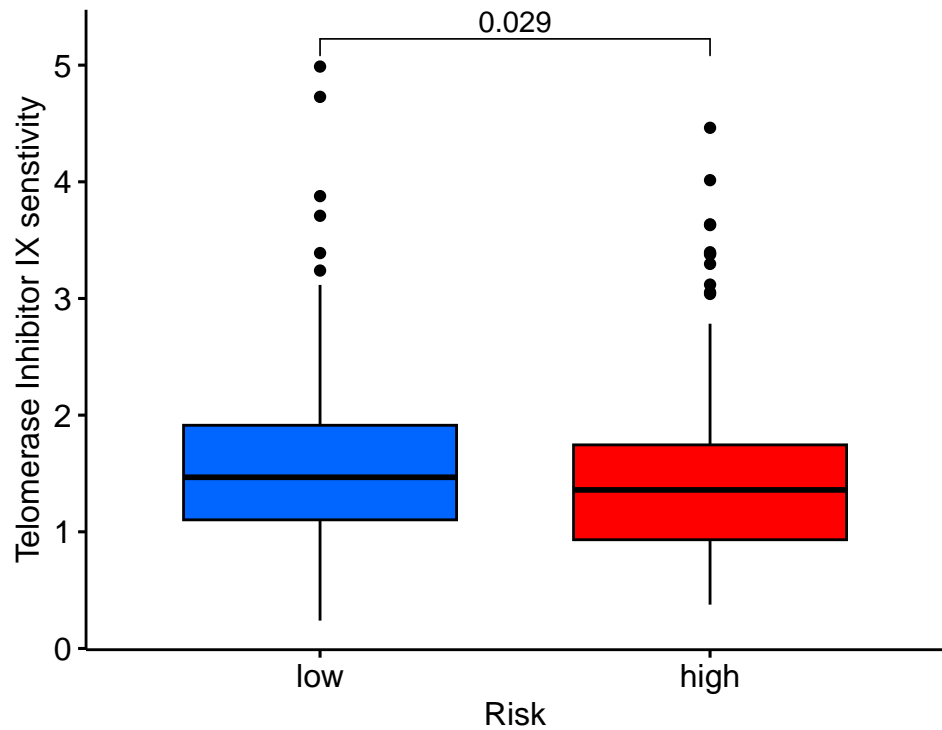

Supplement: Supplementary file 1 — Supplementary Information. [file 41598_2024_53257_MOESM1_ESM.zip › supplementary files/Drug sensitivity of low and high risk group/high risk group better/drugSenstivity.Telomerase Inhibitor IX.pdf]

Risk 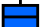 low 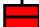 high

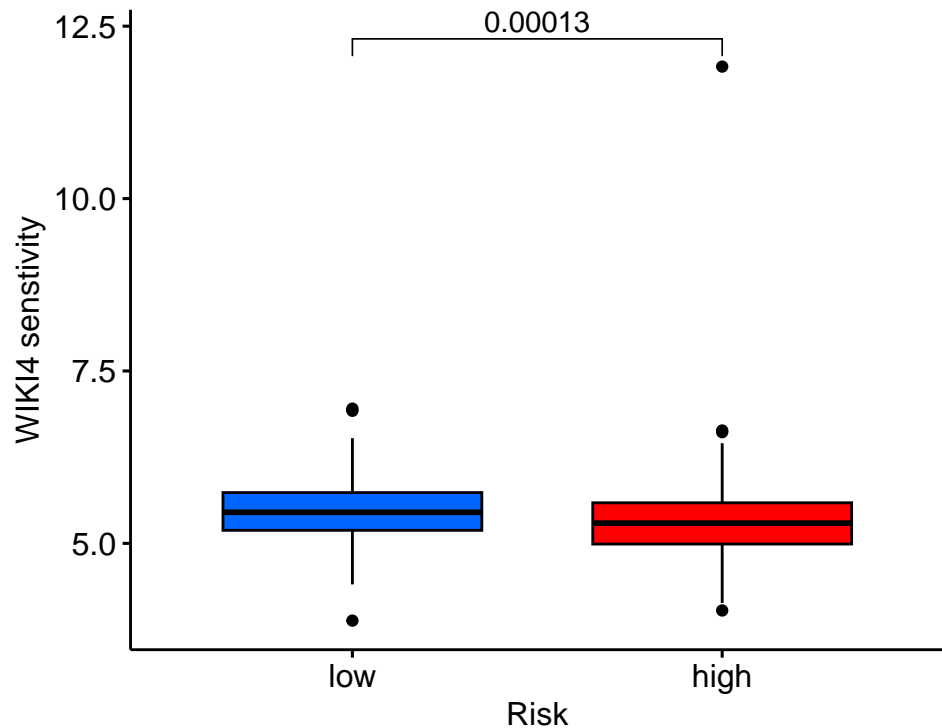

Supplement: Supplementary file 1 — Supplementary Information. [file 41598_2024_53257_MOESM1_ESM.zip › supplementary files/Drug sensitivity of low and high risk group/high risk group better/drugSenstivity.WIKI4.pdf]

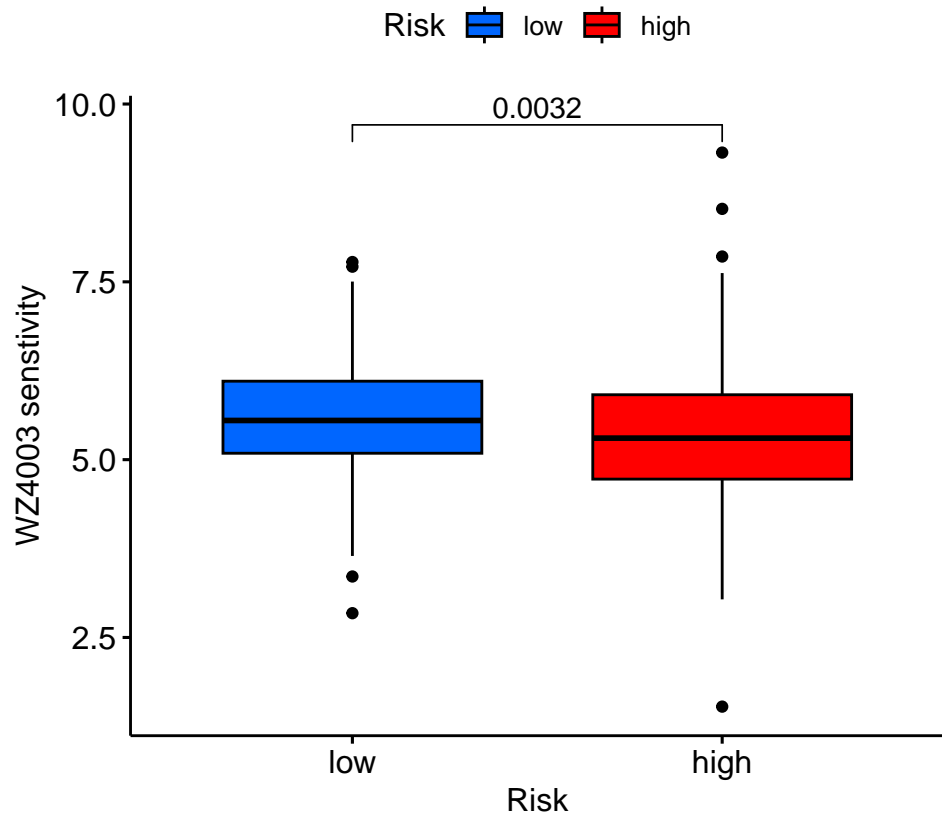

Supplement: Supplementary file 1 — Supplementary Information. [file 41598_2024_53257_MOESM1_ESM.zip › supplementary files/Drug sensitivity of low and high risk group/high risk group better/drugSenstivity.WZ4003.pdf]

Risk 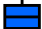 low 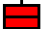 high

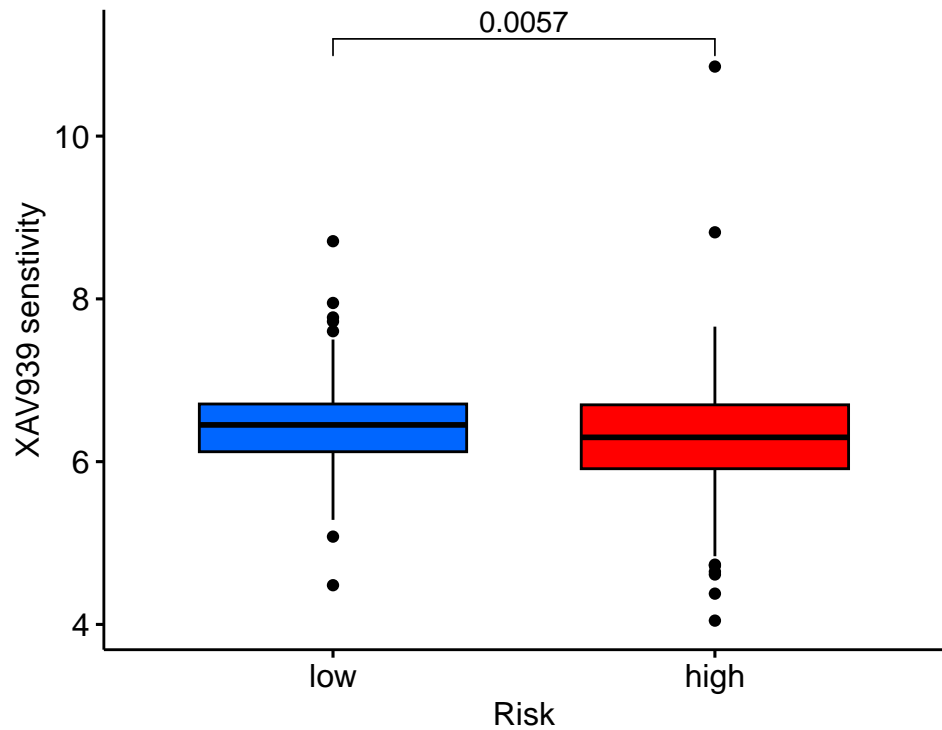

Supplement: Supplementary file 1 — Supplementary Information. [file 41598_2024_53257_MOESM1_ESM.zip › supplementary files/Drug sensitivity of low and high risk group/high risk group better/drugSenstivity.XAV939.pdf]

Risk 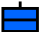 low 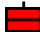 high

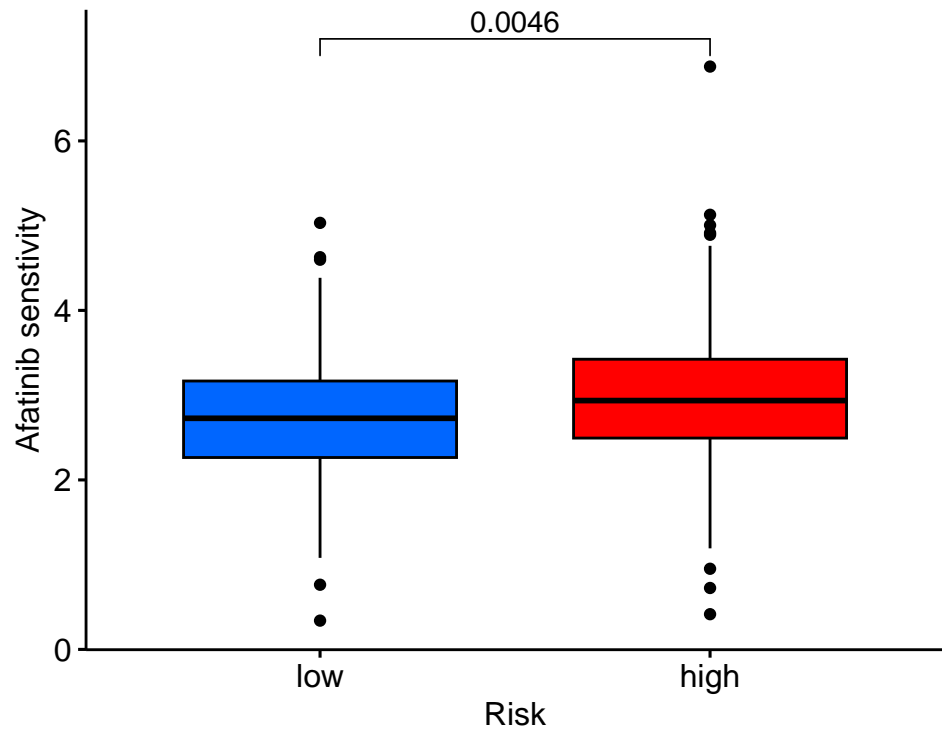

Supplement: Supplementary file 1 — Supplementary Information. [file 41598_2024_53257_MOESM1_ESM.zip › supplementary files/Drug sensitivity of low and high risk group/low risk group better/drugSenstivity.Afatinib.pdf]

Risk low high

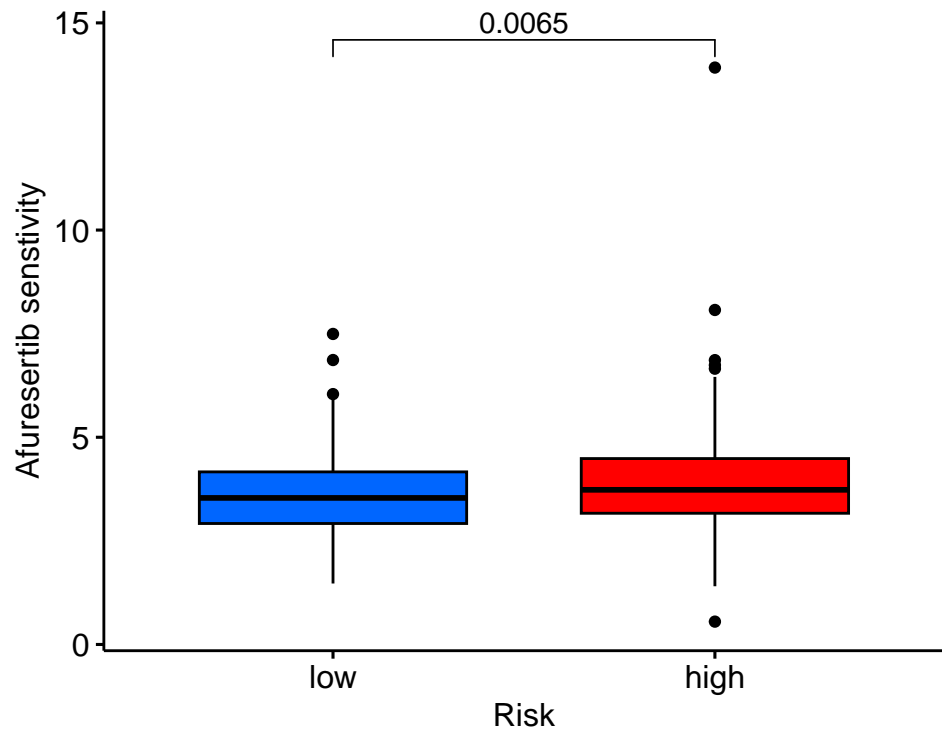

Supplement: Supplementary file 1 — Supplementary Information. [file 41598_2024_53257_MOESM1_ESM.zip › supplementary files/Drug sensitivity of low and high risk group/low risk group better/drugSenstivity.Afuresertib.pdf]

Risk 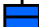 low 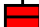 high

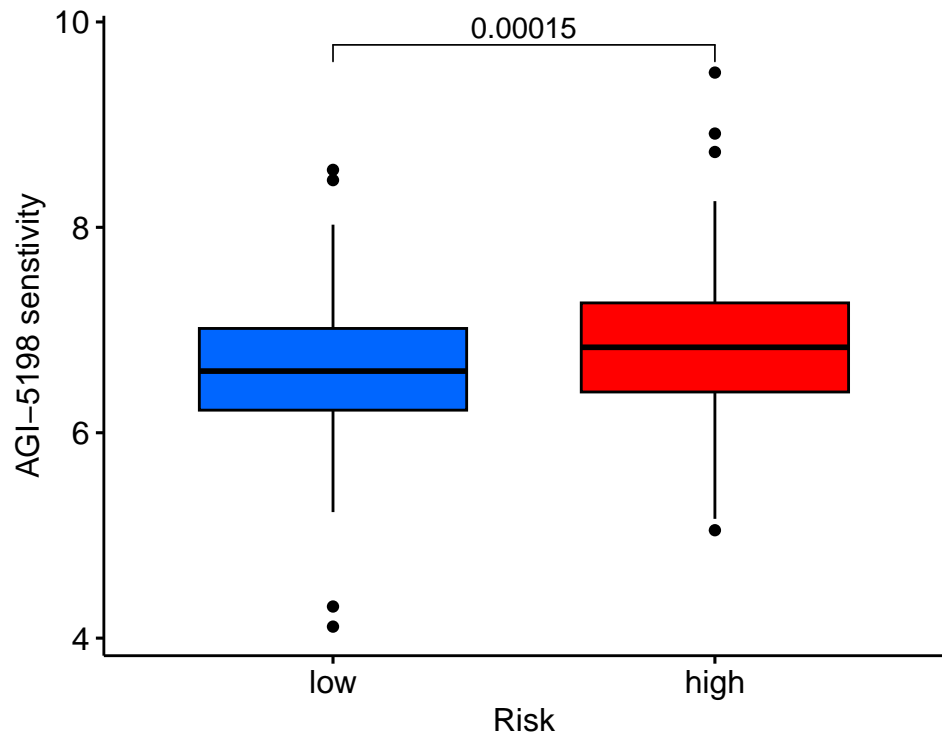

Supplement: Supplementary file 1 — Supplementary Information. [file 41598_2024_53257_MOESM1_ESM.zip › supplementary files/Drug sensitivity of low and high risk group/low risk group better/drugSenstivity.AGI-5198.pdf]

Risk low high

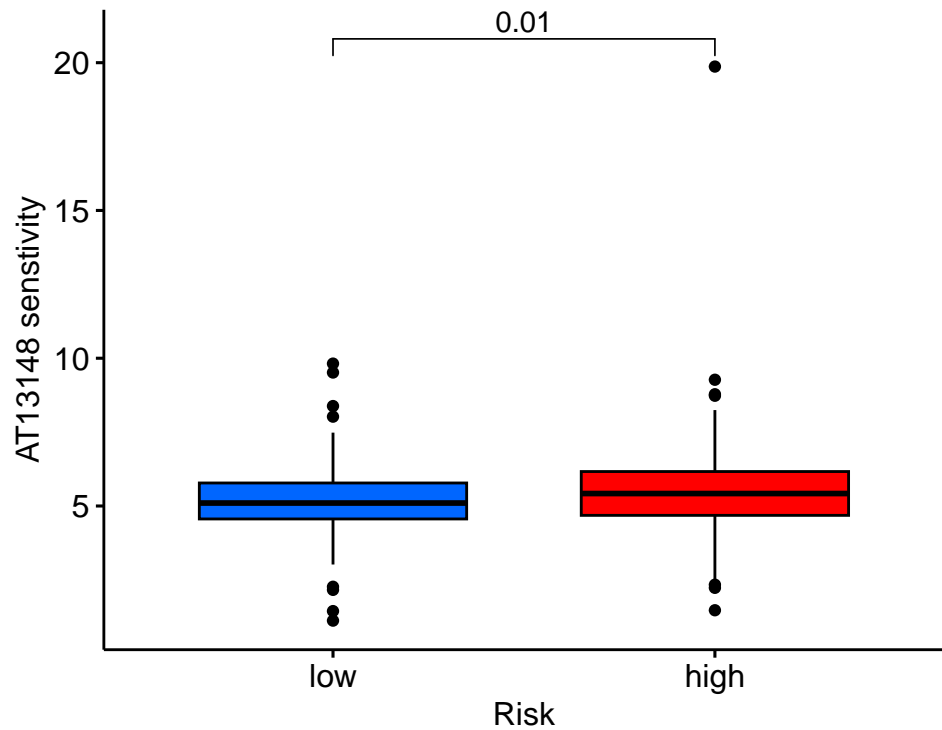

Supplement: Supplementary file 1 — Supplementary Information. [file 41598_2024_53257_MOESM1_ESM.zip › supplementary files/Drug sensitivity of low and high risk group/low risk group better/drugSenstivity.AT13148.pdf]

Risk 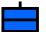 low 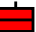 high

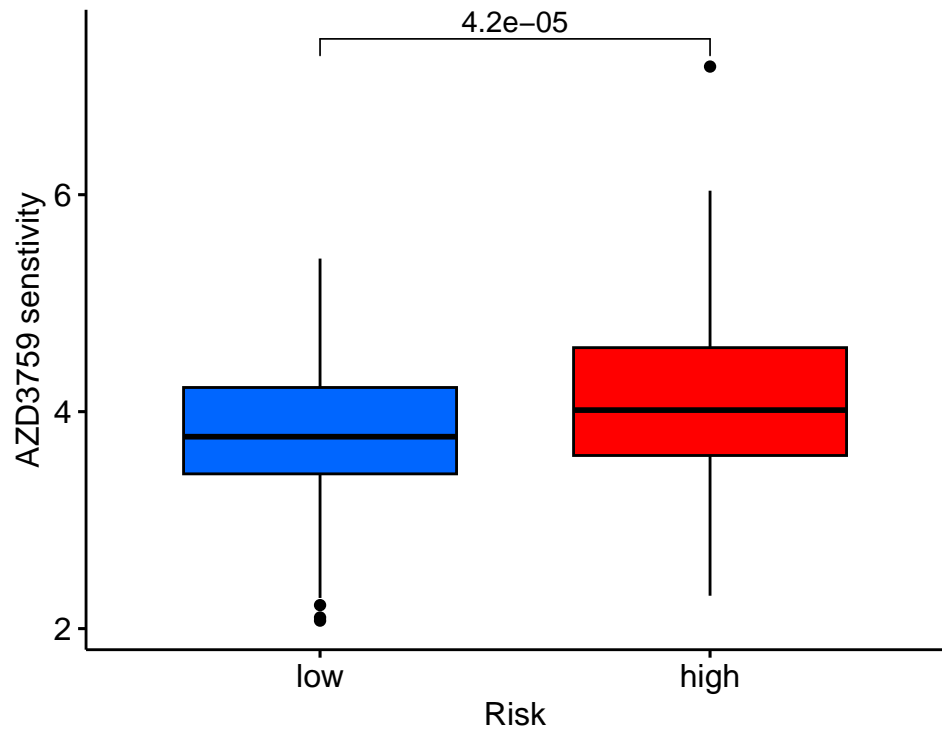

Supplement: Supplementary file 1 — Supplementary Information. [file 41598_2024_53257_MOESM1_ESM.zip › supplementary files/Drug sensitivity of low and high risk group/low risk group better/drugSenstivity.AZD3759.pdf]

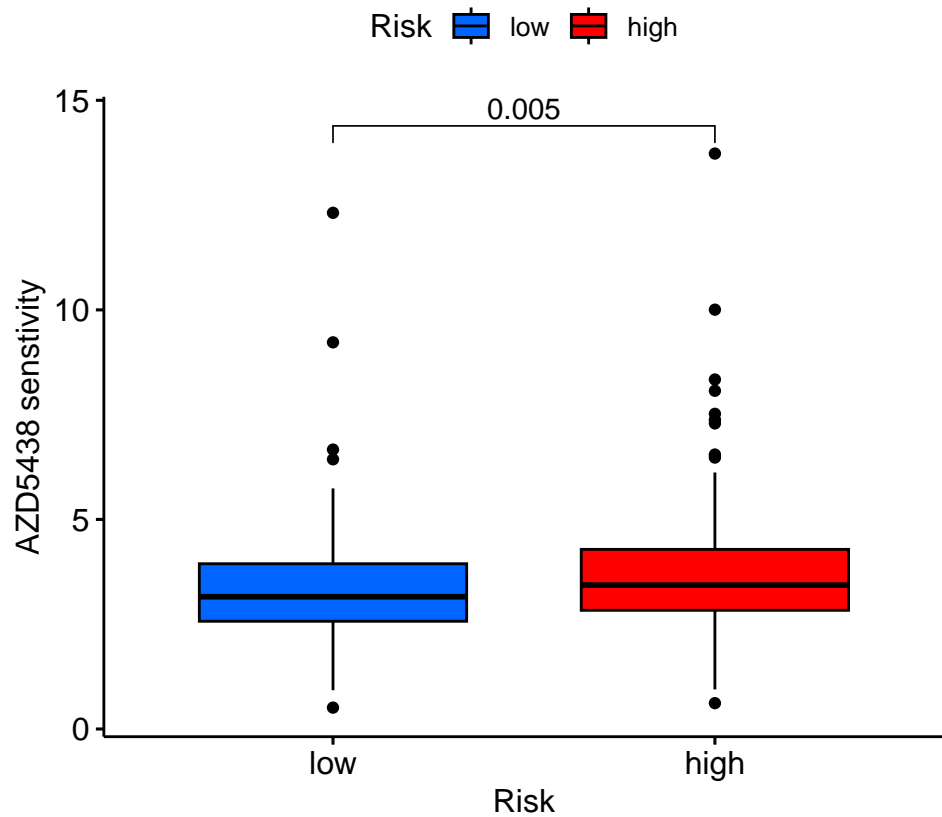

Supplement: Supplementary file 1 — Supplementary Information. [file 41598_2024_53257_MOESM1_ESM.zip › supplementary files/Drug sensitivity of low and high risk group/low risk group better/drugSenstivity.AZD5438.pdf]

Risk 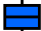 low 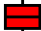 high

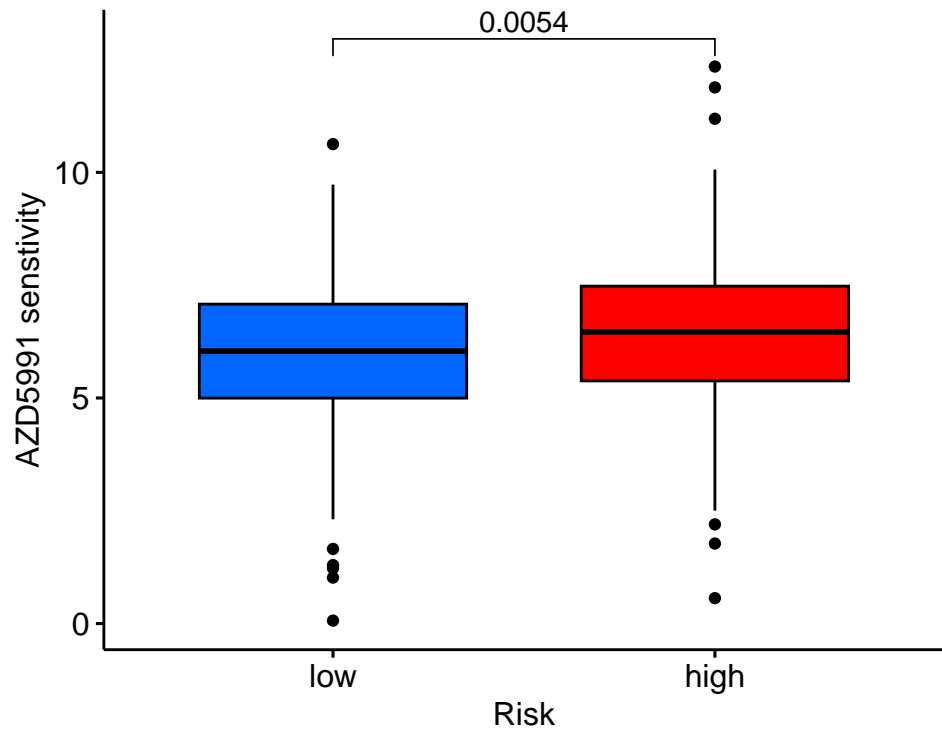

Supplement: Supplementary file 1 — Supplementary Information. [file 41598_2024_53257_MOESM1_ESM.zip › supplementary files/Drug sensitivity of low and high risk group/low risk group better/drugSenstivity.AZD5991.pdf]

Risk low high

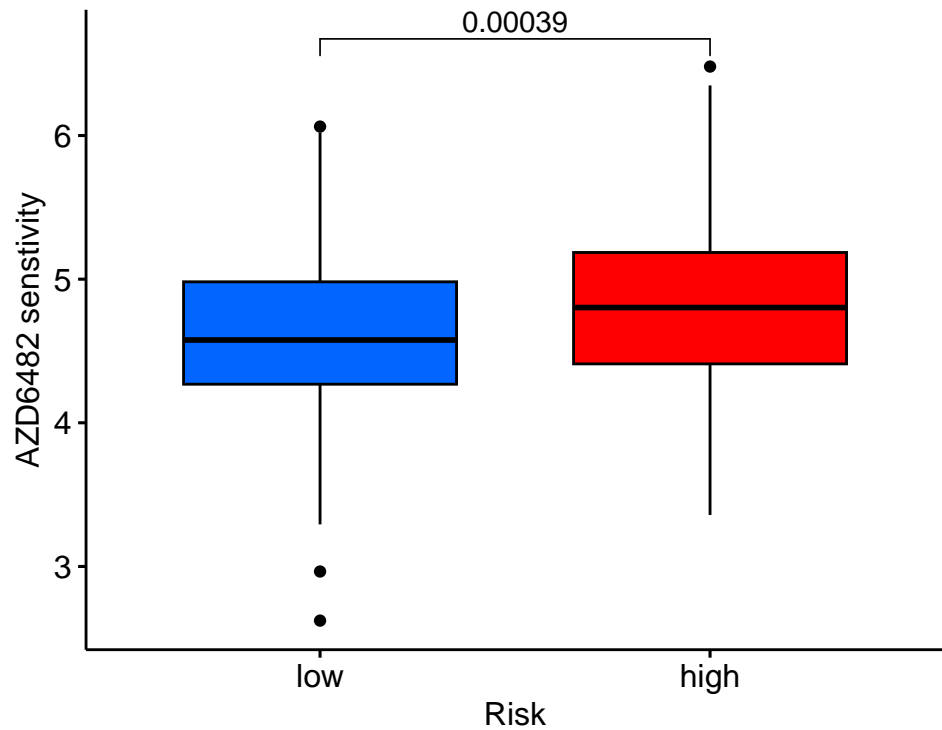

Supplement: Supplementary file 1 — Supplementary Information. [file 41598_2024_53257_MOESM1_ESM.zip › supplementary files/Drug sensitivity of low and high risk group/low risk group better/drugSenstivity.AZD6482.pdf]

Risk 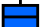 low 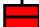 high

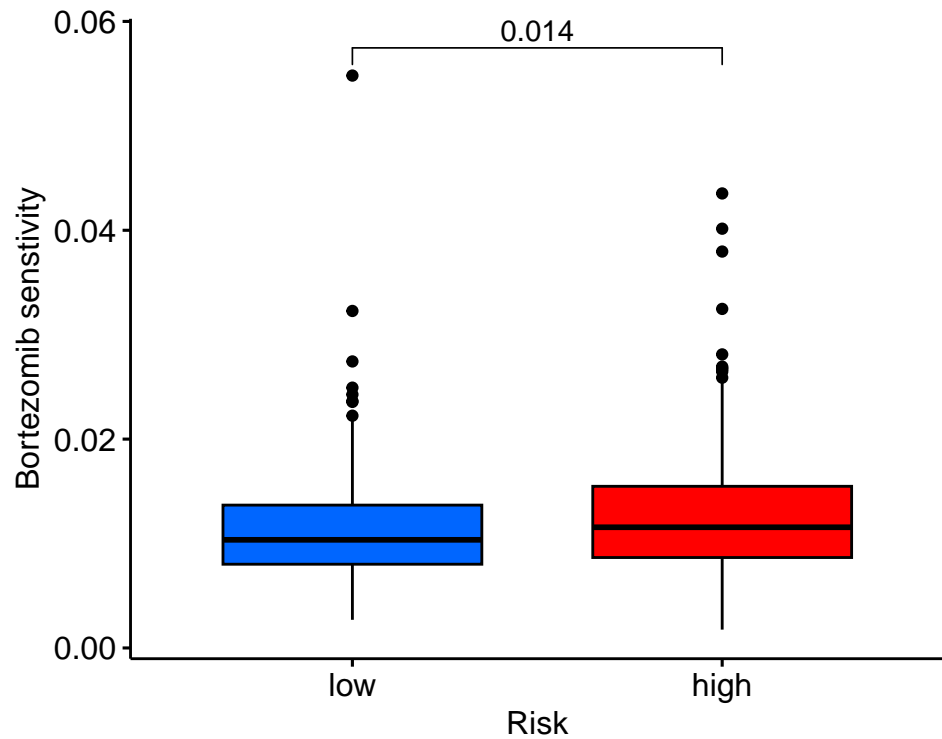

Supplement: Supplementary file 1 — Supplementary Information. [file 41598_2024_53257_MOESM1_ESM.zip › supplementary files/Drug sensitivity of low and high risk group/low risk group better/drugSenstivity.Bortezomib.pdf]

Risk 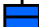 low 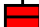 high

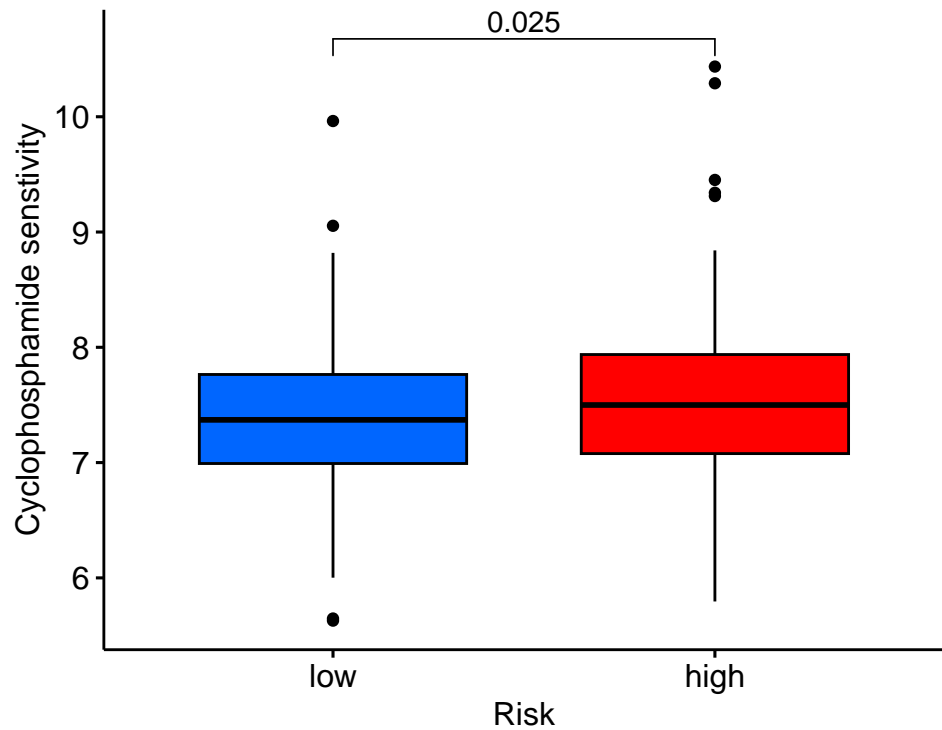

Supplement: Supplementary file 1 — Supplementary Information. [file 41598_2024_53257_MOESM1_ESM.zip › supplementary files/Drug sensitivity of low and high risk group/low risk group better/drugSenstivity.Cyclophosphamide.pdf]

Risk 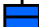 low 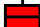 high

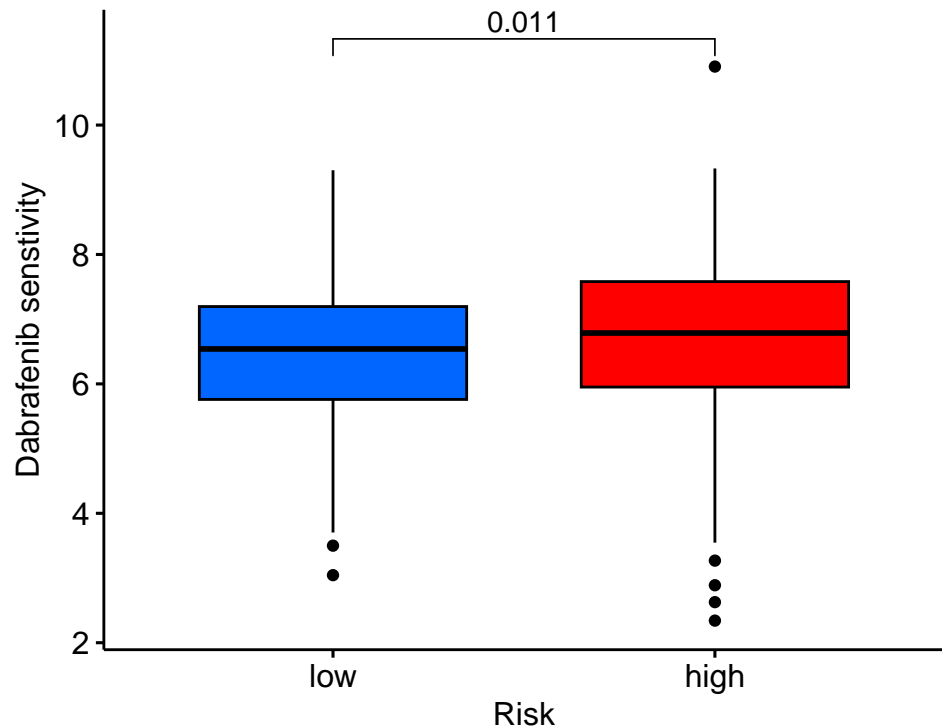

Supplement: Supplementary file 1 — Supplementary Information. [file 41598_2024_53257_MOESM1_ESM.zip › supplementary files/Drug sensitivity of low and high risk group/low risk group better/drugSenstivity.Dabrafenib.pdf]

Risk 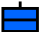 low 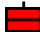 high

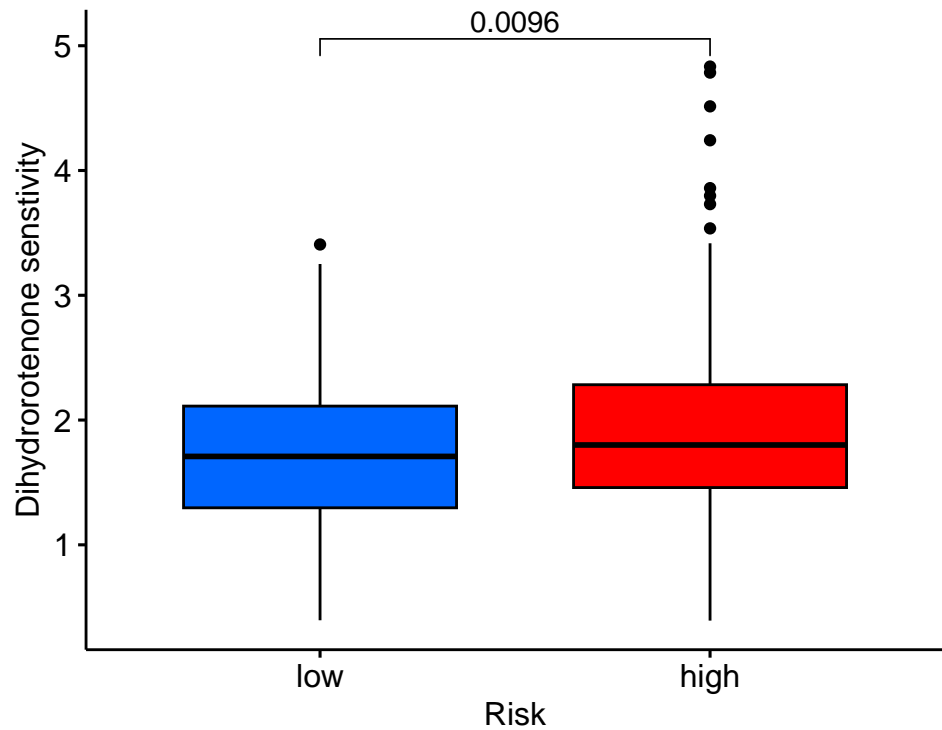

Supplement: Supplementary file 1 — Supplementary Information. [file 41598_2024_53257_MOESM1_ESM.zip › supplementary files/Drug sensitivity of low and high risk group/low risk group better/drugSenstivity.Dihydrorotenone.pdf]

Risk 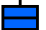 low 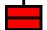 high

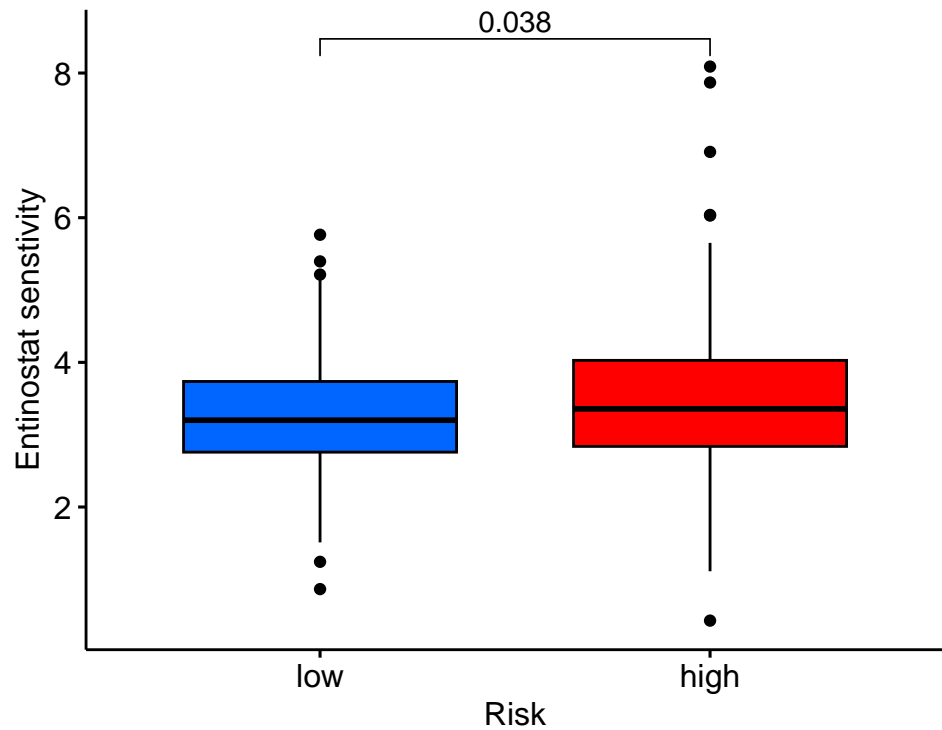

Supplement: Supplementary file 1 — Supplementary Information. [file 41598_2024_53257_MOESM1_ESM.zip › supplementary files/Drug sensitivity of low and high risk group/low risk group better/drugSenstivity.Entinostat.pdf]

Risk 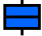 low 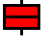 high

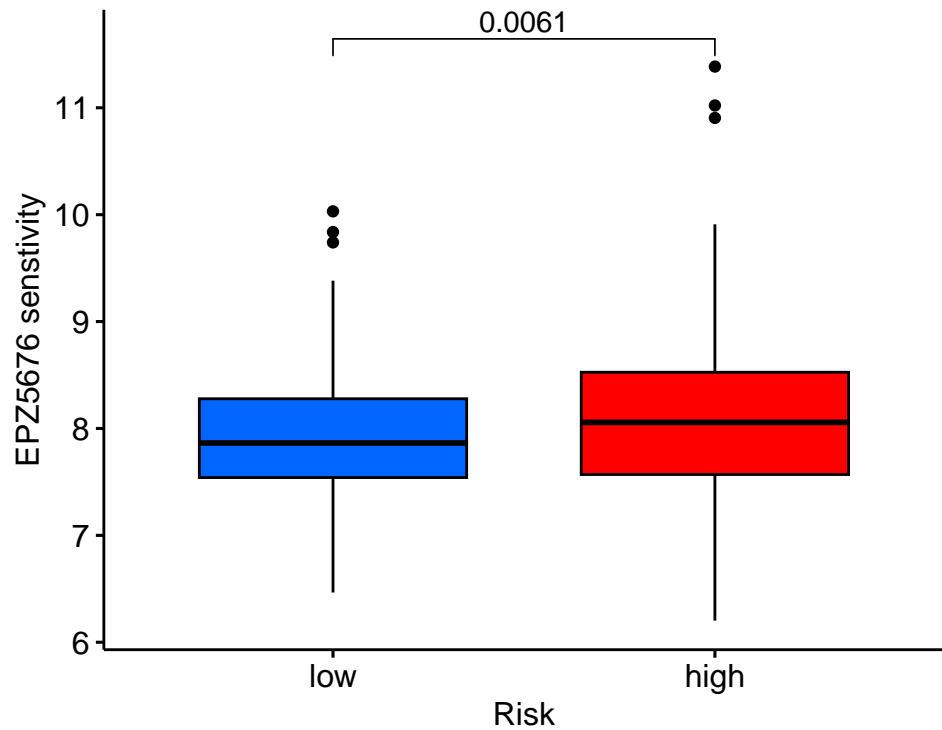

Supplement: Supplementary file 1 — Supplementary Information. [file 41598_2024_53257_MOESM1_ESM.zip › supplementary files/Drug sensitivity of low and high risk group/low risk group better/drugSenstivity.EPZ5676.pdf]

Risk 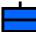 low 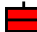 high

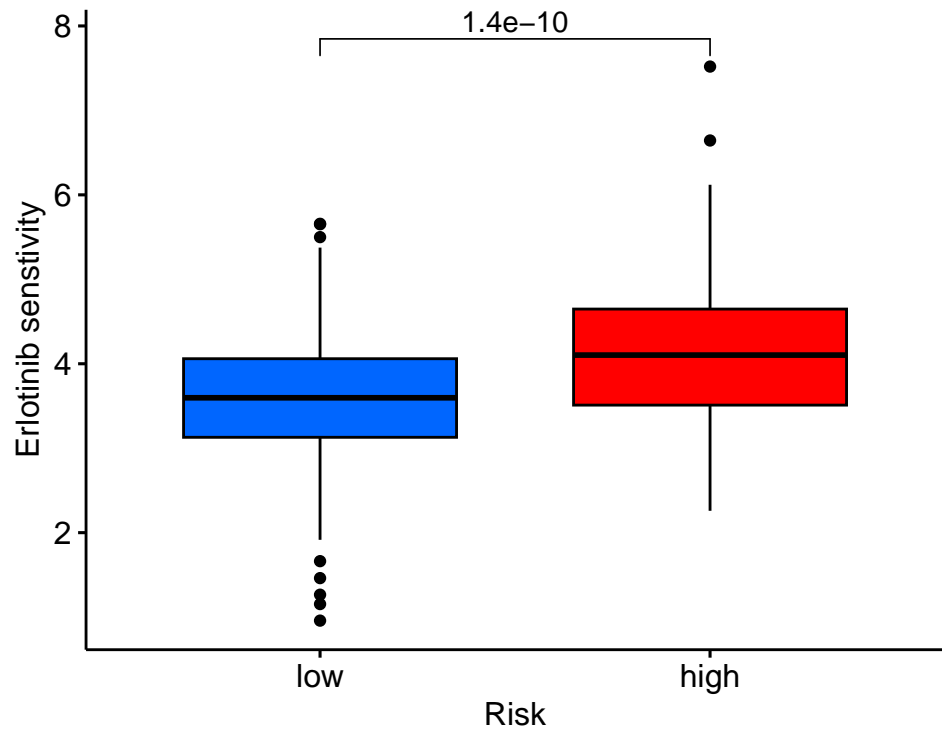

Supplement: Supplementary file 1 — Supplementary Information. [file 41598_2024_53257_MOESM1_ESM.zip › supplementary files/Drug sensitivity of low and high risk group/low risk group better/drugSenstivity.Erlotinib.pdf]

Risk 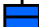 low 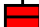 high

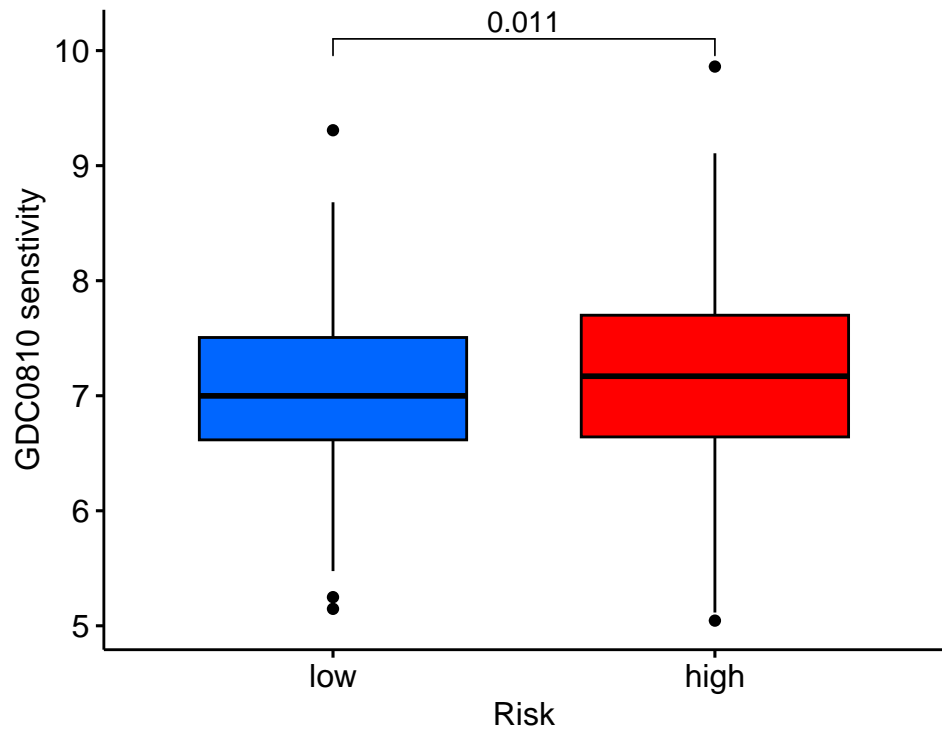

Supplement: Supplementary file 1 — Supplementary Information. [file 41598_2024_53257_MOESM1_ESM.zip › supplementary files/Drug sensitivity of low and high risk group/low risk group better/drugSenstivity.GDC0810.pdf]

Risk 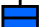 low 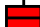 high

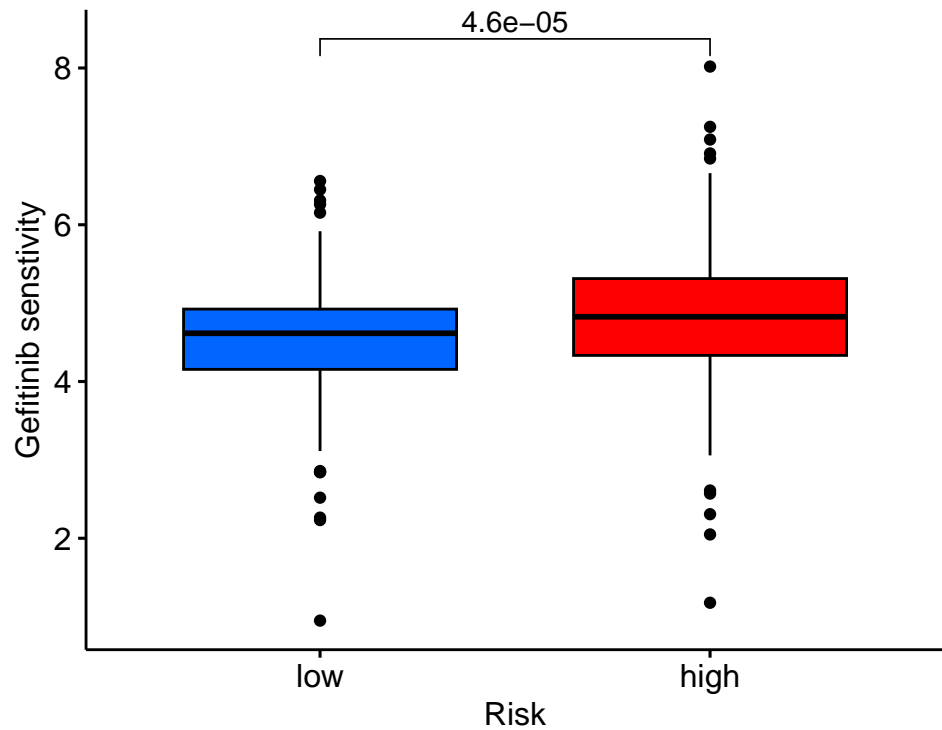

Supplement: Supplementary file 1 — Supplementary Information. [file 41598_2024_53257_MOESM1_ESM.zip › supplementary files/Drug sensitivity of low and high risk group/low risk group better/drugSenstivity.Gefitinib.pdf]

Risk 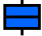 low 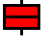 high

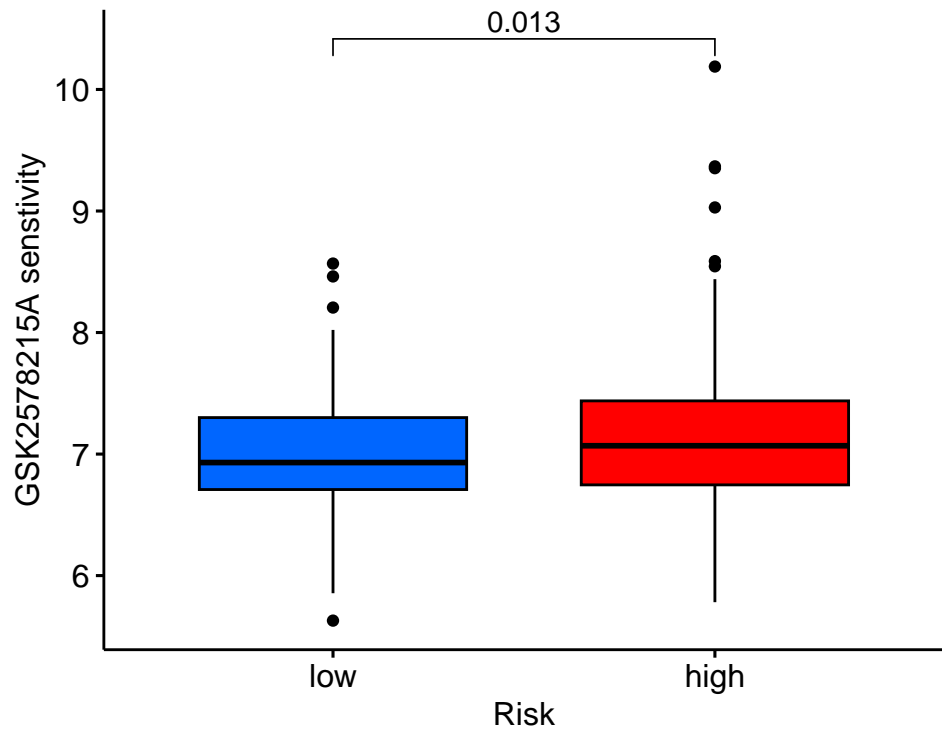

Supplement: Supplementary file 1 — Supplementary Information. [file 41598_2024_53257_MOESM1_ESM.zip › supplementary files/Drug sensitivity of low and high risk group/low risk group better/drugSenstivity.GSK2578215A.pdf]

Risk 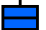 low 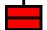 high

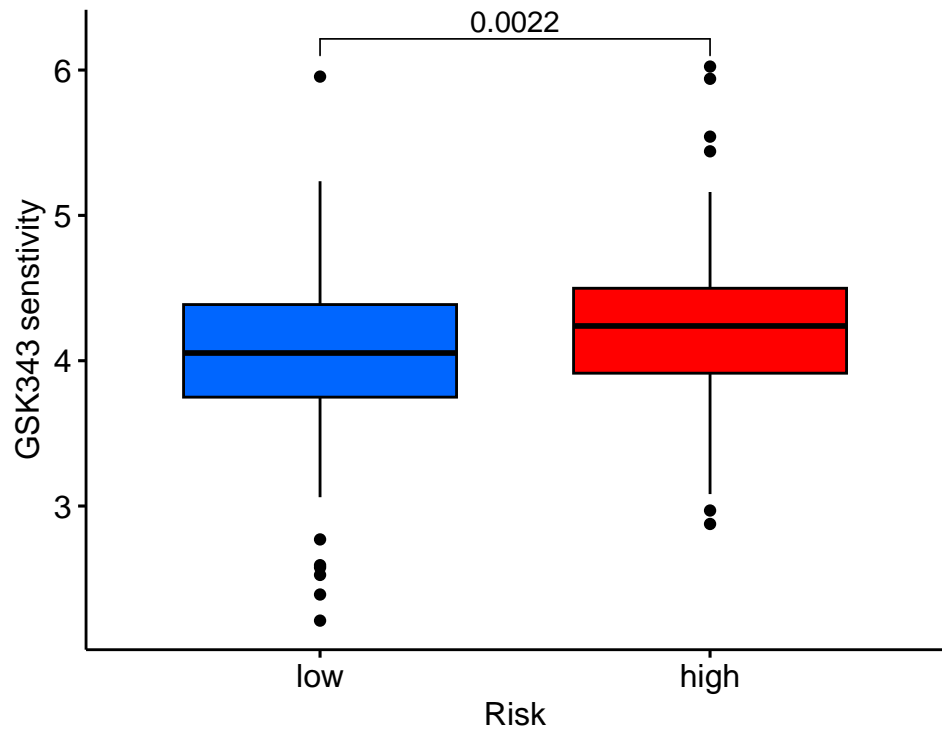

Supplement: Supplementary file 1 — Supplementary Information. [file 41598_2024_53257_MOESM1_ESM.zip › supplementary files/Drug sensitivity of low and high risk group/low risk group better/drugSenstivity.GSK343.pdf]

Risk 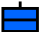 low 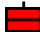 high

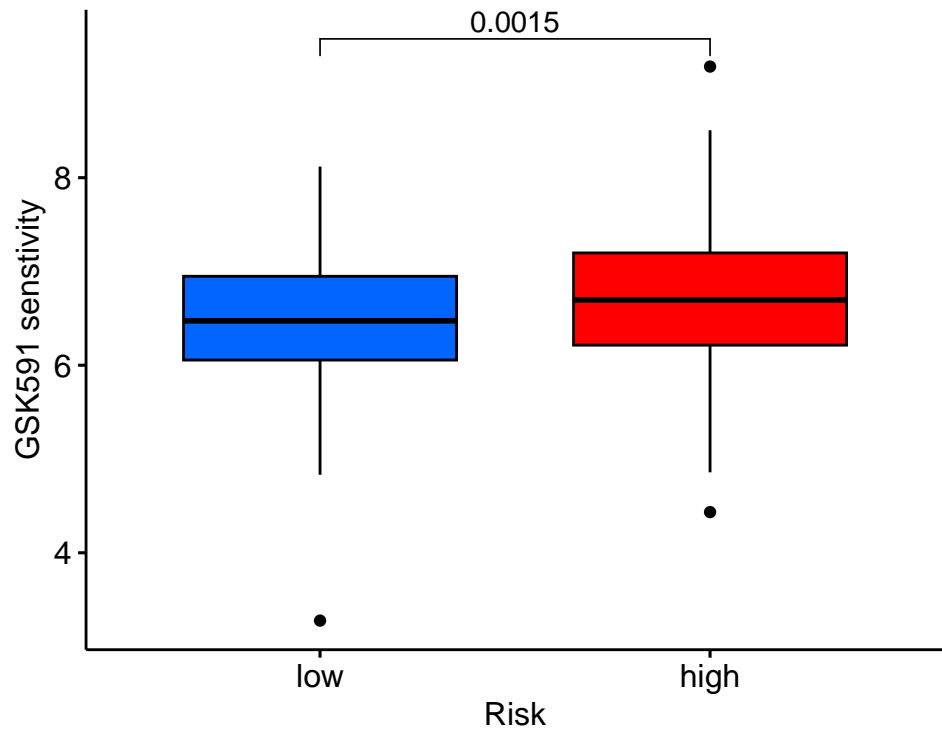

Supplement: Supplementary file 1 — Supplementary Information. [file 41598_2024_53257_MOESM1_ESM.zip › supplementary files/Drug sensitivity of low and high risk group/low risk group better/drugSenstivity.GSK591.pdf]

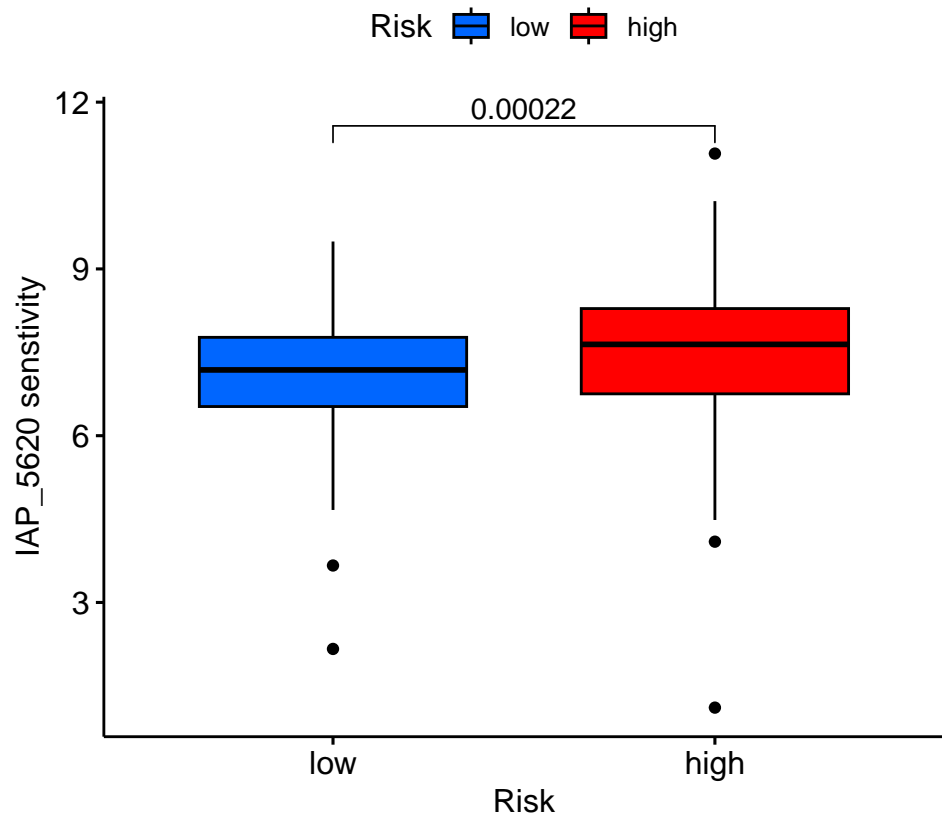

Supplement: Supplementary file 1 — Supplementary Information. [file 41598_2024_53257_MOESM1_ESM.zip › supplementary files/Drug sensitivity of low and high risk group/low risk group better/drugSenstivity.IAP_5620.pdf]

Risk 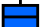 low 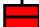 high

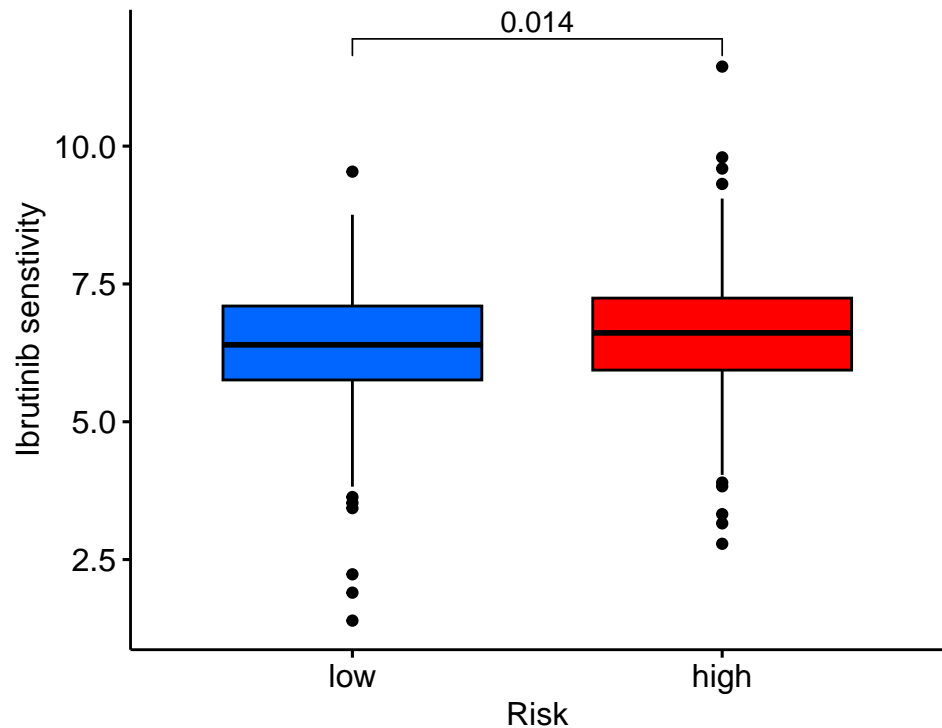

Supplement: Supplementary file 1 — Supplementary Information. [file 41598_2024_53257_MOESM1_ESM.zip › supplementary files/Drug sensitivity of low and high risk group/low risk group better/drugSenstivity.Ibrutinib.pdf]

Risk 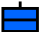 low 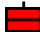 high

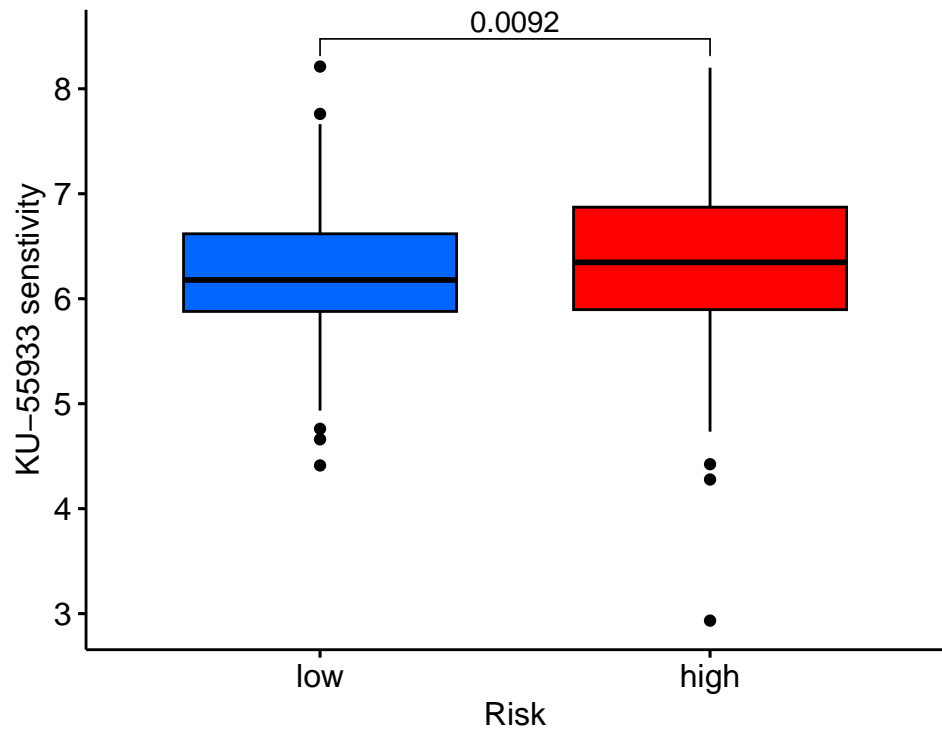

Supplement: Supplementary file 1 — Supplementary Information. [file 41598_2024_53257_MOESM1_ESM.zip › supplementary files/Drug sensitivity of low and high risk group/low risk group better/drugSenstivity.KU-55933.pdf]

Risk 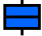 low 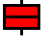 high

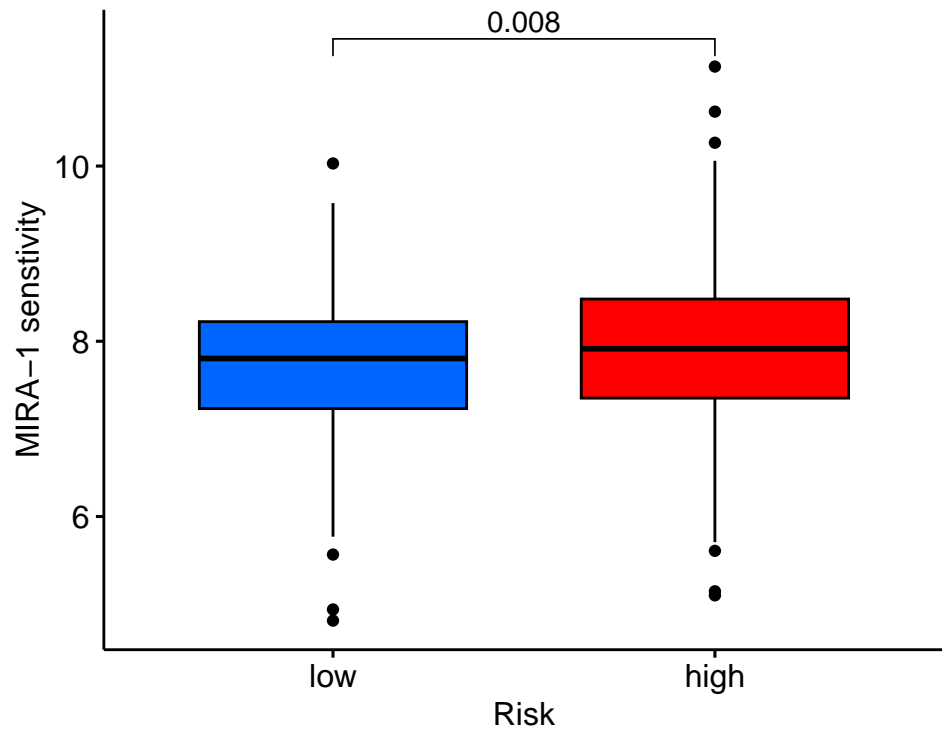

Supplement: Supplementary file 1 — Supplementary Information. [file 41598_2024_53257_MOESM1_ESM.zip › supplementary files/Drug sensitivity of low and high risk group/low risk group better/drugSenstivity.MIRA-1.pdf]

Risk 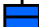 low 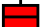 high

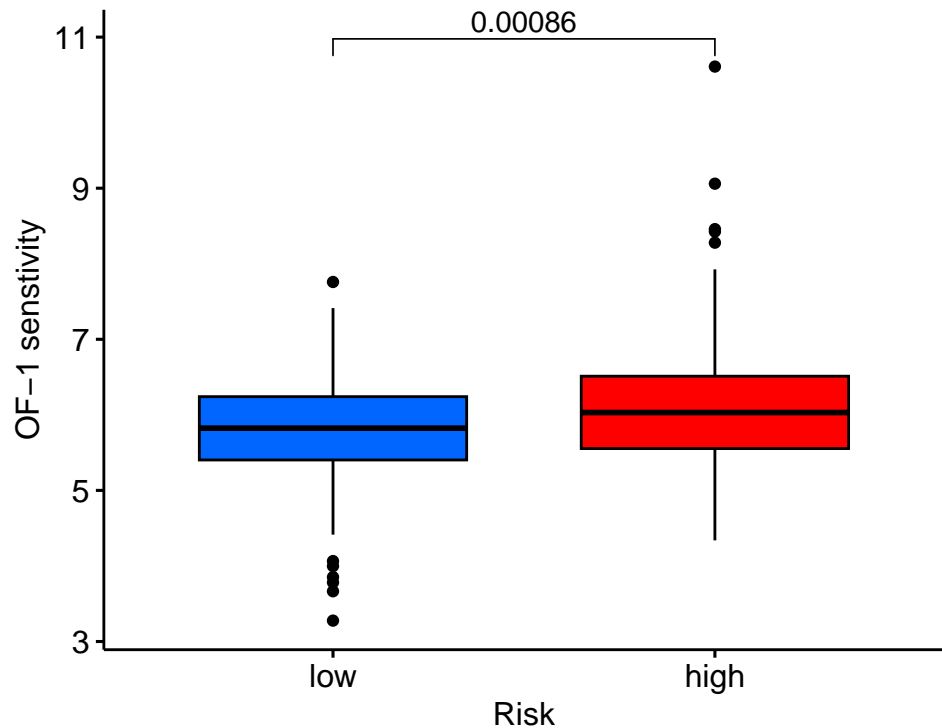

Supplement: Supplementary file 1 — Supplementary Information. [file 41598_2024_53257_MOESM1_ESM.zip › supplementary files/Drug sensitivity of low and high risk group/low risk group better/drugSenstivity.OF-1.pdf]

Risk 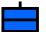 low 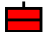 high

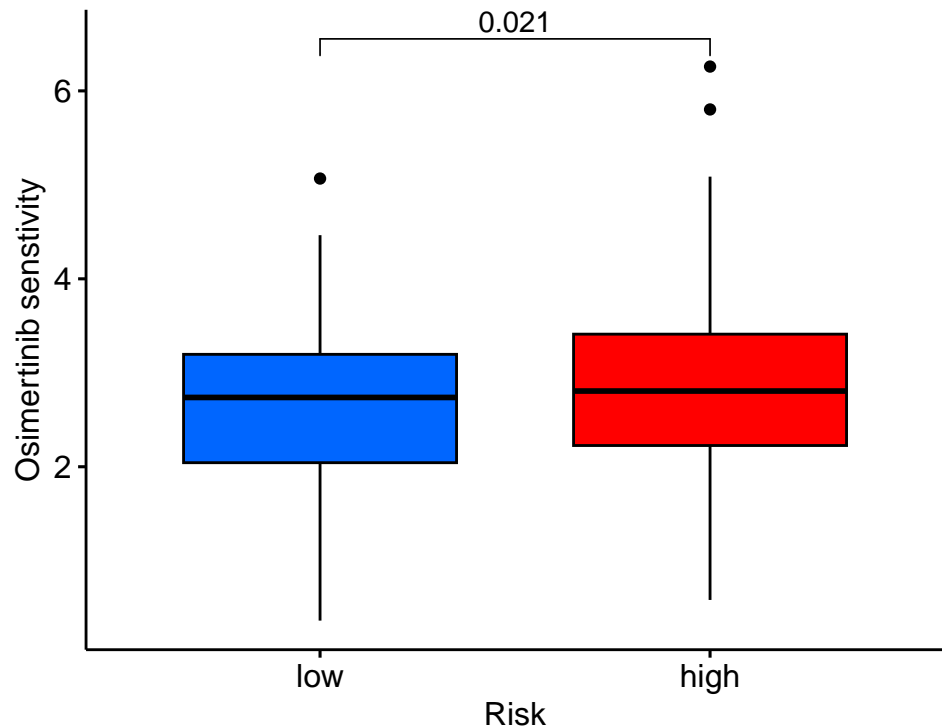

Supplement: Supplementary file 1 — Supplementary Information. [file 41598_2024_53257_MOESM1_ESM.zip › supplementary files/Drug sensitivity of low and high risk group/low risk group better/drugSenstivity.Osimertinib.pdf]

Risk 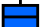 low 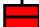 high

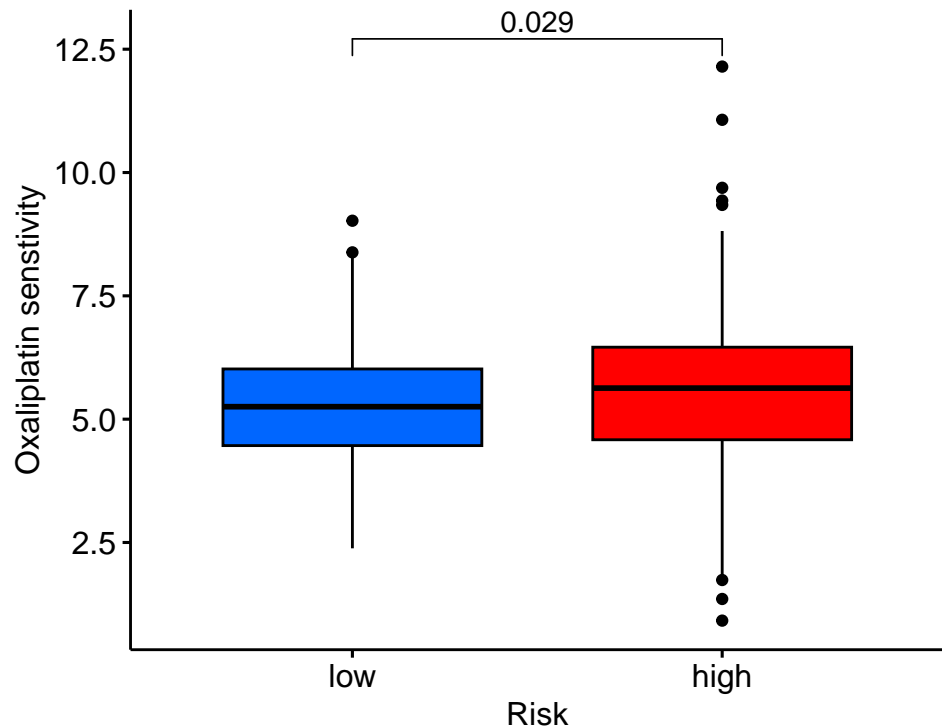

Supplement: Supplementary file 1 — Supplementary Information. [file 41598_2024_53257_MOESM1_ESM.zip › supplementary files/Drug sensitivity of low and high risk group/low risk group better/drugSenstivity.Oxaliplatin.pdf]

Risk 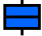 low 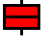 high

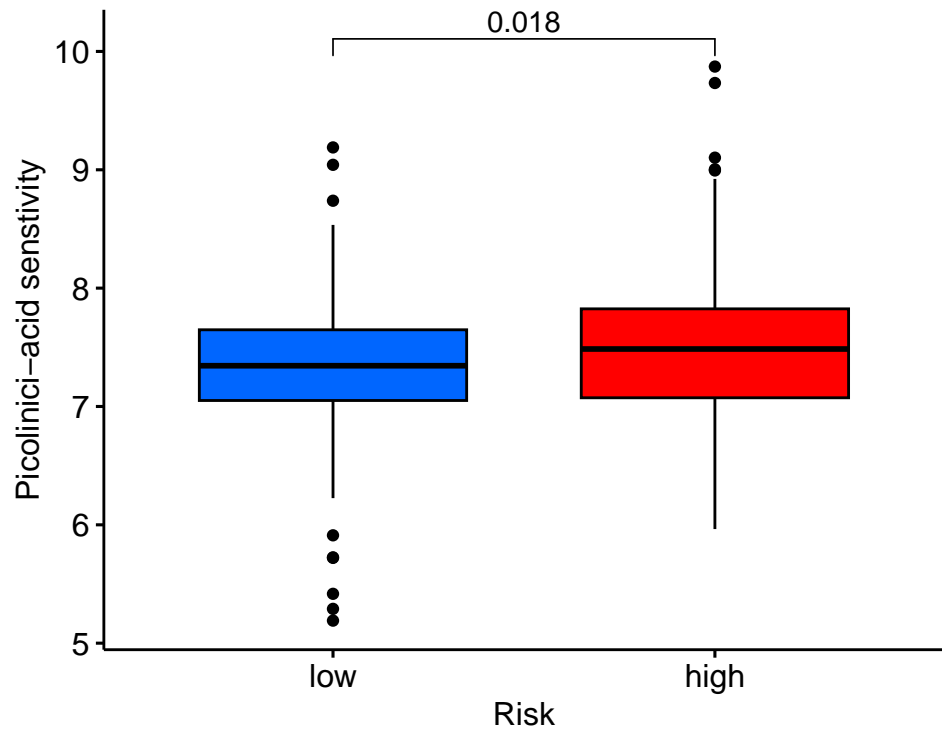

Supplement: Supplementary file 1 — Supplementary Information. [file 41598_2024_53257_MOESM1_ESM.zip › supplementary files/Drug sensitivity of low and high risk group/low risk group better/drugSenstivity.Picolinici-acid.pdf]

Risk 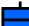 low 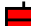 high

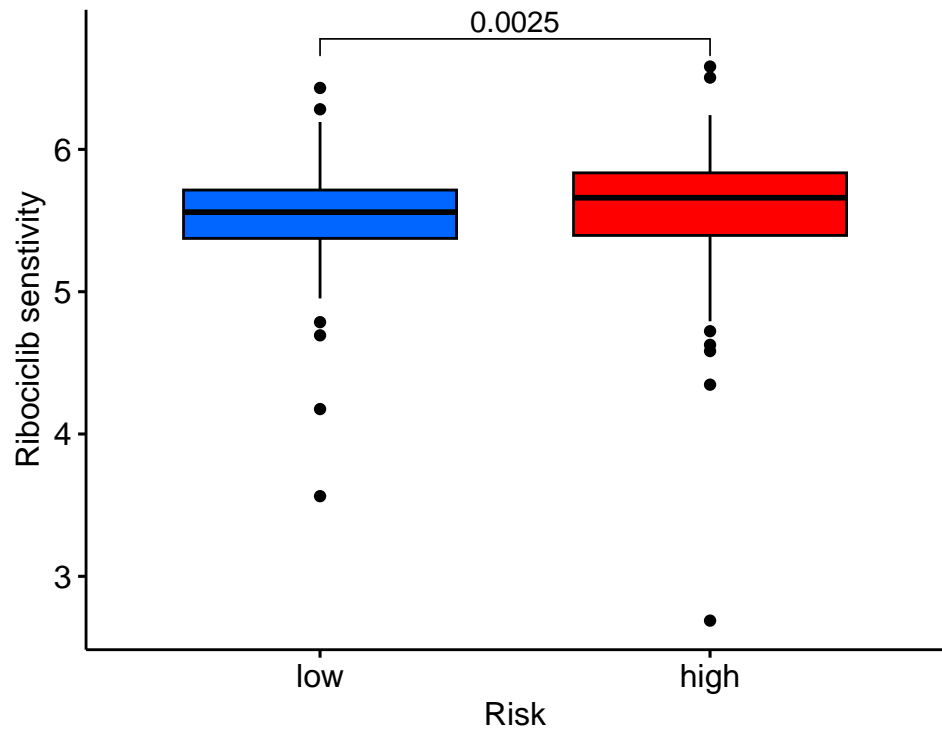

Supplement: Supplementary file 1 — Supplementary Information. [file 41598_2024_53257_MOESM1_ESM.zip › supplementary files/Drug sensitivity of low and high risk group/low risk group better/drugSenstivity.Ribociclib.pdf]

Risk 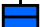 low 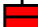 high

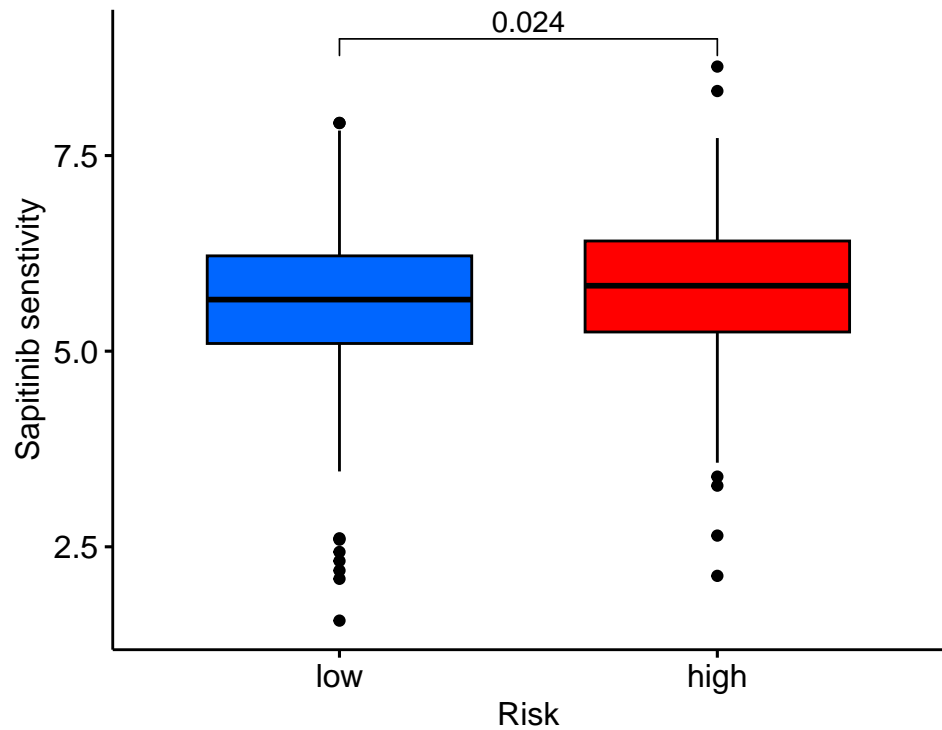

Supplement: Supplementary file 1 — Supplementary Information. [file 41598_2024_53257_MOESM1_ESM.zip › supplementary files/Drug sensitivity of low and high risk group/low risk group better/drugSenstivity.Sapitinib.pdf]

Risk low high

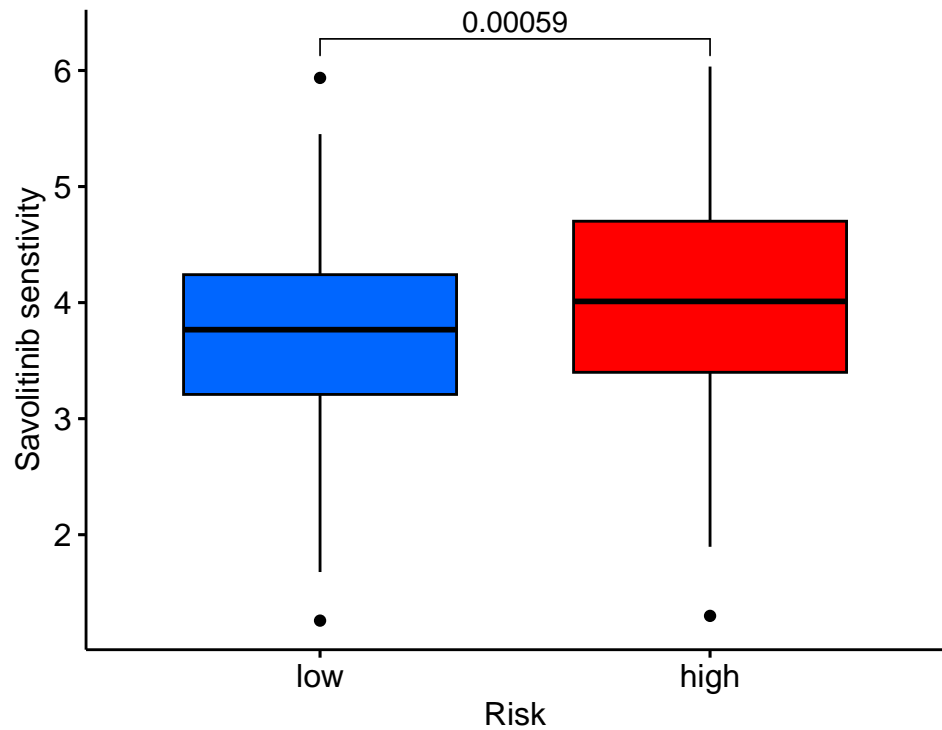

Supplement: Supplementary file 1 — Supplementary Information. [file 41598_2024_53257_MOESM1_ESM.zip › supplementary files/Drug sensitivity of low and high risk group/low risk group better/drugSenstivity.Savolitinib.pdf]

Risk 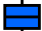 low 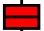 high

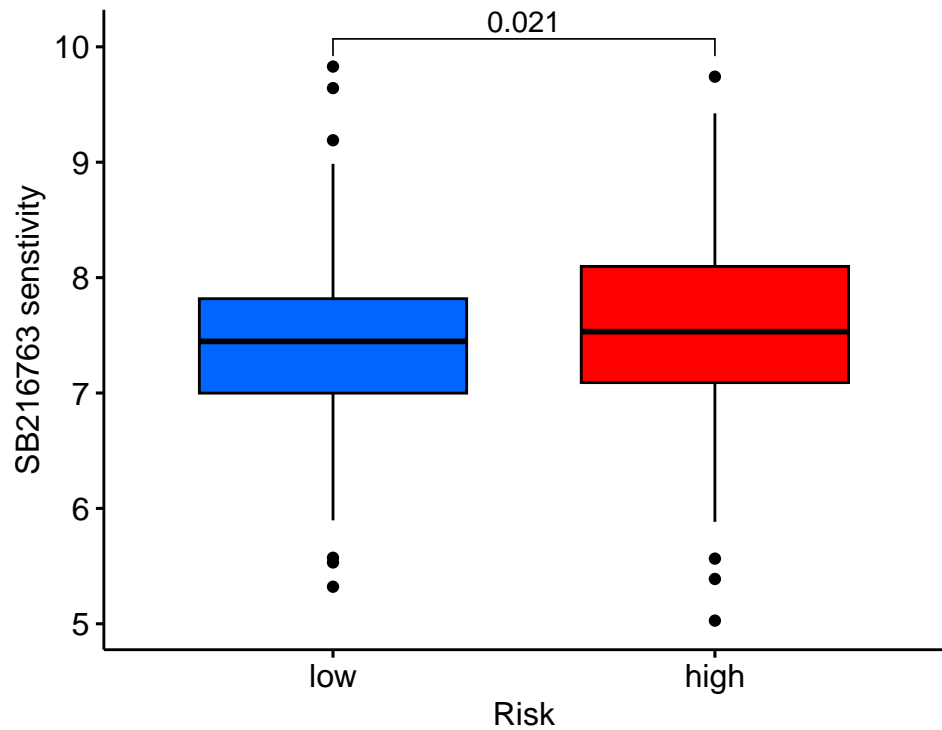

Supplement: Supplementary file 1 — Supplementary Information. [file 41598_2024_53257_MOESM1_ESM.zip › supplementary files/Drug sensitivity of low and high risk group/low risk group better/drugSenstivity.SB216763.pdf]

Risk 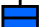 low 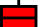 high

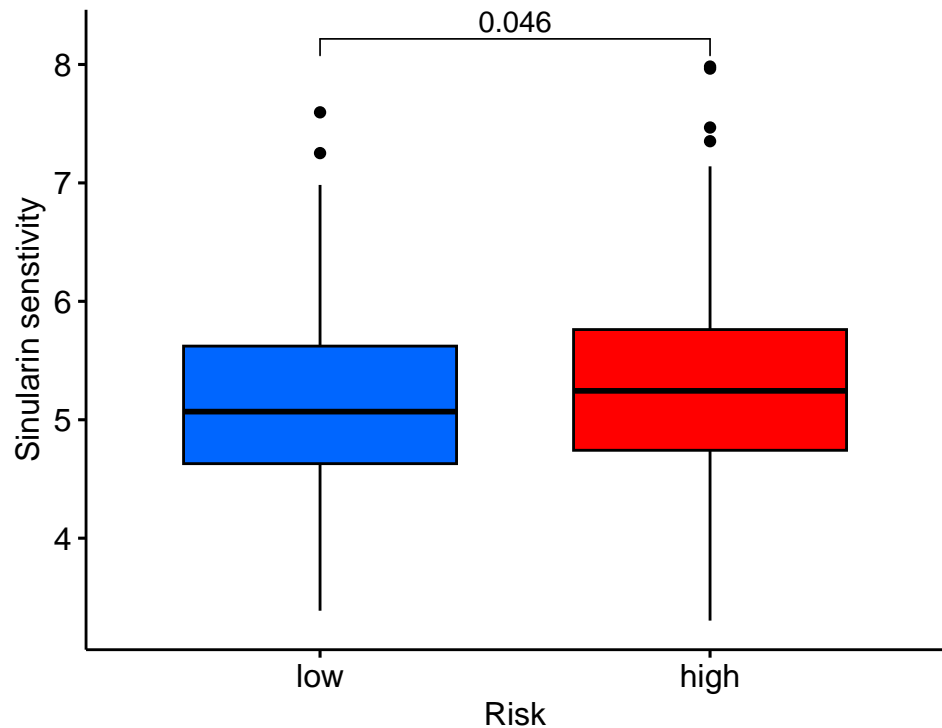

Supplement: Supplementary file 1 — Supplementary Information. [file 41598_2024_53257_MOESM1_ESM.zip › supplementary files/Drug sensitivity of low and high risk group/low risk group better/drugSenstivity.Sinularin.pdf]

Risk 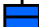 low 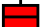 high

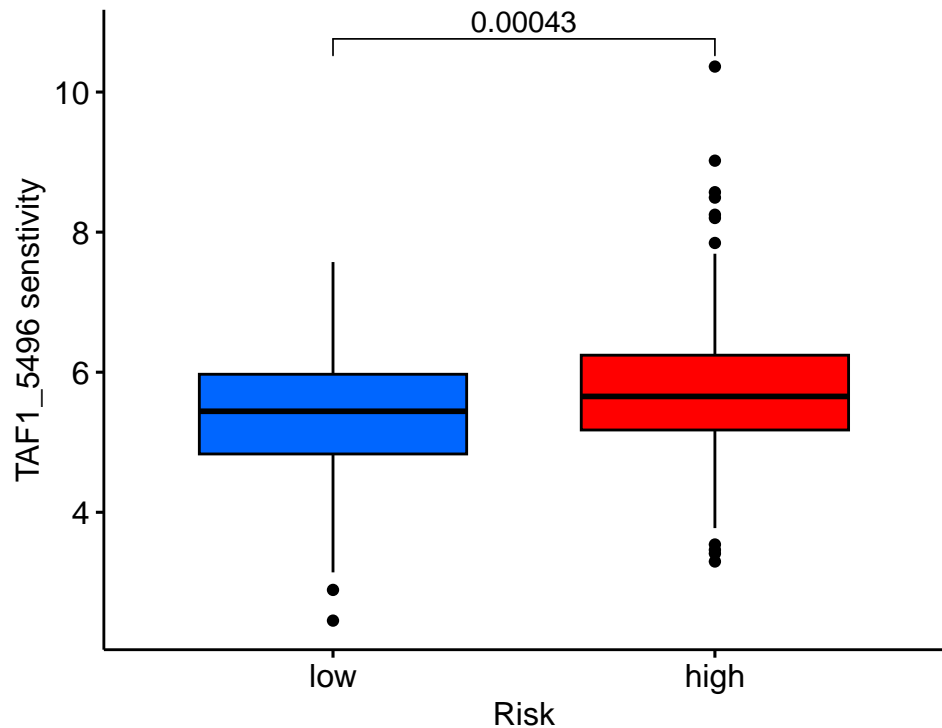

Supplement: Supplementary file 1 — Supplementary Information. [file 41598_2024_53257_MOESM1_ESM.zip › supplementary files/Drug sensitivity of low and high risk group/low risk group better/drugSenstivity.TAF1_5496.pdf]

Risk 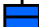 low 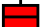 high

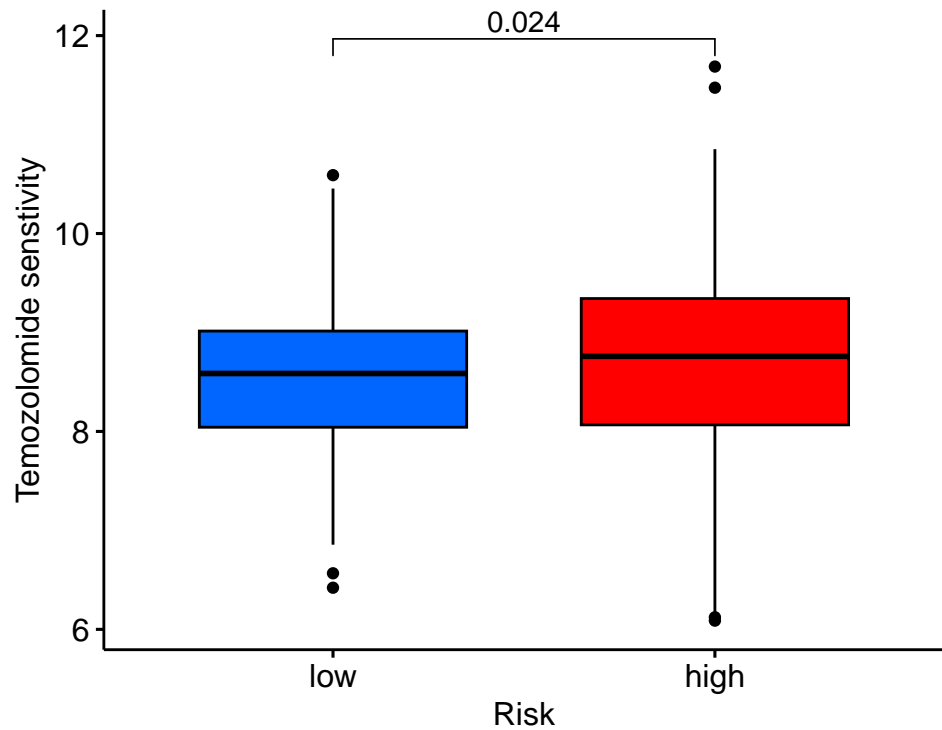

Supplement: Supplementary file 1 — Supplementary Information. [file 41598_2024_53257_MOESM1_ESM.zip › supplementary files/Drug sensitivity of low and high risk group/low risk group better/drugSenstivity.Temozolomide.pdf]

Risk 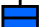 low 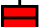 high

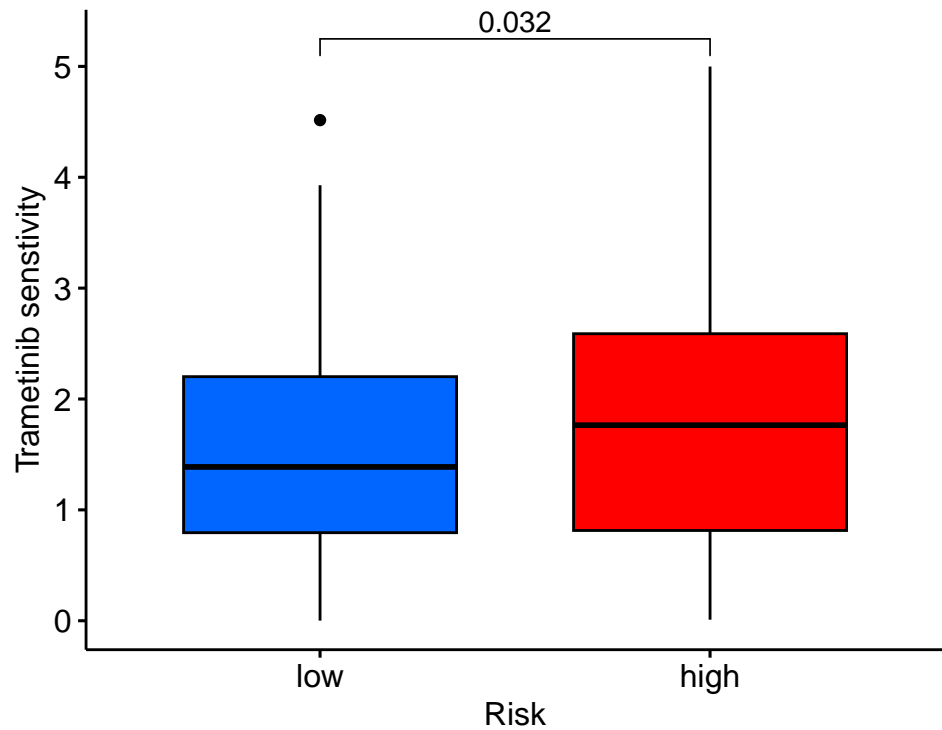

Supplement: Supplementary file 1 — Supplementary Information. [file 41598_2024_53257_MOESM1_ESM.zip › supplementary files/Drug sensitivity of low and high risk group/low risk group better/drugSenstivity.Trametinib.pdf]

Risk 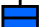 low 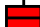 high

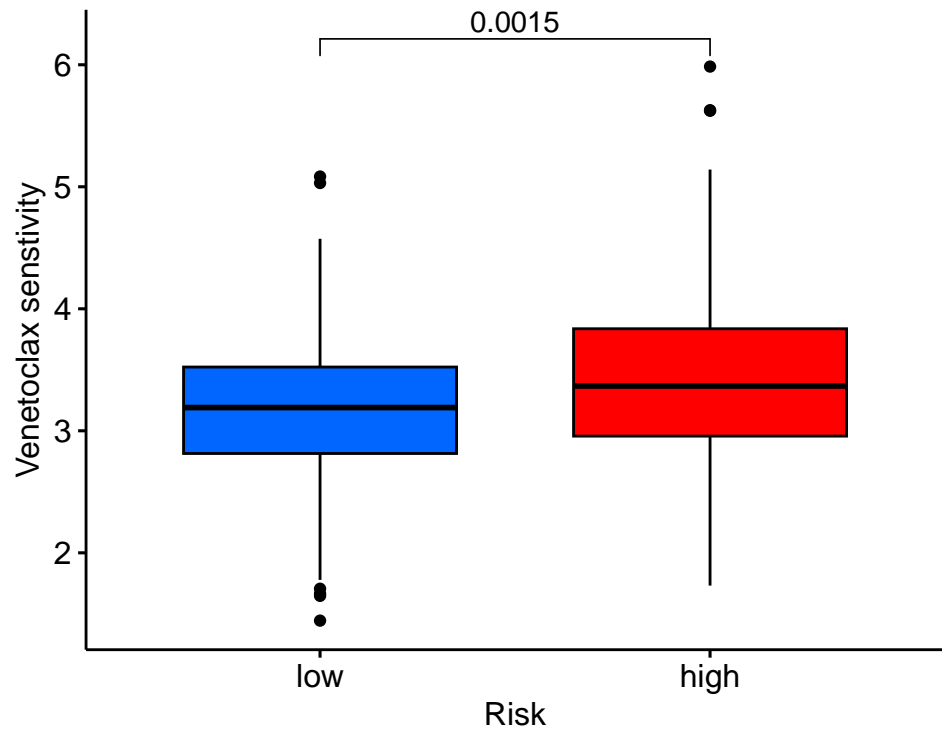

Supplement: Supplementary file 1 — Supplementary Information. [file 41598_2024_53257_MOESM1_ESM.zip › supplementary files/Drug sensitivity of low and high risk group/low risk group better/drugSenstivity.Venetoclax.pdf]
