# Supplementary figures and images for: Construction and validation of a colon cancer prognostic model based on tumor mutation burden-related genes (part 1 of 2)
Source: Sci Rep. 2024 Feb 4;14:2867. doi: 10.1038/s41598-024-53257-z (PMC10838917; doi:10.1038/s41598-024-53257-z)

Risk C1 C2

0.00022

Acetalax sensitivity

12

10

8

6

4

C1

C2

Risk

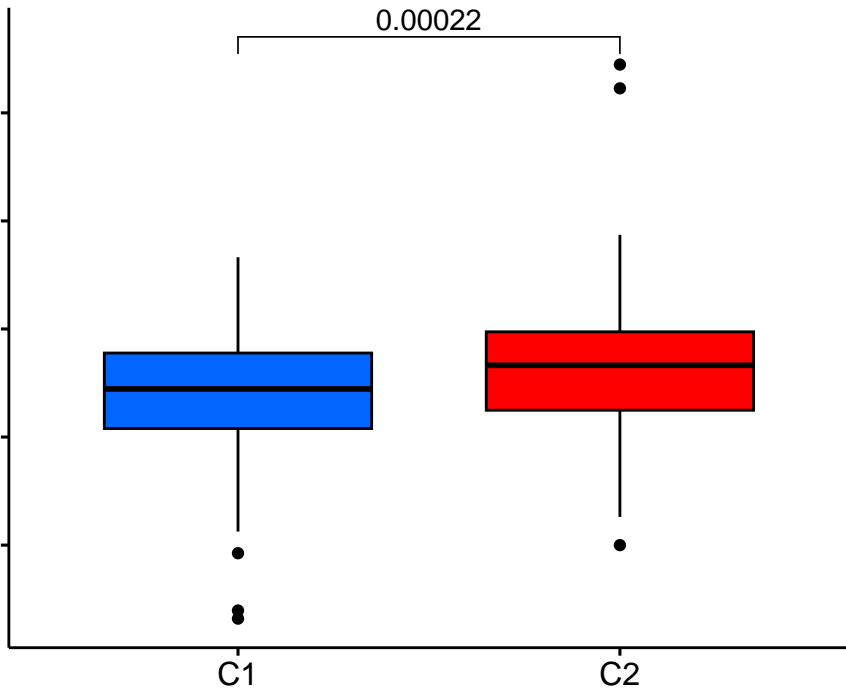

Supplement: Supplementary file 1 — Supplementary Information. [file 41598_2024_53257_MOESM1_ESM.zip › supplementary files/Drug sensitivity of C1 group and C2 group/C1 better/drugSenstivity.Acetalax.pdf]

Risk C1 C2

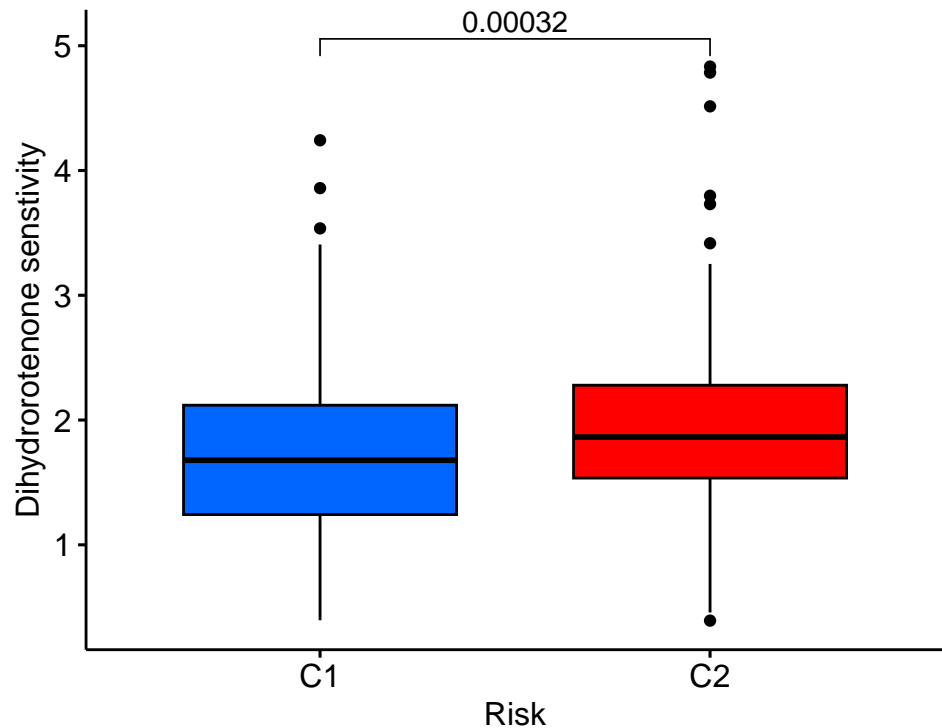

Supplement: Supplementary file 1 — Supplementary Information. [file 41598_2024_53257_MOESM1_ESM.zip › supplementary files/Drug sensitivity of C1 group and C2 group/C1 better/drugSenstivity.Dihydrorotenone.pdf]

Risk C1 C2

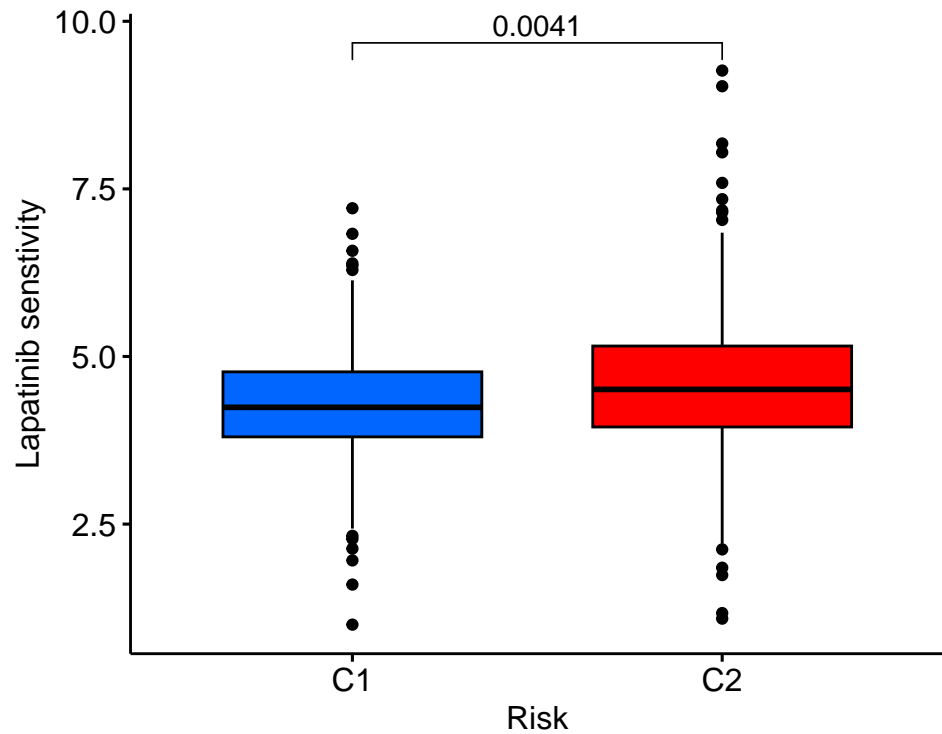

Supplement: Supplementary file 1 — Supplementary Information. [file 41598_2024_53257_MOESM1_ESM.zip › supplementary files/Drug sensitivity of C1 group and C2 group/C1 better/drugSenstivity.Lapatinib.pdf]

Risk C1 C2

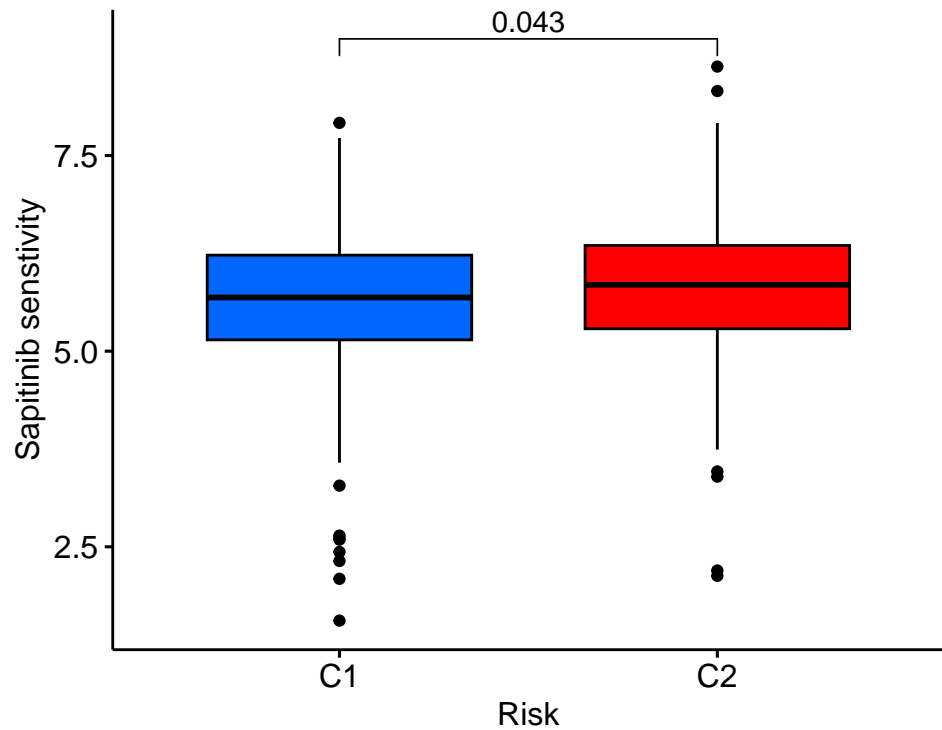

Supplement: Supplementary file 1 — Supplementary Information. [file 41598_2024_53257_MOESM1_ESM.zip › supplementary files/Drug sensitivity of C1 group and C2 group/C1 better/drugSenstivity.Sapitinib.pdf]

TAF1\_5496 sensitivity

Risk C1 C2

0.037

C1

C2

Risk

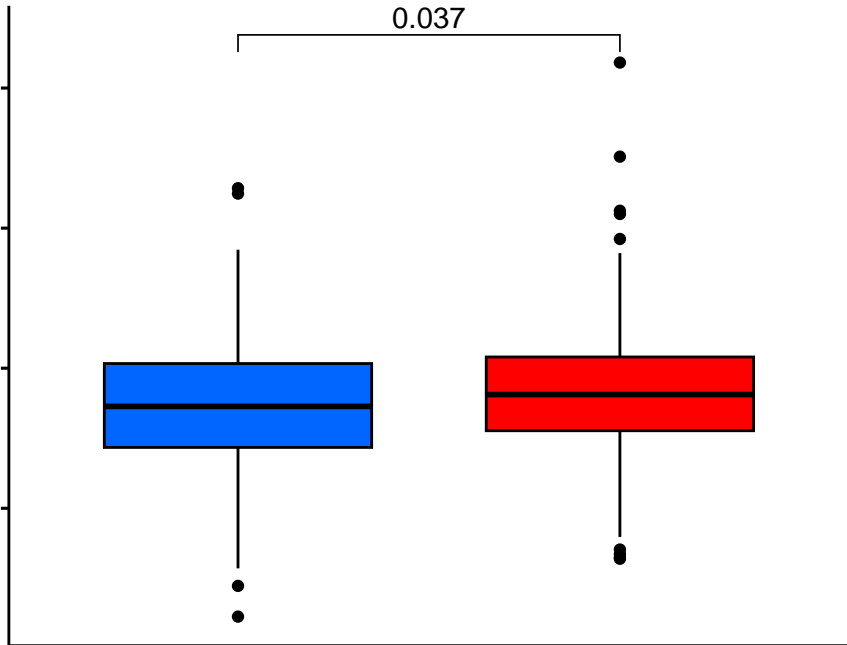

Supplement: Supplementary file 1 — Supplementary Information. [file 41598_2024_53257_MOESM1_ESM.zip › supplementary files/Drug sensitivity of C1 group and C2 group/C1 better/drugSenstivity.TAF1_5496.pdf]

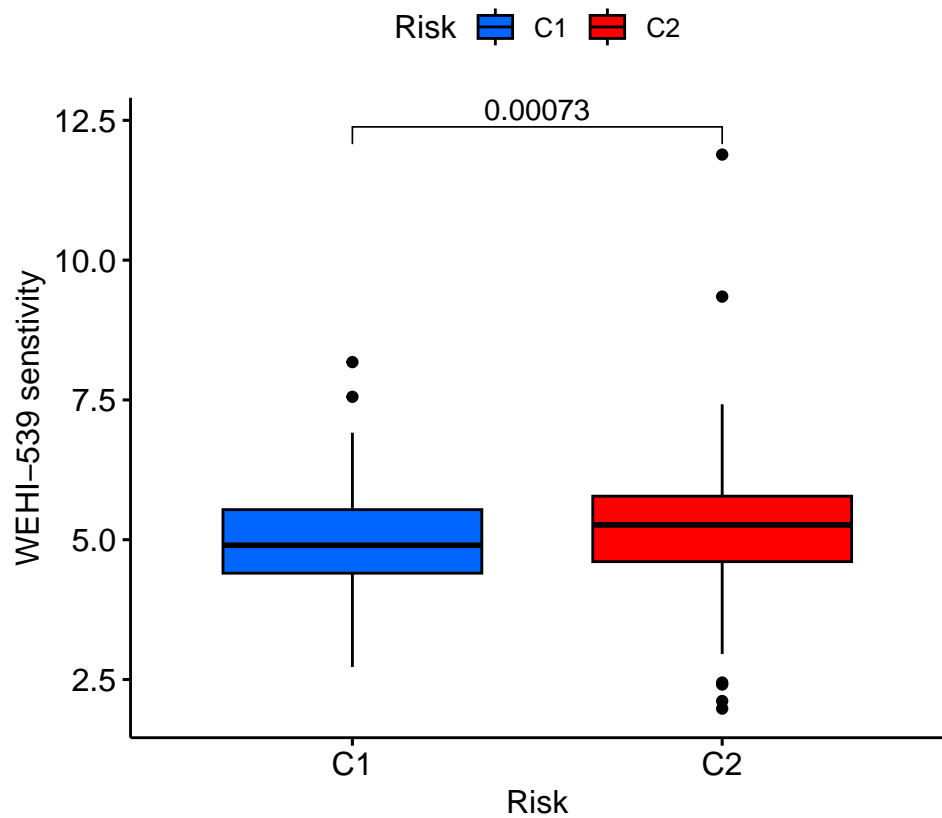

Supplement: Supplementary file 1 — Supplementary Information. [file 41598_2024_53257_MOESM1_ESM.zip › supplementary files/Drug sensitivity of C1 group and C2 group/C1 better/drugSenstivity.WEHI-539.pdf]

Risk C1 C2

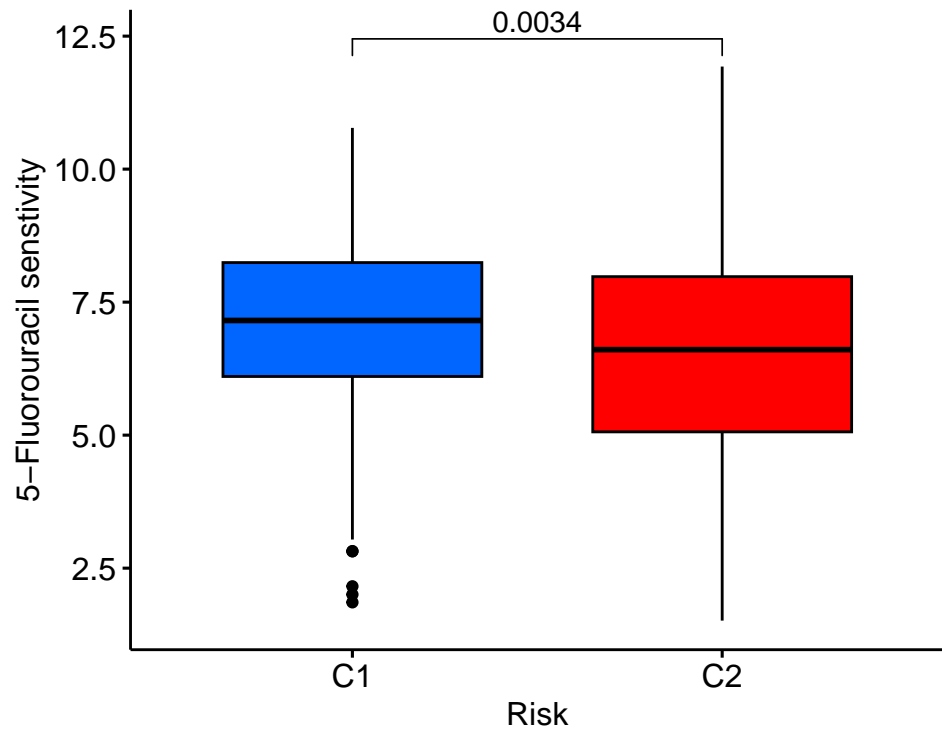

Supplement: Supplementary file 1 — Supplementary Information. [file 41598_2024_53257_MOESM1_ESM.zip › supplementary files/Drug sensitivity of C1 group and C2 group/C2 better/drugSenstivity.5-Fluorouracil.pdf]

Risk C1 C2

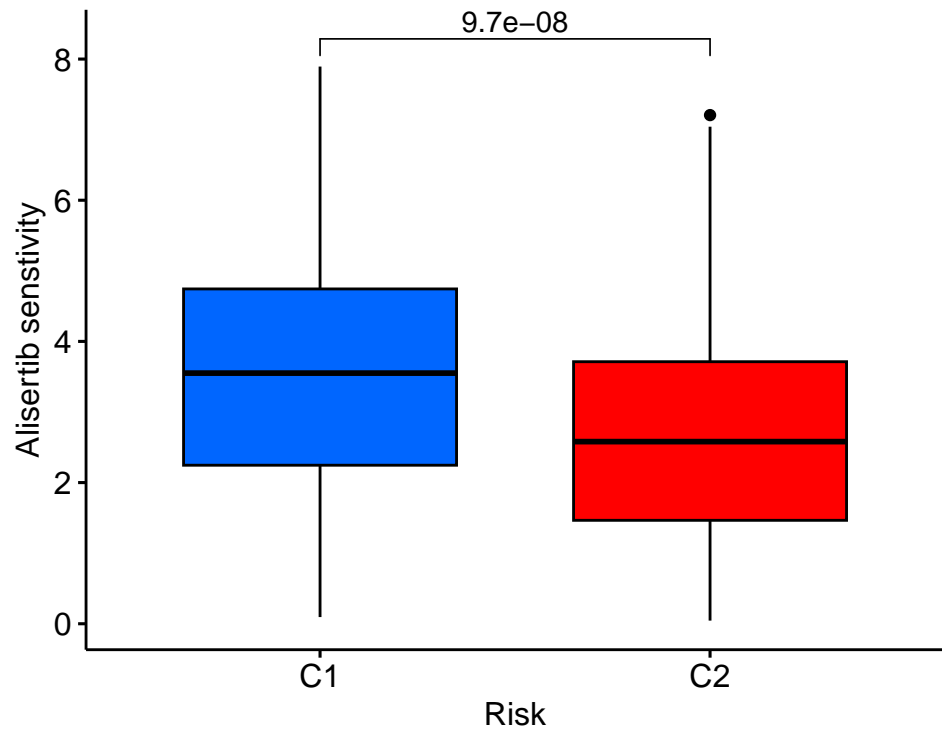

Supplement: Supplementary file 1 — Supplementary Information. [file 41598_2024_53257_MOESM1_ESM.zip › supplementary files/Drug sensitivity of C1 group and C2 group/C2 better/drugSenstivity.Alisertib.pdf]

Risk C1 C2

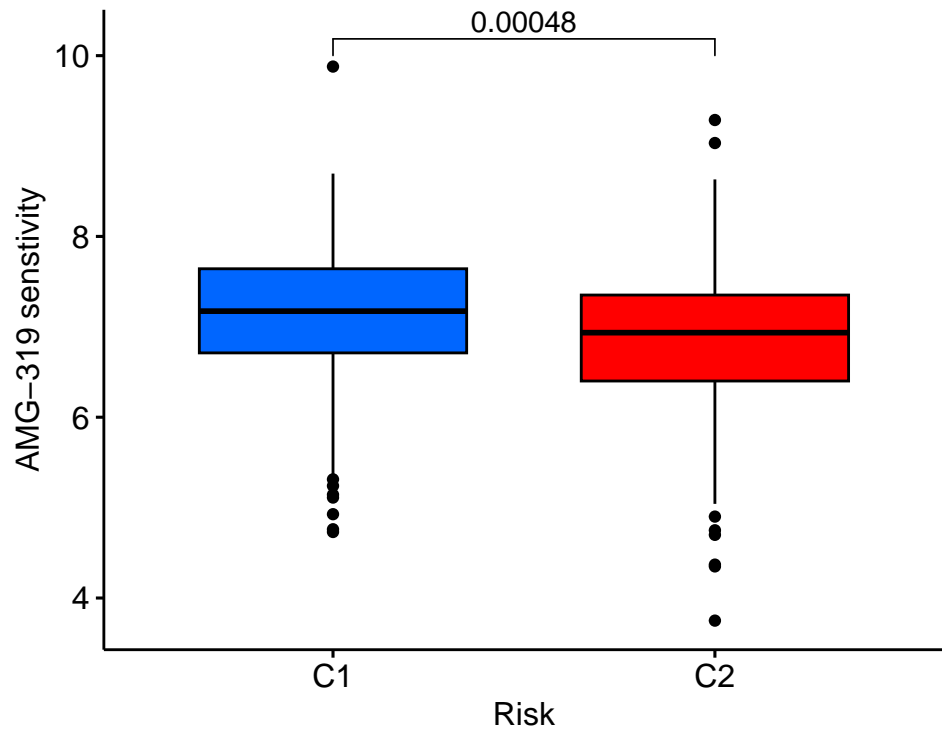

Supplement: Supplementary file 1 — Supplementary Information. [file 41598_2024_53257_MOESM1_ESM.zip › supplementary files/Drug sensitivity of C1 group and C2 group/C2 better/drugSenstivity.AMG-319.pdf]

Risk C1 C2

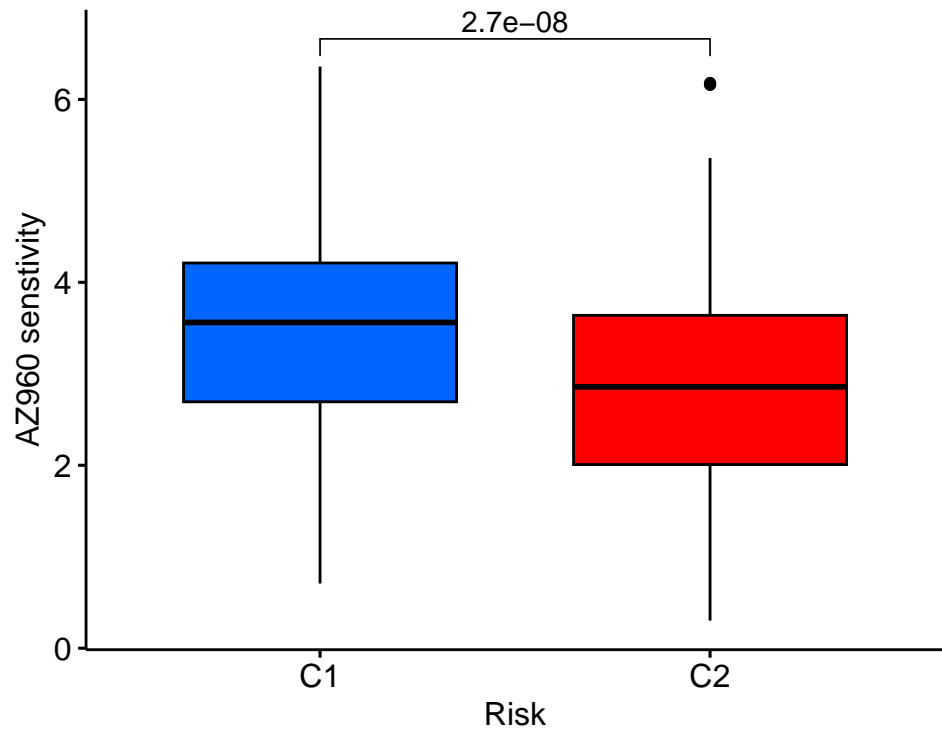

Supplement: Supplementary file 1 — Supplementary Information. [file 41598_2024_53257_MOESM1_ESM.zip › supplementary files/Drug sensitivity of C1 group and C2 group/C2 better/drugSenstivity.AZ960.pdf]

Risk C1 C2

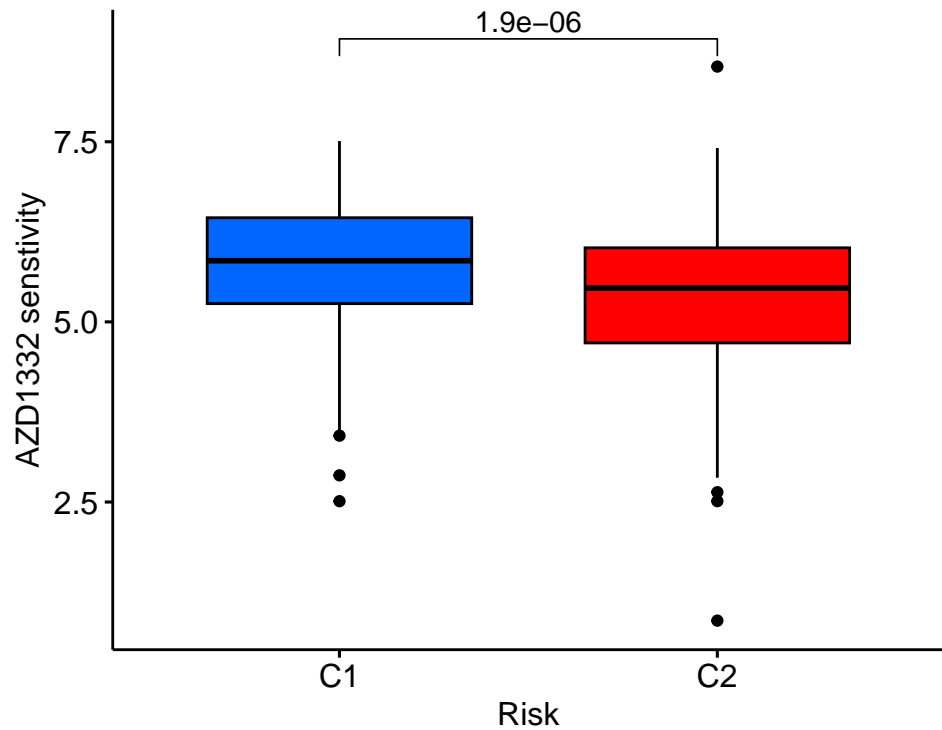

Supplement: Supplementary file 1 — Supplementary Information. [file 41598_2024_53257_MOESM1_ESM.zip › supplementary files/Drug sensitivity of C1 group and C2 group/C2 better/drugSenstivity.AZD1332.pdf]

Risk C1 C2

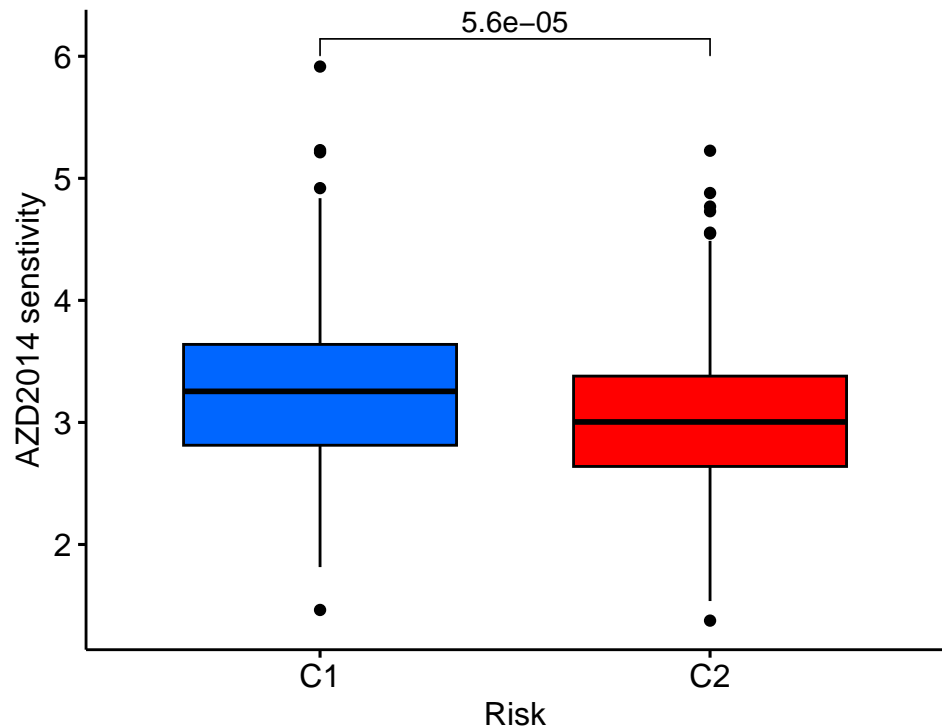

Supplement: Supplementary file 1 — Supplementary Information. [file 41598_2024_53257_MOESM1_ESM.zip › supplementary files/Drug sensitivity of C1 group and C2 group/C2 better/drugSenstivity.AZD2014.pdf]

Risk C1 C2

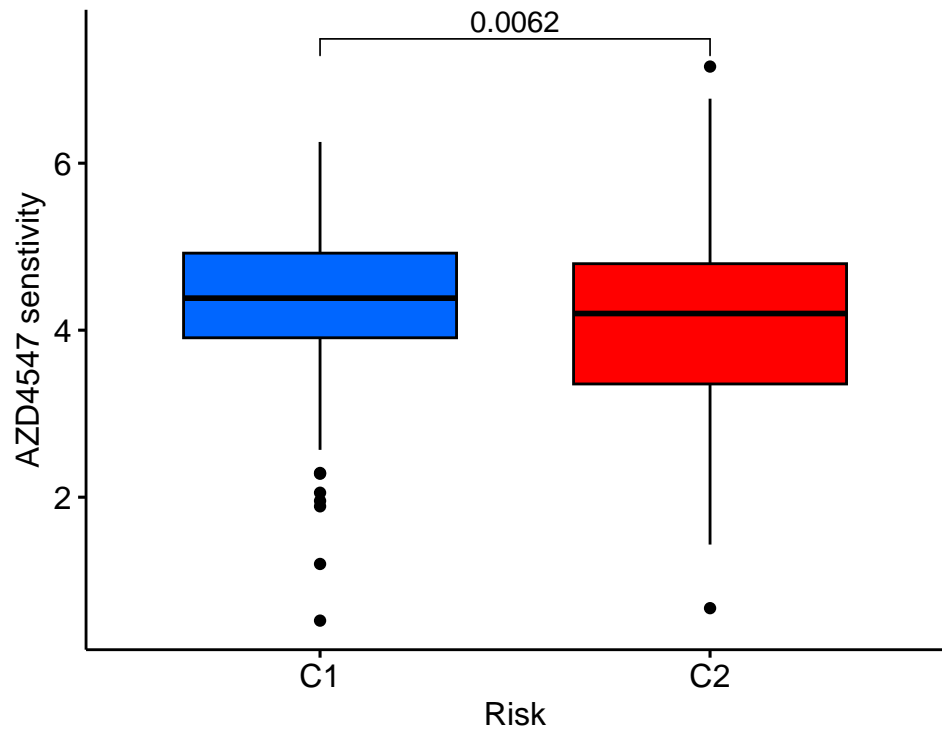

Supplement: Supplementary file 1 — Supplementary Information. [file 41598_2024_53257_MOESM1_ESM.zip › supplementary files/Drug sensitivity of C1 group and C2 group/C2 better/drugSenstivity.AZD4547.pdf]

Risk C1 C2

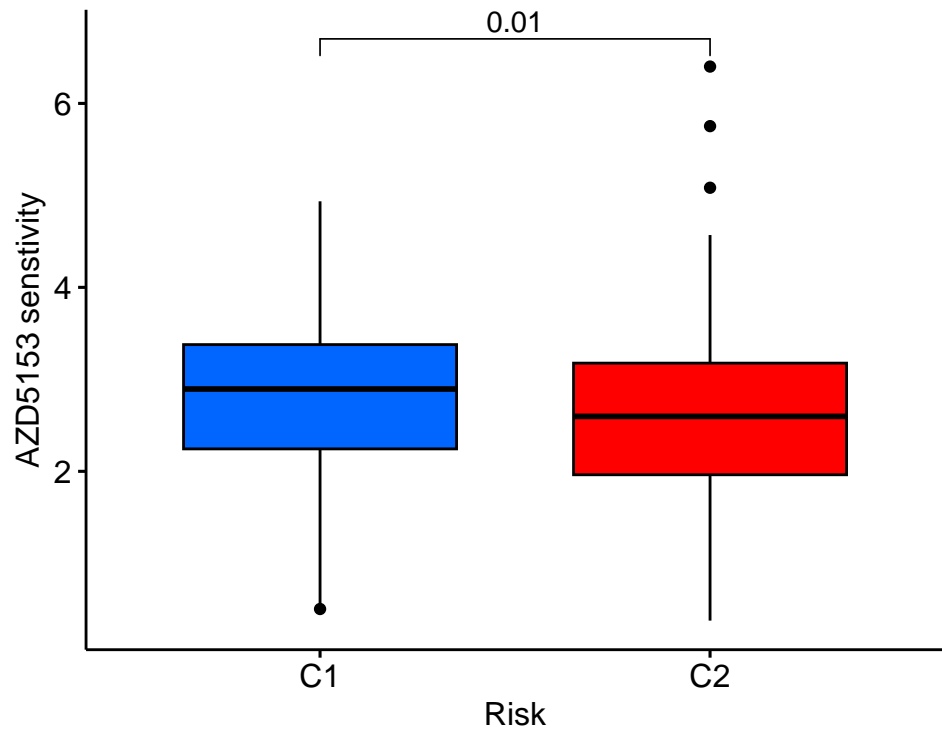

Supplement: Supplementary file 1 — Supplementary Information. [file 41598_2024_53257_MOESM1_ESM.zip › supplementary files/Drug sensitivity of C1 group and C2 group/C2 better/drugSenstivity.AZD5153.pdf]

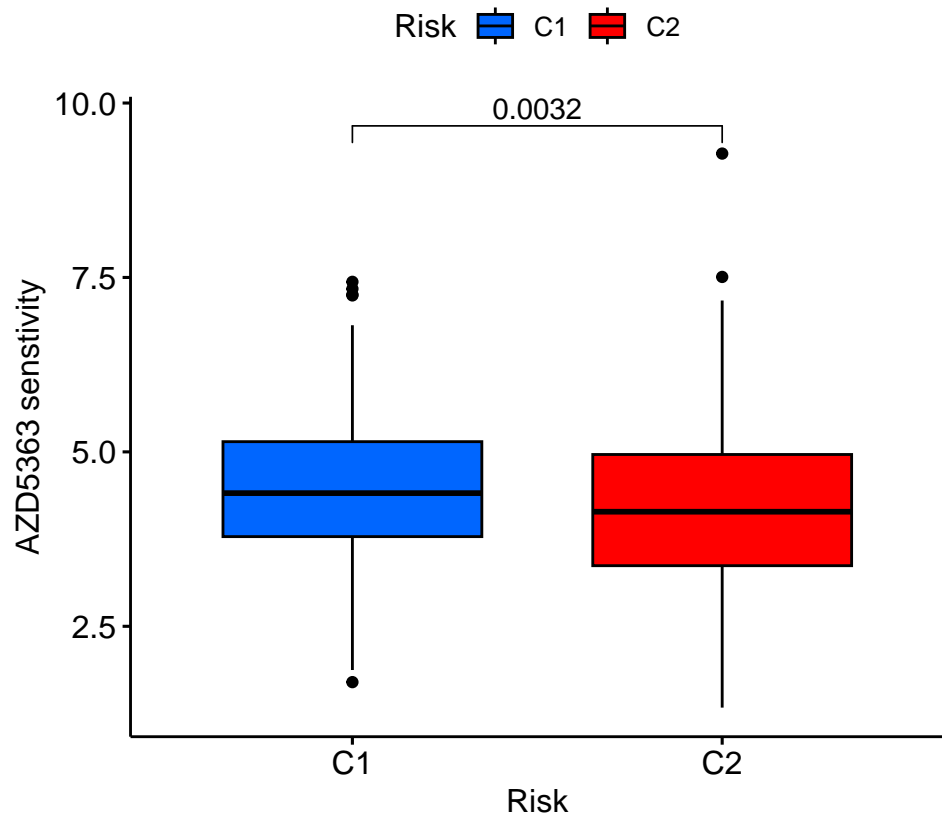

Supplement: Supplementary file 1 — Supplementary Information. [file 41598_2024_53257_MOESM1_ESM.zip › supplementary files/Drug sensitivity of C1 group and C2 group/C2 better/drugSenstivity.AZD5363.pdf]

AZD5438 sensitivity

Risk C1 C2

0.025

C1

C2

Risk

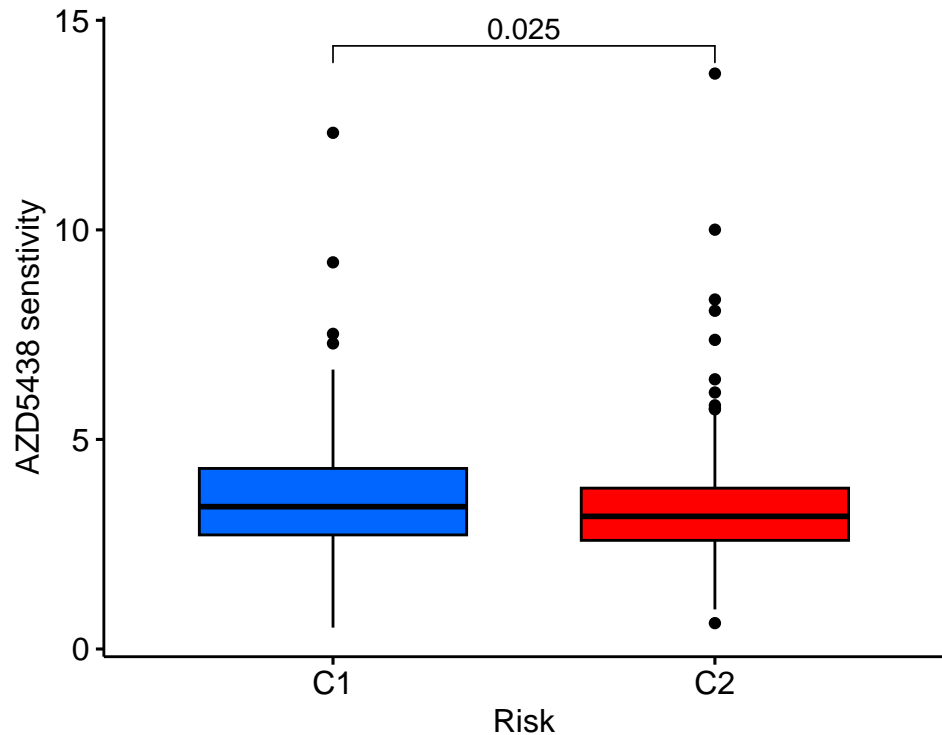

Supplement: Supplementary file 1 — Supplementary Information. [file 41598_2024_53257_MOESM1_ESM.zip › supplementary files/Drug sensitivity of C1 group and C2 group/C2 better/drugSenstivity.AZD5438.pdf]

Risk C1 C2

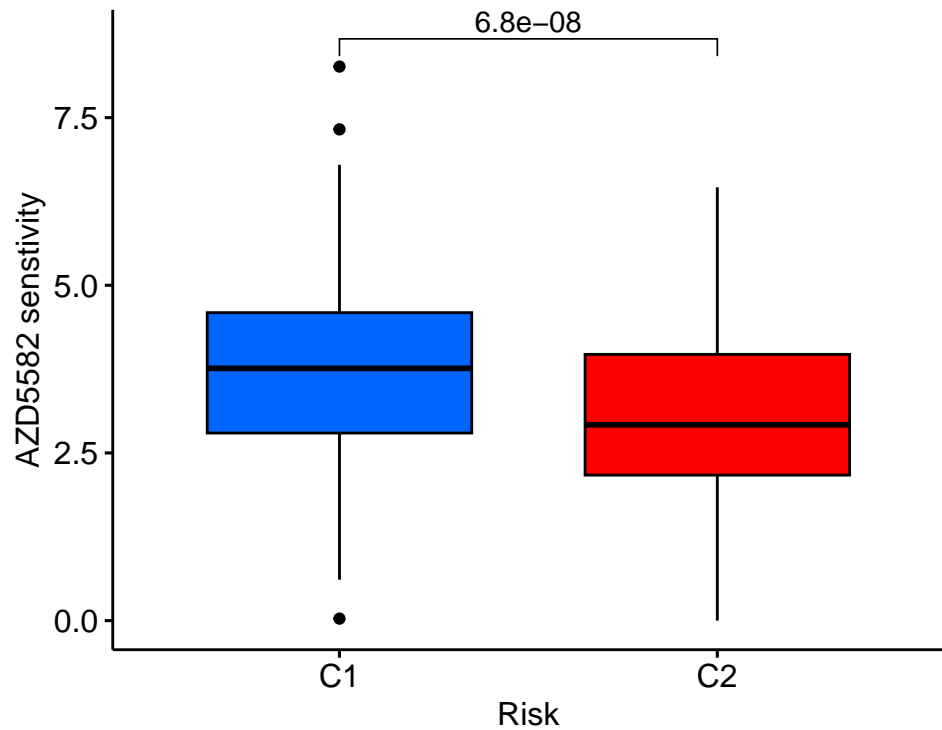

Supplement: Supplementary file 1 — Supplementary Information. [file 41598_2024_53257_MOESM1_ESM.zip › supplementary files/Drug sensitivity of C1 group and C2 group/C2 better/drugSenstivity.AZD5582.pdf]

Risk C1 C2

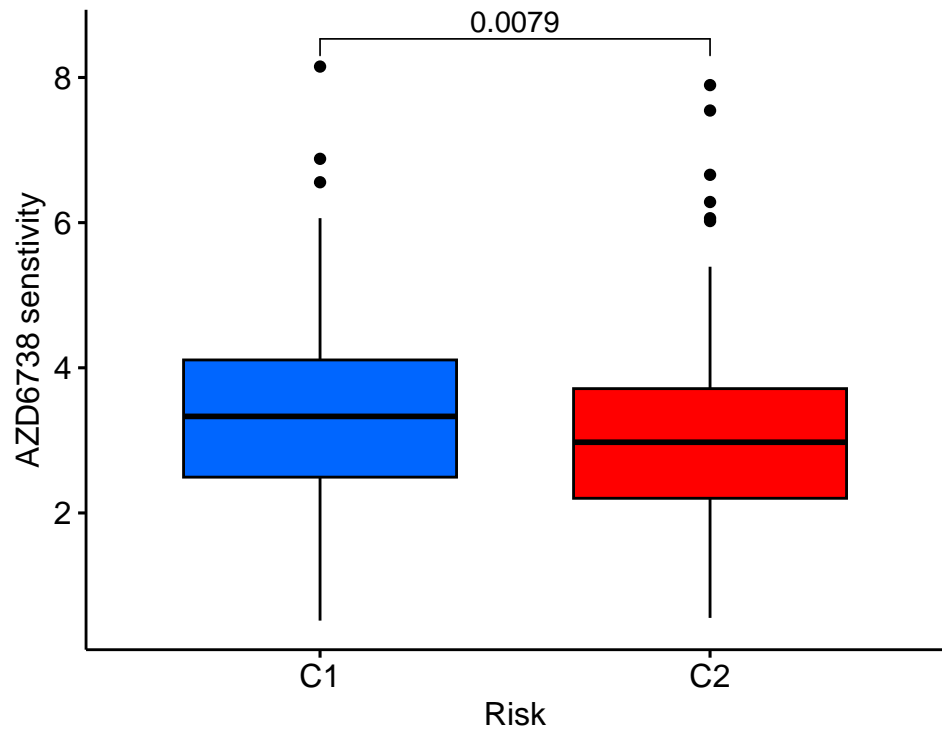

Supplement: Supplementary file 1 — Supplementary Information. [file 41598_2024_53257_MOESM1_ESM.zip › supplementary files/Drug sensitivity of C1 group and C2 group/C2 better/drugSenstivity.AZD6738.pdf]

Risk C1 C2

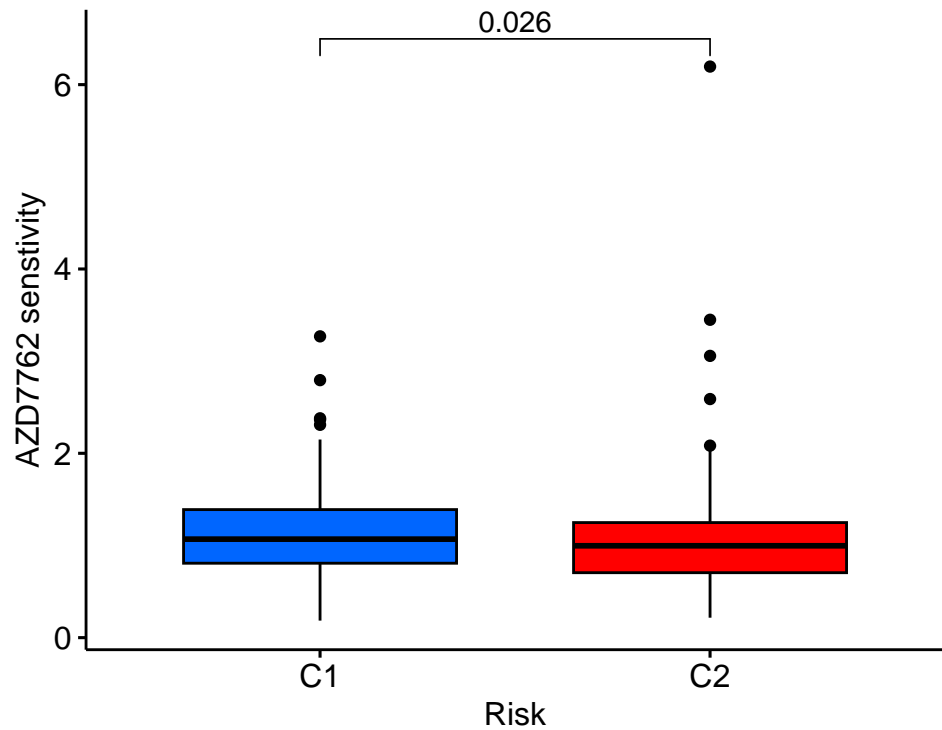

Supplement: Supplementary file 1 — Supplementary Information. [file 41598_2024_53257_MOESM1_ESM.zip › supplementary files/Drug sensitivity of C1 group and C2 group/C2 better/drugSenstivity.AZD7762.pdf]

Risk C1 C2

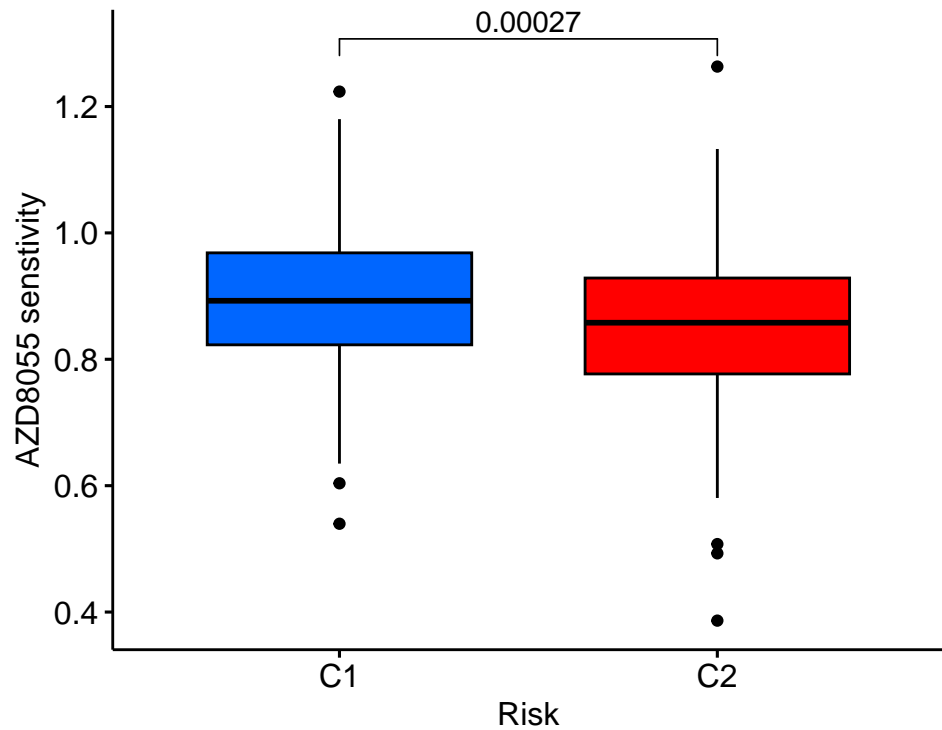

Supplement: Supplementary file 1 — Supplementary Information. [file 41598_2024_53257_MOESM1_ESM.zip › supplementary files/Drug sensitivity of C1 group and C2 group/C2 better/drugSenstivity.AZD8055.pdf]

AZD8186 sensitivity

Risk C1 C2

0.00011

C1

C2

Risk

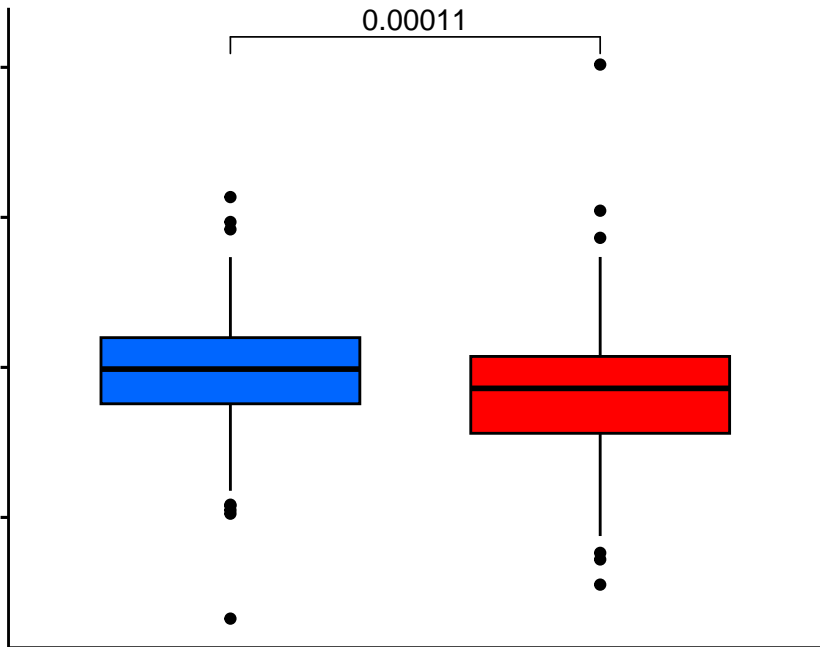

Supplement: Supplementary file 1 — Supplementary Information. [file 41598_2024_53257_MOESM1_ESM.zip › supplementary files/Drug sensitivity of C1 group and C2 group/C2 better/drugSenstivity.AZD8186.pdf]

Risk C1 C2

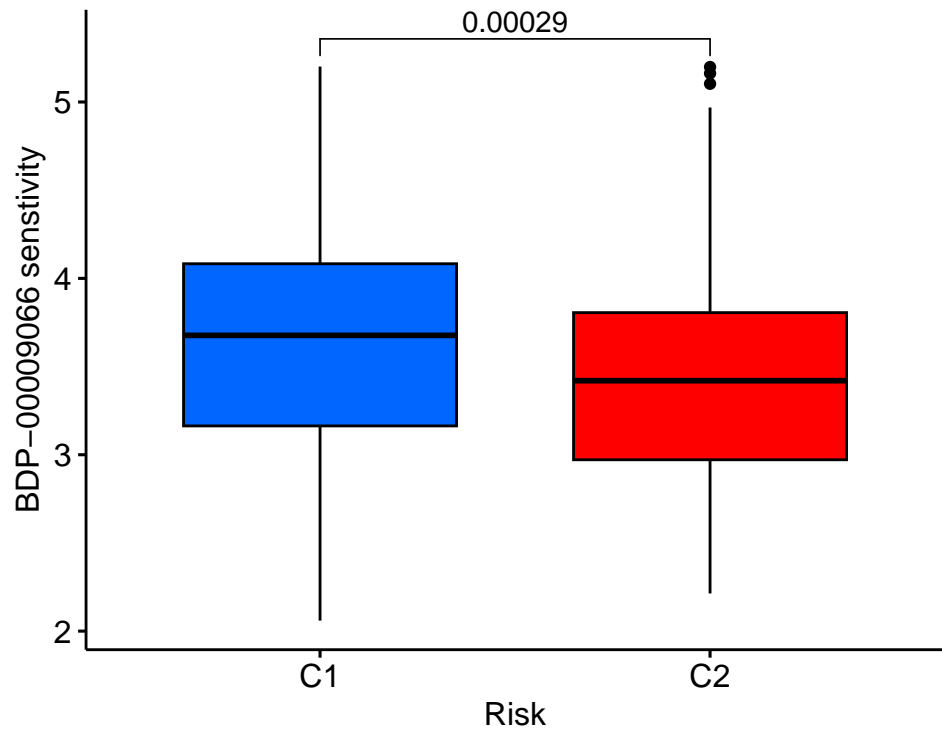

Supplement: Supplementary file 1 — Supplementary Information. [file 41598_2024_53257_MOESM1_ESM.zip › supplementary files/Drug sensitivity of C1 group and C2 group/C2 better/drugSenstivity.BDP-00009066.pdf]

BMS-345541 sensitivity

Risk C1 C2

0.021

C1

C2

Risk

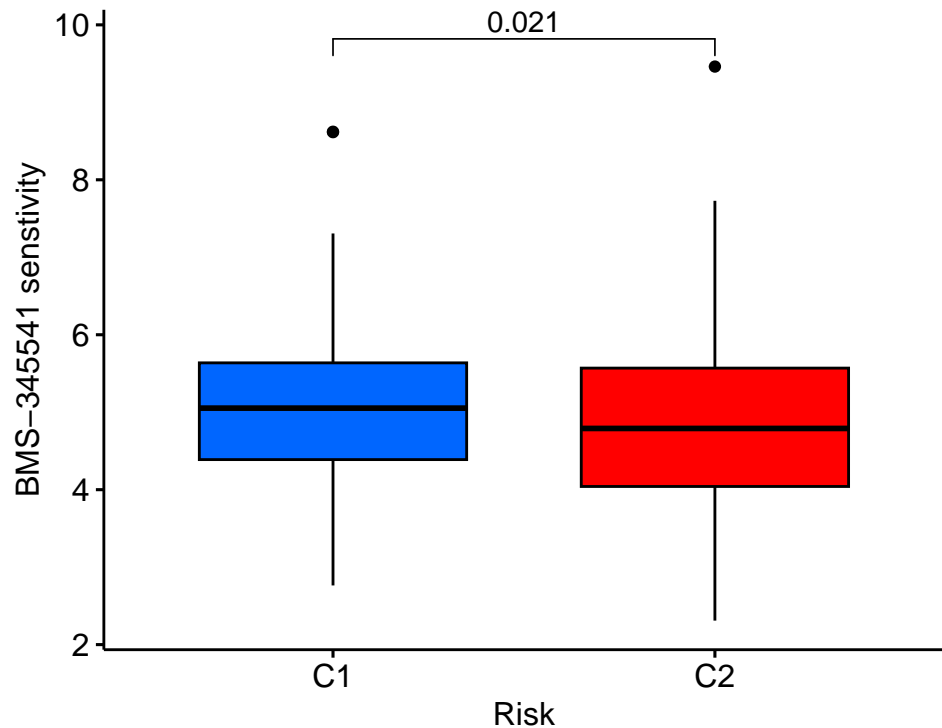

Supplement: Supplementary file 1 — Supplementary Information. [file 41598_2024_53257_MOESM1_ESM.zip › supplementary files/Drug sensitivity of C1 group and C2 group/C2 better/drugSenstivity.BMS-345541.pdf]

Risk C1 C2

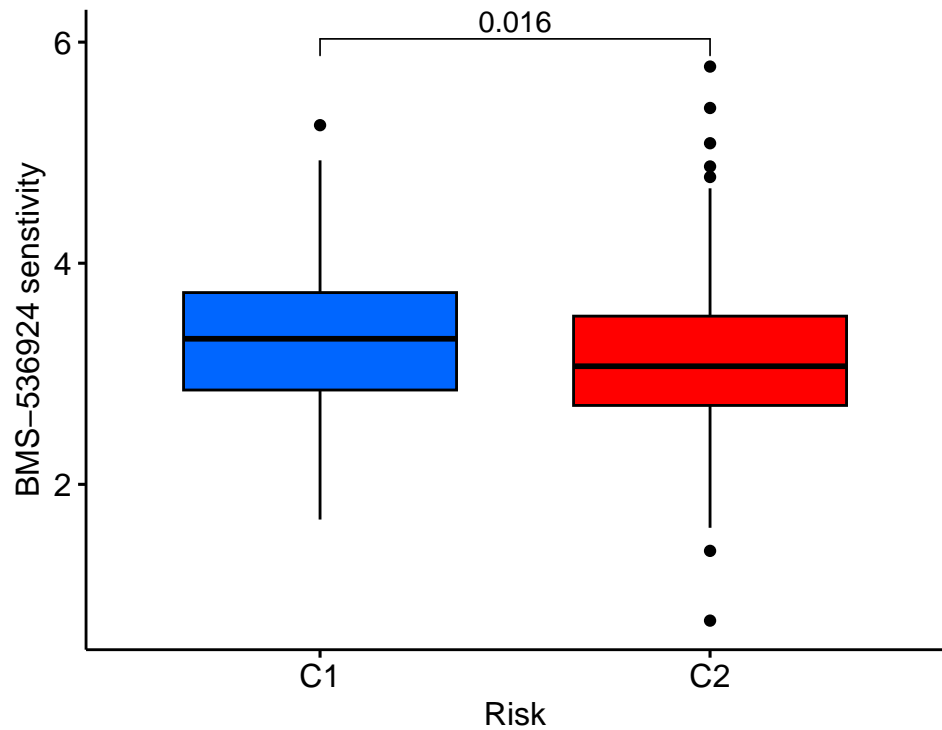

Supplement: Supplementary file 1 — Supplementary Information. [file 41598_2024_53257_MOESM1_ESM.zip › supplementary files/Drug sensitivity of C1 group and C2 group/C2 better/drugSenstivity.BMS-536924.pdf]

Risk C1 C2

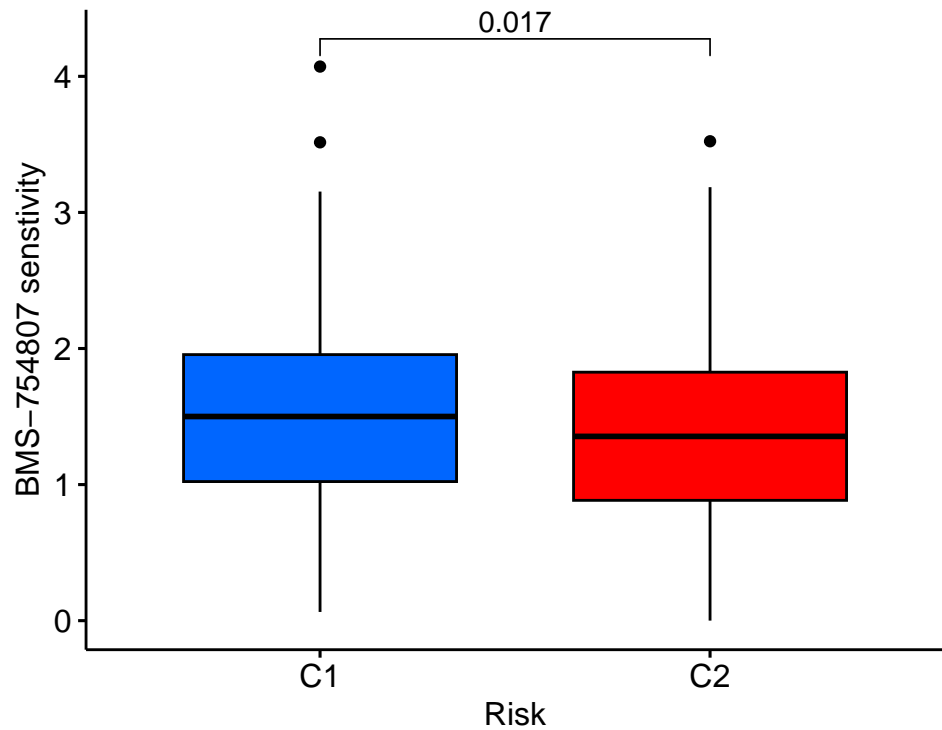

Supplement: Supplementary file 1 — Supplementary Information. [file 41598_2024_53257_MOESM1_ESM.zip › supplementary files/Drug sensitivity of C1 group and C2 group/C2 better/drugSenstivity.BMS-754807.pdf]

Risk C1 C2

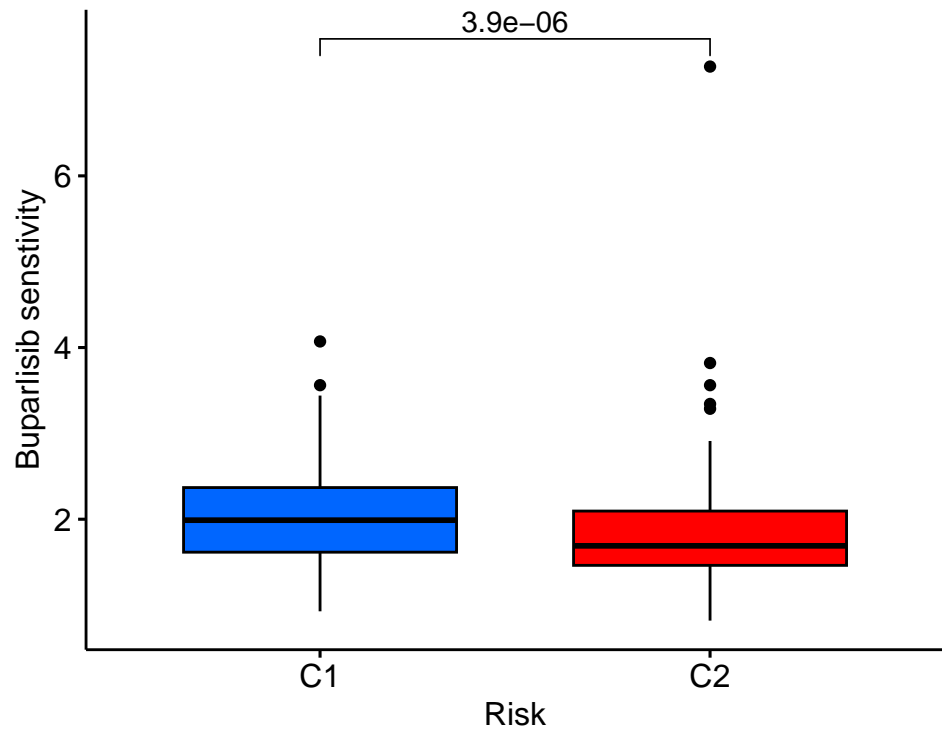

Supplement: Supplementary file 1 — Supplementary Information. [file 41598_2024_53257_MOESM1_ESM.zip › supplementary files/Drug sensitivity of C1 group and C2 group/C2 better/drugSenstivity.Buparlisib.pdf]

Risk C1 C2

0.00012

Camptothecin sensitivity

4

3

2

1

0

C1

C2

Risk

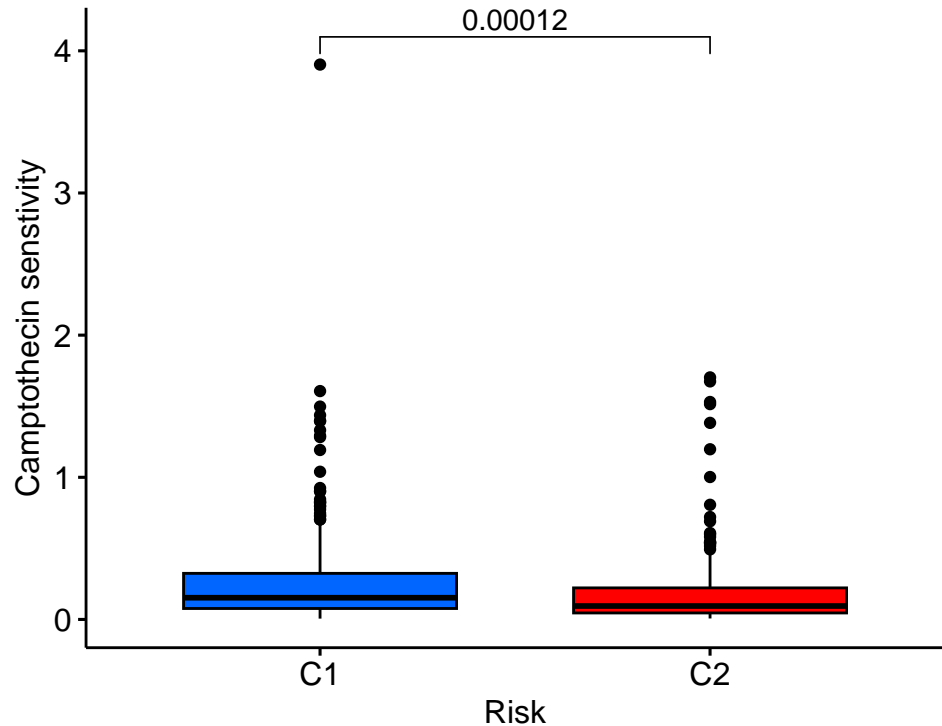

Supplement: Supplementary file 1 — Supplementary Information. [file 41598_2024_53257_MOESM1_ESM.zip › supplementary files/Drug sensitivity of C1 group and C2 group/C2 better/drugSenstivity.Camptothecin.pdf]

Risk C1 C2

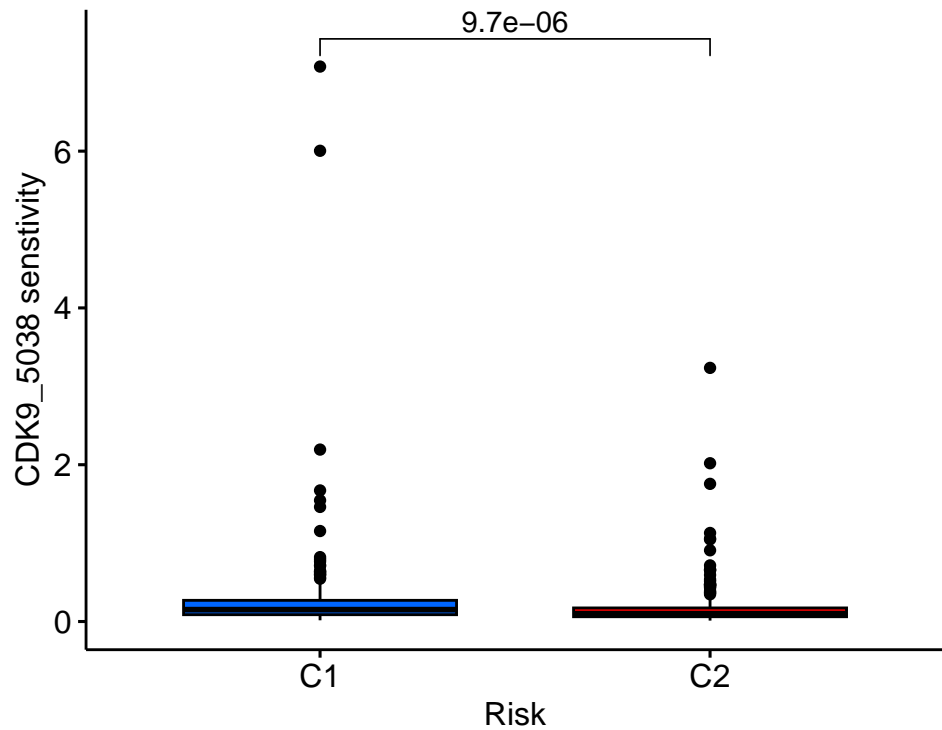

Supplement: Supplementary file 1 — Supplementary Information. [file 41598_2024_53257_MOESM1_ESM.zip › supplementary files/Drug sensitivity of C1 group and C2 group/C2 better/drugSenstivity.CDK9_5038.pdf]

CDK9\_5576 sensitivity

Risk C1 C2

0.0029

20

15

10

5

0

C1

C2

Risk

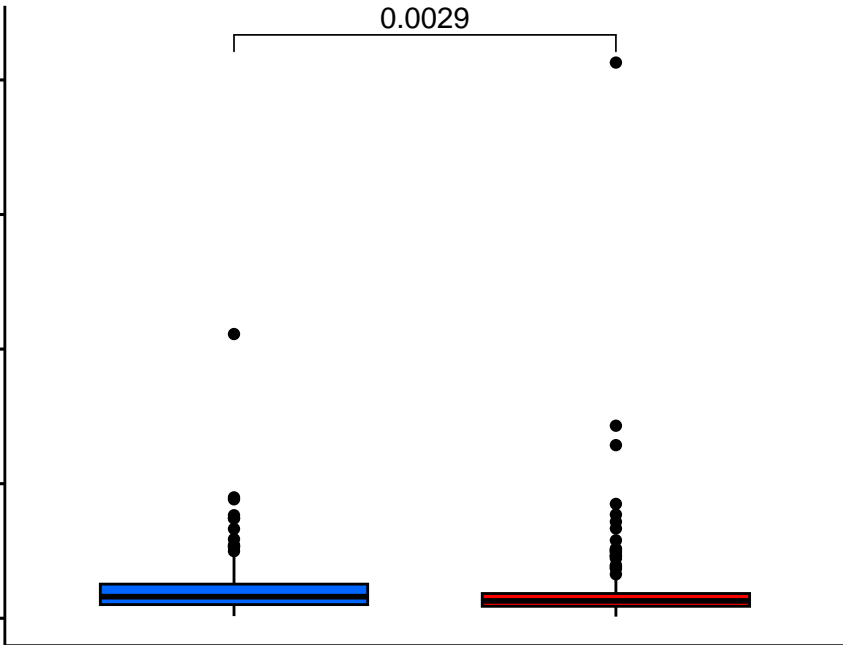

Supplement: Supplementary file 1 — Supplementary Information. [file 41598_2024_53257_MOESM1_ESM.zip › supplementary files/Drug sensitivity of C1 group and C2 group/C2 better/drugSenstivity.CDK9_5576.pdf]

Risk C1 C2

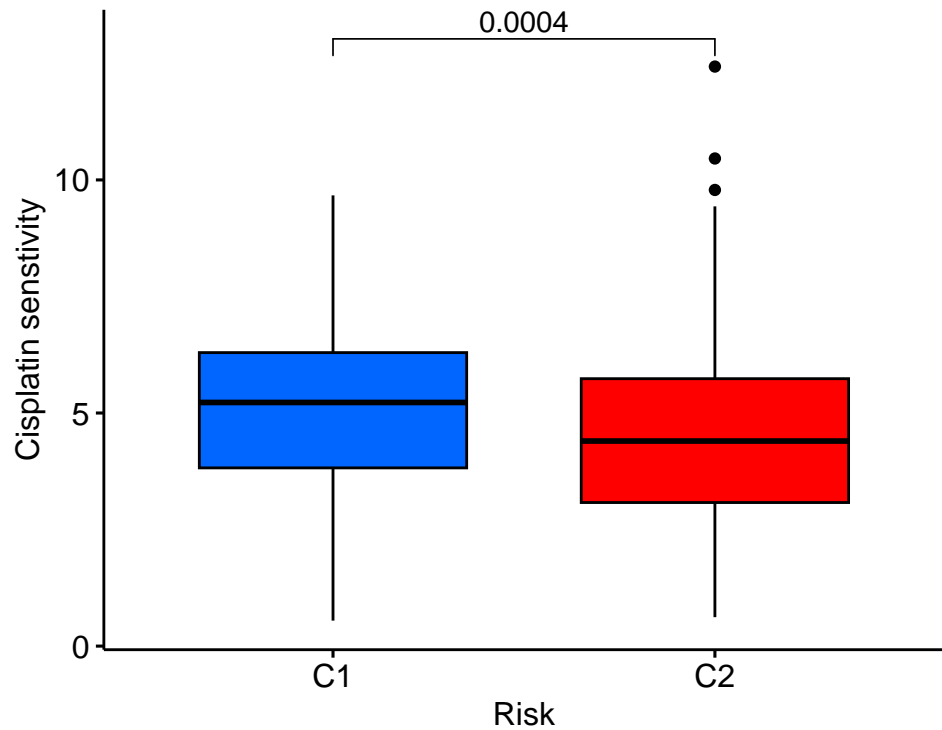

Supplement: Supplementary file 1 — Supplementary Information. [file 41598_2024_53257_MOESM1_ESM.zip › supplementary files/Drug sensitivity of C1 group and C2 group/C2 better/drugSenstivity.Cisplatin.pdf]

Risk C1 C2

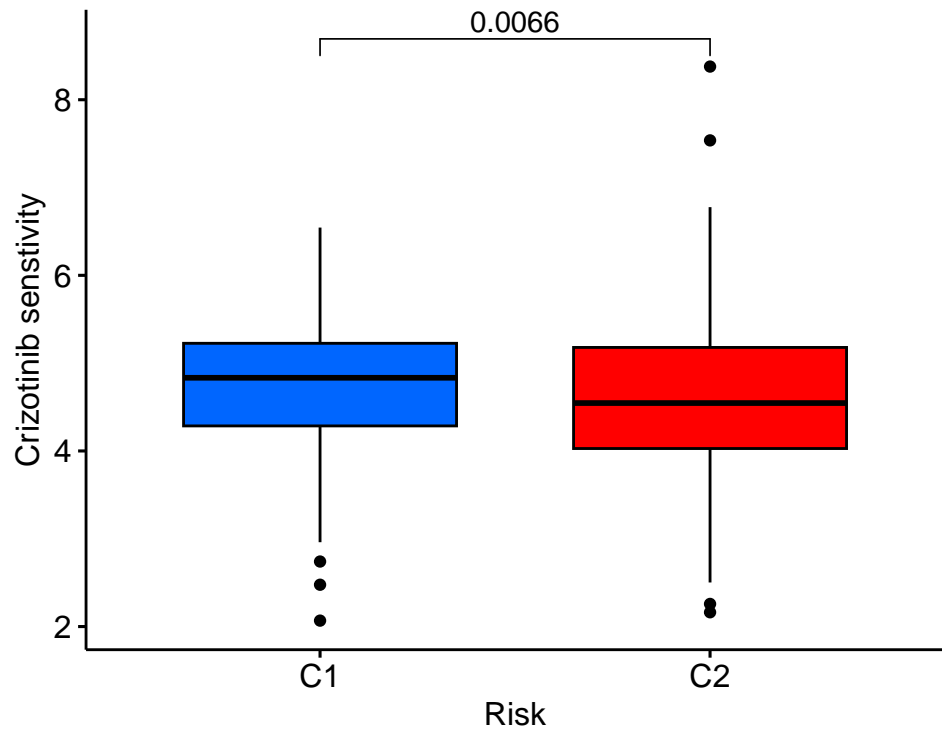

Supplement: Supplementary file 1 — Supplementary Information. [file 41598_2024_53257_MOESM1_ESM.zip › supplementary files/Drug sensitivity of C1 group and C2 group/C2 better/drugSenstivity.Crizotinib.pdf]

Risk 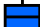 C1 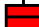 C2

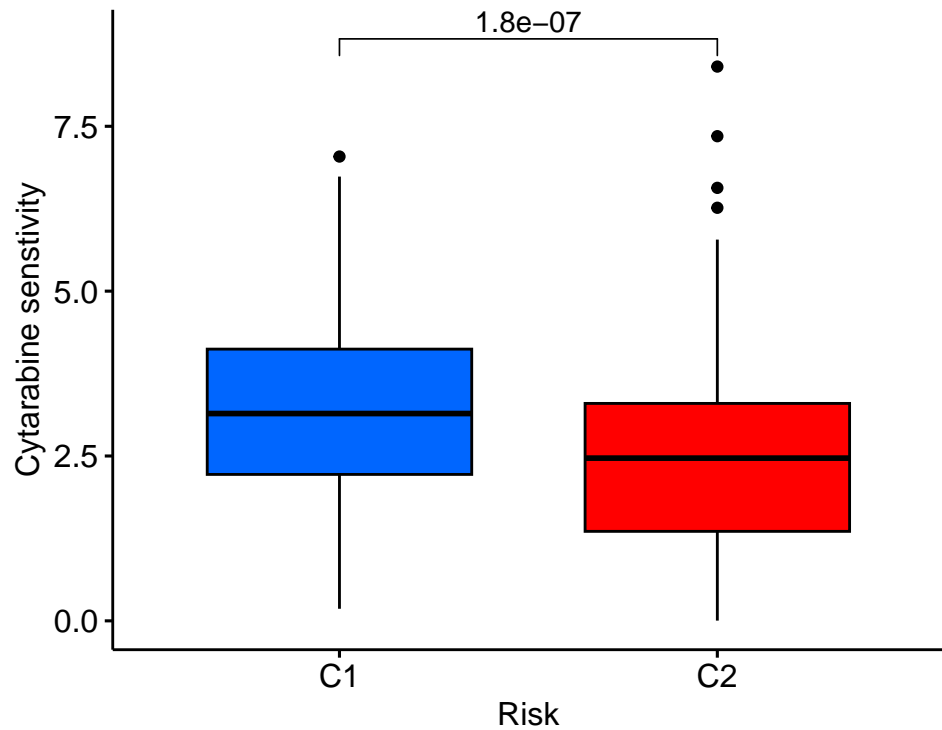

Supplement: Supplementary file 1 — Supplementary Information. [file 41598_2024_53257_MOESM1_ESM.zip › supplementary files/Drug sensitivity of C1 group and C2 group/C2 better/drugSenstivity.Cytarabine.pdf]

Risk C1 C2

1.9e-07

Dabrafenib sensitivity

10

8

6

4

2

C1

C2

Risk

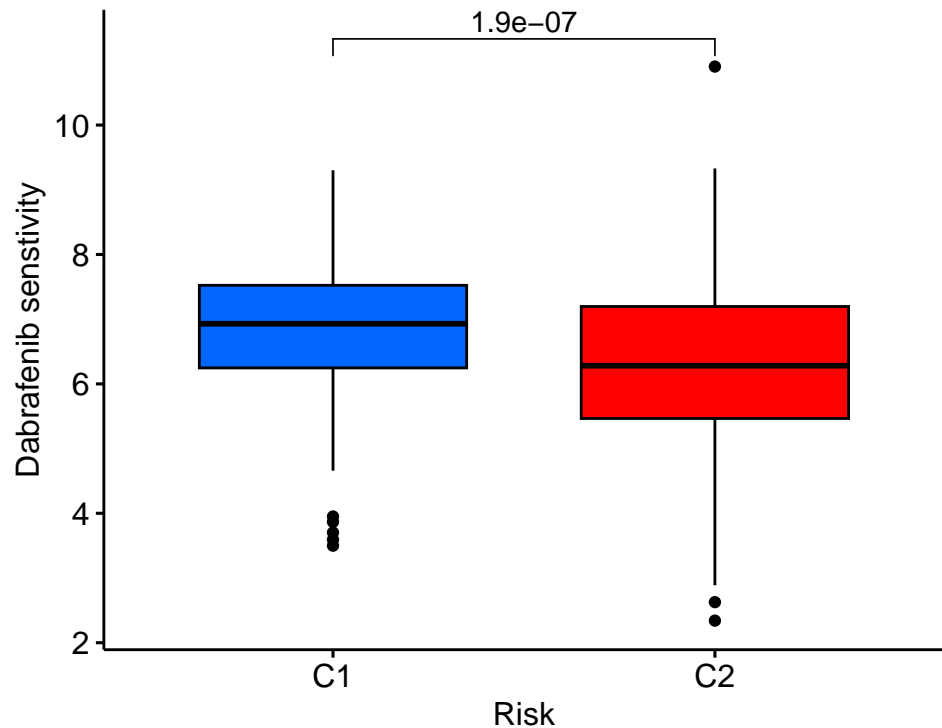

Supplement: Supplementary file 1 — Supplementary Information. [file 41598_2024_53257_MOESM1_ESM.zip › supplementary files/Drug sensitivity of C1 group and C2 group/C2 better/drugSenstivity.Dabrafenib.pdf]

Risk C1 C2

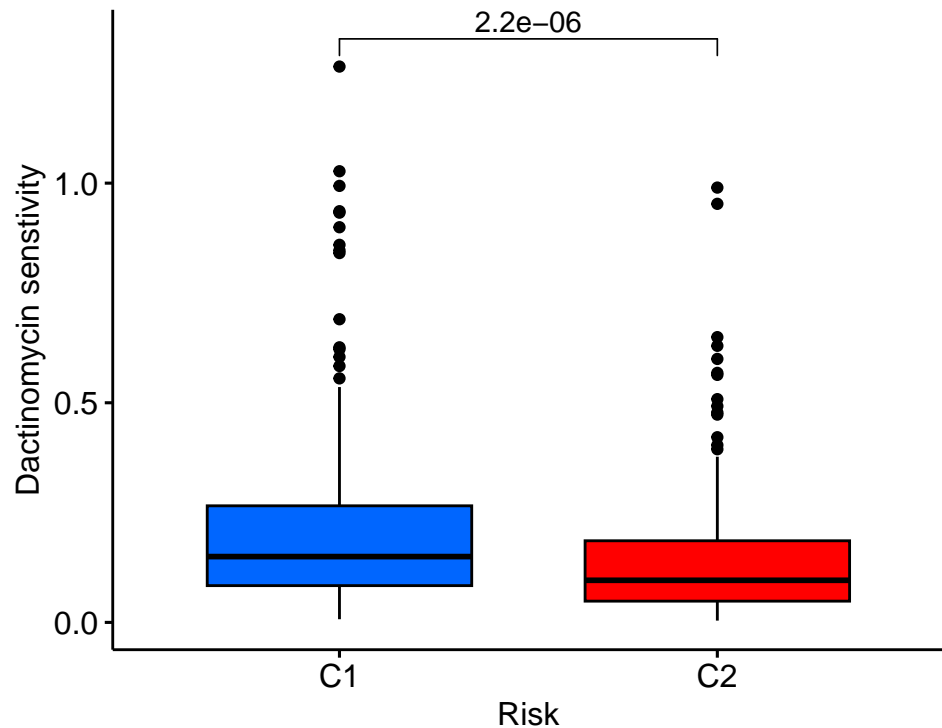

Supplement: Supplementary file 1 — Supplementary Information. [file 41598_2024_53257_MOESM1_ESM.zip › supplementary files/Drug sensitivity of C1 group and C2 group/C2 better/drugSenstivity.Dactinomycin.pdf]

Risk C1 C2

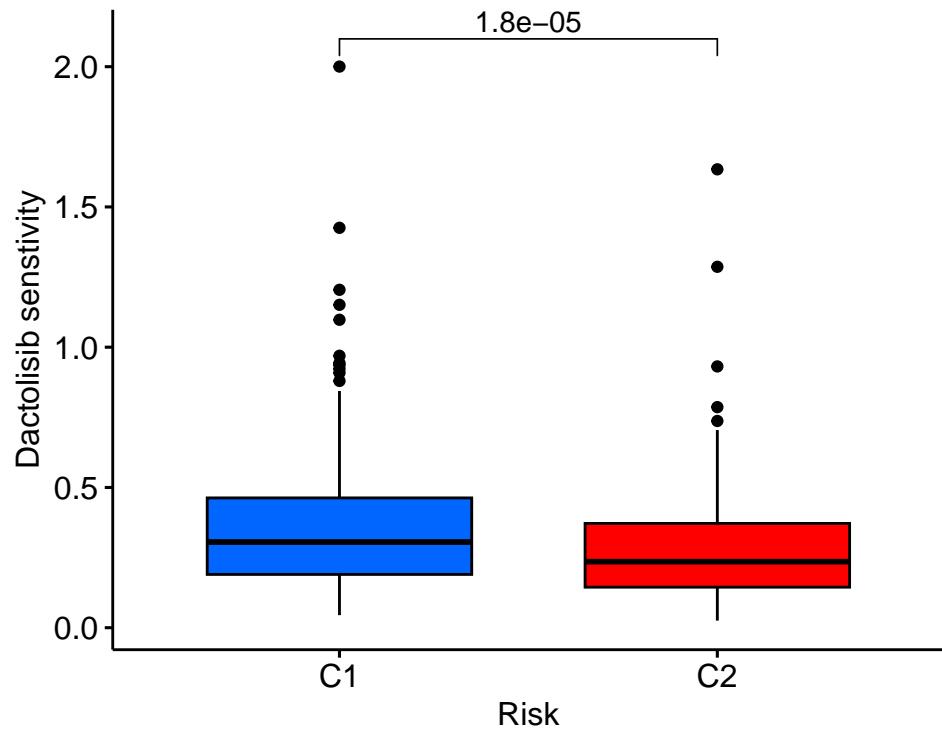

Supplement: Supplementary file 1 — Supplementary Information. [file 41598_2024_53257_MOESM1_ESM.zip › supplementary files/Drug sensitivity of C1 group and C2 group/C2 better/drugSenstivity.Dactolisib.pdf]

Risk C1 C2

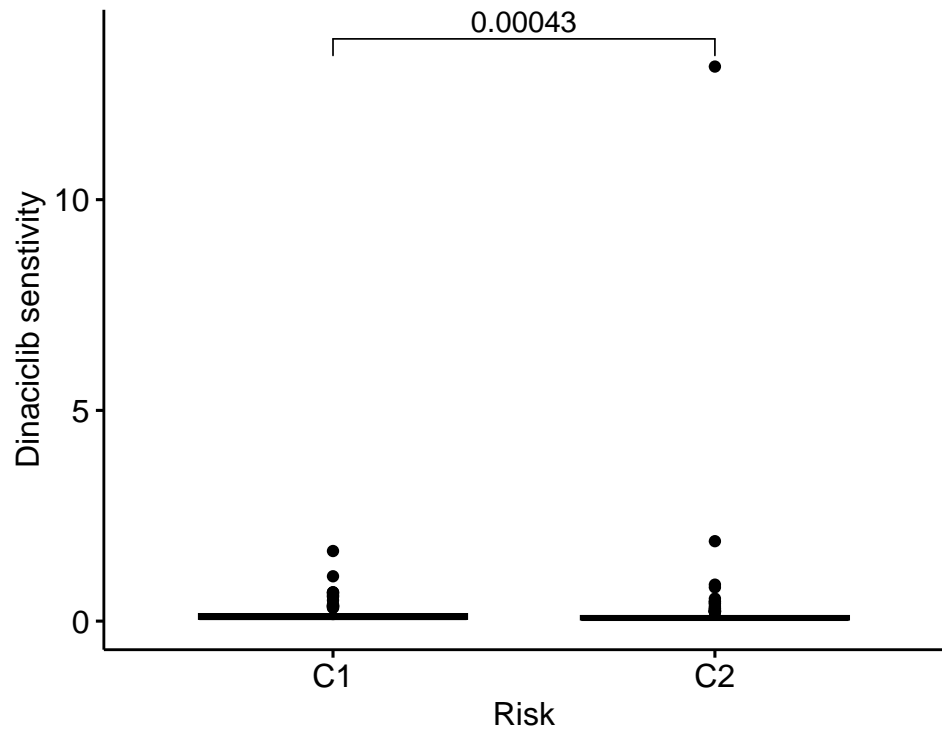

Supplement: Supplementary file 1 — Supplementary Information. [file 41598_2024_53257_MOESM1_ESM.zip › supplementary files/Drug sensitivity of C1 group and C2 group/C2 better/drugSenstivity.Dinaciclib.pdf]

Risk C1 C2

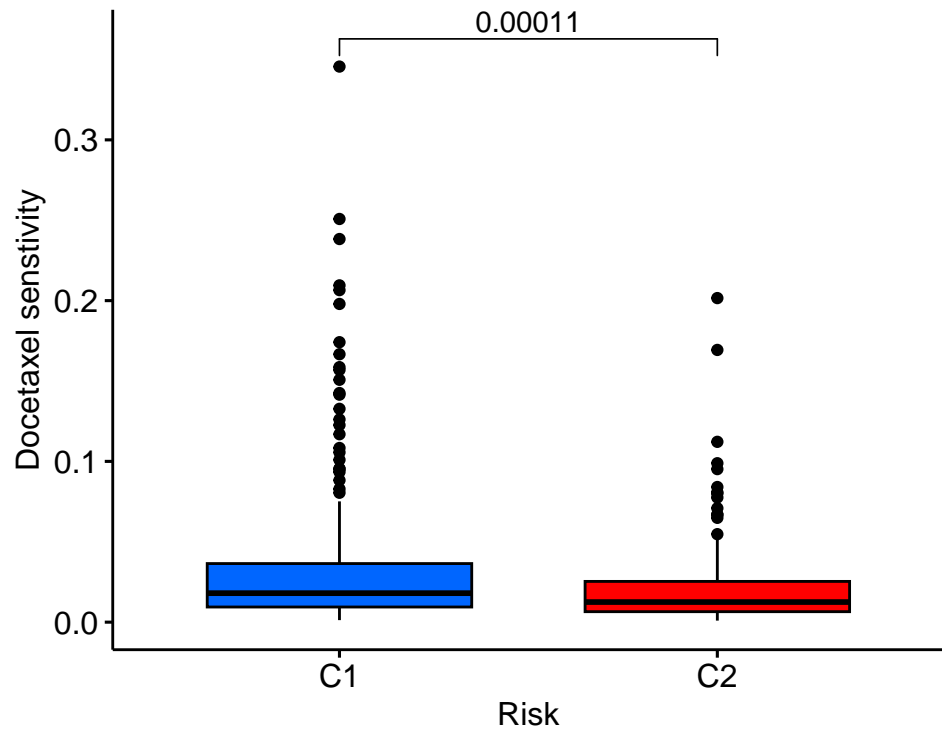

Supplement: Supplementary file 1 — Supplementary Information. [file 41598_2024_53257_MOESM1_ESM.zip › supplementary files/Drug sensitivity of C1 group and C2 group/C2 better/drugSenstivity.Docetaxel.pdf]

Risk C1 C2

1.1e-05

Eg5\_9814 sensitivity

7.5

5.0

2.5

0.0

C1

C2

Risk

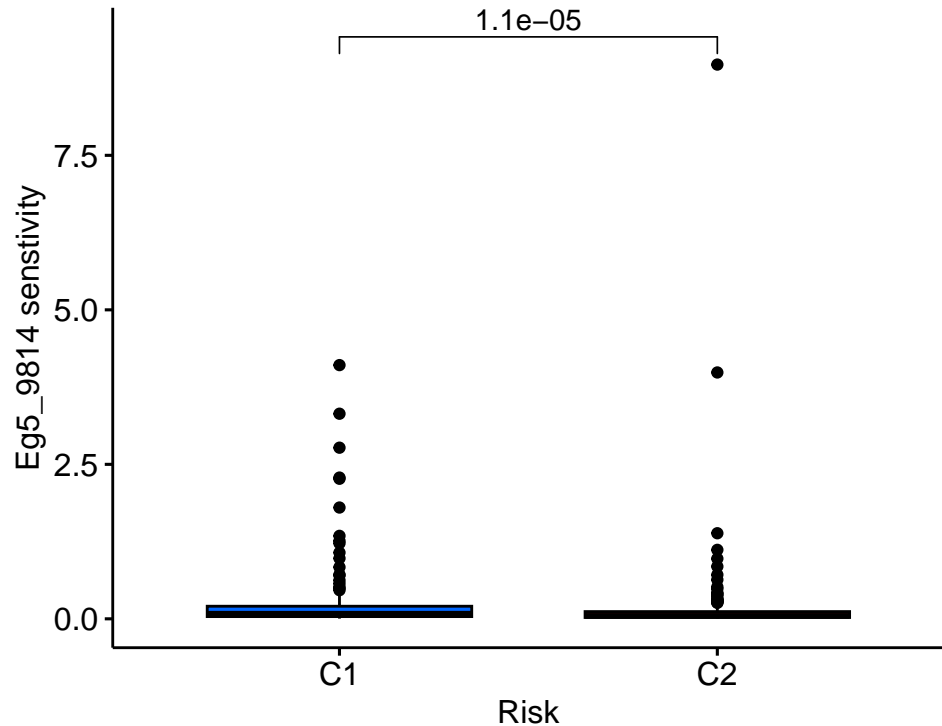

Supplement: Supplementary file 1 — Supplementary Information. [file 41598_2024_53257_MOESM1_ESM.zip › supplementary files/Drug sensitivity of C1 group and C2 group/C2 better/drugSenstivity.Eg5_9814.pdf]

Risk C1 C2

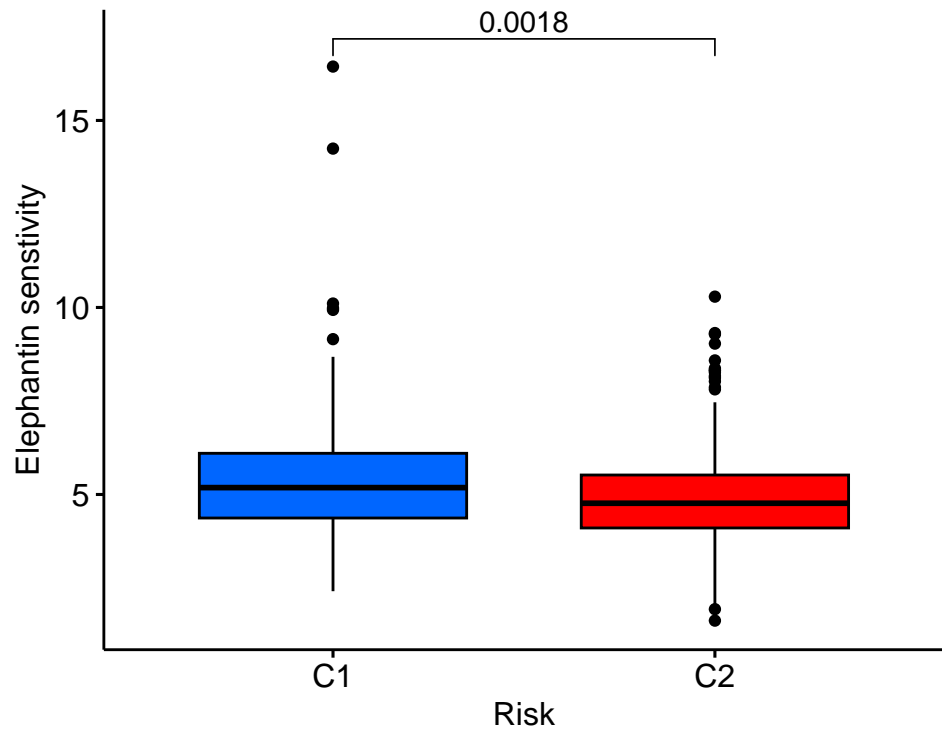

Supplement: Supplementary file 1 — Supplementary Information. [file 41598_2024_53257_MOESM1_ESM.zip › supplementary files/Drug sensitivity of C1 group and C2 group/C2 better/drugSenstivity.Elephantin.pdf]

Risk C1 C2

6.3e-06

Entinostat sensitivity

8

6

4

2

C1

C2

Risk

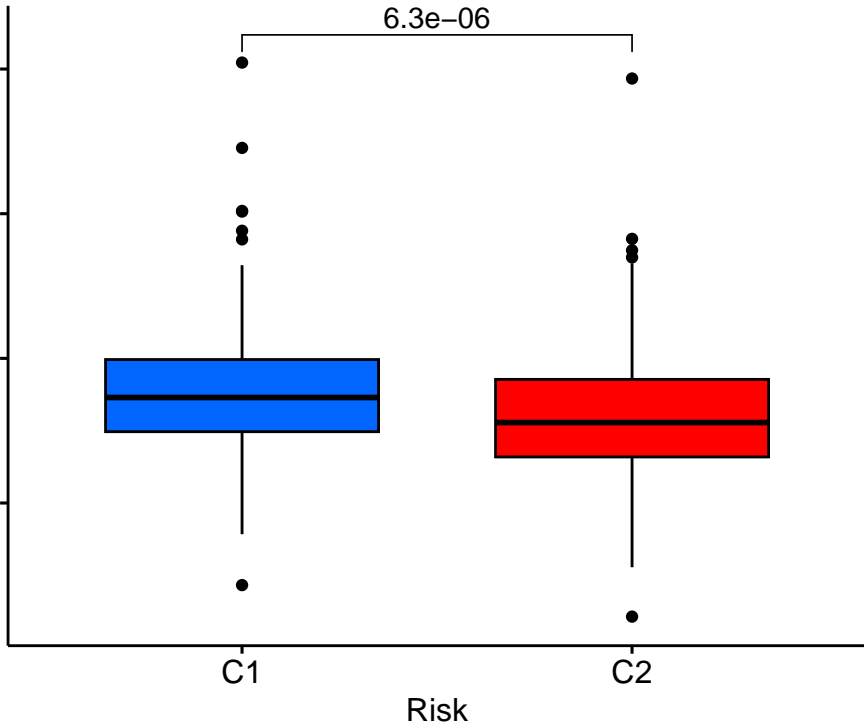

Supplement: Supplementary file 1 — Supplementary Information. [file 41598_2024_53257_MOESM1_ESM.zip › supplementary files/Drug sensitivity of C1 group and C2 group/C2 better/drugSenstivity.Entinostat.pdf]

Risk C1 C2

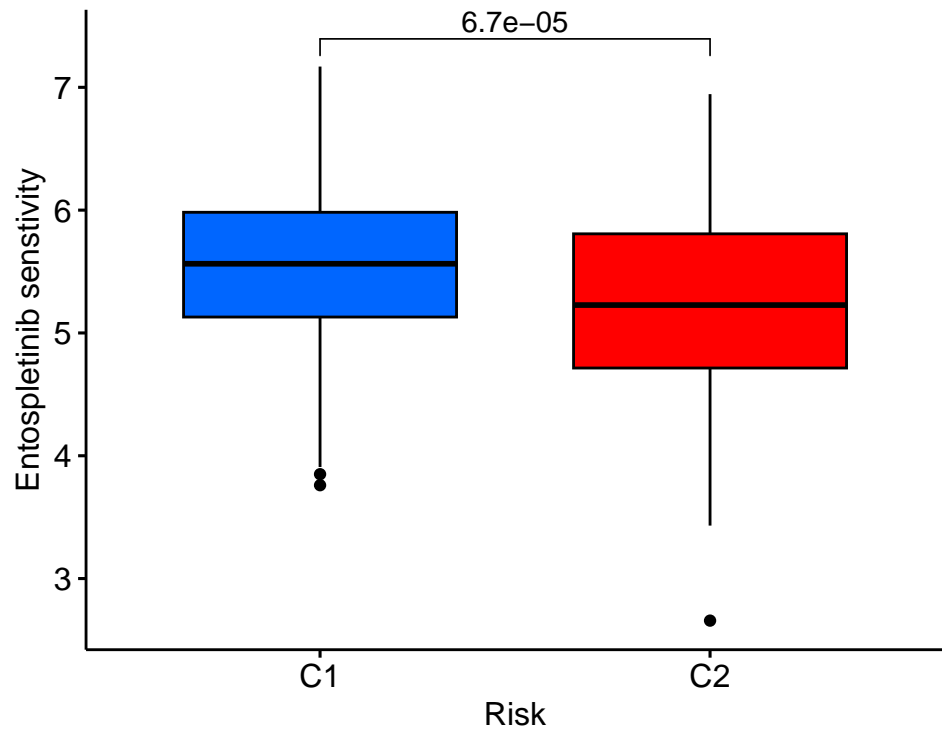

Supplement: Supplementary file 1 — Supplementary Information. [file 41598_2024_53257_MOESM1_ESM.zip › supplementary files/Drug sensitivity of C1 group and C2 group/C2 better/drugSenstivity.Entospletinib.pdf]

Risk C1 C2

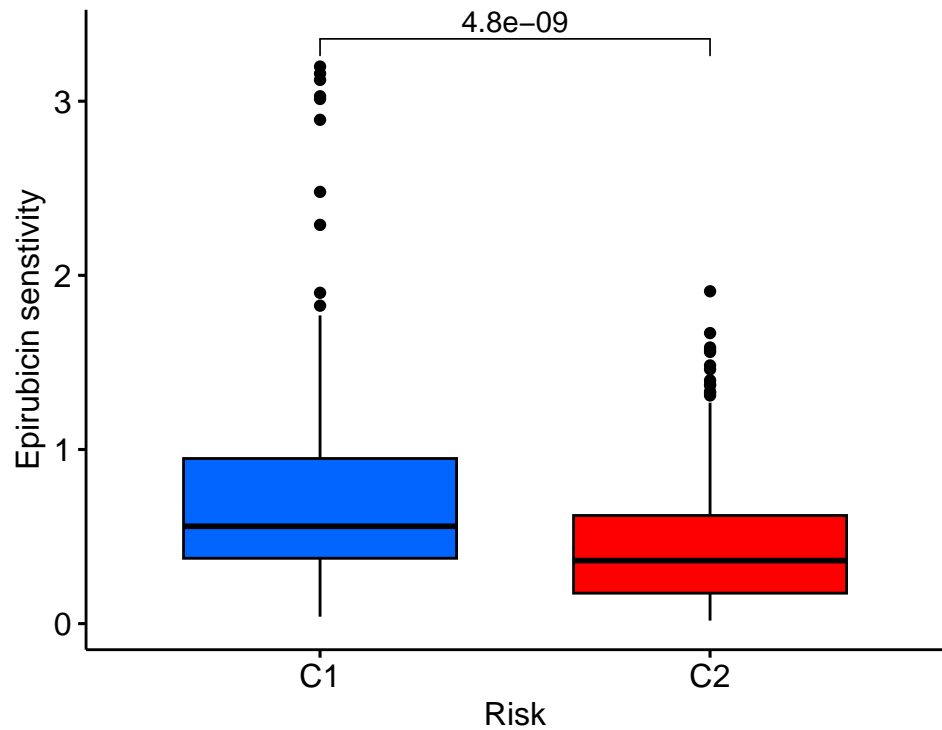

Supplement: Supplementary file 1 — Supplementary Information. [file 41598_2024_53257_MOESM1_ESM.zip › supplementary files/Drug sensitivity of C1 group and C2 group/C2 better/drugSenstivity.Epirubicin.pdf]

Risk C1 C2

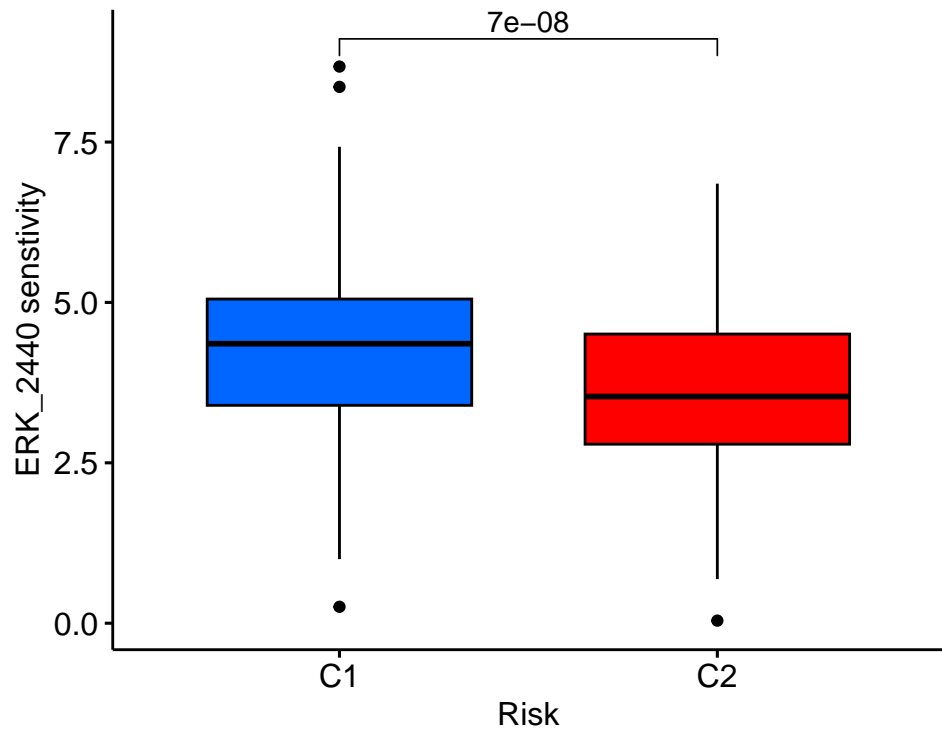

Supplement: Supplementary file 1 — Supplementary Information. [file 41598_2024_53257_MOESM1_ESM.zip › supplementary files/Drug sensitivity of C1 group and C2 group/C2 better/drugSenstivity.ERK_2440.pdf]

Risk C1 C2

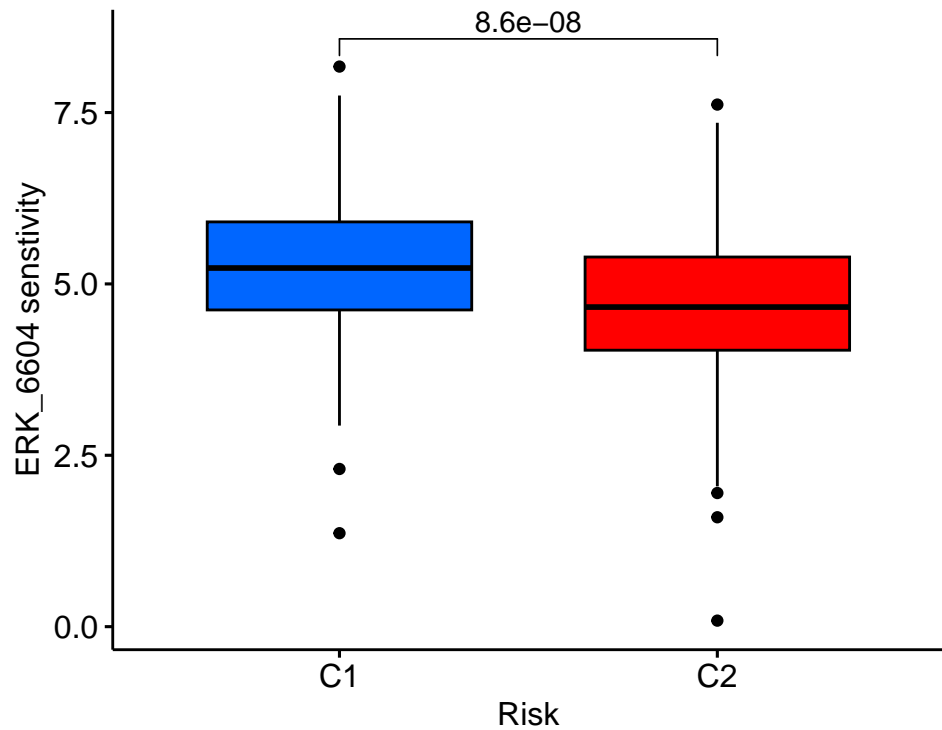

Supplement: Supplementary file 1 — Supplementary Information. [file 41598_2024_53257_MOESM1_ESM.zip › supplementary files/Drug sensitivity of C1 group and C2 group/C2 better/drugSenstivity.ERK_6604.pdf]

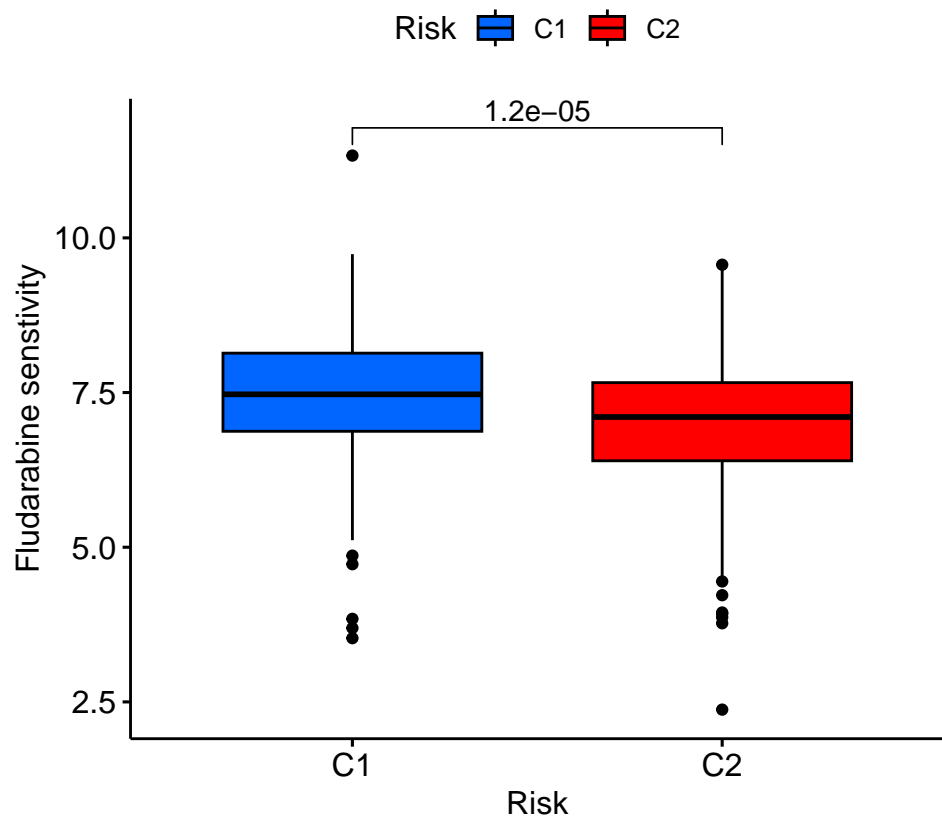

Supplement: Supplementary file 1 — Supplementary Information. [file 41598_2024_53257_MOESM1_ESM.zip › supplementary files/Drug sensitivity of C1 group and C2 group/C2 better/drugSenstivity.Fludarabine.pdf]

Risk C1 C2

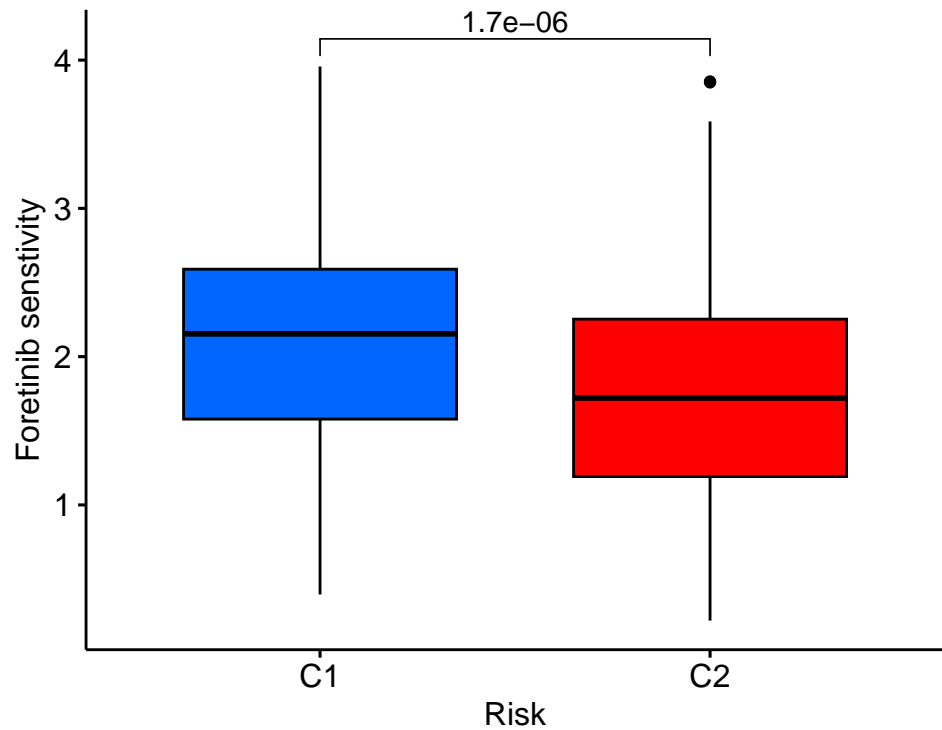

Supplement: Supplementary file 1 — Supplementary Information. [file 41598_2024_53257_MOESM1_ESM.zip › supplementary files/Drug sensitivity of C1 group and C2 group/C2 better/drugSenstivity.Foretinib.pdf]

Risk C1 C2

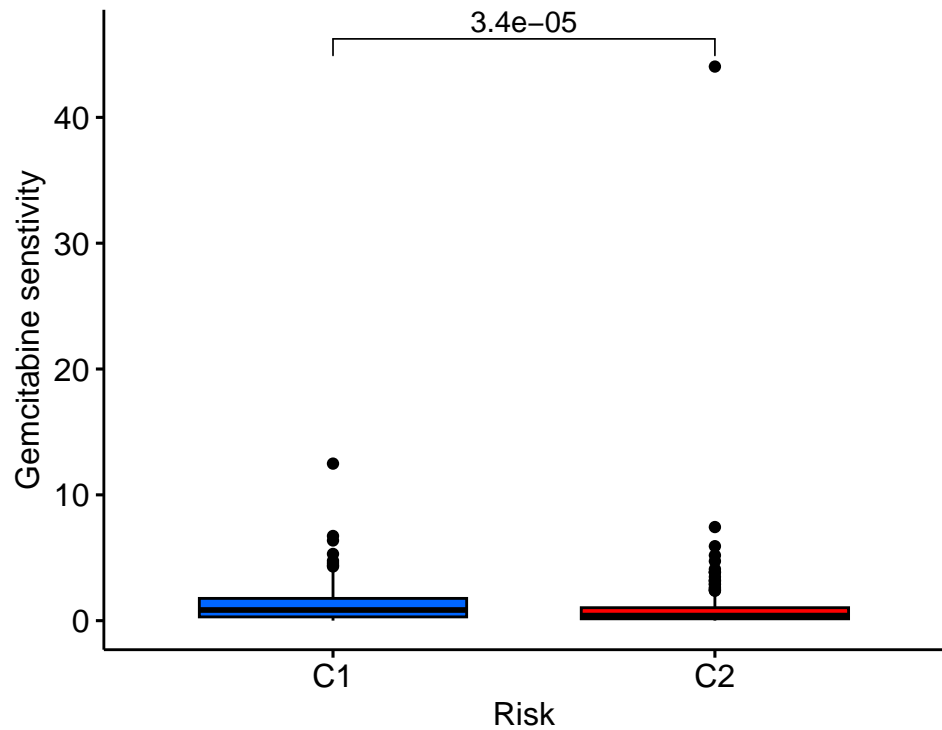

Supplement: Supplementary file 1 — Supplementary Information. [file 41598_2024_53257_MOESM1_ESM.zip › supplementary files/Drug sensitivity of C1 group and C2 group/C2 better/drugSenstivity.Gemcitabine.pdf]

Risk C1 C2

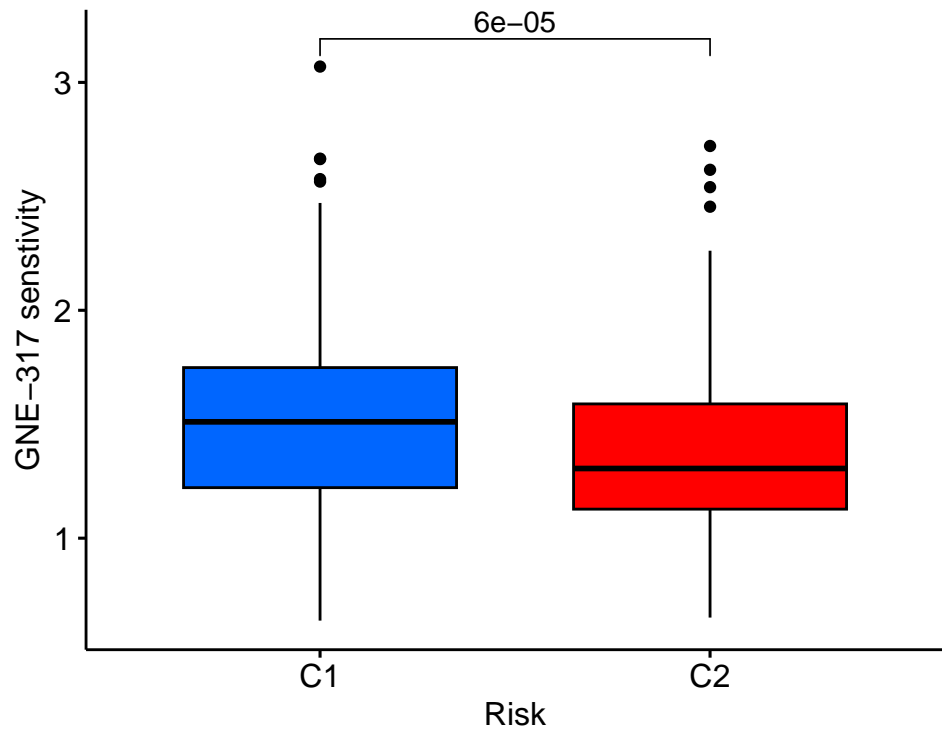

Supplement: Supplementary file 1 — Supplementary Information. [file 41598_2024_53257_MOESM1_ESM.zip › supplementary files/Drug sensitivity of C1 group and C2 group/C2 better/drugSenstivity.GNE-317.pdf]

Risk C1 C2

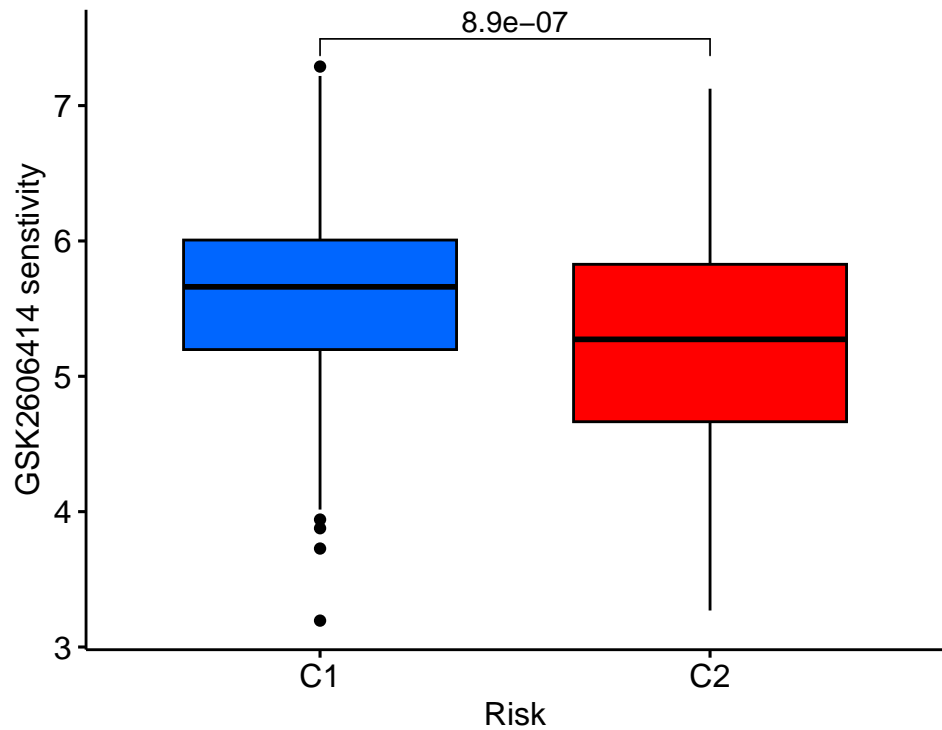

Supplement: Supplementary file 1 — Supplementary Information. [file 41598_2024_53257_MOESM1_ESM.zip › supplementary files/Drug sensitivity of C1 group and C2 group/C2 better/drugSenstivity.GSK2606414.pdf]

Risk C1 C2

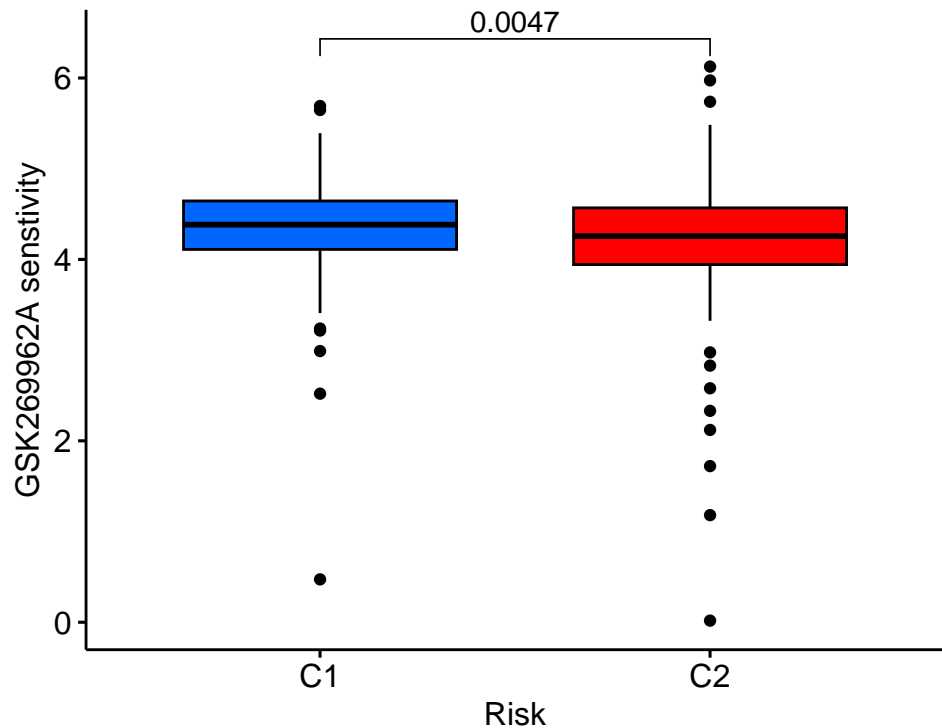

Supplement: Supplementary file 1 — Supplementary Information. [file 41598_2024_53257_MOESM1_ESM.zip › supplementary files/Drug sensitivity of C1 group and C2 group/C2 better/drugSenstivity.GSK269962A.pdf]

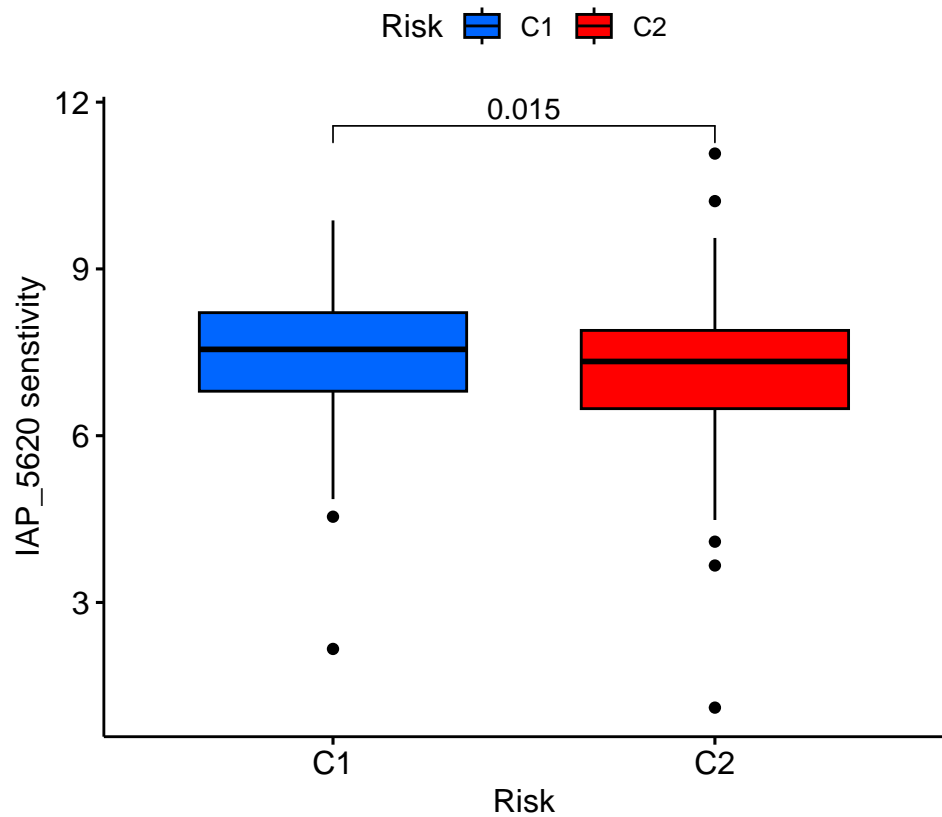

Supplement: Supplementary file 1 — Supplementary Information. [file 41598_2024_53257_MOESM1_ESM.zip › supplementary files/Drug sensitivity of C1 group and C2 group/C2 better/drugSenstivity.IAP_5620.pdf]

Risk C1 C2

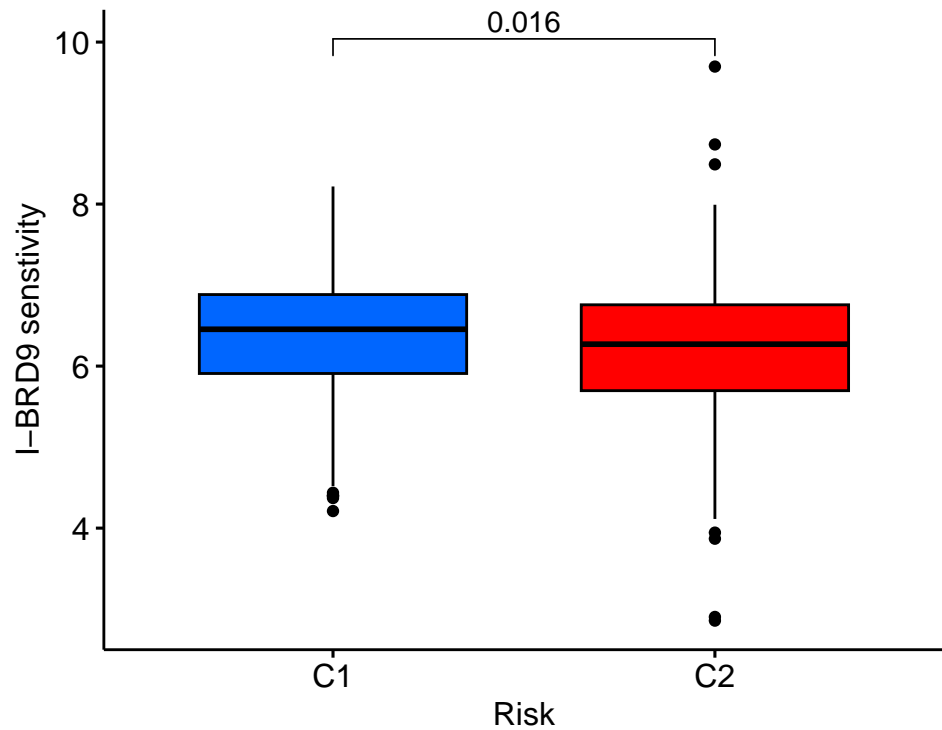

Supplement: Supplementary file 1 — Supplementary Information. [file 41598_2024_53257_MOESM1_ESM.zip › supplementary files/Drug sensitivity of C1 group and C2 group/C2 better/drugSenstivity.I-BRD9.pdf]

Risk C1 C2

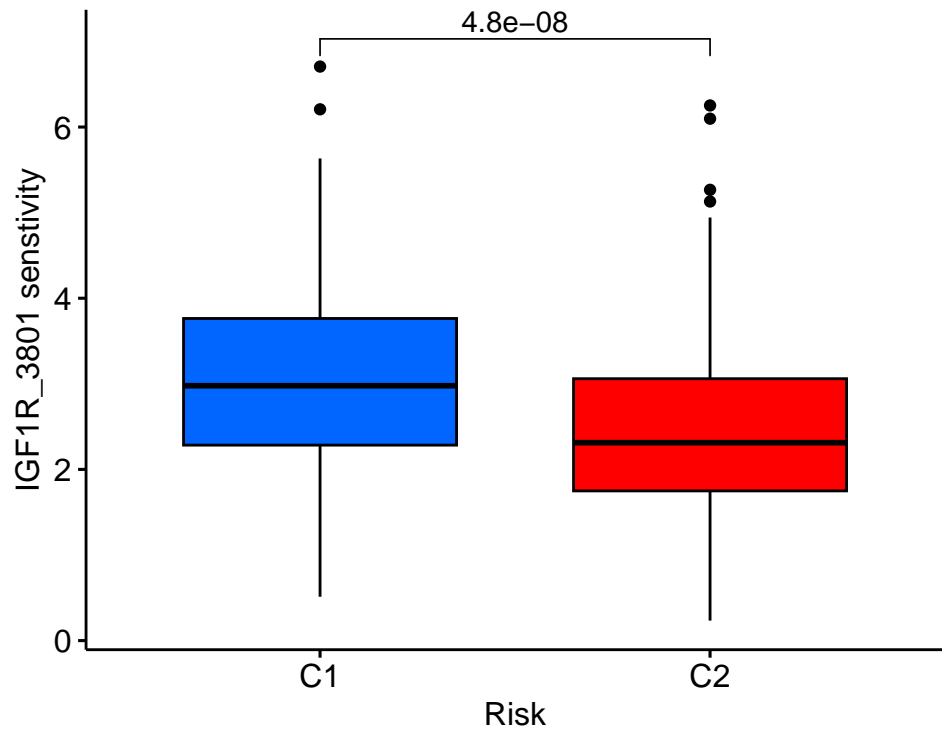

Supplement: Supplementary file 1 — Supplementary Information. [file 41598_2024_53257_MOESM1_ESM.zip › supplementary files/Drug sensitivity of C1 group and C2 group/C2 better/drugSenstivity.IGF1R_3801.pdf]

Risk C1 C2

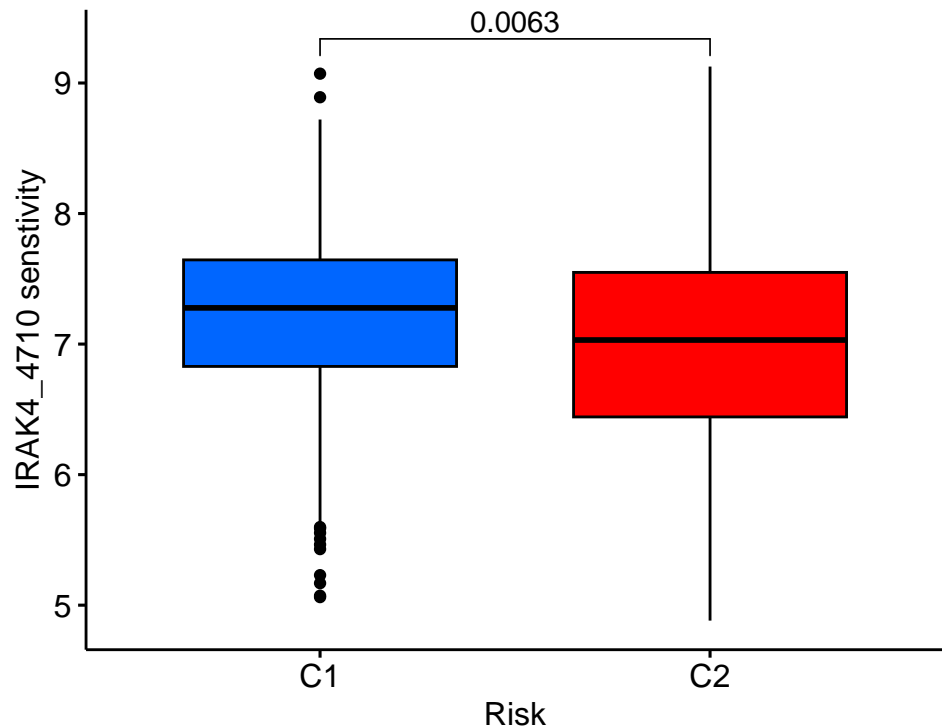

Supplement: Supplementary file 1 — Supplementary Information. [file 41598_2024_53257_MOESM1_ESM.zip › supplementary files/Drug sensitivity of C1 group and C2 group/C2 better/drugSenstivity.IRAK4_4710.pdf]

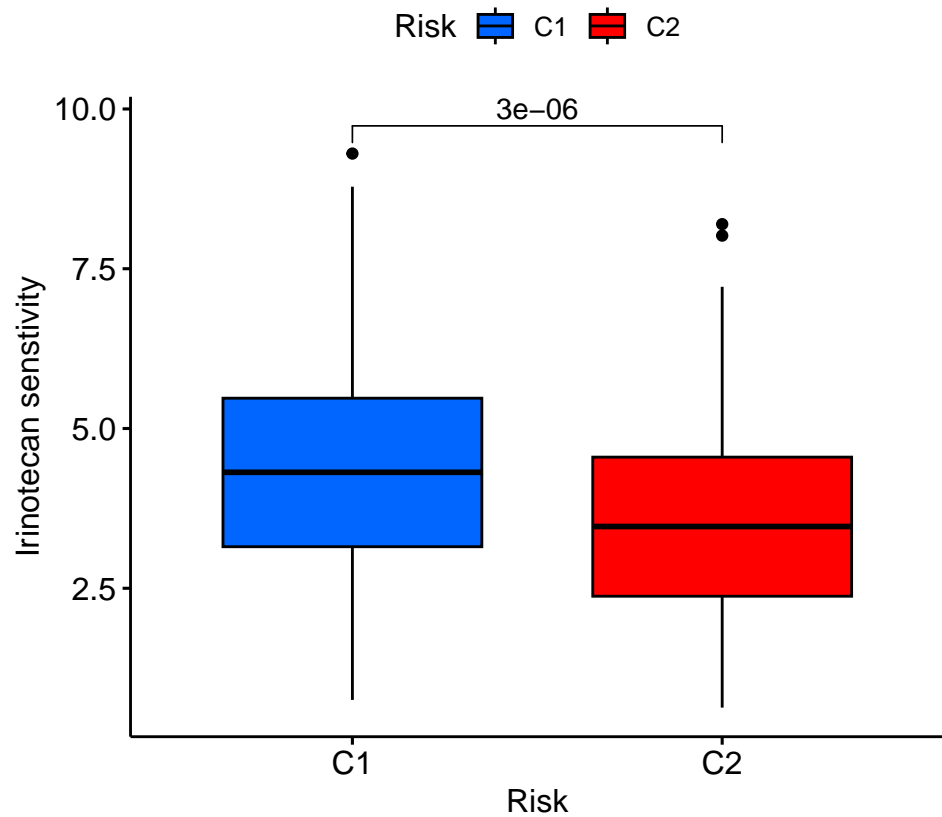

Supplement: Supplementary file 1 — Supplementary Information. [file 41598_2024_53257_MOESM1_ESM.zip › supplementary files/Drug sensitivity of C1 group and C2 group/C2 better/drugSenstivity.Irinotecan.pdf]

Risk C1 C2

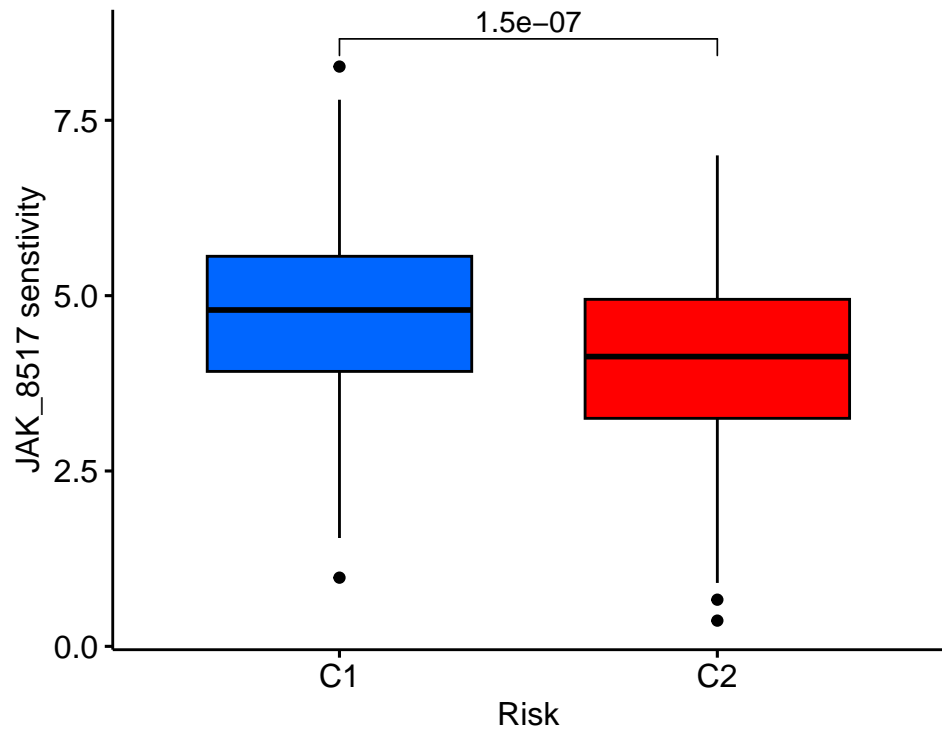

Supplement: Supplementary file 1 — Supplementary Information. [file 41598_2024_53257_MOESM1_ESM.zip › supplementary files/Drug sensitivity of C1 group and C2 group/C2 better/drugSenstivity.JAK_8517.pdf]

Risk C1 C2

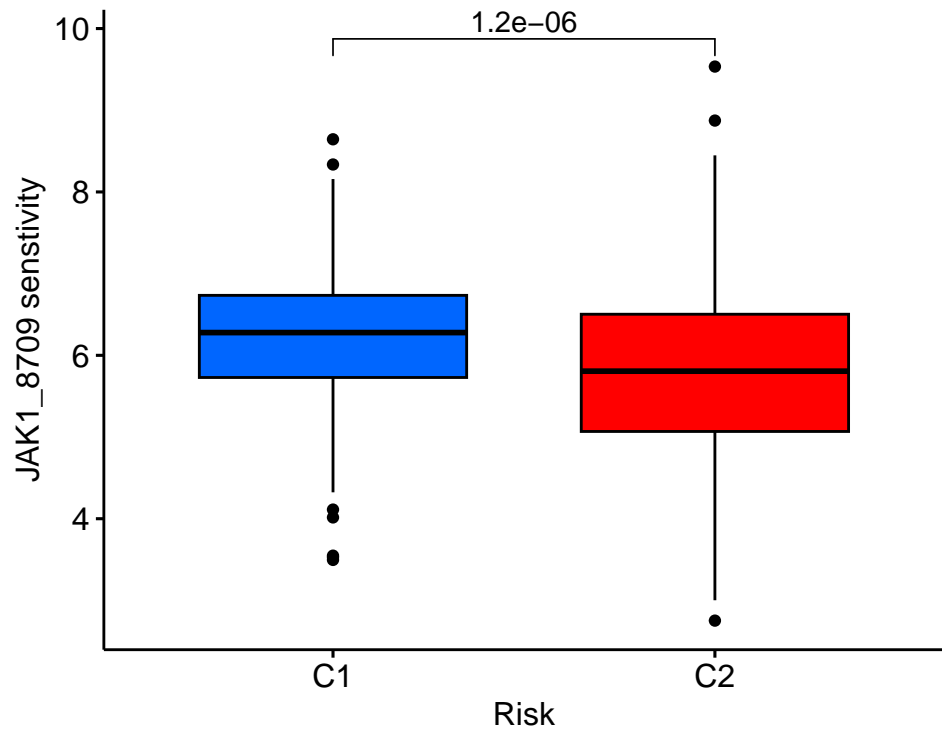

Supplement: Supplementary file 1 — Supplementary Information. [file 41598_2024_53257_MOESM1_ESM.zip › supplementary files/Drug sensitivity of C1 group and C2 group/C2 better/drugSenstivity.JAK1_8709.pdf]

Risk C1 C2

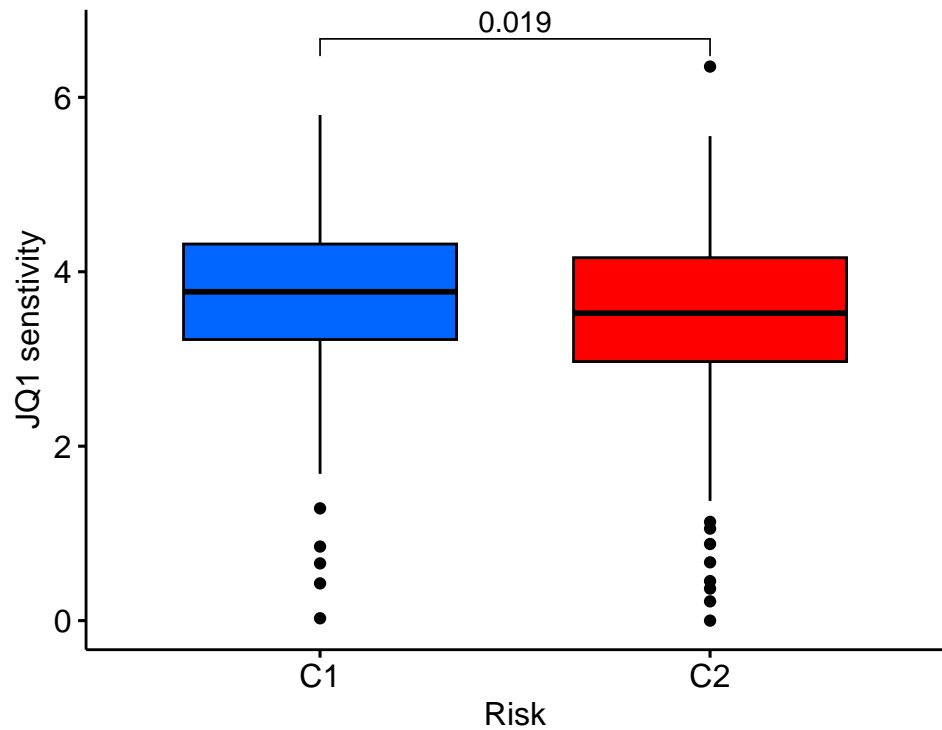

Supplement: Supplementary file 1 — Supplementary Information. [file 41598_2024_53257_MOESM1_ESM.zip › supplementary files/Drug sensitivity of C1 group and C2 group/C2 better/drugSenstivity.JQ1.pdf]

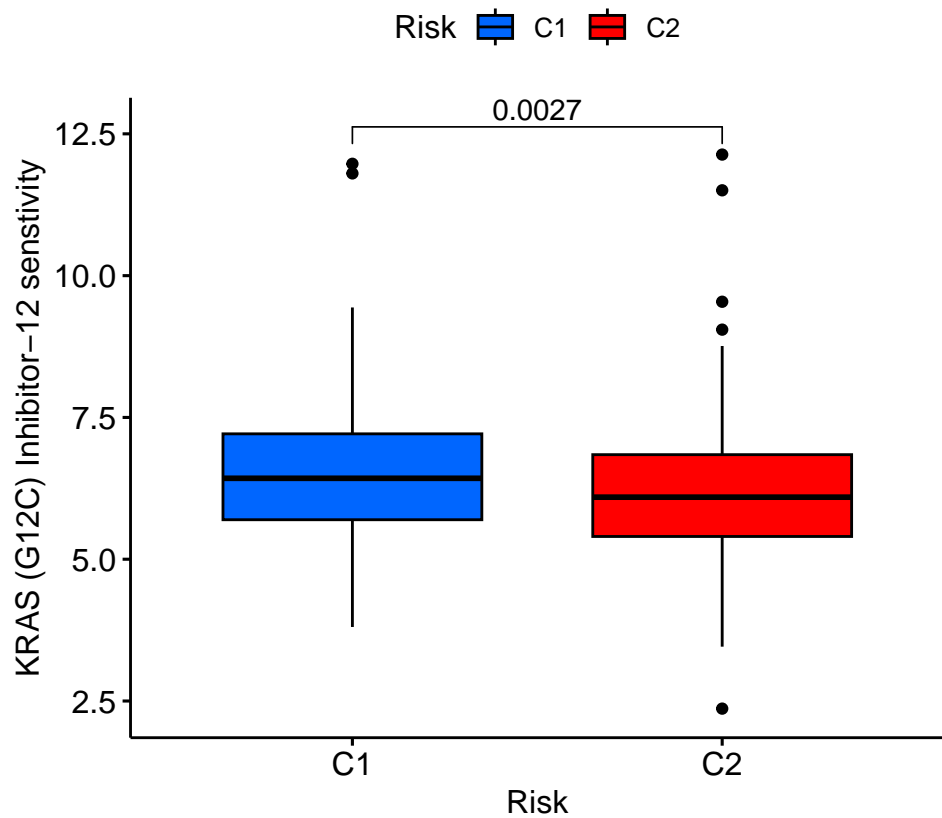

Supplement: Supplementary file 1 — Supplementary Information. [file 41598_2024_53257_MOESM1_ESM.zip › supplementary files/Drug sensitivity of C1 group and C2 group/C2 better/drugSenstivity.KRAS (G12C) Inhibitor-12.pdf]

Risk C1 C2

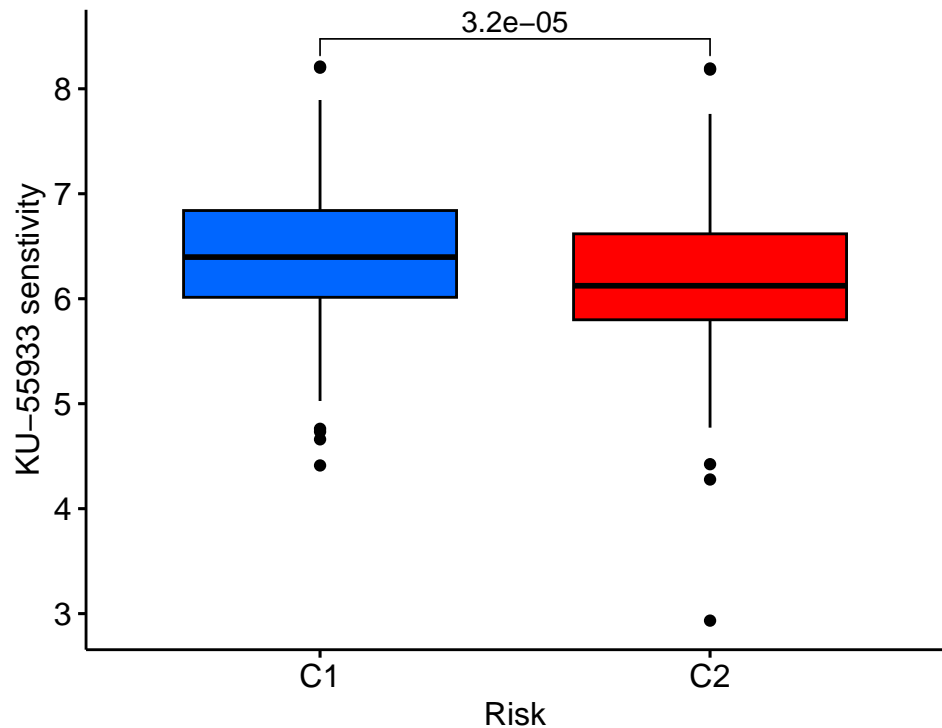

Supplement: Supplementary file 1 — Supplementary Information. [file 41598_2024_53257_MOESM1_ESM.zip › supplementary files/Drug sensitivity of C1 group and C2 group/C2 better/drugSenstivity.KU-55933.pdf]

Risk C1 C2

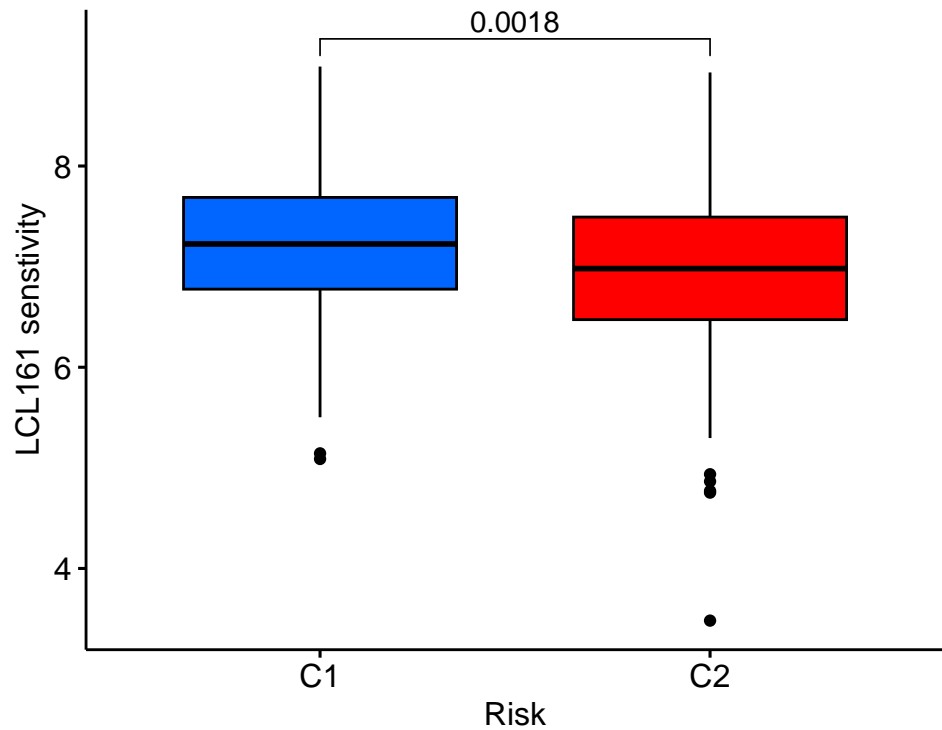

Supplement: Supplementary file 1 — Supplementary Information. [file 41598_2024_53257_MOESM1_ESM.zip › supplementary files/Drug sensitivity of C1 group and C2 group/C2 better/drugSenstivity.LCL161.pdf]

Risk C1 C2

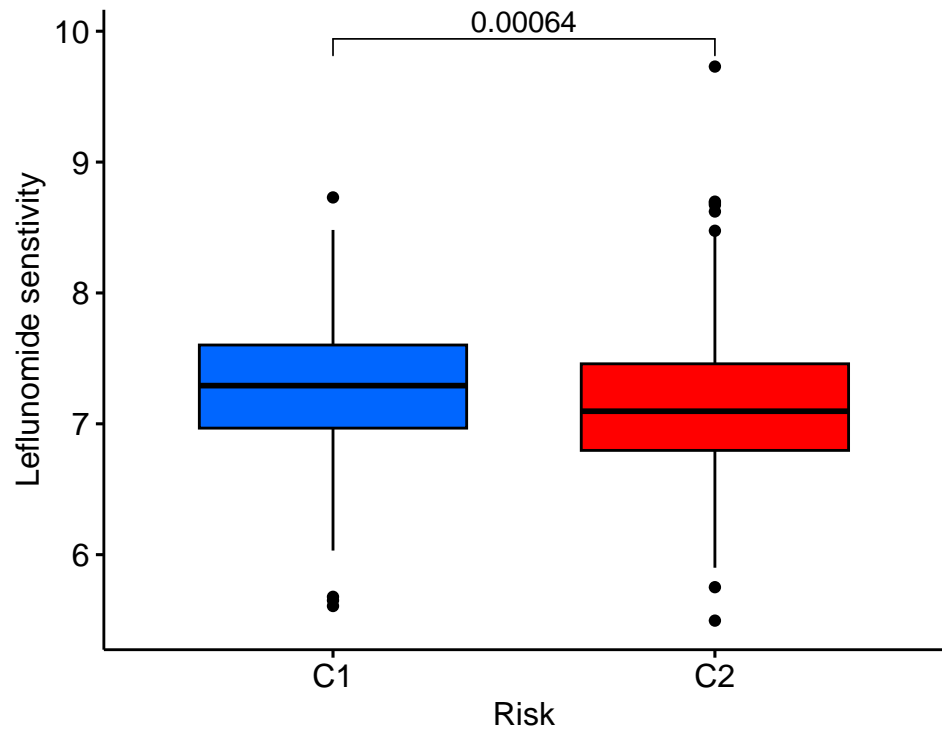

Supplement: Supplementary file 1 — Supplementary Information. [file 41598_2024_53257_MOESM1_ESM.zip › supplementary files/Drug sensitivity of C1 group and C2 group/C2 better/drugSenstivity.Leflunomide.pdf]

LJ1308 sensitivity

Risk C1 C2

0.0031

C1

C2

Risk

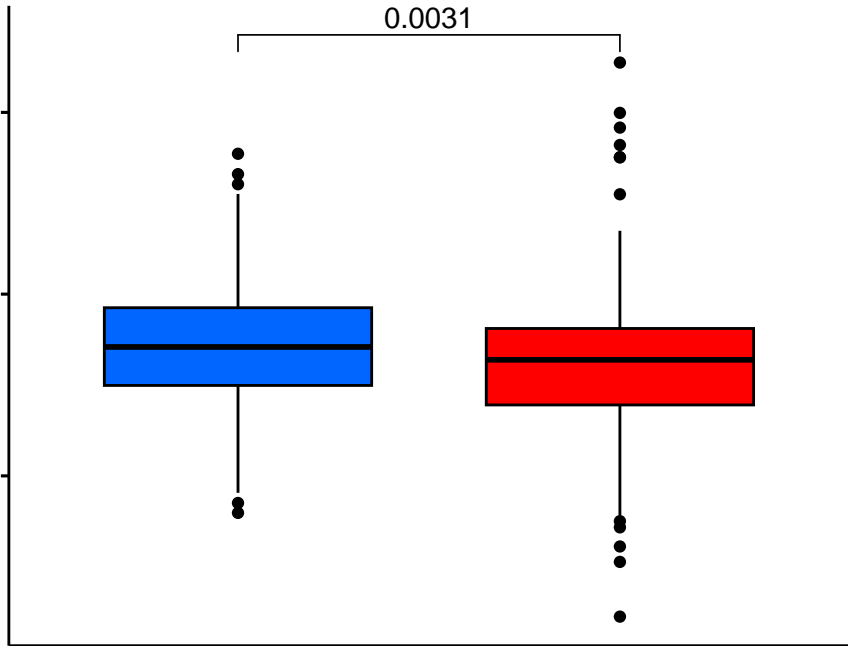

Supplement: Supplementary file 1 — Supplementary Information. [file 41598_2024_53257_MOESM1_ESM.zip › supplementary files/Drug sensitivity of C1 group and C2 group/C2 better/drugSenstivity.LJI308.pdf]

Risk C1 C2

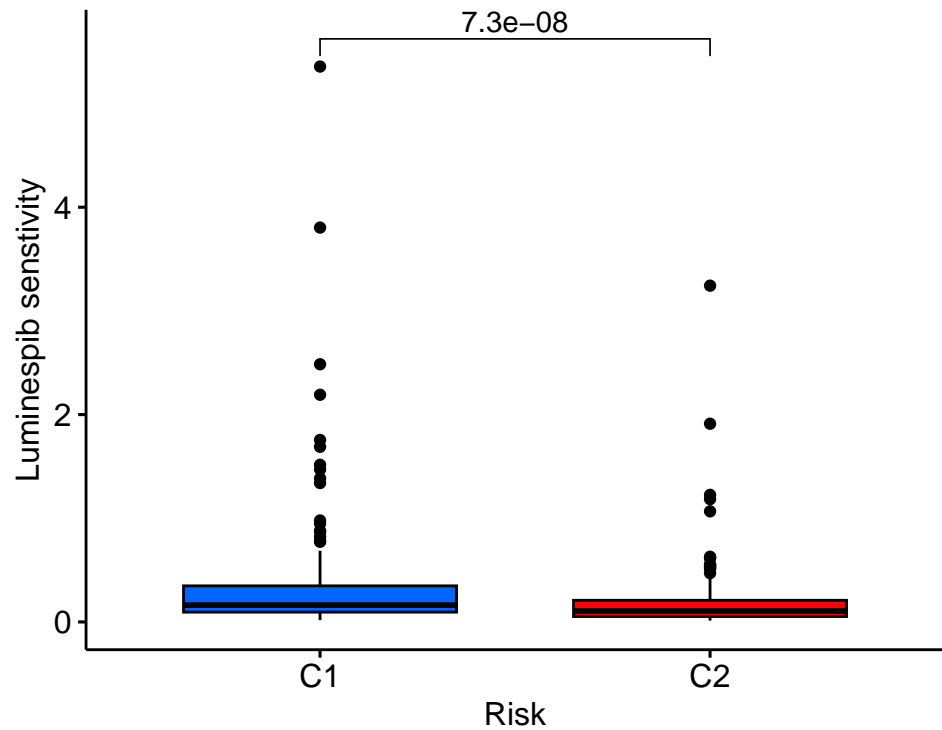

Supplement: Supplementary file 1 — Supplementary Information. [file 41598_2024_53257_MOESM1_ESM.zip › supplementary files/Drug sensitivity of C1 group and C2 group/C2 better/drugSenstivity.Luminespib.pdf]

Risk C1 C2

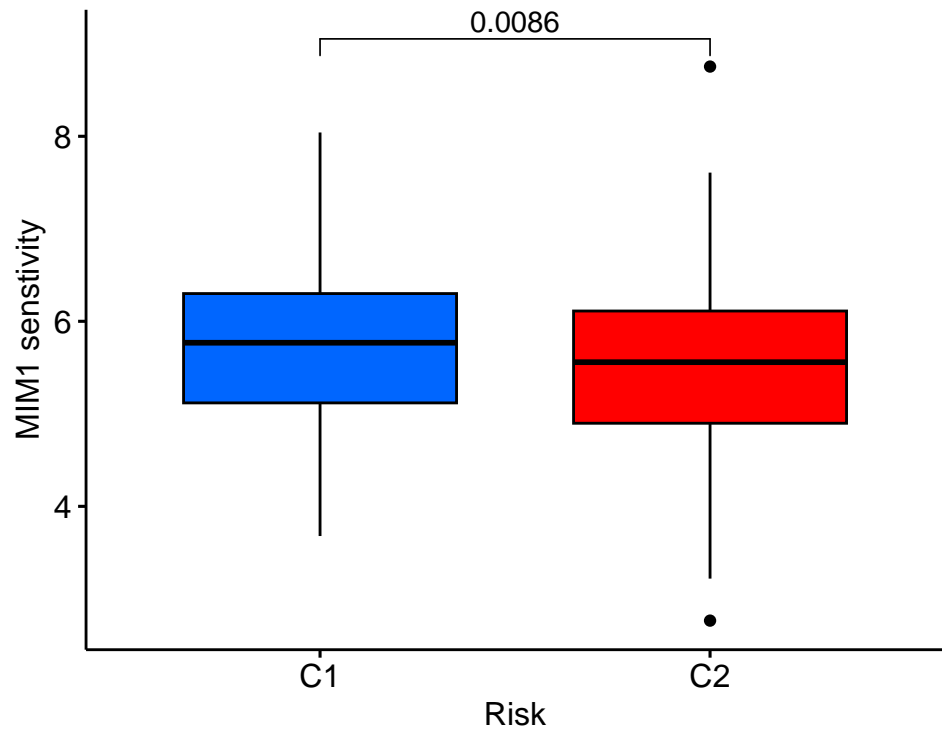

Supplement: Supplementary file 1 — Supplementary Information. [file 41598_2024_53257_MOESM1_ESM.zip › supplementary files/Drug sensitivity of C1 group and C2 group/C2 better/drugSenstivity.MIM1.pdf]

Risk C1 C2

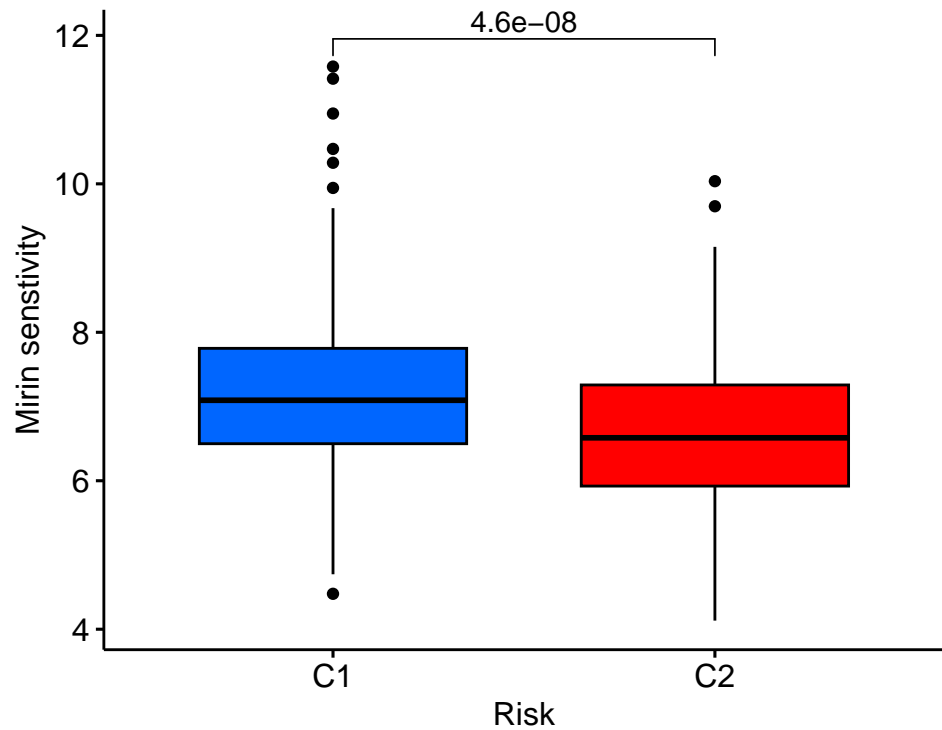

Supplement: Supplementary file 1 — Supplementary Information. [file 41598_2024_53257_MOESM1_ESM.zip › supplementary files/Drug sensitivity of C1 group and C2 group/C2 better/drugSenstivity.Mirin.pdf]

Risk C1 C2

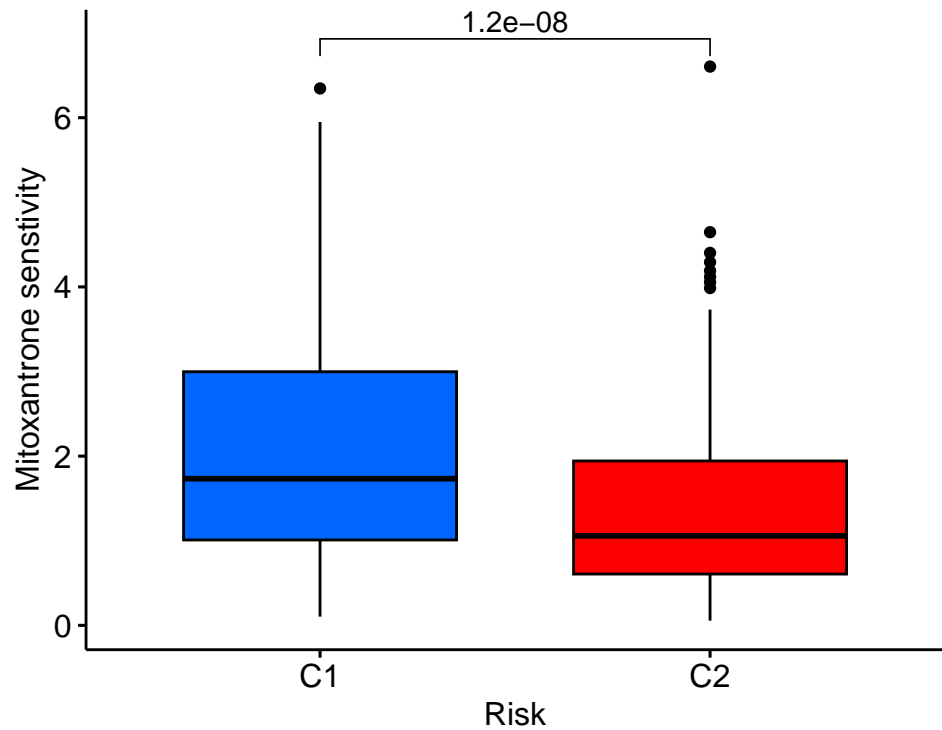

Supplement: Supplementary file 1 — Supplementary Information. [file 41598_2024_53257_MOESM1_ESM.zip › supplementary files/Drug sensitivity of C1 group and C2 group/C2 better/drugSenstivity.Mitoxantrone.pdf]

Risk C1 C2

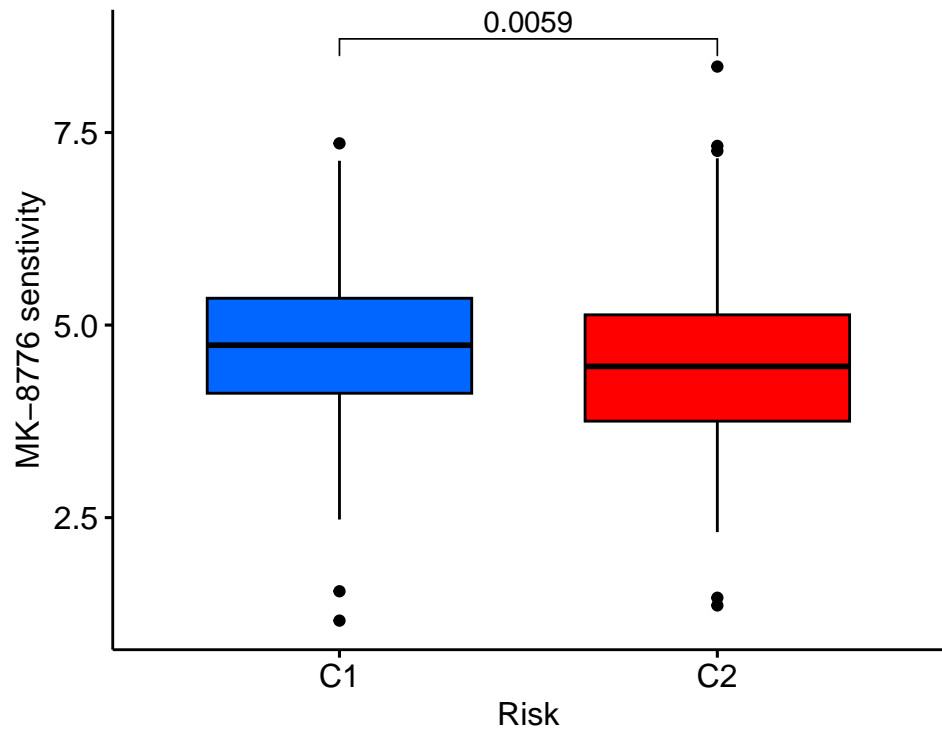

Supplement: Supplementary file 1 — Supplementary Information. [file 41598_2024_53257_MOESM1_ESM.zip › supplementary files/Drug sensitivity of C1 group and C2 group/C2 better/drugSenstivity.MK-8776.pdf]

Risk C1 C2

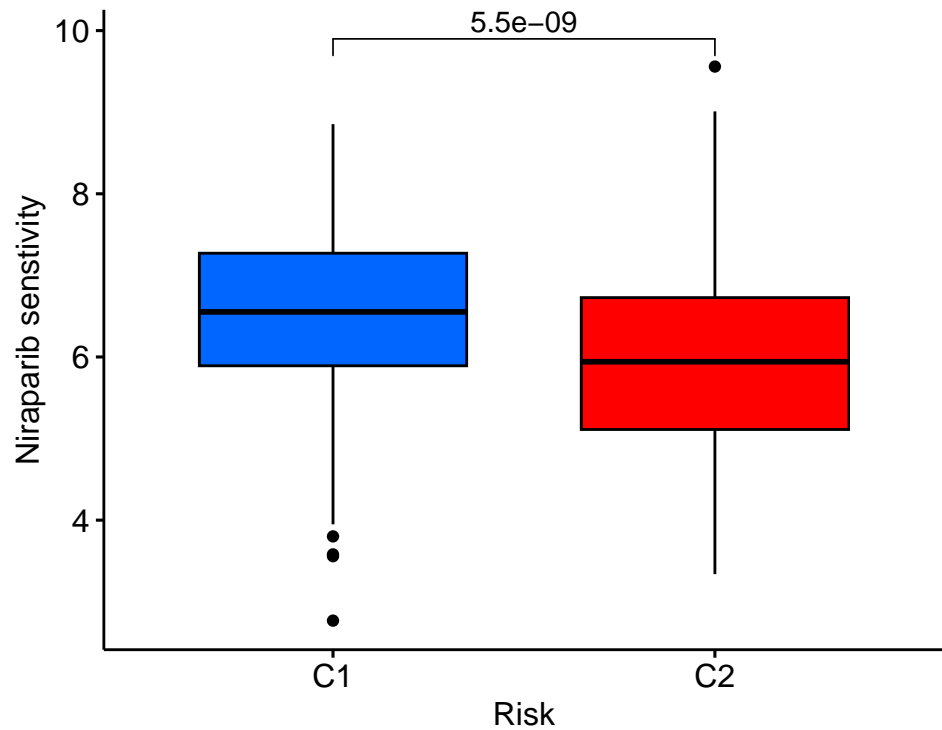

Supplement: Supplementary file 1 — Supplementary Information. [file 41598_2024_53257_MOESM1_ESM.zip › supplementary files/Drug sensitivity of C1 group and C2 group/C2 better/drugSenstivity.Niraparib.pdf]

Risk C1 C2

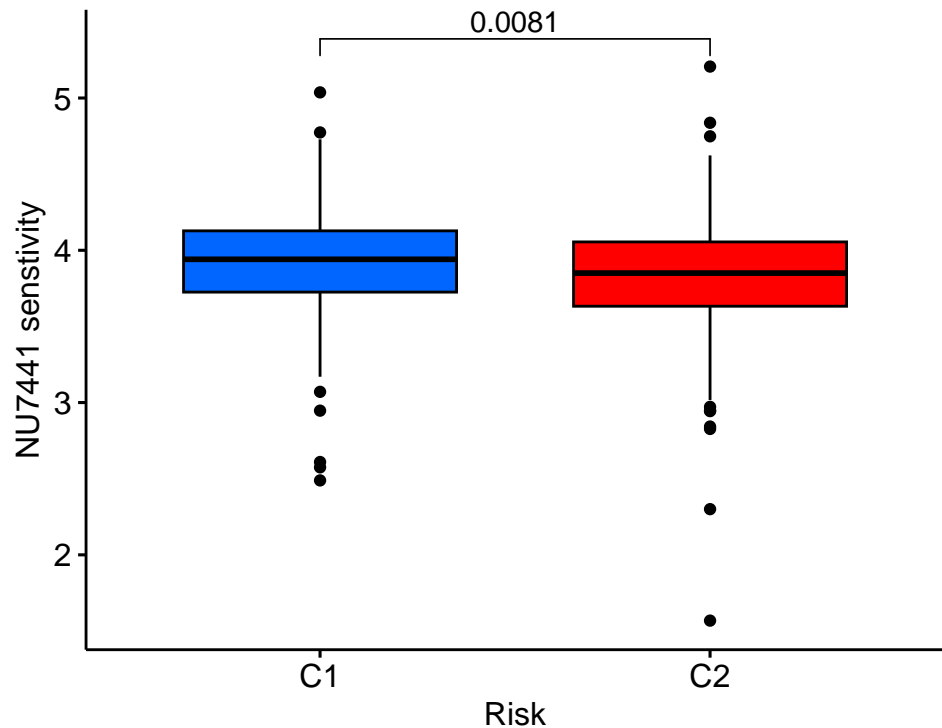

Supplement: Supplementary file 1 — Supplementary Information. [file 41598_2024_53257_MOESM1_ESM.zip › supplementary files/Drug sensitivity of C1 group and C2 group/C2 better/drugSenstivity.NU7441.pdf]

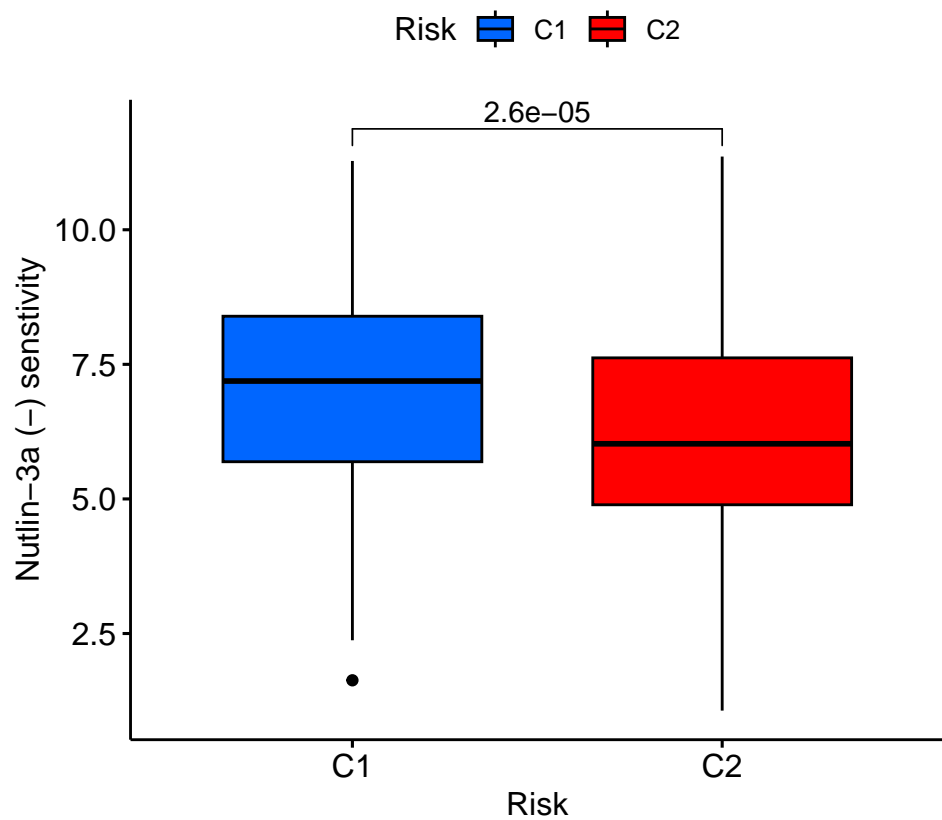

Supplement: Supplementary file 1 — Supplementary Information. [file 41598_2024_53257_MOESM1_ESM.zip › supplementary files/Drug sensitivity of C1 group and C2 group/C2 better/drugSenstivity.Nutlin-3a (-).pdf]

Risk C1 C2

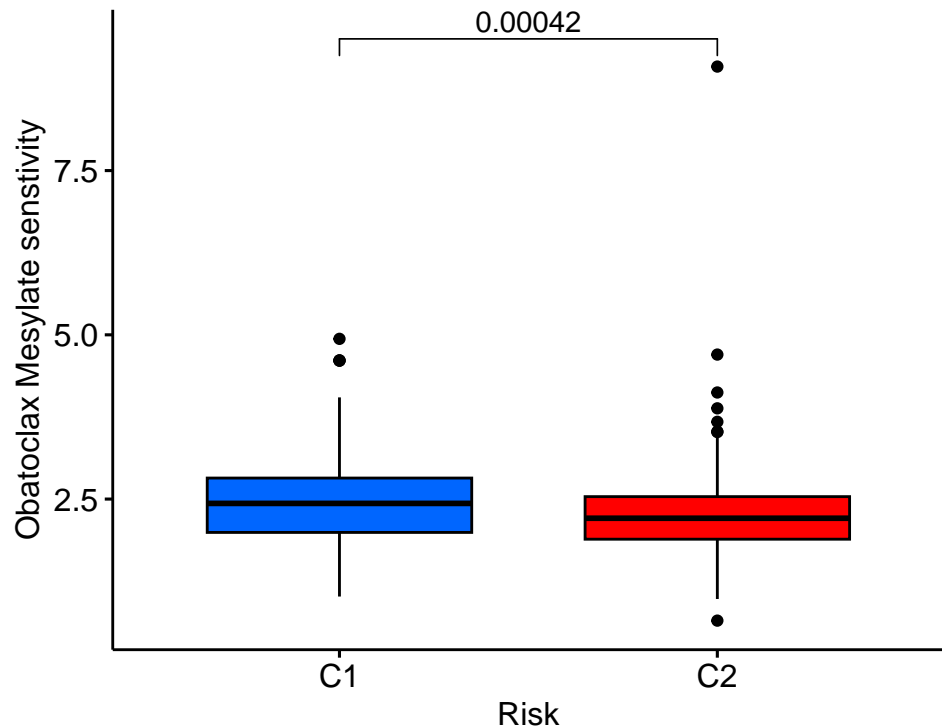

Supplement: Supplementary file 1 — Supplementary Information. [file 41598_2024_53257_MOESM1_ESM.zip › supplementary files/Drug sensitivity of C1 group and C2 group/C2 better/drugSenstivity.Obatoclax Mesylate.pdf]

Risk C1 C2

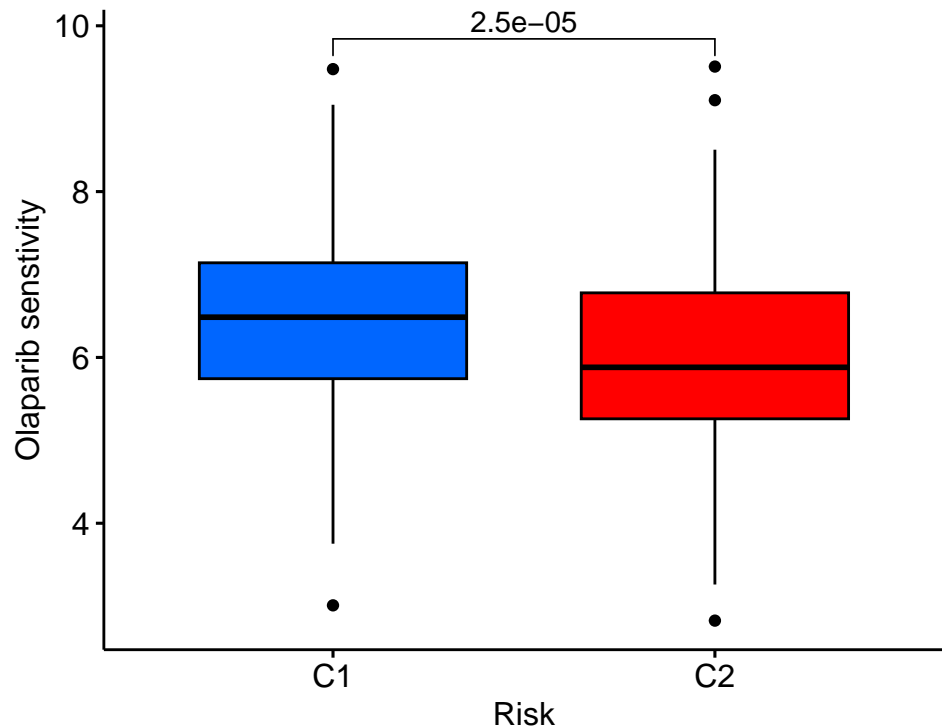

Supplement: Supplementary file 1 — Supplementary Information. [file 41598_2024_53257_MOESM1_ESM.zip › supplementary files/Drug sensitivity of C1 group and C2 group/C2 better/drugSenstivity.Olaparib.pdf]

Risk C1 C2

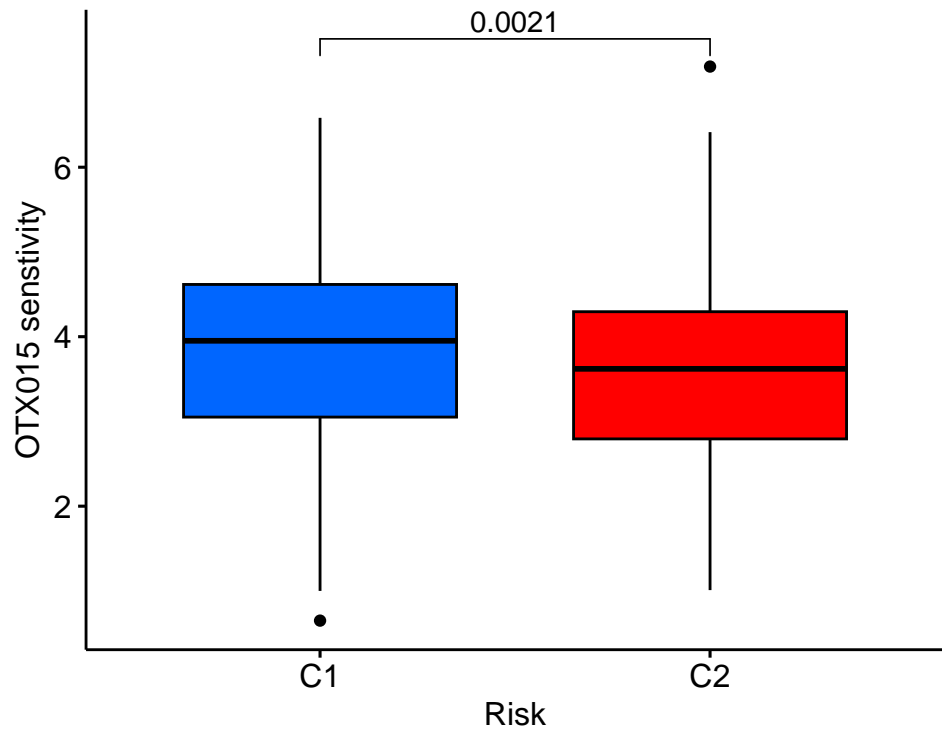

Supplement: Supplementary file 1 — Supplementary Information. [file 41598_2024_53257_MOESM1_ESM.zip › supplementary files/Drug sensitivity of C1 group and C2 group/C2 better/drugSenstivity.OTX015.pdf]

Risk C1 C2

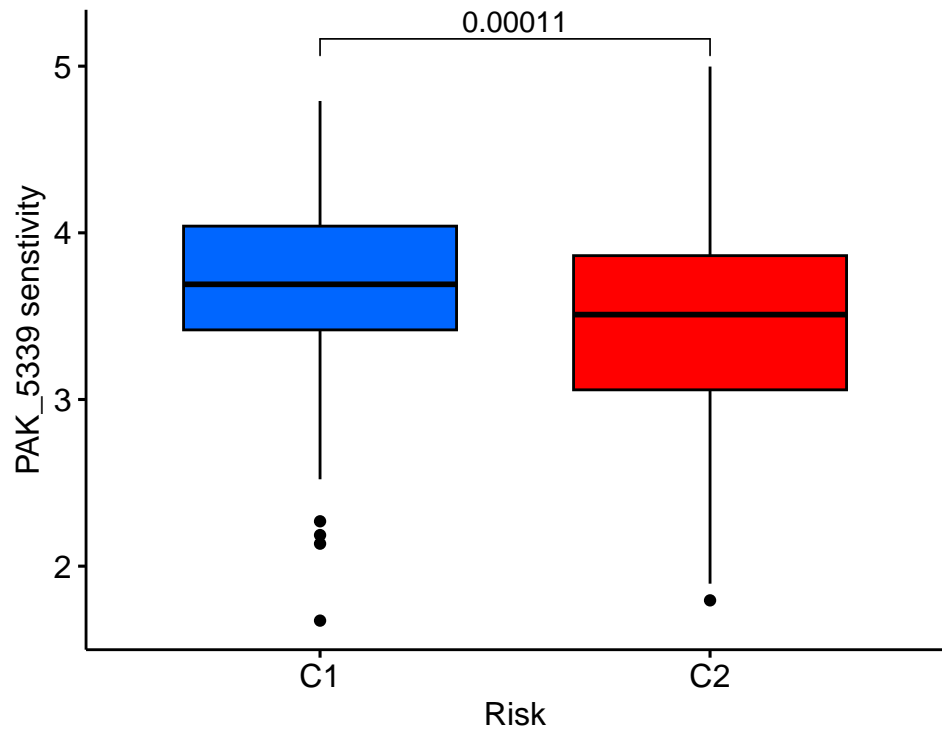

Supplement: Supplementary file 1 — Supplementary Information. [file 41598_2024_53257_MOESM1_ESM.zip › supplementary files/Drug sensitivity of C1 group and C2 group/C2 better/drugSenstivity.PAK_5339.pdf]

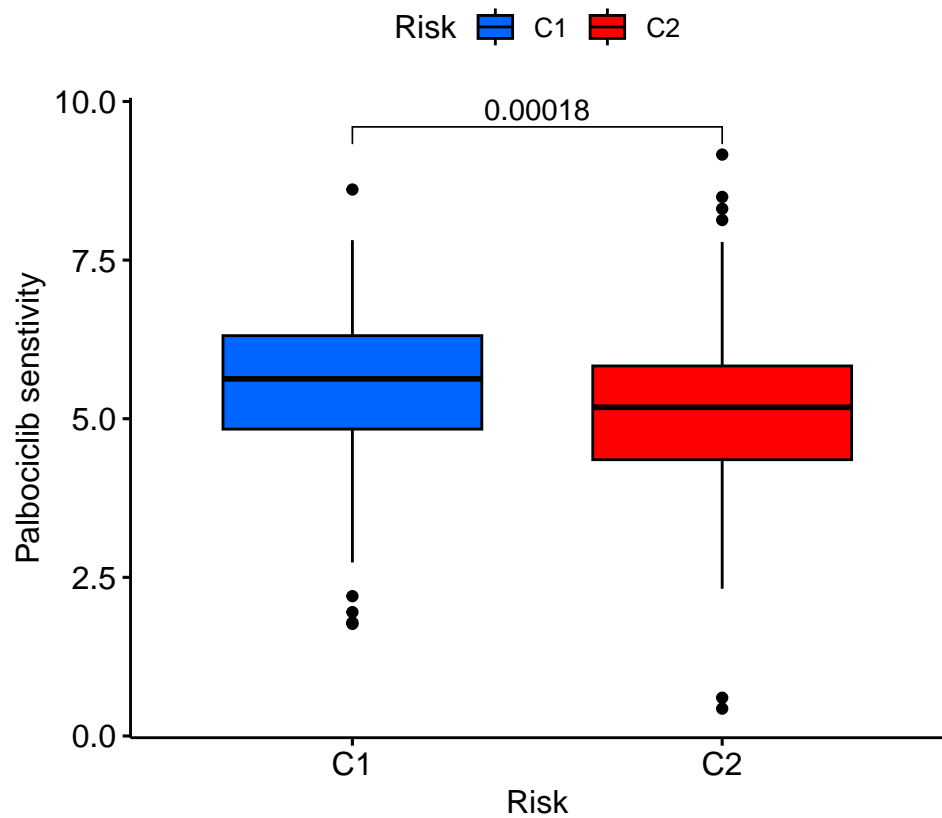

Supplement: Supplementary file 1 — Supplementary Information. [file 41598_2024_53257_MOESM1_ESM.zip › supplementary files/Drug sensitivity of C1 group and C2 group/C2 better/drugSenstivity.Palbociclib.pdf]

Risk C1 C2

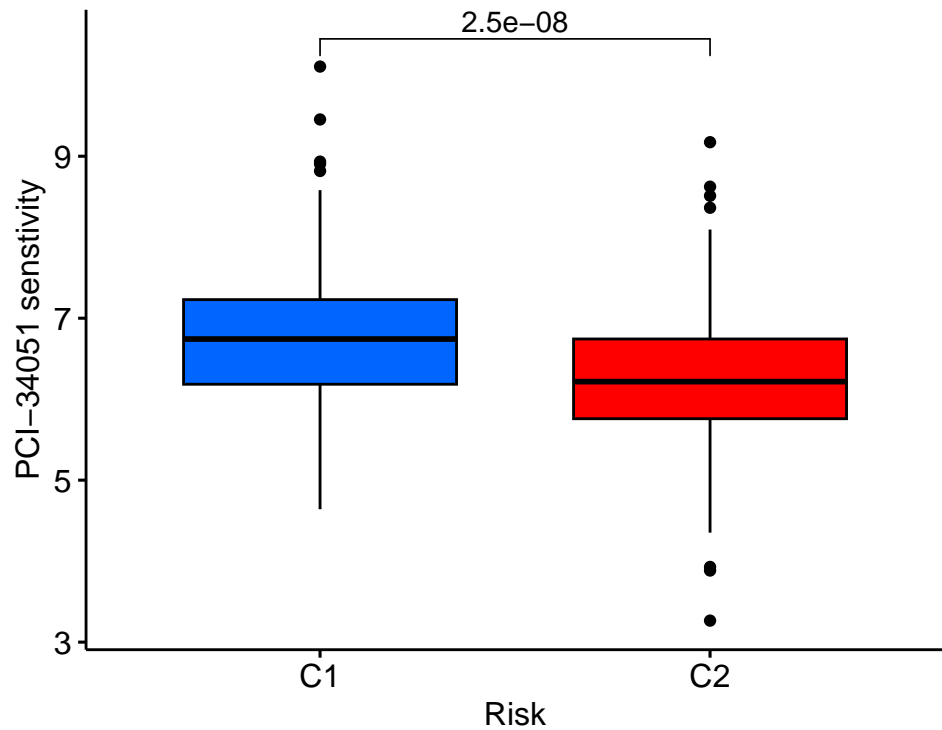

Supplement: Supplementary file 1 — Supplementary Information. [file 41598_2024_53257_MOESM1_ESM.zip › supplementary files/Drug sensitivity of C1 group and C2 group/C2 better/drugSenstivity.PCI-34051.pdf]

Risk C1 C2

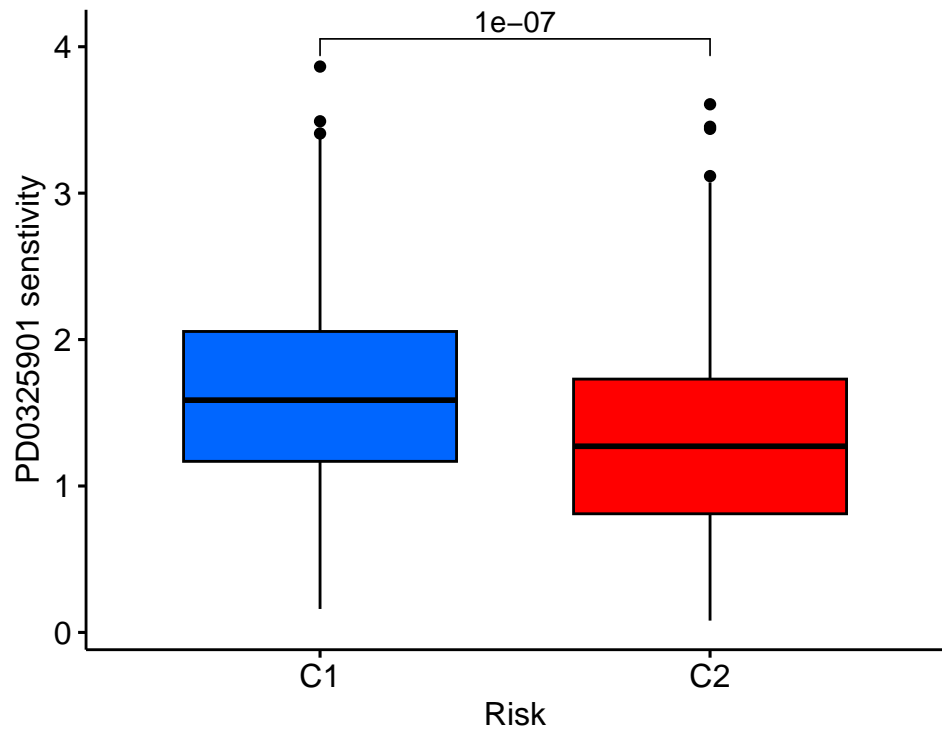

Supplement: Supplementary file 1 — Supplementary Information. [file 41598_2024_53257_MOESM1_ESM.zip › supplementary files/Drug sensitivity of C1 group and C2 group/C2 better/drugSenstivity.PD0325901.pdf]

Risk C1 C2

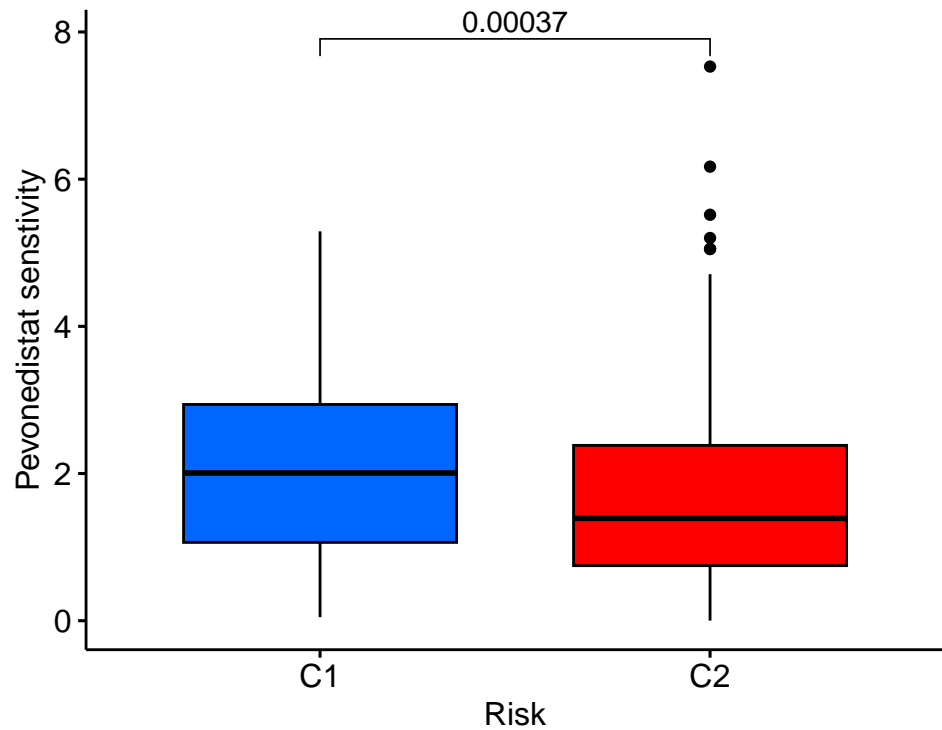

Supplement: Supplementary file 1 — Supplementary Information. [file 41598_2024_53257_MOESM1_ESM.zip › supplementary files/Drug sensitivity of C1 group and C2 group/C2 better/drugSenstivity.Pevonedistat.pdf]

Risk C1 C2

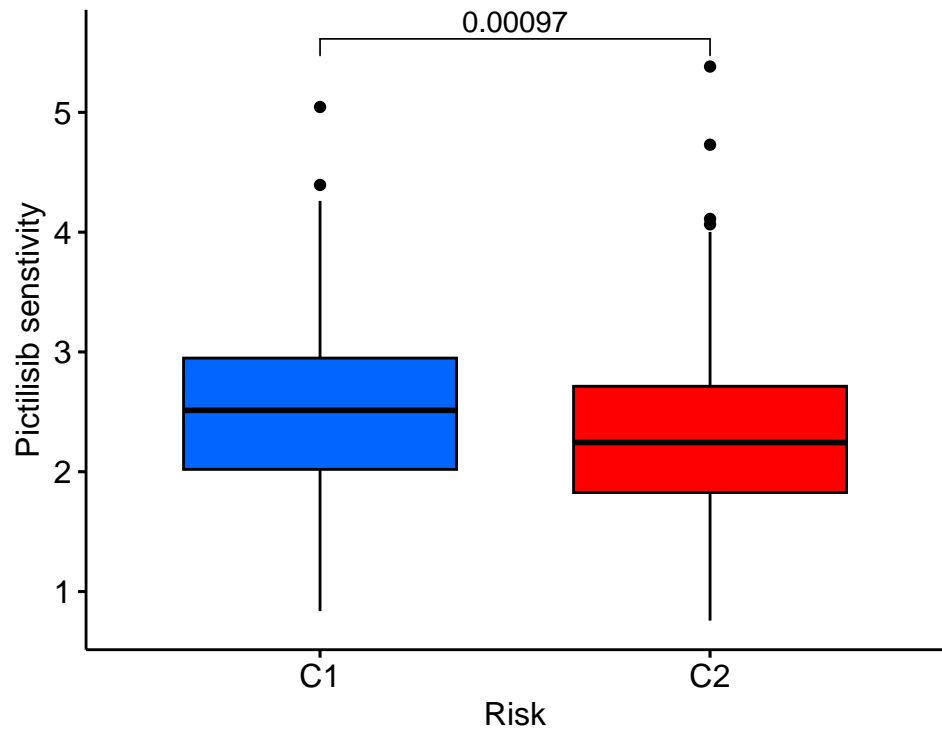

Supplement: Supplementary file 1 — Supplementary Information. [file 41598_2024_53257_MOESM1_ESM.zip › supplementary files/Drug sensitivity of C1 group and C2 group/C2 better/drugSenstivity.Pictilisib.pdf]

Risk C1 C2

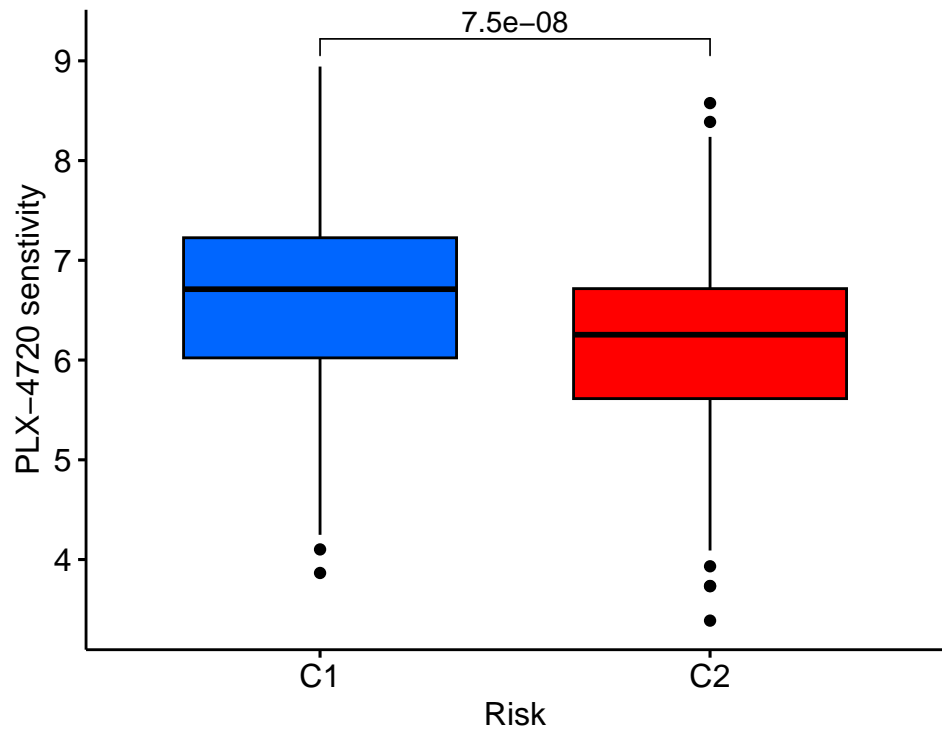

Supplement: Supplementary file 1 — Supplementary Information. [file 41598_2024_53257_MOESM1_ESM.zip › supplementary files/Drug sensitivity of C1 group and C2 group/C2 better/drugSenstivity.PLX-4720.pdf]

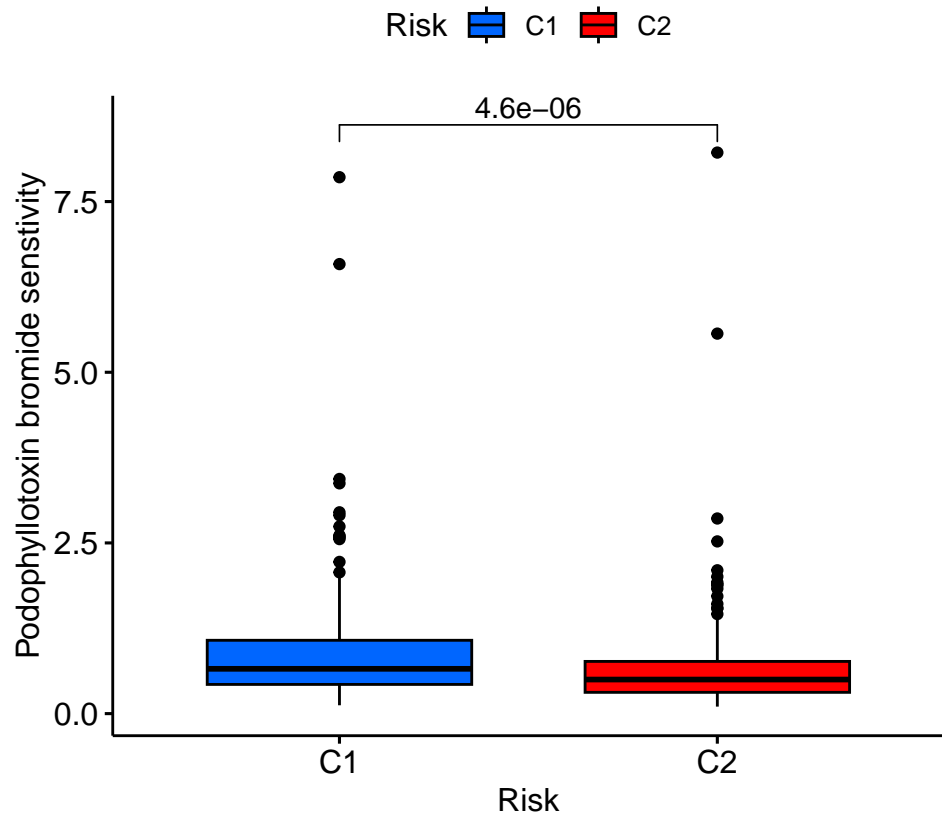

Supplement: Supplementary file 1 — Supplementary Information. [file 41598_2024_53257_MOESM1_ESM.zip › supplementary files/Drug sensitivity of C1 group and C2 group/C2 better/drugSenstivity.Podophyllotoxin bromide.pdf]

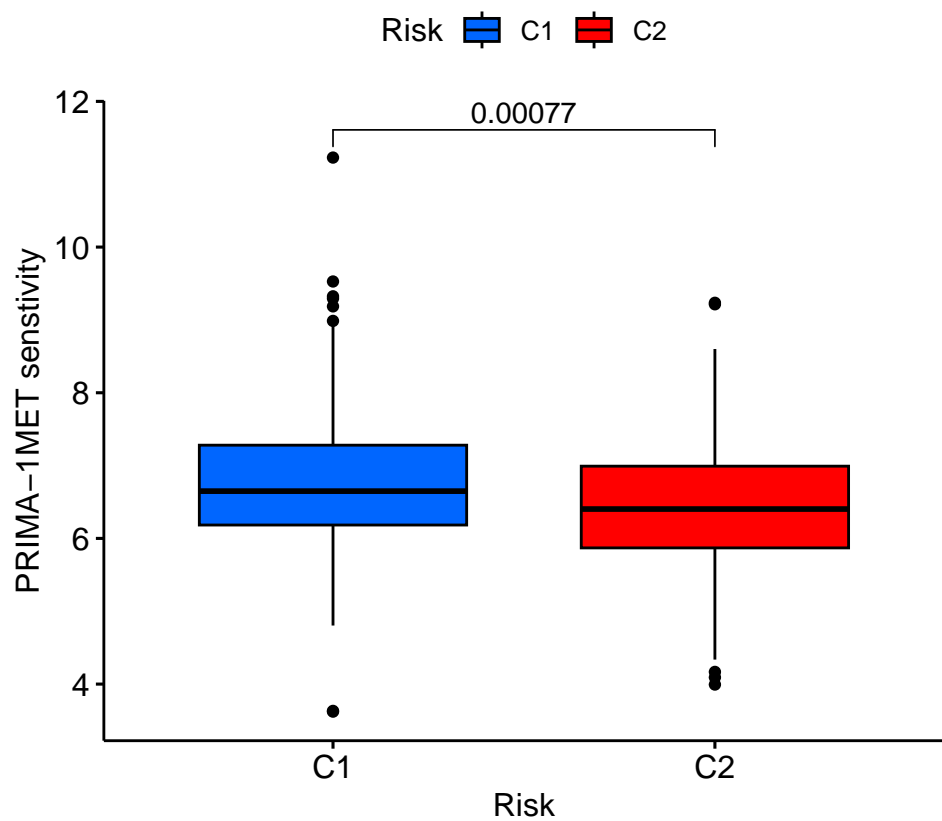

Supplement: Supplementary file 1 — Supplementary Information. [file 41598_2024_53257_MOESM1_ESM.zip › supplementary files/Drug sensitivity of C1 group and C2 group/C2 better/drugSenstivity.PRIMA-1MET.pdf]

Risk C1 C2

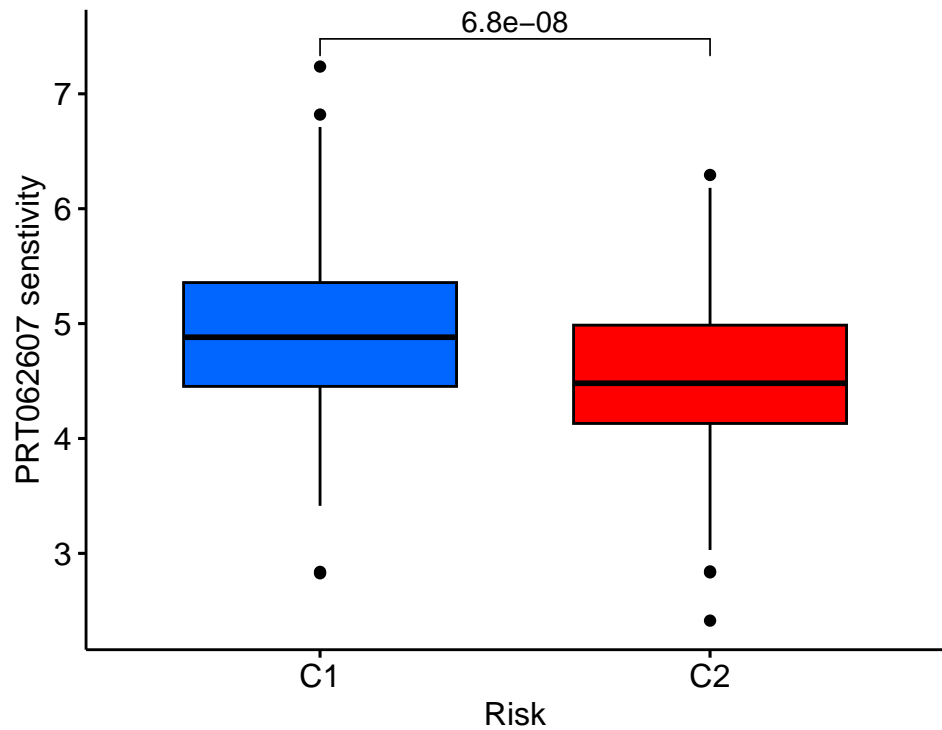

Supplement: Supplementary file 1 — Supplementary Information. [file 41598_2024_53257_MOESM1_ESM.zip › supplementary files/Drug sensitivity of C1 group and C2 group/C2 better/drugSenstivity.PRT062607.pdf]

Risk C1 C2

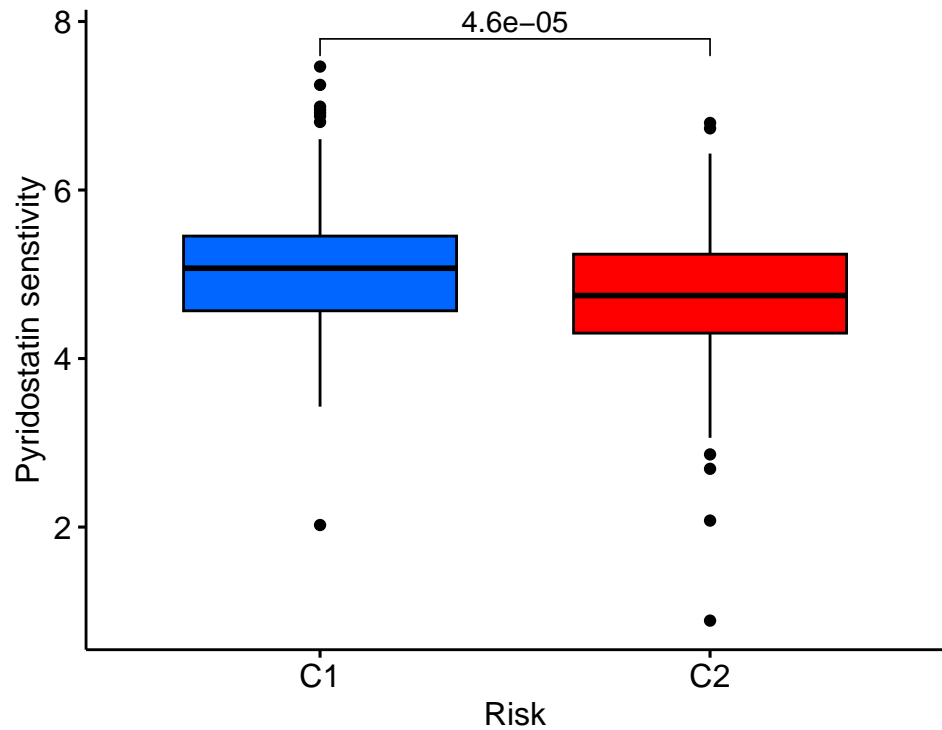

Supplement: Supplementary file 1 — Supplementary Information. [file 41598_2024_53257_MOESM1_ESM.zip › supplementary files/Drug sensitivity of C1 group and C2 group/C2 better/drugSenstivity.Pyridostatin.pdf]

Risk C1 C2

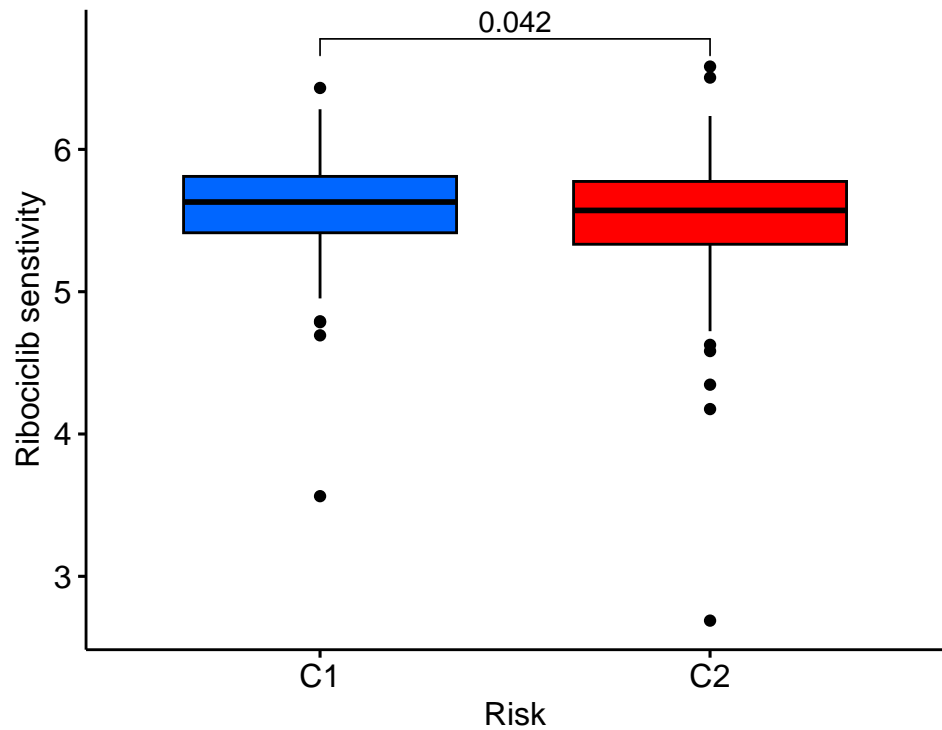

Supplement: Supplementary file 1 — Supplementary Information. [file 41598_2024_53257_MOESM1_ESM.zip › supplementary files/Drug sensitivity of C1 group and C2 group/C2 better/drugSenstivity.Ribociclib.pdf]

Risk C1 C2

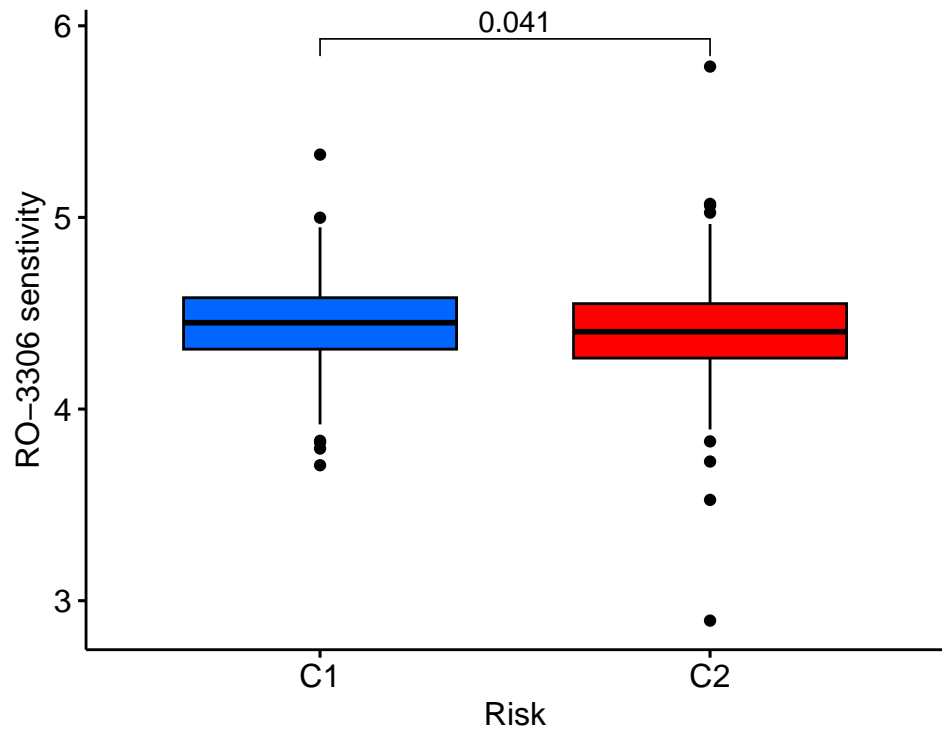

Supplement: Supplementary file 1 — Supplementary Information. [file 41598_2024_53257_MOESM1_ESM.zip › supplementary files/Drug sensitivity of C1 group and C2 group/C2 better/drugSenstivity.RO-3306.pdf]

Risk C1 C2

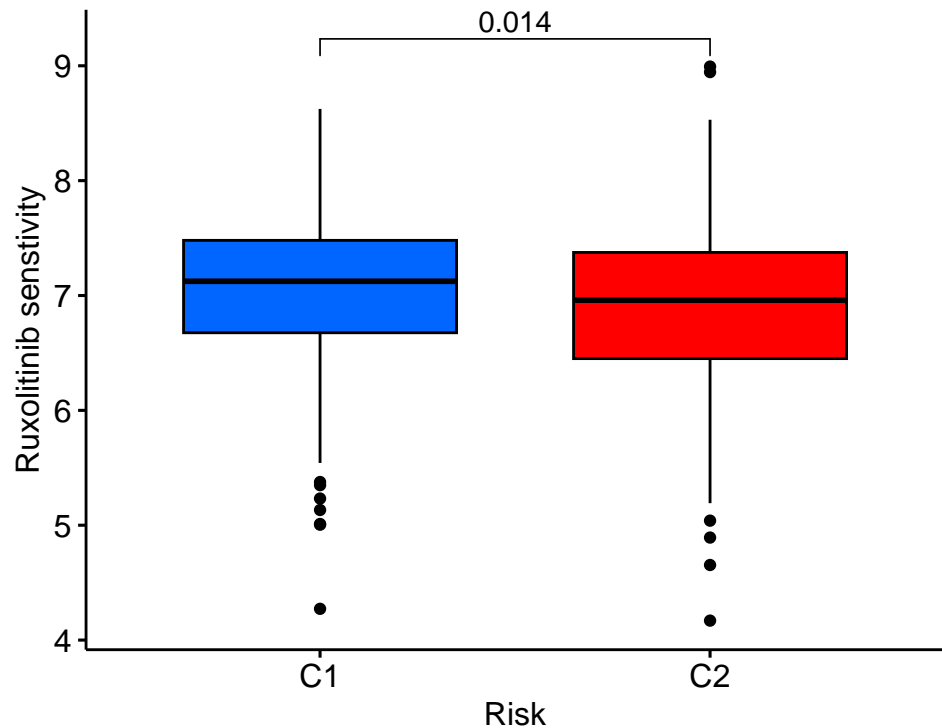

Supplement: Supplementary file 1 — Supplementary Information. [file 41598_2024_53257_MOESM1_ESM.zip › supplementary files/Drug sensitivity of C1 group and C2 group/C2 better/drugSenstivity.Ruxolitinib.pdf]

Risk C1 C2

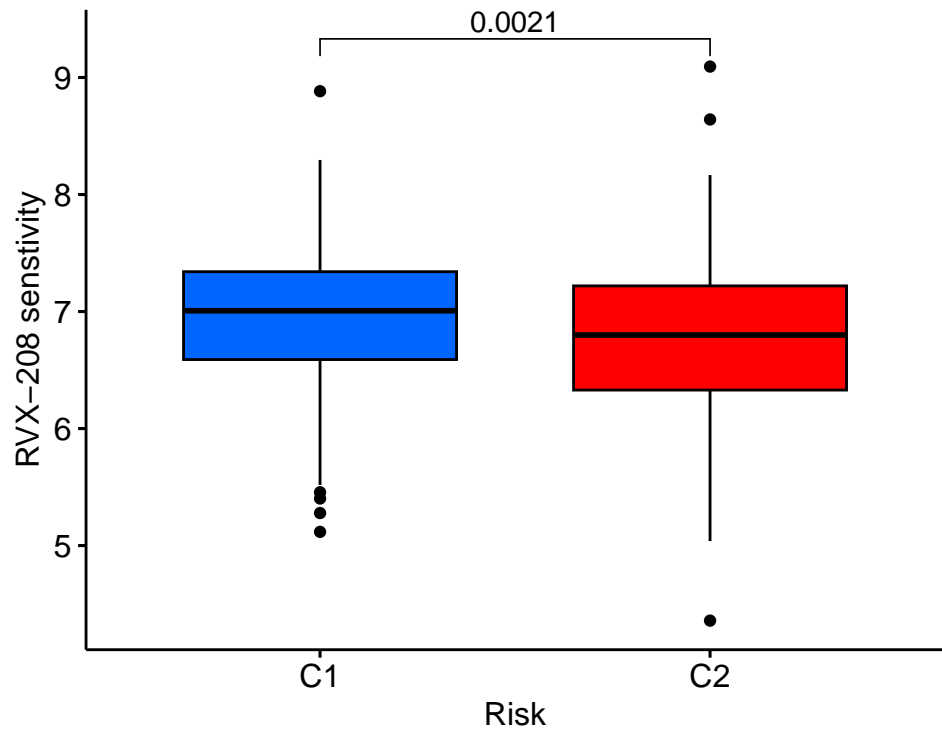

Supplement: Supplementary file 1 — Supplementary Information. [file 41598_2024_53257_MOESM1_ESM.zip › supplementary files/Drug sensitivity of C1 group and C2 group/C2 better/drugSenstivity.RVX-208.pdf]

Risk C1 C2

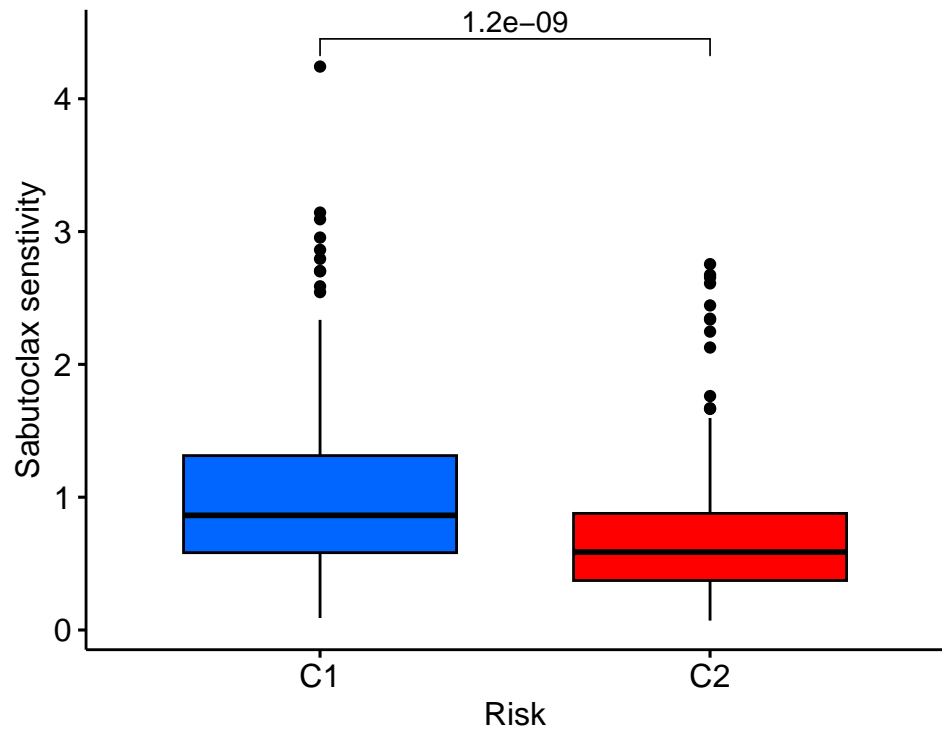

Supplement: Supplementary file 1 — Supplementary Information. [file 41598_2024_53257_MOESM1_ESM.zip › supplementary files/Drug sensitivity of C1 group and C2 group/C2 better/drugSenstivity.Sabutoclax.pdf]

Risk C1 C2

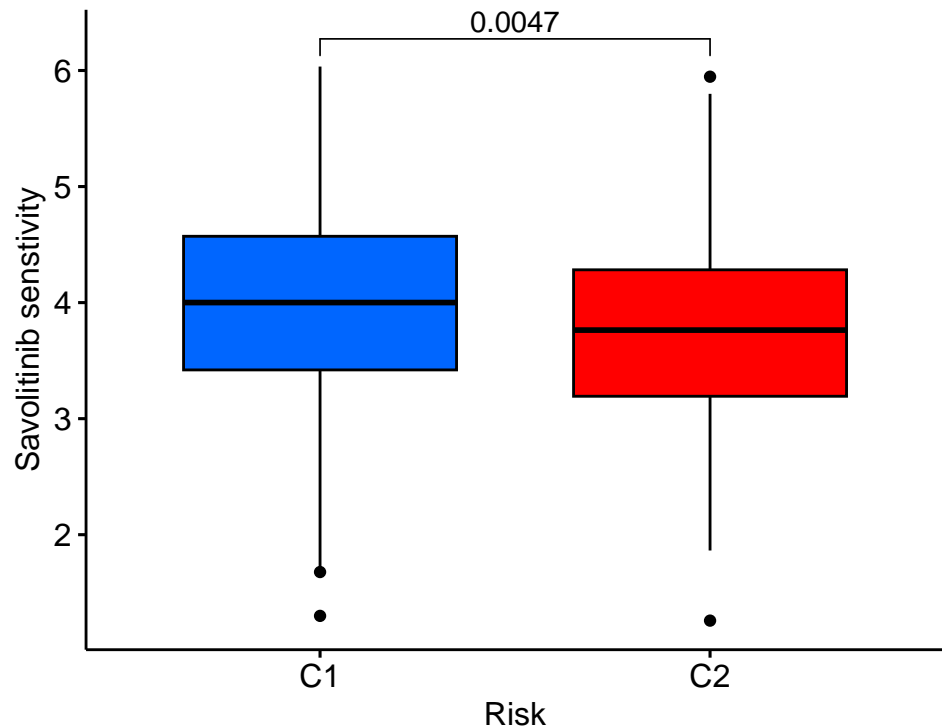

Supplement: Supplementary file 1 — Supplementary Information. [file 41598_2024_53257_MOESM1_ESM.zip › supplementary files/Drug sensitivity of C1 group and C2 group/C2 better/drugSenstivity.Savolitinib.pdf]

Risk C1 C2

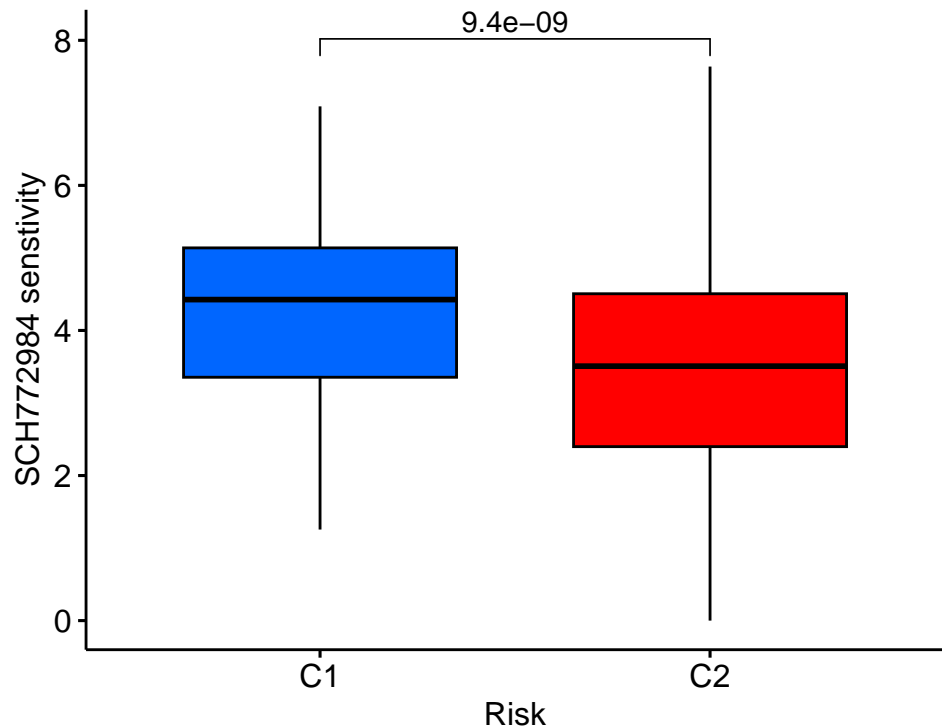

Supplement: Supplementary file 1 — Supplementary Information. [file 41598_2024_53257_MOESM1_ESM.zip › supplementary files/Drug sensitivity of C1 group and C2 group/C2 better/drugSenstivity.SCH772984.pdf]

Risk C1 C2

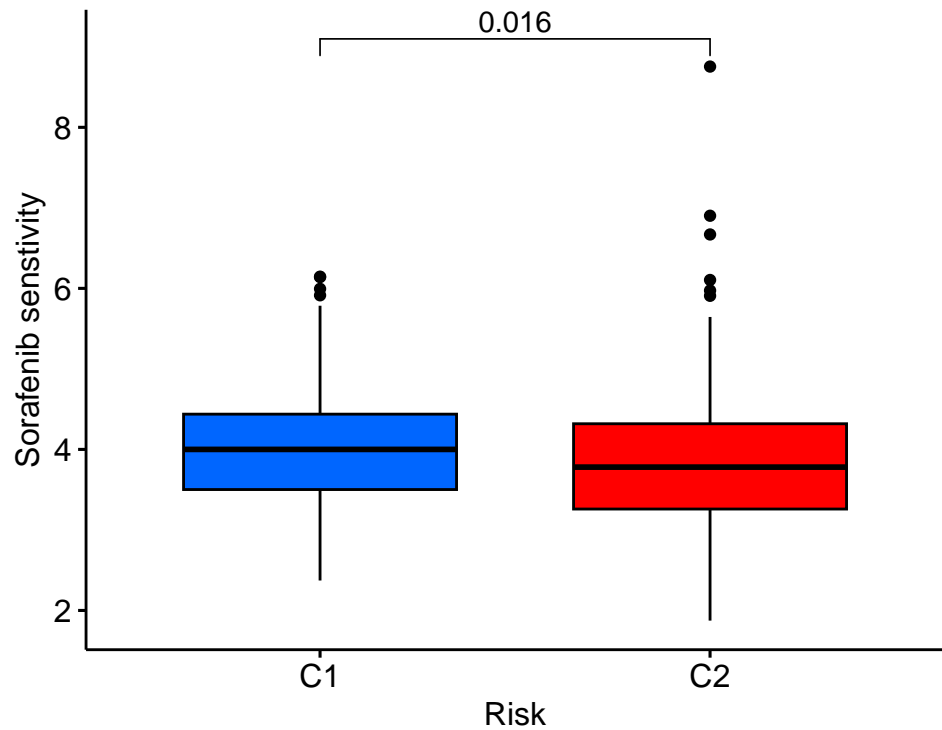

Supplement: Supplementary file 1 — Supplementary Information. [file 41598_2024_53257_MOESM1_ESM.zip › supplementary files/Drug sensitivity of C1 group and C2 group/C2 better/drugSenstivity.Sorafenib.pdf]

Risk C1 C2

6.9e-07

Staurosporine sensitivity

6

4

2

0

C1

C2

Risk

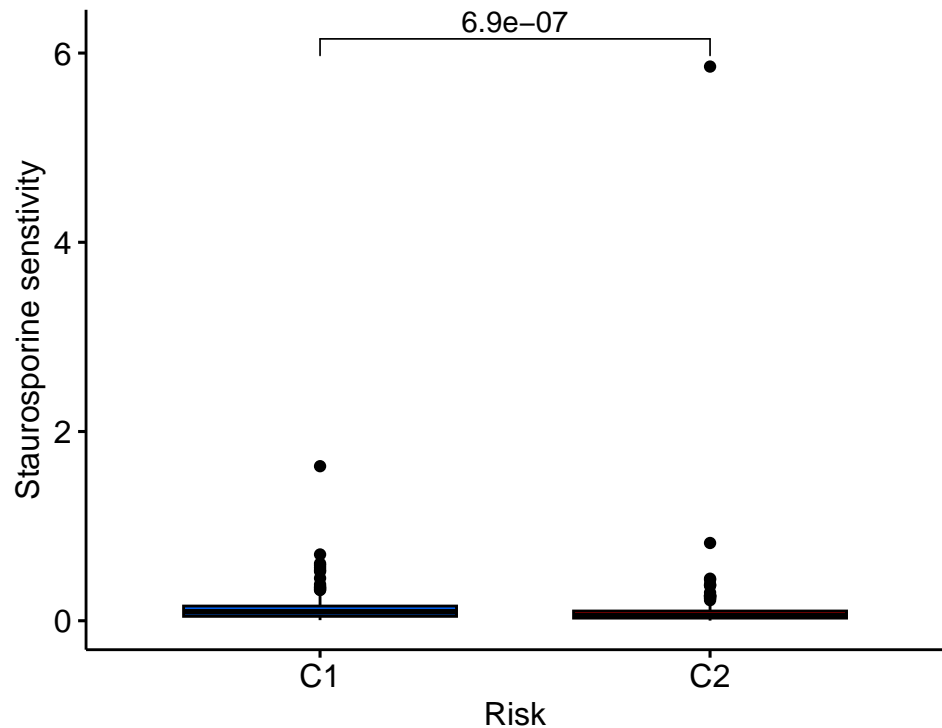

Supplement: Supplementary file 1 — Supplementary Information. [file 41598_2024_53257_MOESM1_ESM.zip › supplementary files/Drug sensitivity of C1 group and C2 group/C2 better/drugSenstivity.Staurosporine.pdf]

Risk C1 C2

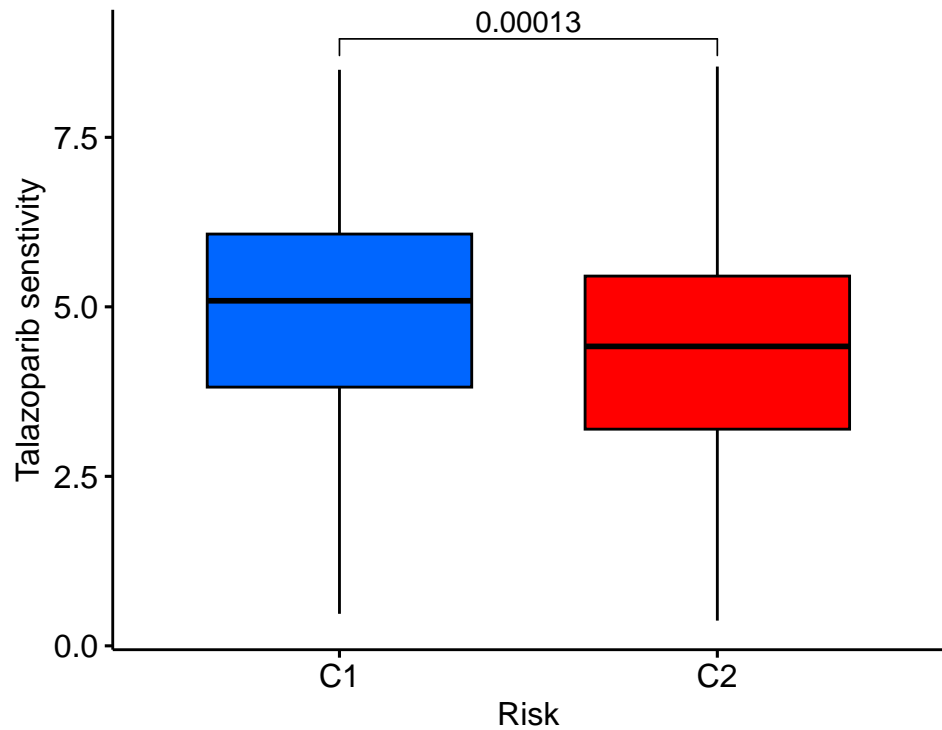

Supplement: Supplementary file 1 — Supplementary Information. [file 41598_2024_53257_MOESM1_ESM.zip › supplementary files/Drug sensitivity of C1 group and C2 group/C2 better/drugSenstivity.Talazoparib.pdf]

Risk C1 C2

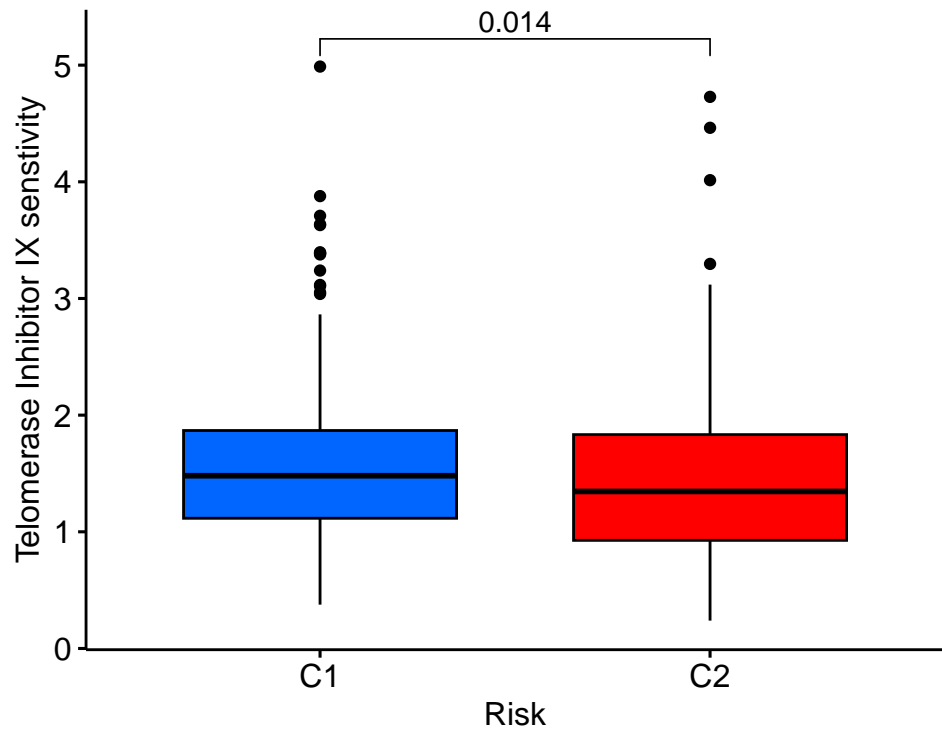

Supplement: Supplementary file 1 — Supplementary Information. [file 41598_2024_53257_MOESM1_ESM.zip › supplementary files/Drug sensitivity of C1 group and C2 group/C2 better/drugSenstivity.Telomerase Inhibitor IX.pdf]

Risk C1 C2

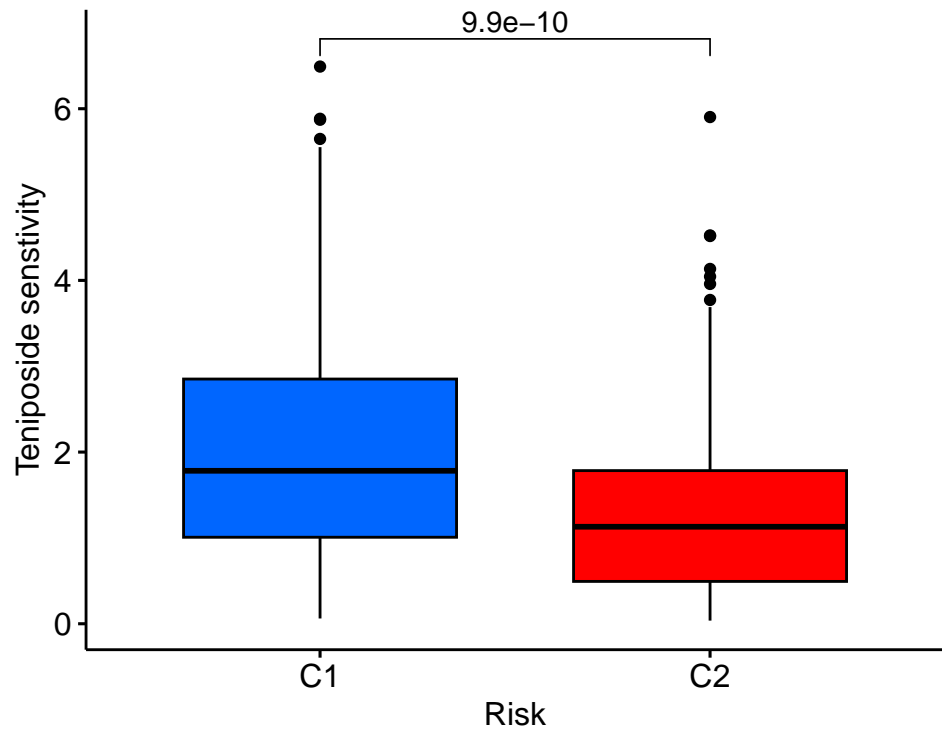

Supplement: Supplementary file 1 — Supplementary Information. [file 41598_2024_53257_MOESM1_ESM.zip › supplementary files/Drug sensitivity of C1 group and C2 group/C2 better/drugSenstivity.Teniposide.pdf]

Risk C1 C2

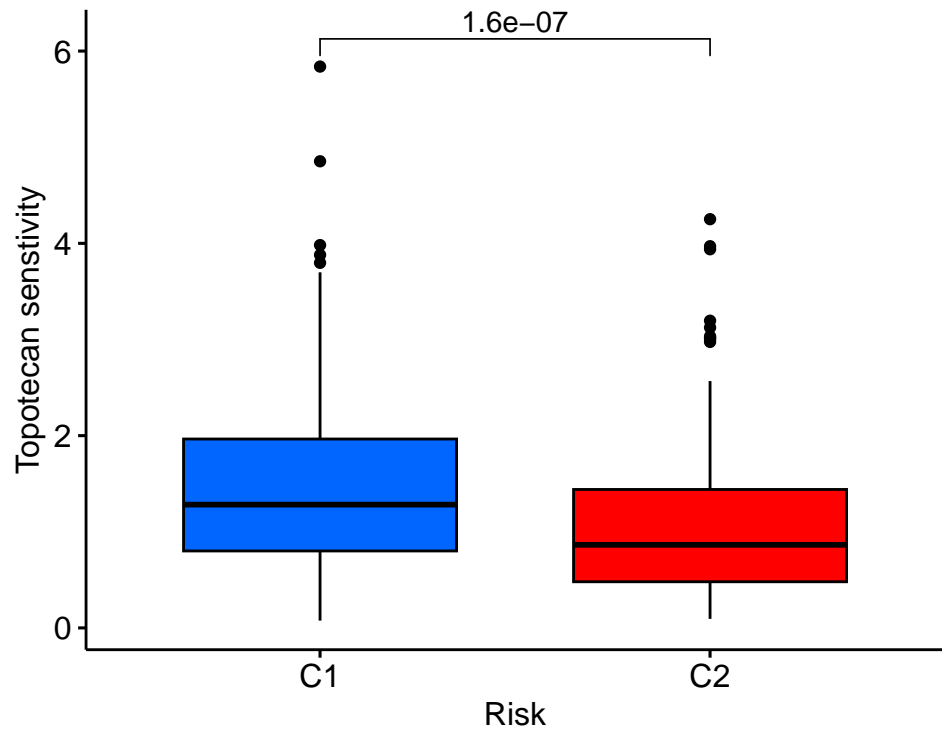

Supplement: Supplementary file 1 — Supplementary Information. [file 41598_2024_53257_MOESM1_ESM.zip › supplementary files/Drug sensitivity of C1 group and C2 group/C2 better/drugSenstivity.Topotecan.pdf]

Risk C1 C2

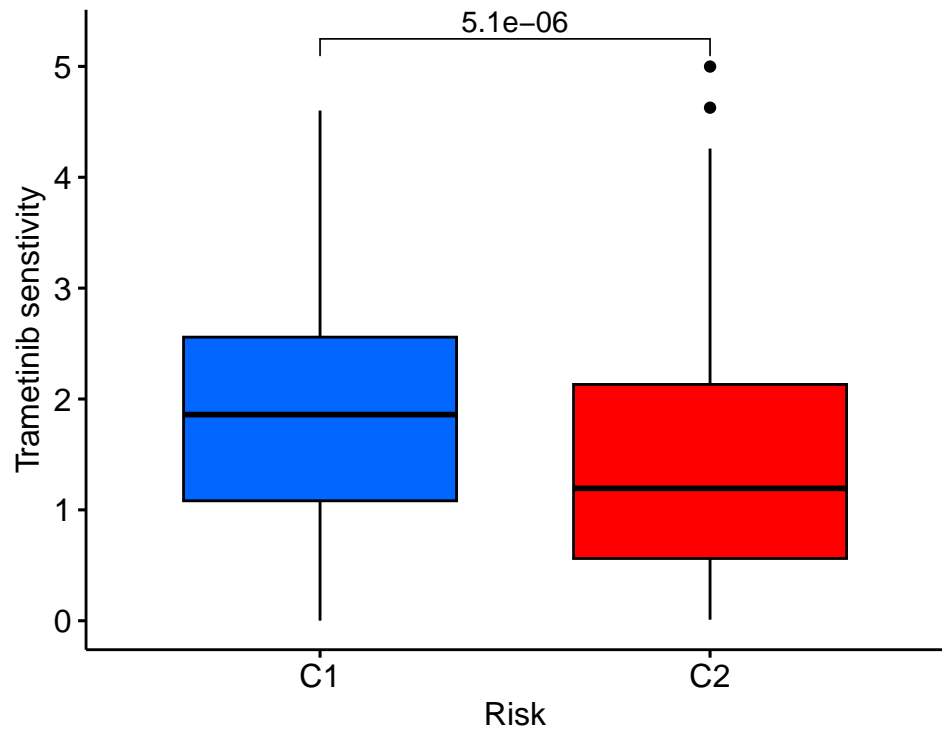

Supplement: Supplementary file 1 — Supplementary Information. [file 41598_2024_53257_MOESM1_ESM.zip › supplementary files/Drug sensitivity of C1 group and C2 group/C2 better/drugSenstivity.Trametinib.pdf]

Risk C1 C2

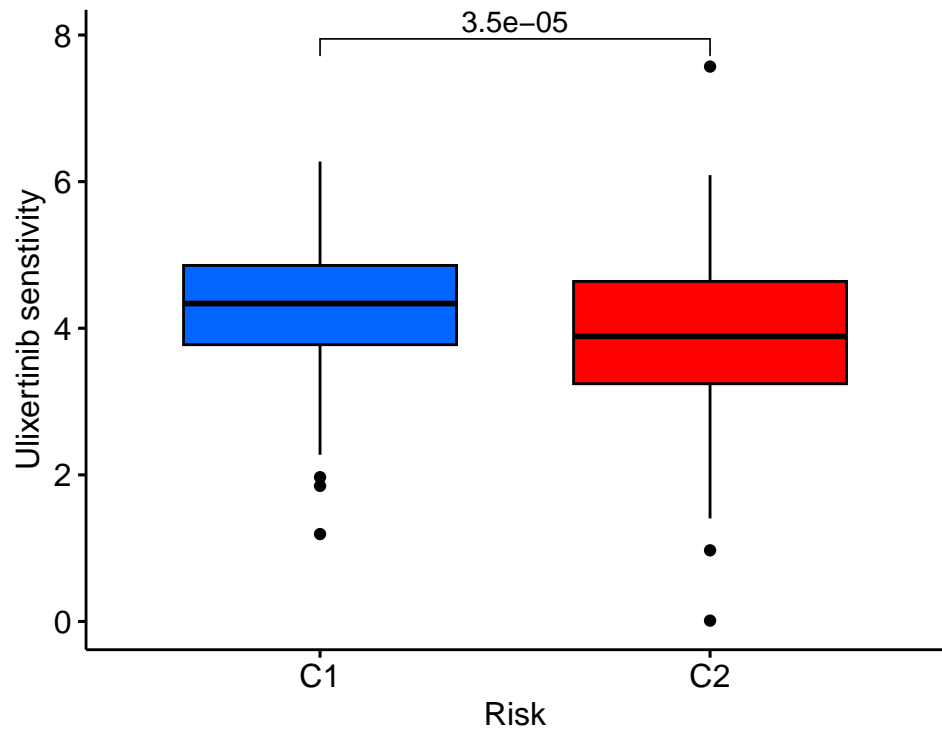

Supplement: Supplementary file 1 — Supplementary Information. [file 41598_2024_53257_MOESM1_ESM.zip › supplementary files/Drug sensitivity of C1 group and C2 group/C2 better/drugSenstivity.Ulixertinib.pdf]
