# Supplementary material for: Construction and validation of a colon cancer prognostic model based on tumor mutation burden-related genes
Source: Sci Rep. 2024 Feb 4;14:2867. doi: 10.1038/s41598-024-53257-z (PMC10838917; doi:10.1038/s41598-024-53257-z)
Supplement: Supplementary file 1 — Supplementary Information. [file drugSenstivity.Luminespib.pdf › supplementary files/Drug sensitivity of low and high risk group/high risk group better/drugSenstivity.Luminespib.pdf]

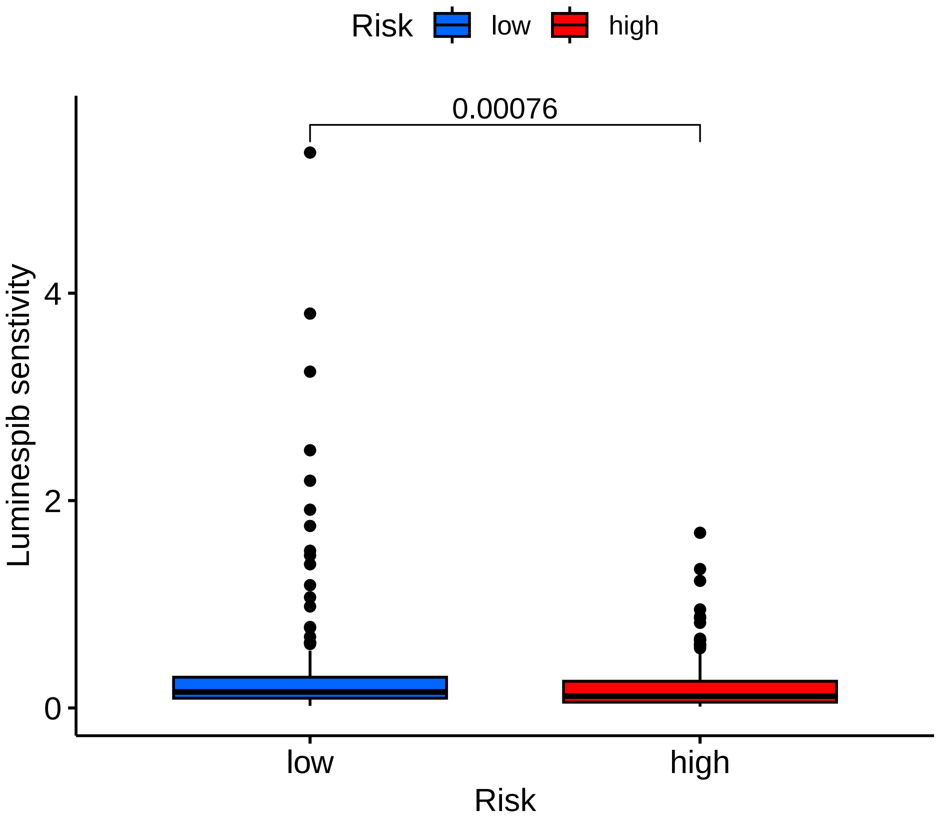
Risk
low
high
0.00076
Luminespib senstivity
4
2
0
low
high
Risk
